# ﻿A revision of the *wilsoni* species group in the millipede genus *Nannaria* Chamberlin, 1918 (Diplopoda, Polydesmida, Xystodesmidae)

**DOI:** 10.3897/zookeys.1096.73485

**Published:** 2022-04-15

**Authors:** Derek A. Hennen, Jackson C. Means, Paul E. Marek

**Affiliations:** 1 Virginia Polytechnic Institute and State University, Department of Entomology, Price Hall, 170 Drillfield Drive, Blacksburg, Virginia, USA Virginia Polytechnic Institute and State University Blacksburg United States of America

**Keywords:** Appalachia, Diplopoda, Nannariini, phylogeography, taxonomy

## Abstract

Although many new species of the millipede genus *Nannaria* Chamberlin, 1918 have been known from museum collections for over half a century, a systematic revision has not been undertaken until recently. There are two species groups in the genus: the *minor* species group and the *wilsoni* species group. In this study, the *wilsoni* species group was investigated. Specimens were collected from throughout its distribution in the Appalachian Mountains of the eastern United States and used for a multi-gene molecular phylogeny. The phylogenetic tree recovered *Nannaria* and the two species groups as monophyletic, with *Oenomaeapulchella* as its sister group. Seventeen new species were described, bringing the composition of the *wilsoni* species group to 24 species, more than tripling its known diversity, and increasing the total number of described *Nannaria* species to 78. The genus now has the greatest number of species in the family Xystodesmidae. Museum holdings of *Nannaria* were catalogued, and a total of 1,835 records used to produce a distribution map of the species group. Live photographs, illustrations of diagnostic characters, ecological notes, and conservation statuses are given. The *wilsoni* species group is restricted to the Appalachian region, unlike the widely-distributed *minor* species group (known throughout eastern North America), and has a distinct gap in its distribution in northeastern Tennessee and adjacent northwestern North Carolina. The *wilsoni* species group seems to be adapted to mesic microhabitats in middle to high elevation forests in eastern North America. New species are expected to be discovered in the southern Appalachian Mountains.

## ﻿Introduction

Millipedes in the family Xystodesmidae encompass a large component of the diplopod fauna in the deciduous forests of the Holarctic, with 539 currently known species (Marek et al. 2014; Means et al. 2021a, b; Mikhaljova 2021). Many species in the family are strikingly aposematic, with bold hues of yellow, orange, and red that are paired with black, thereby advertising their toxic chemical defenses of hydrogen cyanide and benzaldehyde (Marek and Bond 2009; Marek et al. 2018). While all members of the family are chemically defended, a few groups within the family do not appear to rely heavily on chemical defense and aposematism as a main form of defense. This is exemplified by the genus *Nannaria* Chamberlin, 1918, an assemblage of small-bodied (15–38 mm long) millipedes distributed in eastern North America. They are typically chestnut brown to black with a bimaculate pattern of orange to red, or white spots; uncommonly they may also have stripes.

The small size and comparatively subdued colors are atypical for xystodesmids, and previous authors have termed *Nannaria* as non-aposematic due to these traits, but speculated *Nannaria* may be mimicking colorless juveniles of aposematic xystodesmids (Whitehead and Shelley 1992). Additionally, *Nannaria* do not exhibit as much diurnal surface-level activity, traits typically associated with aposematic organisms, as other xystodesmids do, such as the apheloriine species *Apheloriapolychroma* (Marek et al. 2018). More commonly, *Nannaria* species are collected under forest detritus such as dead leaves and stones, or with passive trapping methods such as pitfall traps. They are difficult to deliberately collect through traditional methods of collecting xystodesmid millipedes (Hennen and Shelley 2015; Means et al. 2015). Part of this difficulty stems from their behavior: individuals tend to remain buried in the soil, even as adults. They either stay completely beneath the surface, or with only a portion of their body exposed above the soil.

The distribution of *Nannaria* extends from Arkansas and Missouri east through northern Mississippi to central North Carolina, and north to New York. Within that distribution, the region with the highest number of *Nannaria* species is the Appalachian Mountains of eastern North America. *Nannaria* is classified in the tribe Nannariini, which also includes the monotypic genus *Oenomaea* Hoffman, 1964, known from eastern Tennessee, northern Alabama, and northern Georgia. A recent molecular phylogenetic analysis of Xystodesmidae showed that the Nannariini was sister to a clade composed of the former families Euryuridae and Eurymerodesmidae (now tribes Euryurini and Eurymerodesmini) within the Xystodesmidae (Means et al. 2021a). This study showed that *Oenomaeapulchella* (Bollman, 1889) was sister to *Nannaria*, which was composed of two monophyletic species groups: the *minor* species group and the *wilsoni* species group. Means et al. (2021b), with additional species sampling within the genus, confirmed the monophyly of these species groups, and described 35 new species of the *minor* species group.

Despite the small size and cryptic behavior of *Nannaria*, millipede workers have described 62 species of Nannariini in two genera: *Nannaria* and *Oenomaea* (Marek et al. 2014; Hennen and Shelley 2015; Means et al. 2021a, b). The first species of *Nannaria* was described in 1847 by C. L. Koch, as *Fontariaoblonga* (C. L. Koch, 1847), with its type locality simply stated as Pennsylvania (Koch 1847). Two more species were described in the late 1800s (McNeill 1887; Bollman 1888), but the genus *Nannaria* had not been erected until the early 20^th^ century, with *Nannariaminor* (Chamberlin, 1918) designated as the type species (Chamberlin 1918). Later papers by Chamberlin (1928, 1940, 1947, 1949), Williams and Hefner (1928), Causey (1942, 1950a, b), Hoffman (1948, 1949a, b, 1950), Loomis and Hoffman (1948), Shelley (1975), Hennen and Shelley (2015), and Means et al. (2021a, b) added more species and characters to define the Nannariini. Previous species included in *Nannaria* have been transferred to other genera such as *Boraria* Chamberlin, 1943; *Howellaria* Hoffman, 1950; *Stenodesmus* De Saussure, 1859; and the genera *Castanaria* Causey, 1950 and *Mimuloria* Chamberlin, 1928, the latter two of which were later synonymized with *Nannaria*. The monophyly of the *minor* and *wilsoni* species groups in the tribe has been tested using molecular phylogenetics in Means et al. (2021a, b). However, the monophyly and phylogenetic placement of the tribe Nannariini has never been rigorously tested using molecular phylogenetics until recently (Marek and Bond 2006; Means et al. 2021a, b). The tribe Nannariini is diagnosed by the following main characters: sterna with lateral triangular spines; males with twisted, spatulate pre-gonopodal claws; gonopods with straight or curving acropodites; and a stout, long prefemoral process (Hoffman 1964; Hennen and Shelley 2015; Means et al. 2021b: figs 2, 8). For a detailed summary of the taxonomic history and diagnostic characters of the Nannariini, see Means et al. (2021b).

Previous authors have indicated that many undescribed species, and possibly even genera, remain to be found in the tribe (Hoffman 1999; Hennen and Shelley 2015; Marek et al. 2018), and this has been supported in a recent study of the *minor* species group, in which 35 new species of the genus were named (Means et al. 2021b). However, the *wilsoni* species group has not been investigated, and new species have been uncovered by examination of material in museum collections. Upon study of material from the Virginia Museum of Natural History amassed by Richard Hoffman and museum staff during decades of field collecting and molecular phylogenetics conducted over the past decade, two main clades within the genus were uncovered (Means et al. 2021a, b). One group, whose gonopod morphology was relatively simple, was referred to as the *Nannariaminor* species group, based on *N.minor*, the oldest name in the group (Means et al. 2021a, b). The second group, whose gonopod morphology was more complex due to the twists and additional processes on the acropodite branch, was labeled the *Nannariawilsoni* species group, based on the species *N.wilsoni* Hoffman, 1949. The *Nannariaminor* species group (hereafter referred to as the “*minor* species group”) is more widespread in eastern North America, found across the entire generic range, while the *Nannariawilsoni* species group (hereafter “*wilsoni* species group”) is restricted to the Appalachian Mountains, where it occupies two separated distributions, a northern and a southern portion.

Previous work on the *wilsoni* species group has been mostly limited to species descriptions. The first described species from the group was *N.scutellaria* (Causey 1942), followed by *N.morrisoni* (Hoffman 1948), *N.shenandoa* (Hoffman 1949a), *N.ericacea* and *N.wilsoni* (Hoffman 1949b), *N.austricola* (Hoffman 1950), and most recently, *N.aenigma* (Means et al. 2021a). The discovery of the *wilsoni* species group as a distinct clade from the *minor* species group was only recently proposed and supported by genetic evidence (Means et al. 2021a, b). The gonopod differences between species now included in the *wilsoni* and *minor* groups were vaguely noted by past workers (Hoffman 1948, 1949a; Hennen and Shelley 2015), but were not investigated further. The only notable non-taxonomic study on the *wilsoni* species group is a comparative morphological study of the gonopods of 17 specimens of *N.scutellaria* by Causey (1950), in which a variation in the angle of the acropodite tip and the number and shape of the distal projections of the acropodite were documented by the author.

With the discovery of 52 undescribed species in the two groups, the ambitious goal of describing them all was split between two projects. The species of the *minor* species group were treated in a first revision (Means et al. 2021b), while the species of the *wilsoni* species group are treated here. The aims of this study are to: (1) confirm the monophyly of the *wilsoni* species group using molecular phylogenetics, (2) describe new species of this species group, and (3) provide full taxonomic accounts for each species in the species group, including natural history notes and an investigation of the ecology of the species.

## ﻿Materials and methods

### ﻿Field collection

*Nannaria* specimens were collected by raking leaf litter and digging at/beneath the soil-litter interface in deciduous forest habitats throughout the eastern United States, according to the methods described in Means et al. (2015). Millipedes were collected with the goal of preserving material for both genetic and morphological analysis. Efforts were made to collect samples of all described species and any undescribed species represented in museum collections. *Nannaria* holdings from the Virginia Museum of Natural History (**VMNH**, Martinsville, Virginia), Virginia Tech Insect Collection (**VTEC**, Blacksburg, Virginia) and the North Carolina Museum of Natural Sciences (**NCSM**, Raleigh, North Carolina) were examined and georeferenced, if geographical coordinates were not originally provided, with the program GEOLocate (Rios 2018). Uncertainty estimates for the georeferenced coordinates were included as the most inclusive error radius that encompassed the recorded locality for each specimen. Natural history specimens were then databased with catalogue numbers beginning with the prefix NAN (e.g., NAN0001). The preceding museum material represented the bulk of *Nannaria* holdings worldwide, and provided locality data for putative new species. With this museum material, further *Nannaria* specimen records from the Symbiota Collections of Arthropods Network (**SCAN**) database (https://scan-bugs.org/) were acquired, including those from the National Parks Service’s Great Smoky Mountains National Park natural history collection (**GRSM**) and the Museum of Comparative Zoology, Harvard University (**MCZ**). All *wilsoni* species group specimens and records used for this study are listed in Suppl. material 1.

Sampling sites were selected based on the historical natural history collections material, with a focus on unsampled areas within the generic distribution. Field collecting took place in seven states: Georgia, Kentucky, North Carolina, South Carolina, Tennessee, West Virginia, and Virginia. Additional states in the eastern United States were visited, but yielded no specimens of the *wilsoni* species group. Scientific collecting permits were acquired for collecting and are listed in the acknowledgements section.

### ﻿Morphological investigation

After field collection of specimens, millipedes were processed using the workflow described by Means et al. (2021b). Millipedes were photographed on a moss background in the laboratory with a Canon EOS 6D camera and a 50-mm lens. They were then given a catalog number with the prefix MPE (e.g., MPE02123). Twenty-four legs were removed from the right side of the body and preserved in RNAlater or 100% ethanol and stored in a -80 °C freezer in the VTEC (https://www.collection.ento.vt.edu) for molecular analysis. The rest of the body was preserved in 70% isopropanol for morphological analysis and deposited in the VTEC. Measurements for the following six characters were taken from a male specimen of each species using a Leica M125 stereomicroscope, after Marek (2010), and all following measurements are given in millimeters: body length (**BL**), collum width (**CW**), intergenal width (**IW**), interantennal socket width (**ISW**), body ring 10 width (**B10W**), and body ring 10 height (**B10H**). Morphological coding and scoring of taxonomic characters for species descriptions and diagnoses was accomplished using the program Mesquite (Maddison and Maddison 2010) to produce a matrix of qualitative male and female exoskeletal and genitalic characters for species diagnosis, description, and comparison (Suppl. material 2 and 3). A total of 54 qualitative morphological characters (21 male external morphology characters, 25 gonopodal characters, eight cyphopodal characters) were scored for all species in the *wilsoni* species group, incorporating seven new and 47 previously-used taxonomic characters in morphological studies of Xystodesmidae (Marek and Bond 2006; Means et al. 2021a, b). The left gonopod of each species was photographed with a Canon 6D camera with a 65 mm Canon MP-E macro lens mounted on a Passport II Portable Digital Imaging System (Canon, Tokyo, Japan; Visionary Digital, Charlottesville, USA). Photographs were focus stacked with the program Helicon Focus Pro v.6.7.1 (HeliconSoft, Kharkiv, Ukraine), and the composite image was outlined in Adobe Illustrator CS6. Distribution maps were made using the program SimpleMappr (Shorthouse 2010) and Adobe Illustrator CS6. Type specimens of each new species were deposited in the Field Museum of Natural History (**FMNH**), the North Carolina Museum of Natural Sciences (**NCSM**), the Virginia Tech Insect Collection (**VTEC**), and the Virginia Museum of Natural History (**VMNH**). Type repositories for previously-described species include the Smithsonian Institution, National Museum of Natural History (**USNM**, Washington, D.C.), the Academy of Natural Sciences of Drexel University (**ANSP**, Philadelphia, Pennsylvania), and the Florida State Collection of Arthropods (**FSCA**, Gainesville, Florida).

For species description, we used a morphology based species delimitation criterion that species are diagnosable from others by a combination of unique characteristics. Under this criterion, a total of 17 new species were identified, in addition to the seven species already described in the *wilsoni* species group. Species delimitation followed criteria in Marek (2010) and Means et al. (2021a, b), and using the morphological data (i.e., comparison of gonopod shape), new species hypotheses were proposed.

### ﻿Phylogenetic inference

A phylogenetic tree of the genus *Nannaria*, including specimens of both the *minor* and *wilsoni* species groups, was estimated from two mitochondrial (16S, CO1) and four nuclear (28S, EF1a, fbox, and RPB1) gene regions using primers developed by Means et al. (2021b). Amplification procedures for the newly developed primers are available in Suppl. material 4, and procedures for previously developed primers can be found in Means and Marek (2017). Outgroup species were selected from other Nannariini genera (*O.pulchella*) and from the tribes most closely related to Nannariini, the Eurymerodesmini and Euryurini. Terminals of the genera *Eurymerodesmus* Brölemann, 1900 and *Euryurus* C. L. Koch, 1847 were used to root the tree. Genomic DNA was extracted from millipede legs and purified with a Qiagen DNeasy kit according to the manufacturer’s instructions. The DNA was then amplified via polymerase chain reaction and sequenced with a Sanger protocol, as in Means and Marek (2017) and Means et al. (2021b). Chromatograms were trimmed, bases called, and overlapping fragments assembled into contiguous sequences with the program Mesquite using phred and phrap in the Chromaseq module (Maddison and Maddison 2010; Ewing et al. 1998). The program PRANK (Probabilistic Alignment Kit, Löytynoja and Goldman 2005) was used for sequence alignment using the default gap opening and extension probabilities. Aligned sequences from PRANK were subsequently partitioned by gene and codon position (in protein coding genes) and by intron and exon boundaries (EF1a, RPB1) in Mesquite and then concatenated, following methods in Means et al. (2021b). IQ-TREE 2 (Version 2.0.6, Minh et al. 2020; Chernomor et al. 2016) was then used to estimate a phylogeny under maximum likelihood (ML) as an optimality criterion with 1,000 bootstrap pseudoreplicates. Node support values were inferred based on bootstrap sampling, and node support values greater than 70 were considered to be well-supported. ModelFinder (Kalyaanamoorthy et al. 2017) was used to select the best-fit model with edge-linked-proportional partition models. IQ-TREE 2 was also used to measure nucleotide frequencies and base composition and to perform a χ2-test for compositional heterogeneity for each gene alignment using only nannariine taxa, with a null hypothesis of homogeneity. To compare separate gene histories, individual gene trees were also estimated in IQ-TREE2 using the same methods as for the concatenated tree.

## ﻿Results

### ﻿Field collection

**Figure 1.**
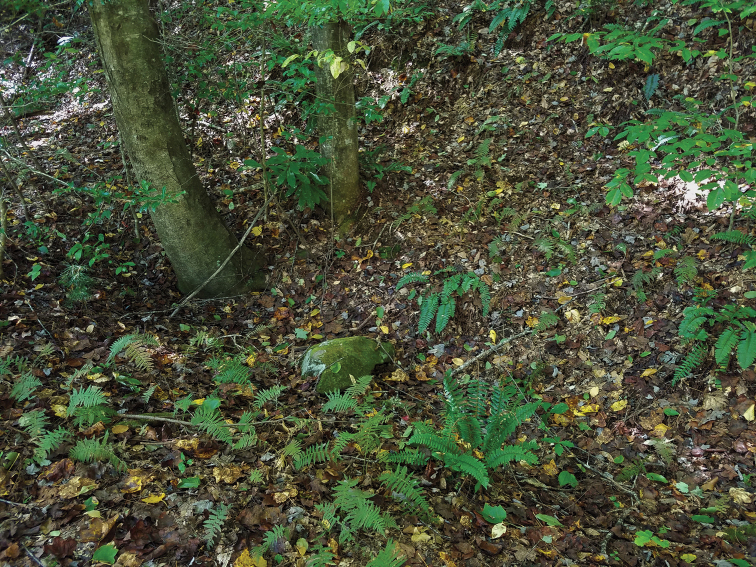
Typical habitat of species in the *Nannariawilsoni* group. This photograph shows a representative mesic forest during early fall, with a leaf litter layer comprised of tuliptree (*Liriodendrontulipifera*), hemlock (*Tsuga* sp.), alder (*Alnus* sp.), ironwood (*Carpinuscaroliniana*), and rhododendron (*Rhododendron* sp.). Henderson County, North Carolina.

In total, 1,835 *Nannaria* specimens and their specimen-level label records in the *wilsoni* group were examined for this study. Of these, 374 specimens were recently collected (i.e., between 2003 and 2019) and were deposited in the VTEC. Based on the field collections conducted, *Nannaria* specimens often exhibited a patchy distribution, with only a few individuals in each encounter. In this sense, it is unclear if this was a true reflection of their supposed low abundance compared to other syntopic millipedes, or a result of their comparatively cryptic habits (compared to other large xystodesmid millipedes). Their cryptic body coloration and burrowing behavior resulted in specimens being difficult to regularly collect, and when found, were most often partly buried in the soil, with only a small portion of their dorsum exposed above the soil. Due to this difficulty, sampling strategies were subsequently adjusted, with field collectors’ focus shifting towards habitats with abundant hemlock (*Tsuga* spp.) and rhododendron (*Rhododendron* spp.), as these plants provide shady, moist microhabitats in their understory (Rohr et al. 2009). Preference was also given to riparian habitats, which also tended to have cool, moist soil that *Nannaria* seemed to prefer. In addition to hemlock and rhododendron, the most commonly encountered plant species include: oaks (*Quercus* spp.), maples (*Acer* spp.), tuliptree (*Liriodendrontulipifera*), pawpaw (*Asiminatriloba*), witch hazel (*Hamamelis* spp.), birches (*Betula* spp.), American beech (*Fagusgrandifolia*), dogwoods (*Cornus* spp.), black cherry (*Prunusserotina*), hickories (*Carya* spp.), and sassafras (*Sassafrasalbidum*). An example of typical habitat for the *wilsoni* group is in Fig. 1, and descriptive habitat information for each specimen is given in Suppl. material 1.

**Figure 2.**
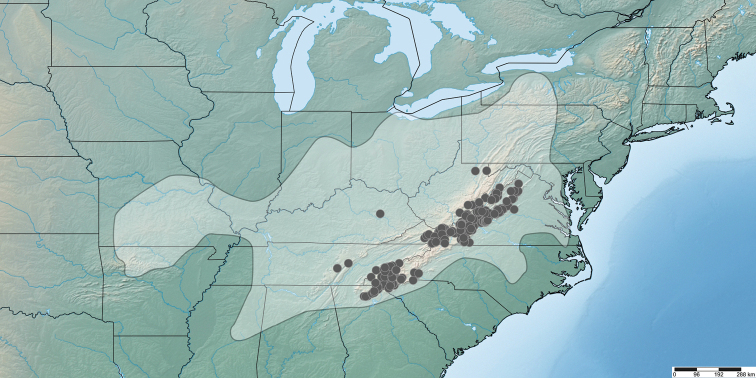
Distribution of the *Nannariawilsoni* species group. *Nannariawilsoni* species group specimens are indicated by black dots within the *Nannaria* generic distribution (white shaded region). Biogeographical cluster 1 (Central Appalachian Mountains Species Cluster) are the cluster of dots primarily in Virginia, and Biogeographical cluster 2 (Southern Appalachian Mountains Species Cluster) are those primarily in western North Carolina.

The *wilsoni* species group is restricted to the Appalachian region of the eastern United States, within the following U.S. Level III ecoregions (Omernik and Griffith 2014): Blue Ridge, Ridge and Valley, Southwestern Appalachians, Central Appalachians, and Western Allegheny Plateau (Fig. 2). The distribution of the *wilsoni* species group is split into two separate sections: the central Appalachians portion (West Virginia, Virginia, and Kentucky) and the southern Appalachians portion (North Carolina, South Carolina, Tennessee, and Georgia). There is a 140 km gap in the distribution in northeastern Tennessee and adjacent northwestern North Carolina. This gap was initially considered an artifact of collecting bias, but concerted collecting trips to this area revealed only *minor* group species, indicating that this is a true gap in the distribution.

Species of the *wilsoni* group are sympatric with the *minor* group throughout most of the *wilsoni* group’s distribution, except for northern Georgia and adjacent southwestern North Carolina. Outlying records of the *wilsoni* group in east-central Tennessee, eastern Kentucky, and northeastern West Virginia indicate that the *wilsoni* group likely has a larger distribution than is currently known, however these areas are poorly-collected for *Nannaria*.

### ﻿Molecular phylogenetics

Our phylogenetic analysis used a concatenated supermatrix which included six genes from 204 taxa and had a total length of 4,651 bp. Gene boundaries are as follows: 16S (1–845), COI (846–1,403), EF1a (1,404–2,098), 28S (2,099–3,193), RPB1 (3,194–4,254), fbox (4,255–4,651). These six genes were subdivided by ModelFinder into eight partitions. Of the 4,651 characters, 2,939 were constant, 691 were parsimony uninformative, and 1,021 were parsimony informative. Partition one contained 16S with TVM+F+I+G4 as its best-fit model, and had 351 parsimony informative characters. Partition two contained the first codon position of COI with TIM3+F+I+G4 as its best-fit model, and had 35 parsimony informative characters. Partition three contained the second codon position of COI and the first and second codon positions of RPB1 with TPM2+F+R3 as its best-fit model, and had 73 parsimony informative characters. Partition four contained the third codon position of COI with TIM3+F+I+G4 as its best-fit model, and had 166 parsimony informative characters. Partition five contained the first and second codon positions of both fbox and EF1a with TIM3e+R3 as its best-fit model, and had 19 parsimony informative characters. Partition six contained the third codon positions of both fbox and EF1a with TIM+F+R2 as its best-fit model, and had 95 parsimony informative characters. Partition seven contained the third codon position and intron of RPB1 and the intron of EF1a with HKY+F+R3 as its best-fit model, and had 219 parsimony informative characters. Partition eight contained 28S with TVM+F+R3 as its best-fit model, and had 63 parsimony informative characters. The observed mean base pair composition for the concatenated matrix was *A* = 0.238, *C* = 0.193, *G* = 0.263, and *T* = 0.306. Nucleotide base frequencies for 16S were *A* = 0.28, *C* = 0.07, *G* = 0.23, *T* = 0.43 and average gap/ambiguity was 16.09%. Nucleotide base frequencies for COI were *A* = 0.20, *C* = 0.14, *G* = 0.24, *T* = 0.42 and average gap/ambiguity was 2.11%. Nucleotide base frequencies for EF1a were *A* = 0.26, *C* = 0.23, *G* = 0.26, *T* = 0.26 and average gap/ambiguity was 22.98%. Nucleotide base frequencies for 28S were *A* = 0.15, *C* = 0.29, *G* = 0.36, *T* = 0.21 and average gap/ambiguity was 7.45%. Nucleotide base frequencies for RPB1 were *A* = 0.31, *C* = 0.17, *G* = 0.23, *T* = 0.29 and average gap/ambiguity was 8.36%. Nucleotide base frequencies for fbox were *A* = 0.23, *C* = 0.27, *G* = 0.28, *T* = 0.23 and average gap/ambiguity was 0.23%. No taxa were found to have failed the χ2 test for stationarity (P > 0.05, df = 3), and nucleotide frequency was found to be homogenous for each gene region for all taxa tested. Mean uncorrected percent difference of COI sequences between *Nannaria* species in the *wilsoni* group is 9.97% (maximum: 19.54%, minimum: 0%, standard deviation: 3.11%). The aligned sequences used in the phylogenetic analysis have been deposited in GenBank, and a complete list of sequences and associated accession numbers are given in Suppl. material 5.

The estimation of the phylogenetic history here tested recovered Nannariini as monophyletic, with the monophyletic genus *Nannaria* split into the two clades: *wilsoni* and *minor* species groups (Suppl. material 6).

**Figure 3.**
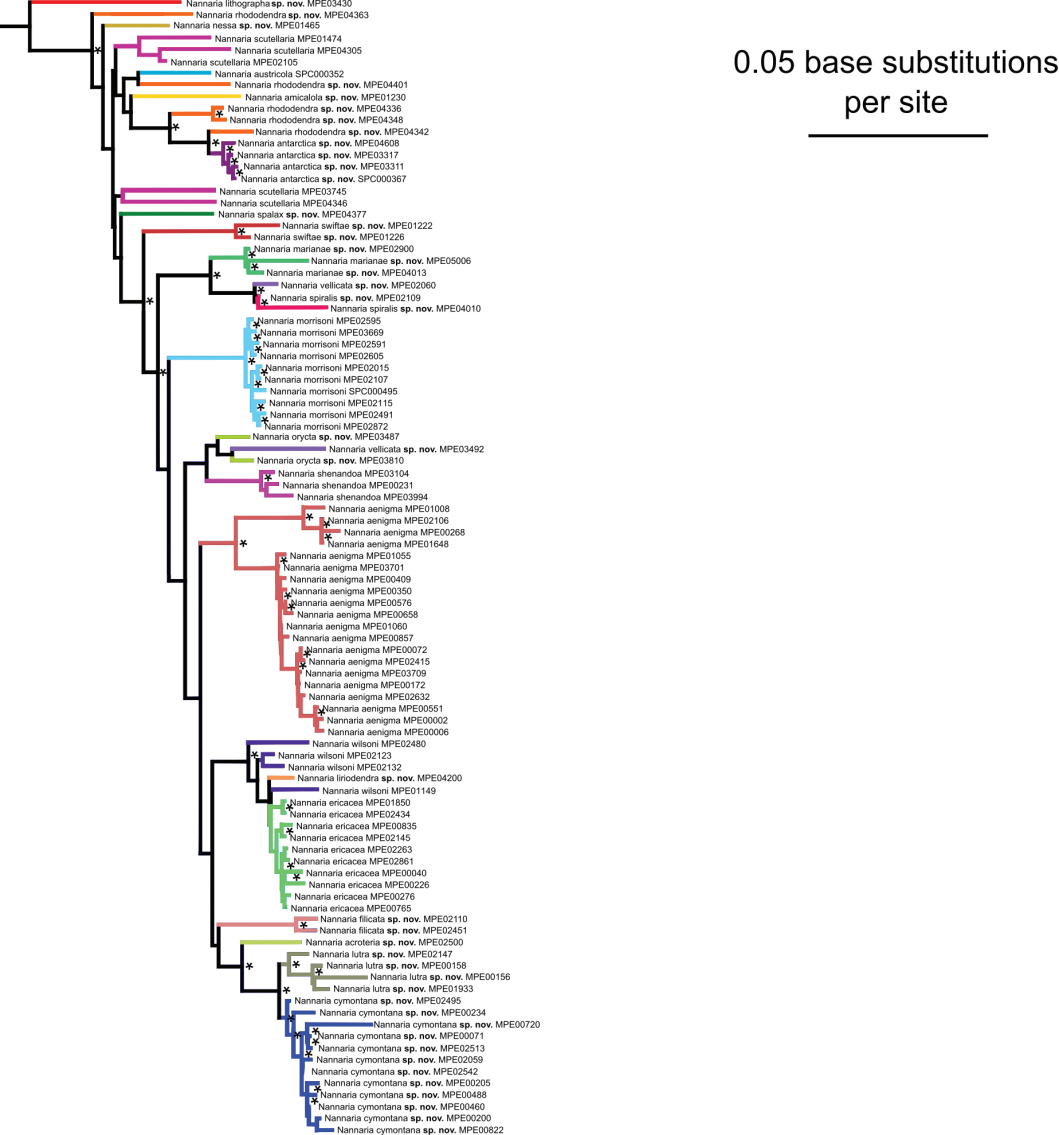
Maximum likelihood phylogeny of the *Nannariawilsoni* species group. Phylogeny estimated from the gene regions 16S, COI, EF1a, 28S, RPB1, and fbox. Branches colored by species identity. Bootstrap support values > 70 indicated by asterisks (*).

Both of the *Nannaria* species groups were recovered as monophyletic (bootstrap support = 79), and *O.pulchella* was sister to *Nannaria* (bootstrap support = 100). In each of the individual gene trees, the two species groups were only recovered as monophyletic and well-supported with bootstrap values > 70 in the EF1a tree (Suppl. material 7). Within the *wilsoni* group (Fig. 3), individual species clades were generally well-supported with bootstrap values > 70, though many species from the Southern Appalachian Mountains had lower bootstrap support. Higher-level relationships within the clade, however, were not strongly supported, with an average bootstrap support of 45. Average bootstrap support within the *wilsoni* group clade was 68, and 49 of 95 nodes had bootstrap support > 70. Species from the southern portion of the distribution were generally a paraphyletic grade in the phylogeny.

### ﻿Taxonomy

The following species accounts include 17 new species and seven previously described species, bringing the total number of species in the *wilsoni* group to 24. Gonopod illustrations in anterior and magnified posterior views for each species are given in Fig. 4, along with a labeled diagram of a representative gonopod. Species are listed in alphabetical order. In each diagnosis, the treated species is compared with other species in the *wilsoni* group that are geographically close and/or morphologically similar. The descriptions are included in a scored morphological matrix to facilitate straightforward species comparison (Suppl. material 2 and 3). Photographs of live specimens (when available) and gonopod illustrations in anterior, medial, and posterior views are given for each species in Figs 5–50. A key to species based on male specimens is provided after the species accounts.

**Figure 4.**
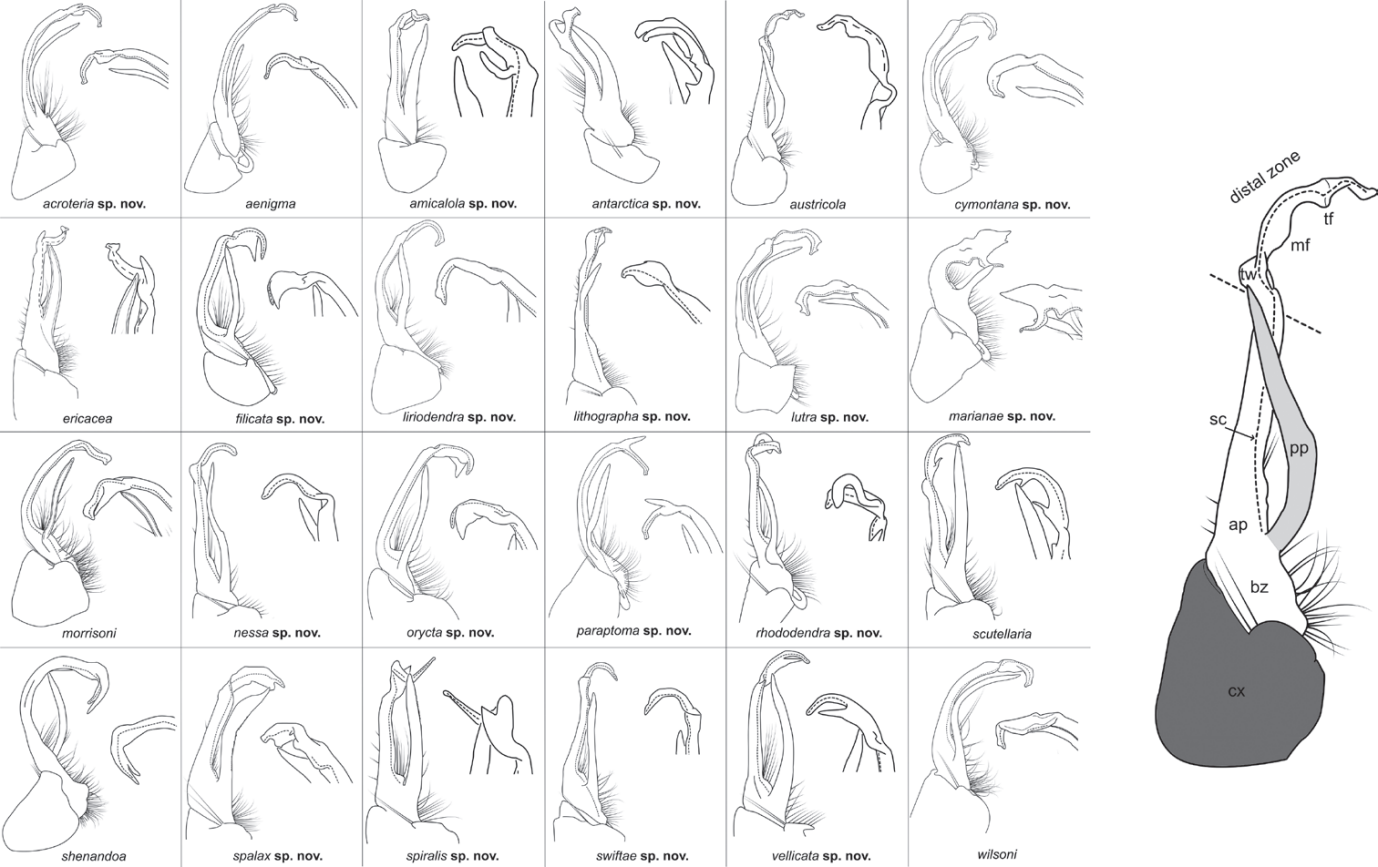
*Nannariawilsoni* species group left male acropodites: (left) anterior view and (right) magnified posterior view. At right, *N.austricola* gonopod diagram, anterior view, color-coded by region: coxa (dark grey), prefemur (light grey), and acropodite (white). A dashed line indicates the beginning of the distal zone region of the acropodite. Abbreviations: **ap**, acropodite; **bz**, basal zone; **cx**, coxa; **mf**, acropodite medial flange; **pp**, prefemoral process; **sc**, seminal canal; **tf**, acropodite tip medial flange; **tw**, anterior bend twist.

Morphological terms used in the species descriptions and diagnoses follow the precedent set by previous xystodesmid workers (e.g., Marek 2010; Means and Marek 2017, Means et al. 2021b). The gonopod terminology is defined here for clarity (Fig. 4). The coxa (**cx**) is the most proximal part of the gonopod to the body of the millipede, and is typically rounded, with a basal coxal apodeme for muscle attachment. On the mesal side of the gonopod is the cannula, a thin, hooked tube that connects to the base of the acropodite and forms the beginning of the seminal canal. The acropodite (**ap**) is the main gonopod branch and is large and falcate in the *wilsoni* species group. It may be entire or bifurcate distally, with various twists and modified flanges. At its base between the coxa (**cx**) and prefemoral process attachment, is the basal zone (**bz**), which may have a lateral bulge. The prefemoral process (**pp**) is the secondary branch of the gonopod and typically attaches at the base of the acropodite, and varies in form from acicular to curved. Medial to the prefemoral process is the seminal canal (**sc**), which begins at the tapered terminal apex of the cannula. The seminal canal is a thin, open groove within the acropodite that winds its way to the tip of the acropodite. In a few taxa, there is a quadrate basomedial process posterior to the seminal canal at the base of the acropodite (Fig. 44A). The anterior bend of the gonopod marks the beginning of the distal zone of the gonopod, and is where the acropodite curves. The anterior bend in the *wilsoni* species group typically has a twist (**tw**), which can be weak (a slightly helical twist) to strong (a concave pinch or crimping of the acropodite). Immediately after the anterior bend is the medial flange (**mf**) of the acropodite, a structure on the medial side of the gonopod that can be variously modified into several different shapes. Distal to the medial flange is the acropodite tip medial flange (**tf**), which can also take a multitude of forms, and is separated from the medial flange by its location closer to the tip of the acropodite than to the anterior bend. The acropodite tip lateral flange is a modified flange distally on the lateral side of the gonopod, immediately before the tip.

### ﻿Nannaria taxonomy

#### Class Diplopoda de Blainville in Gervais, 1844

**Infraclass Helminthomorpha Pocock, 1887**

**Order Polydesmida Leach, 1815**

**Family Xystodesmidae Cook, 1895**

##### Subfamily Rhysodesminae Brolemann, 1916

###### ﻿Tribe Nannariini

Taxon classificationAnimaliaPolydesmidaXystodesmidae

Hoffman, 1964

48A57B60-F980-5C03-8814-0F171BAD6010

####### Tribe diagnosis.

The genera *Nannaria* and *Oenomaea* (tribe Nannariini) can be separated from closely related genera by the following combination of characters. The tribe Nannariini have triangular sternal projections laterally on the sterna, while non-Nannariini lack lateral sternal projections but may have rounded sternal lobes (as in *Pleuroloma* Rafinesque, 1820) or “scooped out” sterna (as in *Gyalostethus* Hoffman, 1965) or unmodified sterna (as in Apheloriini). Males with pregonopodal claws twisted and spatulate; non-Nannariini males with pregonopodal claws simple, bisinuate (Means et al. 2021a: fig. 16H). Gonopods with straight or gently curving acropodites and long prefemoral processes; gonopods never with circular or sigmoid acropodites as in *Apheloria* Chamberlin, 1921 or *Sigmoria* Chamberlin, 1939, and gonopods without small, hooked prefemoral processes as in *Apheloria*, and never lacking a prefemoral process completely as in some Apheloriini. Size small to moderate (body length 15–38 mm). Dorsum chestnut brown to black with orange to red, or sometimes white, paranotal spots, sometimes with orange to red stripes connecting the spots.

###### ﻿Genus Nannaria

Taxon classificationAnimaliaPolydesmidaXystodesmidae

Chamberlin, 1918

F4C0ECE3-E977-59CA-B232-CDA5BA6294CB

Nannaria Chamberlin, 1918: 124. Attems 1938: 199. Chamberlin and Hoffman 1958: 39. Hoffman 1964: 33. Jeekel 1971: 274. Hoffman 1980: 159. Hoffman 1999: 365 (349 in pdf version). Shelley et al. 2000: 113. Hennen and Shelley 2015: 16. Means et al. 2021a: 16. Means et al. 2021b: 18.
Mimuloria Chamberlin, 1928: 155. Chamberlin and Hoffman 1958: 37. Hoffman 1964: 33. Jeekel 1971: 273. Hennen and Shelley 2015: 5. Means et al. 2021a: S65.
Castanaria Causey, 1950c: 1. Chamberlin and Hoffman 1958: 37.

####### Type species.

*Nannariaminor* Chamberlin, 1918, by original designation. Taxa included: 78, see Table 1.

**Table 1.** List of all *Nannaria* species. Species are listed in alphabetical order, with species group listed alongside each species.

No.	Species	Species group
1	*N.acroteria* sp. nov.	*wilsoni* group
2	*N.aenigma* Means, Hennen & Marek, 2021	*wilsoni* group
3	*N.alpina* Means, Hennen & Marek, 2021	*minor* group
4	*N.ambulatrix* Means, Hennen & Marek, 2021	*minor* group
5	*N.amicalola* sp. nov.	*wilsoni* group
6	*N.antarctica* sp. nov.	*wilsoni* group
7	*N.asta* Means, Hennen & Marek, 2021	*minor* group
8	*N.austricola* Hoffman, 1950	*wilsoni* group
9	*N.blackmountainensis* Means, Hennen & Marek, 2021	*minor* group
10	*N.bobmareki* Means, Hennen & Marek, 2021	*minor* group
11	*N.botrydium* Means, Hennen & Marek, 2021	*minor* group
12	*N.breweri* Means, Hennen & Marek, 2021	*minor* group
13	*N.castanea* (McNeill, 1887)	*minor* group
14	*N.castra* Means, Hennen & Marek, 2021	*minor* group
15	*N.caverna* Means, Hennen & Marek, 2021	*minor* group
16	*N.cingulata* Means, Hennen & Marek, 2021	*minor* group
17	*N.conservata* Chamberlin, 1940	*minor* group
18	*N.cryomaia* Means, Hennen & Marek, 2021	*minor* group
19	*N.cymontana* sp. nov.	*wilsoni* group
20	*N.daptria* Means, Hennen & Marek, 2021	*minor* group
21	*N.davidcauseyi* Causey, 1950	*minor* group
22	*N.dilatata* (Hennen & Shelley, 2015)	*minor* group
23	*N.domestica* Shelley, 1975	*minor* group
24	*N.equalis* Chamberlin, 1949	*minor* group
25	*N.ericacea* Hoffman, 1949	*wilsoni* group
26	*N.filicata* sp. nov.	*wilsoni* group
27	*N.fowleri* Chamberlin, 1947	*minor* group
28	*N.fracta* Means, Hennen & Marek, 2021	*minor* group
29	*N.fritzae* Means, Hennen & Marek, 2021	*minor* group
30	*N.hardeni* Means, Hennen & Marek, 2021	*minor* group
31	*N.hippopotamus* Means, Hennen & Marek, 2021	*minor* group
32	*N.hokie* Means, Hennen, & Marek, 2021	*minor* group
33	*N.honeytreetrailensis* Means, Hennen & Marek, 2021	*minor* group
34	*N.ignis* Means, Hennen & Marek, 2021	*minor* group
35	*N.kassoni* Means, Hennen & Marek, 2021	*minor* group
36	*N.komela* Means, Hennen & Marek, 2021	*minor* group
37	*N.laminata* Hoffman, 1949	*minor* group
38	*N.liriodendra* sp. nov.	*wilsoni* group
39	*N.lithographa* sp. nov.	*wilsoni* group
40	*N.lutra* sp. nov.	*wilsoni* group
41	*N.marianae* sp. nov.	*wilsoni* group
42	*N.mcelroyorum* Means, Hennen & Marek, 2021	*minor* group
43	*N.minor* Chamberlin, 1918	*minor* group
44	*N.missouriensis* Chamberlin, 1928	*minor* group
45	*N.monsdomia* Means, Hennen & Marek, 2021	*minor* group
46	*N.morrisoni* Hoffman, 1948	*wilsoni* group
47	*N.nessa* sp. nov.	*wilsoni* group
48	*N.oblonga* (Koch, 1847)	*minor* group
49	*N.ohionis* Loomis & Hoffman, 1948	*minor* group
50	*N.orycta* sp. nov.	*wilsoni* group
51	*N.paraptoma* sp. nov.	*wilsoni* group
52	*N.paupertas* Means, Hennen & Marek, 2021	*minor* group
53	*N.piccolia* Means, Hennen & Marek, 2021	*minor* group
54	*N.rhododendra* sp. nov.	*wilsoni* group
55	*N.rhysodesmoides* (Hennen & Shelley, 2015)	*minor* group
56	*N.rutherfordensis* Shelley, 1975	*minor* group
57	*N.scholastica* Means, Hennen & Marek, 2021	*minor* group
58	*N.scutellaria* Causey, 1942	*wilsoni* group
59	*N.serpens* Means, Hennen & Marek, 2021	*minor* group
60	*N.sheari* Means, Hennen & Marek, 2021	*minor* group
61	*N.shenandoa* Hoffman, 1949	*wilsoni* group
62	*N.sigmoidea* (Hennen & Shelley, 2015)	*minor* group
63	*N.simplex* Hoffman, 1949	*minor* group
64	*N.solenas* Means, Hennen & Marek, 2021	*minor* group
65	*N.spalax* sp. nov.	*wilsoni* group
66	*N.spiralis* sp. nov.	*wilsoni* group
67	*N.spruilli* Means, Hennen & Marek, 2021	*minor* group
68	*N.stellapolis* Means, Hennen & Marek, 2021	*minor* group
69	*N.stellaradix* Means, Hennen & Marek, 2021	*minor* group
70	*N.suprema* Means, Hennen & Marek, 2021	*minor* group
71	*N.swiftae* sp. nov.	*wilsoni* group
72	*N.tasskelsoae* Means, Hennen & Marek, 2021	*minor* group
73	*N.tennesseensis* (Bollman, 1888)	*minor* group
74	*N.tenuis* Means, Hennen & Marek, 2021	*minor* group
75	*N.terricola* (Williams & Hefner, 1928)	*minor* group
76	*N.tsuga* Means, Hennen & Marek, 2021	*minor* group
77	*N.vellicata* sp. nov.	*wilsoni* group
78	*N.wilsoni* Hoffman, 1949	*wilsoni* group

#### Taxa included in the *wilsoni* species group

*Nannariaacroteria* sp. nov.

*Nannariaaenigma* Means, Hennen, & Marek, 2021

*Nannariaamicalola* sp. nov.

*Nannariaantarctica* sp. nov.

*Nannariaaustricola* Hoffman, 1950

*Nannariacymontana* sp. nov.

*Nannariaericacea* Hoffman, 1949

*Nannariafilicata* sp. nov.

*Nannarialiriodendra* sp. nov.

*Nannarialithographa* sp. nov.

*Nannarialutra* sp. nov.

*Nannariamarianae* sp. nov.

*Nannariamorrisoni* Hoffman, 1948

*Nannarianessa* sp. nov.

*Nannariaorycta* sp. nov.

*Nannariaparaptoma* sp. nov.

*Nannariarhododendra* sp. nov.

*Nannariascutellaria* Causey, 1942

*Nannariashenandoa* Hoffman, 1949

*Nannariaspalax* sp. nov.

*Nannariaspiralis* sp. nov.

*Nannariaswiftae* sp. nov.

*Nannariavellicata* sp. nov.

*Nannariawilsoni* Hoffman, 1949

**Genus diagnosis.** See diagnosis in Means et al. (2021b).

**Nannariawilsoni species-group diagnosis.** The *minor* species group and *wilsoni* species group can be separated by the following characters: Acropodite setae extending to at most halfway up length of acropodite (except in *N.lithographa* sp. nov.), and not reaching distal zone as in the *minor* species group. Anterior bend of acropodite typically with a gently curving or abrupt helical twist, not absent as in the *minor* species group. Prefemoral process arising from the base of the acropodite, or rarely, from the side of the acropodite, but never from an acropodite shelf as in the *minor* species group. Females often with cyphopod receptacle modified or enlarged; the cyphopod receptacle is unmodified in the *minor* species group. Female 2^nd^ leg coxae laterally expanded, partly or entirely covering cyphopod aperture, female 2^nd^ leg coxae not expanded in the *minor* species group.

### ﻿*Nannariawilsoni* species group

#### ﻿ Nannaria acroteria sp. nov.

Taxon classificationAnimaliaPolydesmidaXystodesmidae

BB6BFDCD-2A69-5BE4-B479-52D142141DAF

http://zoobank.org/0E1B503C-4F84-48B2-8EA1-81FDD44D91CD

**Figure 5.**
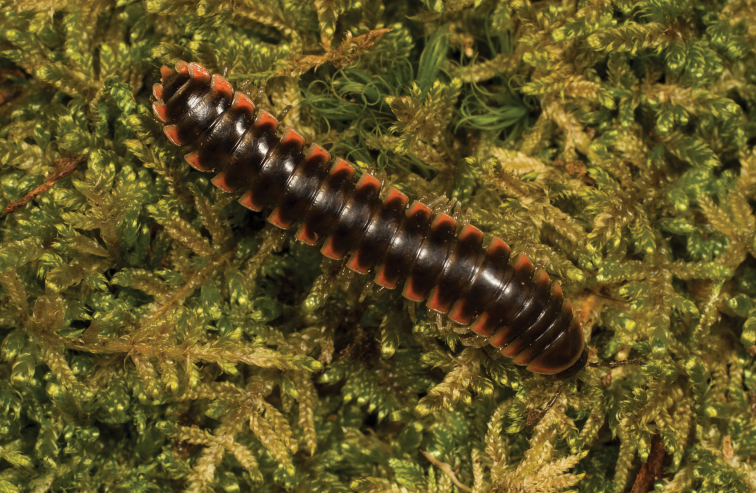
*Nannariaacroteria* sp. nov., in situ, male holotype (MPE02500) from Giles County, Virginia.

**Figure 6.**
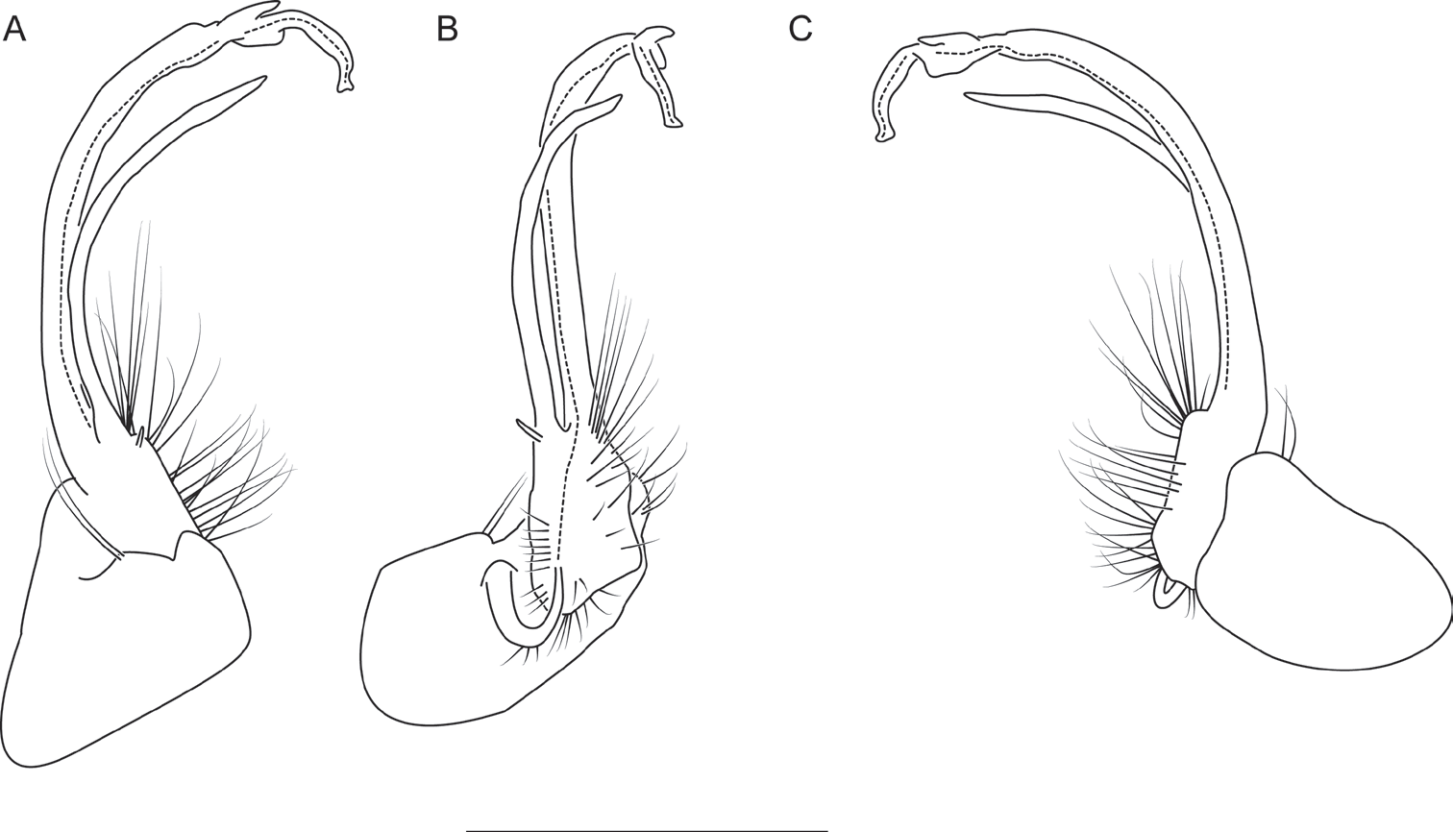
Left gonopod of *Nannariaacroteria* sp. nov. male holotype (MPE02500, Giles County, Virginia) **A** anterior view **B** medial view **C** posterior view. Scale bar: 1 mm.

Vernacular name: The Fork Mountain Twisted-Claw Millipede Figs 5 , 6

##### Material examined.

**Type material: *Holotype***: United States – **Virginia** • ♂; Giles County, Kire, Jefferson National Forest, in gully beside North Fork Mountain Rd; 37.4493°N, -80.5201°W, ± 6m; elev. 876 m; 27 April 2017; D. A. Hennen, J. C. Means, P. E. Marek, P. Shorter, V. Wong leg.; gully with small stream, litter moderately moist, rhododendron, oak, maple, mountain laurel, some pine, rocky, with large logs; VTEC, MPE02500. ***Paratypes***: United States – **Virginia** • 2 ♀♀; same collection data as for holotype; VTEC, MPE02564, MPE02567. **Non type material**: United States – **West Virginia** • 1 ♂; Greenbrier County, SW end of Kate’s Mtn. summit Picnic Area; 37.7638°N, -80.302°W, ± 3000m; 24 June 1967; W. A. Shear leg.; VMNH, NAN0025 • 2 specimens; Greenbrier County, Greenbrier State Forest, along 3 mi. of Young’s Nature Trail; 37.7394°N, -80.3336°W; 28 August 1973; W. A. Shear leg.; VMNH, NAN0029 • 1 ♂; Greenbrier County, Kate’s Mtn. summit; 37.7638°N, -80.302°W, ± 3000m; 10 April 1969; W. A. Shear leg.; VMNH, NAN0032 • 2 specimens; Greenbrier County, 8.5 miles W of Lewisburg; 37.8156°N, -80.5913°W, ± 5000m; 23 September 1949; J. P. E. Morrison leg.; VMNH, NAN0422 • 1 ♀; Monroe County, CR-6/2, 0.25 rd km S Greenbrier Co.; 7.6882°N, -80.3644°W; elev. 816.5 m; 18 July 2005; P. E. Marek, C. Spruill leg.; VTEC, SPC000674. Complete material examined information listed in Suppl. material 1.

##### Diagnosis.

Adults of *Nannariaacroteria* sp. nov. can be separated from the geographically close and morphologically similar species *N.lutra* sp. nov. and *N.aenigma* by the following characters. Prefemoral process sinuous and strongly curving, rather than acicular or slightly curving as in *N.aenigma*. Acropodite dorsal projection triangular, rather than rounded as in *N.lutra* sp. nov. Acropodite tip lateral flange absent, rather than bilobed as in *N.lutra* sp. nov. Additionally, *Nannariaacroteria* sp. nov. has a triangular process at the base of its prefemoral process, which is absent in the other two species.

##### Description.

Suppl. material 2. Based on holotype (♂) MPE02500 and paratype (♀) MPE02564.

**Measurements**: Taken from holotype (♂) MPE02500: BL = 27.80, CW = 3.96, IW = 2.76, ISW = 0.81, B10W = 4.75, B10H = 2.68. **Color.** Tergites with two paranotal red spots, collum outlined in red, and tergites with background black (Fig. 5). **Gonopods**. Male gonopod acropodite arc gradually curving (Fig. 6A–C). Acropodite with smoothly undulating twist at anterior bend (Fig. 6C), and acropodite medial flange lobed and with a medium-sized dorsal, triangular tooth (Fig. 6C). Acropodite tip medial and lateral flange both absent. Acropodite tip entire, directed ventrally (Fig. 6B). Prefemoral process sinuous (Fig. 6A) and curving medially (Fig. 6B). Prefemoral process tip directed medially, length almost as long as acropodite. Prefemoral process beset at base with a short, thin triangular process (Fig. 6A, B). **Cyphopods.** Female cyphopod receptacle triangular.

##### Variation.

No notable variation observed.

##### Distribution.

Only known from the type locality in Giles County, Virginia and nearby localities in Greenbrier and Monroe counties, West Virginia (Fig. 51).

##### Ecological notes.

The type locality is a rocky mesic mixed forest with oak, maple, rhododendron, mountain laurel, and pine, bisected by a stream. The elevation of the type locality is 876 m.

##### Etymology.

This species is named for the small projection at the base of the prefemoral process, a unique trait within the *wilsoni* species group. The specific name is a feminine adjective derived from the Latin *acroterium*, meaning a small pedestal or projection.

#### ﻿ Nannaria aenigma

Taxon classificationAnimaliaPolydesmidaXystodesmidae

Means, Hennen & Marek, 2021

9C0C911C-4943-5656-BCA2-71F5E70A3227

**Figure 7.**
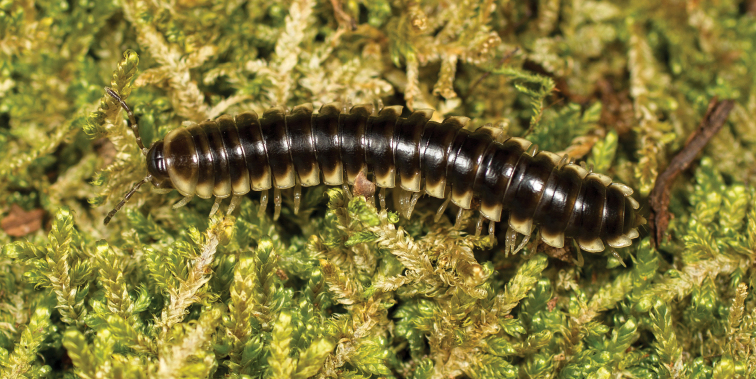
*Nannariaaenigma*, in situ, male holotype (MPE02632) from Smyth County, Virginia.

**Figure 8.**
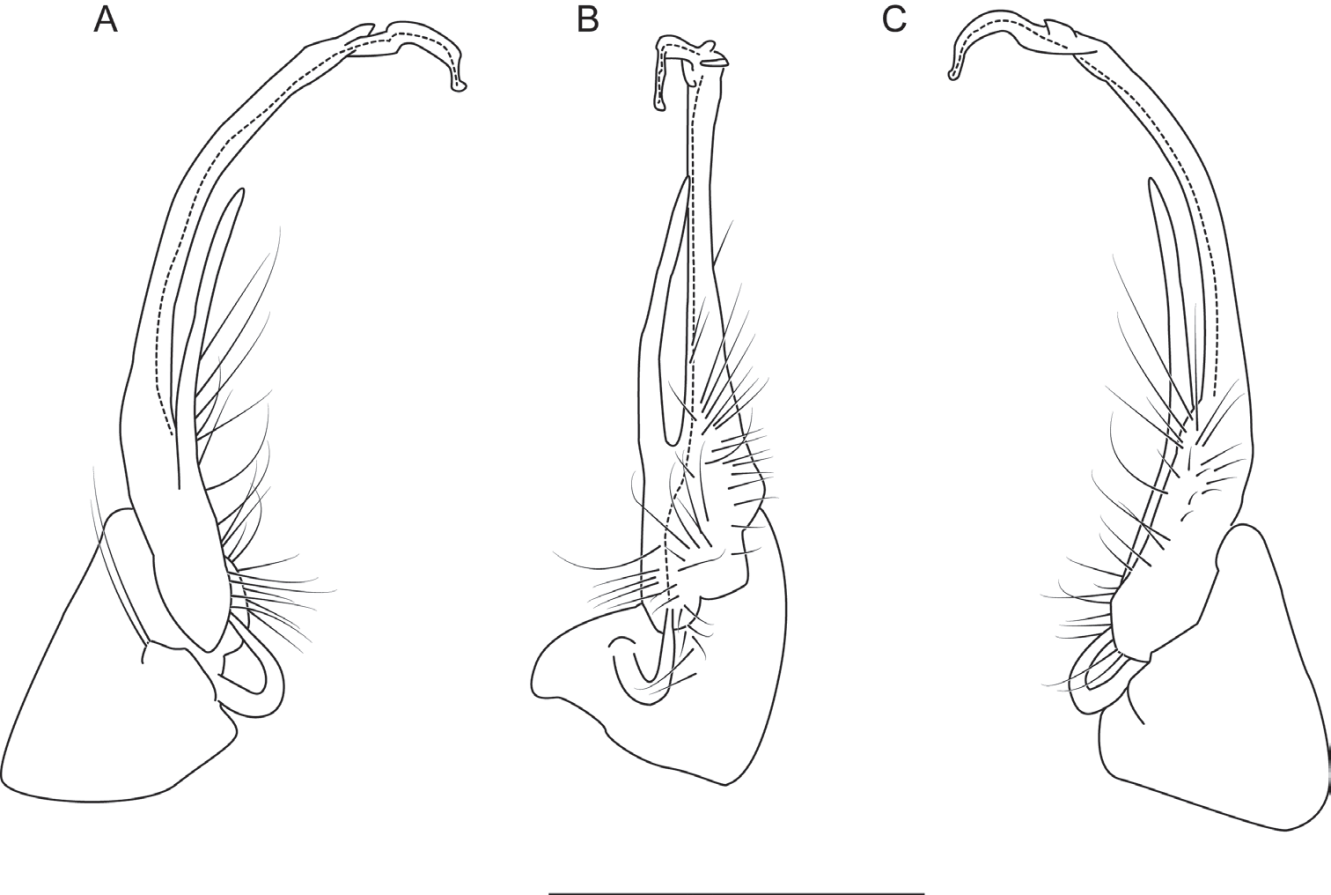
Left gonopod of *Nannariaaenigma* holotype (MPE02632, Smyth County, Virginia) **A** anterior view **B** medial view **C** posterior view. Scale bar: 1 mm.

Vernacular name: The New River Twisted-Claw Millipede Figs 7 , 8

Nannaria aenigma Means, Hennen & Marek in Means et al., 2021a: 16, S65, figs 3, 5S, T, 14.

##### Material examined.

**Type material: *Holotype***: United States – **Virginia** • ♂; Smyth County, Sugar Grove: Raccoon Branch Wilderness Campground, Jefferson National Forest, Raccoon Branch Trail; 36.7454°N, -81.4259°W, ±7 m; elev. 858 m; 5 May 2017; D. A. Hennen leg.; moist deciduous leaf litter of rhododendron and eastern hemlock; VTEC, MPE02632. ***Paratype***: United States – **Virginia** • 1 ♀; same collection data as for holotype; VTEC, MPE02633. **Non type material**: United States – **Virginia** • 1 ♂; Bland County, Burkes Garden, SW Garden Mtn., NE facing slope; 37.0783°N, -81.4152°W, ± 5000m; 30 June 1967; W. A. Shear leg.; VMNH, NAN0028 • 1 specimen; Bland County, west slope Walker Mtn along Va. Hy. 738; 37.1015°N, -80.8836°W, ± 3000m; 17 March 1989; R. L. Hoffman leg.; VMNH, NAN0421 • 1 specimen; Bland County, west side of Little Walker Mtn., 2 mi. S of Long Spur; 37.0638°N, -80.9481°W, ± 3000m; 31 March 1967; R. L. Hoffman, Gardner leg.; VMNH, NAN0425 • 1 ♂; Bland County, Rt 42 roadside picnic area for AT just S of Possum Creek; 36.9859°N, -81.4076°W, ± 12m; elev. 827 m; 13 October 2014; P. E. Marek leg.; VTEC, MPE00002 • 2 ♂♂; Bland County, VA-Rt 623 3km North Jct w/ Va 42 in Burke’s Garden Wilderness Area in Garden Mtn, 5.7 km WxNW of Walker Mtn; 37.0593°N, -81.2906°W, ± 7m; elev. 951 m; 14 October 2014; P. E. Marek, E. Francis, J. C. Means, T. McCoy leg.; VTEC, MPE00006, MPE00007 • 2 ♂♂; Bland County, 1 km N of jct with Rt. 42, on roadside, 6.6 km south of Burkes Garden; 37.0393°N, -81.3477°W, ± 3m; elev. 891 m; 14 May 2014; J. C. Means, P. E. Marek, E. Francis leg.; VTEC, MPE00018, MPE00019 • 3 ♂♂ and 1 ♀; Bland County, Ceres, off rt. 622, halfway up mountain, dry streambed; 36.9199°N, -81.4448°W, ± 4m; elev. 915 m; 10 June 2015; J. C. Means, P. Shorter leg.; VTEC, MPE00551, MPE00552, MPE00581, MPE00582 • 2 ♀♀; Bland County, side of rt. 717 pull off, in gorge up dry creek bed; 37.0093°N, -81.1674°W, ± 4m; elev. 762 m; 10 June 2015; J. C. Means, P. L. Shorter leg.; VTEC, MPE00583, MPE00584 • 1 ♂; Bland County, 3.15 air km W Hicksville, south slope of Rich Mountain; 37.1924°N, -81.1703°W; elev. 788 m; 21 March 2016; P. E. Marek, J. C. Means, A. Prewitt leg.; VTEC, MPE01008 • 1 ♂; Bland County, 1.4 air km SSW Carnot, north slope of Little Walker Mountain; 37.0306°N, -81.1035°W, ± 3m; elev. 802 m; 21 March 2016; P. E. Marek, J. C. Means, A. Prewitt leg.; VTEC, MPE01033 • 2 ♂♂; Bland County, 0.7 air km S Carnot, along CR 603 north slope of Little Walker Mountain; 37.0346°N, -81.0945°W, ± 2m; elev. 800 m; 21 March 2016; P. E. Marek, J. C. Means, A. Prewitt leg.; VTEC, MPE01040, MPE01041 • 2 ♂♂; Bland County, 1.2 air km SSW Carnot, nr intersection of Little Walker Creek and powerline; 37.0329°N, -81.1041°W, ± 3m; elev. 755 m; 21 March 2016; P. E. Marek, J. C. Means, A. Prewitt; VTEC, MPE01044, MPE01045 • 1 ♂; Bland County, 1.1 air km SSW Carnot, north of Little Walker Creek; 37.0352°N, -81.1055°W, ± 3m; elev. 804 m; 28 March 2016; P. E. Marek, J. C. Means, A. Prewitt leg.; VTEC, MPE01055 • 1 ♂; Bland County, 1.3 air km W Carnot, north of CR 717 on south slope Walker Mountain; 37.0391°N, -81.1114°W, ± 2m; elev. 871 m; 28 March 2016; P. E. Marek, J. C. Means, A. Prewitt leg.; VTEC, MPE01057 • 1 ♂; Bland County, 1.9 air km WNW Carnot, crest of Walker Mountain; 37.0466°N, -81.1159°W, ± 3m; elev. 1168 m; 30 March 2016; P. E. Marek, J. C. Means, A. Prewitt leg.; VTEC, MPE01059 • 3 ♂♂; Bland County, 2.0 air km WNW Carnot, crest of Walker Mountain; 37.0493°N, -81.1161°W, ± 2m; elev. 1118 m; 30 March 2016; P. E. Marek, J. C. Means, A. Prewitt leg.; VTEC, MPE01060, MPE01061, MPE01062 • 4 ♂♂; Bland County, 1.8 km WNW Carnot, next to rock face along Walker Mountain road; 37.0478°N, -81.1155°W, ± 4m; elev. 1184 m; 17 May 2016; P. Shorter, D. A. Hennen, D. Krall, A. Prewitt leg.; VTEC, MPE01197, MPE01199, MPE01200, MPE01201 • 1 ♂ and 3 ♀♀; Bland County, 1.8 km WNW Carnot, down steep hill along Walker Mountain road; 37.0478°N, -81.116°W, ± 5m; elev. 1178 m; 17 May 2016; P. L. Shorter, D. A. Hennen, A. Prewitt, D. Krall leg.; VTEC, MPE01205, MPE01203, MPE01204, MPE01206 • 1 ♂; Bland County, 2.4 km W Carnot, along Walker Mountain Road; 37.043°N, -81.1227°W, ± 5m; elev. 1131 m; 17 May 2016; P. Shorter, D. A. Hennen, D. Krall, A. Prewitt leg.; VTEC, MPE01216 • 1 ♂; Bland County, 3.2 km SW Hicksville, beside Grapefield Rd at forest access road; 37.1845°N, -81.1691°W; elev. 690 m; 23 May 2016; P. Shorter, V. Wong, D. Krall leg.; VTEC, MPE01419 • 2 ♂♂; Bland County, 1.2 km SSW Carnot, nr intersection of Little Walker Creek and powerline; 37.0329°N, -81.1043°W, ± 5m; elev. 813 m; 17 May 2016; D. A. Hennen, P. L. Shorter, D. Krall, A. Prewitt leg.; VTEC, MPE03701, MPE03702 • 1 ♂; Bland County, CR623, 2.8 rd km N Sharon Springs, S slope of Brushy Mtn; 37.0549°N, -81.2989°W; elev. 887 m; 26 May 2004; P. E. Marek leg.; VTEC, SPC000304 • 2 ♂♂; Bland County, Garden Mountain Quad, CR623, 2.8 rd km N Sharon Springs, S slope of Brushy Mountain; 37.0594°N, -81.2989°W; elev. 862 m; 14 May 2005; D. Beamer, M. Beamer leg.; VTEC, SPC000550, SPC000551 • 1 ♂; Grayson County, Lewis Fork, Fox Cr., Lewis Fork Trail; 36.682°N, -81.5159°W; 27 May 1984; Baumann, Nelson leg.; NCSM, NAN0478 • 1 specimen; Grayson County, Mount Rogers, beech-maple zone; 36.6597°N, -81.5447°W, ± 2000m; 19 May 1975; Douglas Ogle leg.; VMNH, NAN0420 • 1 specimen; Grayson County, W of Independence; 36.6296°N, -81.1715°W, ± 3000m; 18 June 1950; R. L. Hoffman leg.; VMNH, NAN0430 • 4 specimens; Pulaski County, Draper Mtn. above Pulaski; 37.0188°N, -80.7823°W, ± 3000m; 4 October 1959; R. L. Hoffman, R. Crabill leg.; VMNH, NAN0419 • 1 ♂ and 1♀; Pulaski County, off of 738, north side of Brush Mtn. range (Croy Mtn. or Little Walker); 37.1018°N, -80.8867°W; elev. 678 m; 10 May 2015; J. C. Means leg.; VTEC, MPE00350, MPE00392 • 2 ♂♂ and 1 ♀; Pulaski County, gully off rt 641 gravel rd.; 37.073°N, -80.873°W; elev. 671 m; 13 May 2015; J. C. Means leg.; VTEC, MPE00409, MPE00416, MPE00412 • 1 ♀; Pulaski County, north side of Draper Mtn.; 37.0252°N, -80.7752°W, ± 2m; elev. 740 m; 28 July 2015; P. E. Marek, J. C. Means, V. Wong, P. Shorter leg.; VTEC, MPE00759 • 1 ♀; Pulaski County, in gully next to private drive off Case Knife Road; 37.0332°N, -80.8087°W, ± 3m; elev. 639 m; 28 July 2015; P. E. Marek, P. Shorter, J. C. Means, V. Wong leg.; VTEC, MPE00762 • 2 ♂♂ and 2 ♀♀; Pulaski County, VA-650; 37.0248°N, -80.7842°W, ± 7m; elev. 667 m; 1 October 2015; V. Wong, P. E. Marek leg.; VTEC, MPE00857, MPE00858, MPE00856, MPE00859 • 1 specimen; Russell County, SE side of Beartown Mountain; 36.9244°N, -81.8786°W, ± 2000m; 23 April 2003; A. C. Chazal, C. S. Hobson, et al. leg.; VMNH, NAN0412 • 1 specimen; Russell County, Clinch Mtn., 1 mile SE of Repass; 36.9759°N, -81.7932°W, ± 1000m; elev. 1128 m; 5 July 1962; R. L. Hoffman leg.; VMNH, NAN0418 • 1 ♀; Smyth County, Sugar Grove: Raccoon Branch Wilderness campground, near beginning of Raccoon Branch Trail by campsite 8; 36.7462°N, -81.4247°W, ± 6m; elev. 855 m; 5 May 2017; D. A. Hennen leg.; VTEC, MPE02635 • 2 specimens; Smyth County, Grindstone Camp Area, 4.5 mi. W of Troutdale; 36.6875°N, -81.5423°W, ± 500m; 24 May 1975; R. L. Hoffman leg.; VMNH, NAN0411 • 1 specimen; Smyth County, Big Branch north slope of Whitetop Mtn; 36.658°N, -81.599°W, ± 1000m; 3 May 1992; K. A. Buhlmann leg.; VMNH, NAN0414 • 1 specimen; Smyth County, Va. Hy. 600, halfway between Konnarock and Elk Garden; 36.6582°N, -81.5884°W, ± 2000m; elev. 1219 m; 10 May 1982; R. L. Hoffman, et al. leg.; VMNH, NAN0417 • 1 specimen; Smyth County, Big Walker Mtn. W of Hungry Mother State Park; 36.8927°N, -81.559°W, ± 3000m; 9 September 1956; R. L. Hoffman leg.; VMNH, NAN0423 • 3 specimens; Smyth County, NW slope of Iron Mtn., 7 mi. SE of Chilhowie; 36.7012°N, -81.6133°W, ± 3000m; 4 May 1964; R. L. Hoffman leg.; VMNH, NAN0424 • 1 ♂; Smyth County, Mt Rogers Nat’l Rec Area, FR 84; 36.7067°N, -81.6028°W, ± 2m; elev. 1138 m; 25 June 2014; J. C. Means, P. E. Marek, E. Francis leg.; VTEC, MPE00072 • 1 ♂ and 7 ♀♀; Smyth County, Side of road (VA-16) on west slope of Big Walker Mtn, ca. 3 mi W of Hungry Mother State Park; 36.9112°N, -81.5317°W, ± 6m; elev. 1054 m; 9 September 2014; J. C. Means leg.; VTEC, MPE00172, MPE00168, MPE00170, MPE00171, MPE00173, MPE00175, MPE00176, MPE00177 • 2 ♂♂; Smyth County, south of Laurel Bed Lake, off Tumbling Creek Rd.; 36.9384°N, -81.8118°W, ± 8m; elev. 956 m; 13 June 2016; J. C. Means, D. A. Hennen leg.; VTEC, MPE01680, MPE01683 • 1 specimen; Tazewell County, River Mtn. summit, NW, above Bluefield; 37.223°N, -81.2479°W, ± 5000m; 17 April 1973; C. J. Chapman leg.; VMNH, NAN0030 • 7 specimens; Tazewell County, Burkes Garden, crest of Garden Mtn at Rt. 623; 37.1222°N, -81.3647°W, ± 3000m; elev. 1158 m; 14 April 1965; Radford College herpetology class leg.; VMNH, NAN0318 • 4 specimens; Tazewell County, East River Mtn.; 37.2125°N, -81.2979°W, ± 3000m; 11 September 1955; R. L. Hoffman leg.; VMNH, NAN0416 • 2 ♂♂ and 1 ♀; Tazewell County, Reese Bowen’s property, down by Indian Creek; 37.0148°N, -81.41°W, ± 2m; elev. 735 m; 28 October 2014; J. C. Means, R. Bowen leg.; VTEC, MPE00256, MPE00268, MPE00267 • 1 specimen; Washington County, 2 mi SE of Taylors Valley; 36.6146°N, -81.6827°W, ± 3000m; 7 May 1972; R. L. Hoffman, L. S. Knight leg.; VMNH, NAN0426 • 5 specimens; Washington County, 4 miles SW of Konnarock; 36.6306°N, -81.6647°W, ± 3000m; 28 April 1951; L. Hubricht leg.; VMNH, NAN0429 • 1 ♂; Washington County, south of Laurel Bed Lake, on Laurel Bed Road, pull off on road; 36.9477°N, -81.8242°W, ± 9m; elev. 1047 m; 13 June 2016; J. C. Means, D. A. Hennen leg.; VTEC, MPE01648 • 1 ♀; Washington County, south of Laurel Bed Lake, off Tumbling Creek Rd.; 36.9384°N, -81.8118°W, ± 8m; elev. 956 m; 13 June 2016; J. C. Means, D. A. Hennen leg.; VTEC, MPE01681 • 1 ♂; Washington County, Hayter’s Gap, pull off along Brumley Gap Rd (CR 689) before Dublin Ln.; 36.8304°N, -81.9593°W, ± 9m; elev. 587 m; 8 June 2016; J. C. Means, D. A. Hennen leg.; VTEC, MPE02106 • 2 specimens; Wythe County, Gullion Fork Wildlife Management Area, w. of Blackfork; 36.9816°N, -81.2829°W, ± 3000m; 1 September 1964; D. E. Marvin, et al. leg.; VMNH, NAN0427 • 1 specimen; Wythe County, 3 mi. S of Speedwell on U.S. Hy. 52, W side of Iron Mtn.; 36.7702°N, -81.1717°W, ± 5000m; 13 July 1962; R. L. Hoffman leg.; VMNH, NAN0428 • 1 ♂; Wythe County, Seven Sisters Campground; 37.0195°N, -81.1355°W, ± 3m; elev. 788 m; 10 June 2015; J. C. Means, P. L. Shorter leg.; VTEC, MPE00576 • 4 ♀♀; Wythe County, off Oriole Dr.; 37.016°N, -81.2436°W, ± 4m; elev. 804 m; 16 June 2015; J. C. Means, P. L. Shorter leg.; VTEC, MPE00649, MPE00650, MPE00652, MPE00653 • 1 ♂; Wythe County, off rt. 686 side of road near Deer Trail Campground, marshland; 37.0223°N, -81.2048°W, ± 3m; elev. 729 m; 16 June 2015; J. C. Means, P. L. Shorter leg.; VTEC, MPE00658 • 2 ♂♂; Wythe County, Crawfish Valley, channel Rock Hollow trail 1 mile from Strawberry Rd. end; 36.9526°N, -81.3247°W; 24 March 2017; C. W. Harden leg.; VTEC, MPE02415, MPE02423 • 5 ♂♂; Wythe County, Crawfish Valley, Channel Rock, follow trail about 1.5 miles from Strawberry Rd. end; 36.9585°N, -81.3189°W; 24 March 2017; C. W. Harden leg.; VTEC, MPE02417, MPE02418, MPE02425, MPE02426, MPE02427 • 2 ♂♂ and 3 ♀♀; Wythe County, side of Oriole Rd. in moist creek bed; 37.0157°N, -81.246°W, ± 5m; elev. 780 m; 11 June 2015; J. C. Means, P. L. Shorter leg.; VTEC, MPE03709, MPE03723, MPE03724, MPE03725, MPE03726. Complete material examined information listed in Suppl. material 1.

##### Diagnosis.

Adults of *Nannariaaenigma* can be separated from the geographically close and morphologically similar species *N.acroteria* sp. nov. and *N.wilsoni* by the following characters. Acropodite medial flange with a dorsal median projection, rather than lacking a projection as in *N.wilsoni*. Prefemoral process acicular or slightly curving, length 3/4 or less the length of the acropodite, rather than having a sinuous and strongly curving prefemoral process with length greater than 3/4 the length of the acropodite, as in *N.acroteria* sp. nov. and *N.wilsoni*.

##### Description.

Suppl. material 2. Based on holotype (♂) MPE02632 and paratype (♀) MPE02633.

**Measurements**: Taken from holotype (♂) MPE02632: BL = 31.95, CW = 4.20, IW = 2.79, ISW = 0.88, B10W = 5.19, B10H = 3.01. **Color.** Tergites with two paranotal white spots, collum outlined in white, and tergites with background black (Fig. 7). **Gonopods.** Male gonopod acropodite arc gradually curving (Fig. 8A). Acropodite with smoothly-undulating helical twist at anterior bend (Fig. 8C). Acropodite medial flange laminate, with a dorsal triangular tooth (Fig. 8C) and acropodite tip lacking medial and lateral flanges. Acropodite tip entire, slightly constricted distally (Fig. 8A–C); directed caudally (Fig. 8B). Prefemoral process acicular, straight, with only a slight curve (Fig. 8A–C). Prefemoral process tip directed cephalically (Fig. 8A). Prefemoral process length about half that of the acropodite. **Cyphopods.** Female cyphopod receptacle triangular.

##### Variation.

The dorsal triangular tooth process on the gonopod acropodite varies in size and shape, but is generally triangular. The acropodite tip is sometimes more elaborately lobed before the distal constriction in some specimens. The prefemoral process varies in its length, sometimes being almost as long as the acropodite. *Nannariaaenigma* varies in color as well, with some specimens having pink to red paranotal spots instead of white.

##### Distribution.

*Nannariaaenigma* is distributed throughout southwest Virginia, and is known from the following counties: Bland, Grayson, Pulaski, Russell, Smyth, Tazewell, Washington, and Wythe (Fig. 51). In the eastern portion of its distribution, it is limited by the course of the New River. It may eventually be found in West Virginia, Tennessee, and North Carolina, as some of its locality records are very near the borders of these states.

##### Ecological notes.

This species has been found in mesic deciduous forest habitats, as well as ericaceous hemlock-rhododendron coves, and at elevations ranging from 587 m to 1219 m.

##### Etymology.

The specific name is a noun in apposition, derived from the Latin *aenigma*, for something obscure or a riddle. This is in reference to the question-mark like shape of the acropodite (Means et al. 2021a).

#### ﻿ Nannaria amicalola sp. nov.

Taxon classificationAnimaliaPolydesmidaXystodesmidae

9B587F6C-E86D-5E28-A42E-2E7870304F1D

http://zoobank.org/7220D2E8-34A9-4105-B7E6-49386E99AAEC

**Figure 9.**
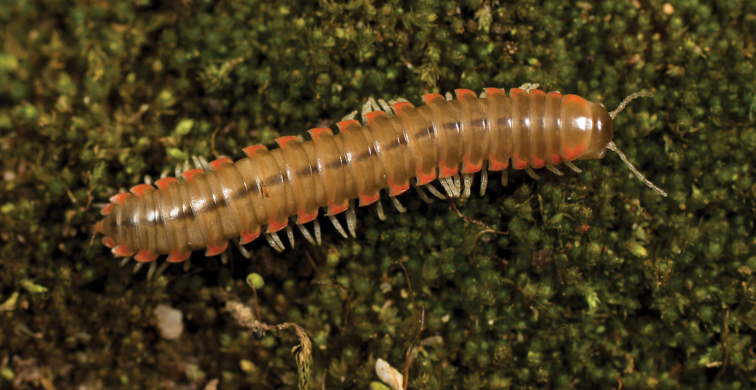
*Nannariaamicalola* sp. nov., in situ, female paratype (MPE01230) from Lumpkin County, Georgia.

**Figure 10.**
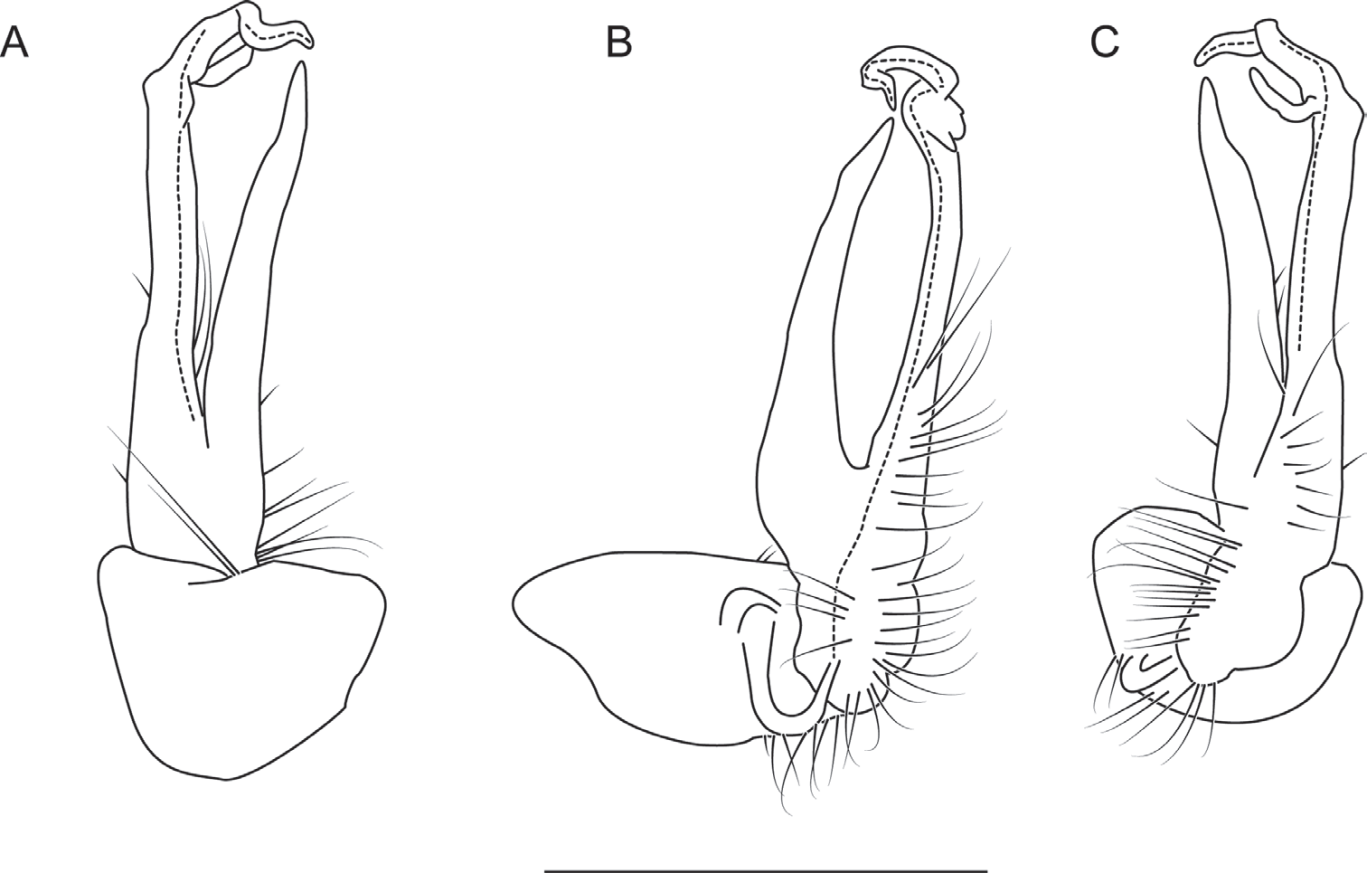
Left gonopod of *Nannariaamicalola* sp. nov. holotype male (NAN0319, Dawson County, GA) **A** anterior view **B** medial view **C** posterior view. Scale bar: 1 mm.

Vernacular name: The Tumbling Waters Twisted-Claw Millipede Figs 9 , 10

Nannaria sp. nov. ‘Amicolola’: Means et al. 2021b: 85.

##### Material examined.

**Type material: *Holotype***: United States – **Georgia** • ♂; Dawson County, 6 miles W of Amicalola Falls; 34.5681°N, -84.3140°W, ±5000m; 6 November 1960; L. Hubricht leg.; ex ravine; VMNH, NAN0319. ***Paratypes***: United States – **Georgia** • 1 ♀; same collection data as for holotype; VMNH, NAN0671 • 2 ♀♀; Lumpkin County, 3.5 km NW Nimblewill on Nimblewill Gap Rd/FS 28-2, before road crosses Chester Creek, on short path to Nimblewill Creek; 34.5753°N, -84.1757°W; elev. 590 m; 24 May 2016; J. C. Means, D. A. Hennen leg.; ex rhododendron, maple, oak, hemlock gully with lots of poison ivy, moist litter; VTEC, MPE01230, MPE01280. **Non type material**: United States – **Georgia** • 1 ♀; Dawson County, Amicalola Falls State Park; 34.5611°N, -84.2474°W; 16 April 1978; R. M. Shelley, R. T. Ashton leg.; NCSM, NAN0473. Complete material examined information listed in Suppl. material 1.

##### Diagnosis.

Adults of *Nannariaamicalola* sp. nov. can be separated from the geographically close and morphologically similar species *N.antarctica* sp. nov. and *N.rhododendra* sp. nov. by the following characters. Acropodite anterior bend acutely bent, rather than slightly twisted as in *N.rhododendra* sp. nov. Acropodite medial flange long and hooked, curving cephalically rather than lobed and sinuous as in *N.rhododendra* sp. nov. Acropodite tip entire, not bifurcate as in *N.antarctica* sp. nov.

##### Description.

Suppl. material 2. Based on holotype (♂) NAN0319 and paratype (♀) MPE01230.

**Measurements**: Taken from holotype (♂) NAN0319: BL = 24.40, CW = 3.56, IW = 2.82, ISW = 0.96, B10W = 4.70, B10H = 3.00. **Color.** Tergites with two paranotal orange spots, collum outlined in orange, and tergites with background chestnut brown (Fig. 9). **Gonopods.** Acropodite arc straight, with abrupt bend distally (Fig. 10A–C). Acropodite with acute twist at anterior bend (Fig. 10A), acropodite medial flange tooth-like, long, and hooked, curving cephalically (Fig. 10C). Acropodite tip medial and lateral flanges absent. Acropodite tip entire, curving and directed ventrally (Fig. 10B). Prefemoral process laminate, very wide at base (Fig. 10B); straight, tapering to tip with a slight curve, with tip directed cephalically (Fig. 10A). **Cyphopods.** Female cyphopod receptacle enlarged into a triangular hood, covering cyphopod valves.

##### Variation.

No noticeable variation was observed.

##### Distribution.

*Nannariaamicalola* sp. nov. is only known from Dawson and Lumpkin counties in northern Georgia (Fig. 52).

##### Ecological notes.

This species inhabits ravines and riparian areas with a mix of moist oak, maple, hemlock, and rhododendron forest.

##### Etymology.

This species is named for nearby Amicalola Falls State Park, which itself is named after the Cherokee expression *amo* and *kalola* for “tumbling waters.” The specific name is a noun in apposition.

#### ﻿ Nannaria antarctica sp. nov.

Taxon classificationAnimaliaPolydesmidaXystodesmidae

CD5E8599-CB55-5220-BACE-3B348433EFC1

http://zoobank.org/A076437D-7513-42E4-84FD-EF177B248FCF

**Figure 11.**
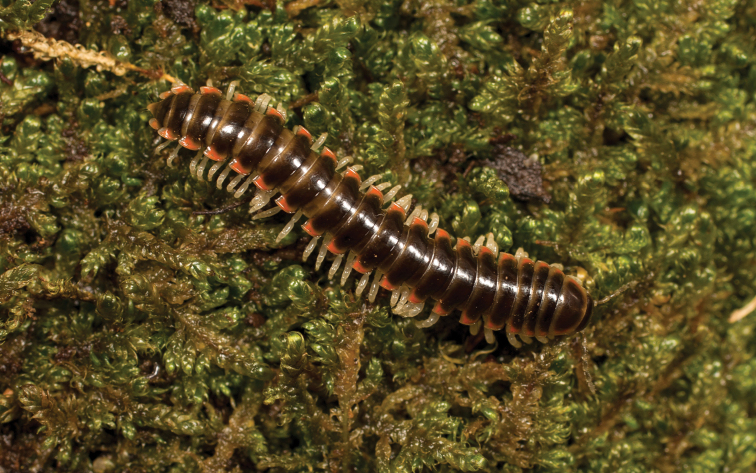
*Nannariaantarctica* sp. nov., in situ, male paratype (MPE03317) from Towns County, Georgia.

**Figure 12.**
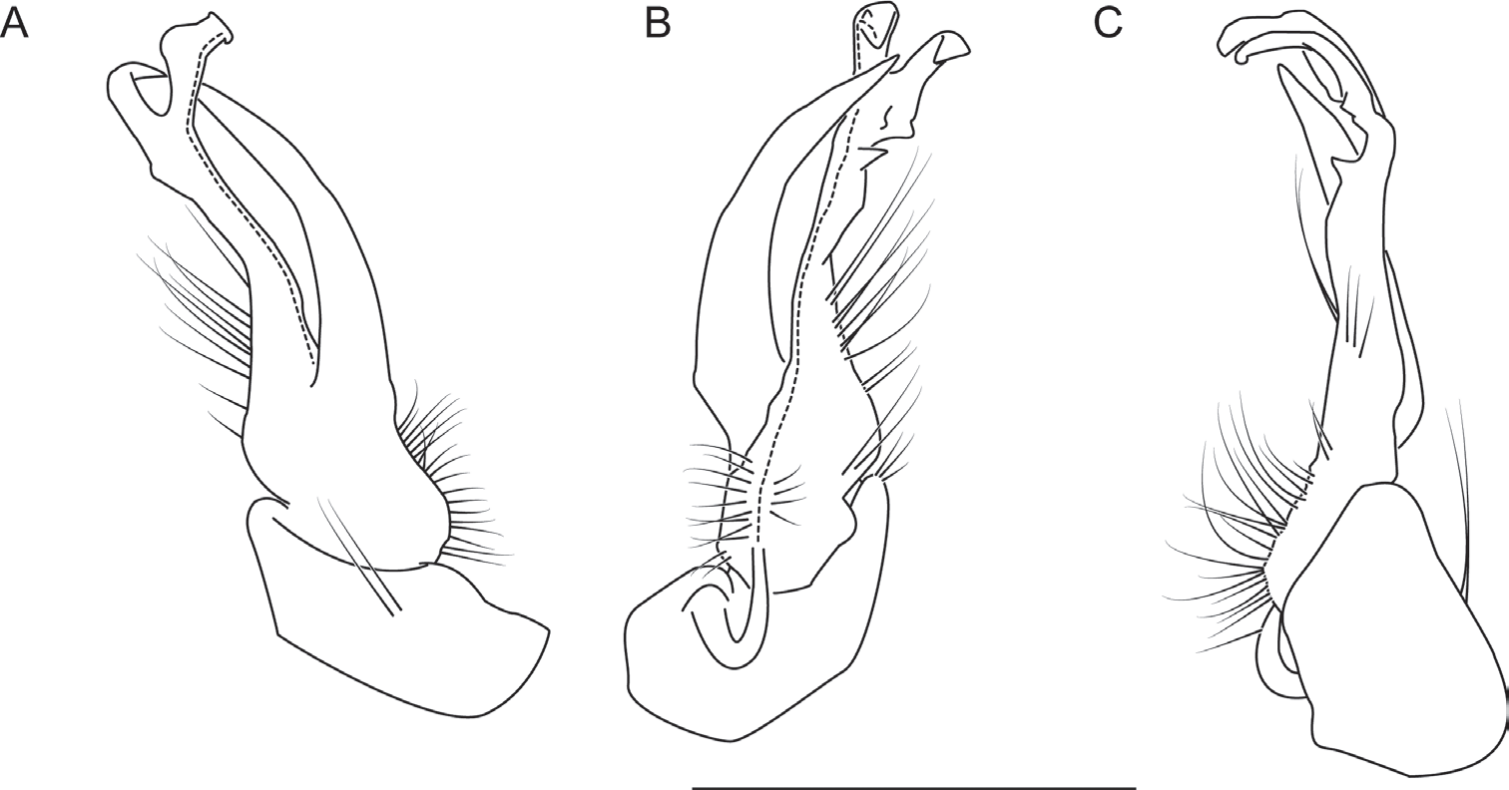
Left gonopod of *Nannariaantarctica* sp. nov. male holotype (MPE03311, Towns County, Georgia) **A** anterior view **B** medial view **C** posterior view. Scale bar: 1 mm.

Vernacular name: The Chilled Twisted-Claw Millipede Figs 11 , 12

##### Material examined.

**Type material: *Holotype***: United States – **North Carolina** • ♂; Macon County, 9.79 mi WSW of Franklin, at scenic view spot along US 64 beside Poplar Cove Creek; 35.1241°N, -83.5392°W, ±10m; elev. 1082 m; 26 October 2017; D. A. Hennen, J. C. Means leg.; steep hillside with moist dark crumbly soil in tuliptree, buckeye, birch, cherry woods beside mountain steam; VTEC, MPE03311. ***Paratypes***: United States – **Georgia** • 3 ♂♂ and 3 ♀♀; Towns County, 9.5 air mi. (15.3 km) NNW of Helen: Enota Mountain Retreat, off Georgia State Route 180, trail by primitive campground, above Henson Creek; 34.8364°N, -83.7710°W, ±13m; elev. 787 m; 25 October 2017; D. A. Hennen, J. C. Means leg.; moist deciduous woods with crumbly, dark soil, rhododendron, hemlock, tuliptree forest; VTEC, MPE03317; VMNH, MPE03380; FMNH, MPE03382; VTEC, MPE03378; VMNH, MPE03379; FMNH, MPE03381. **Non type material**: United States – **Georgia** • 1 ♂; Dawson County, 6 miles W of Amicalola Falls; 34.5681°N, -84.314°W, ± 4000m; 6 November 1960; L. Hubricht leg.; VMNH, NAN0672 • 8 ♂♂; Towns County, 17 miles NW Clayton, Burnt Cabin Br.; 34.989°N, -83.5586°W; 30 September 1994; G. Wharton leg.; NCSM, NAN0187, NAN0188, NAN0481 • 6 ♂♂ and 2 ♀♀; Towns County, 9.5 air miles (15.3 km) NNW of Helen: Enota Mountain Retreat, off Georgia State Route 180, trail by primitive campground, above Henson Creek; 34.8364°N, -83.771°W, ± 13m; elev. 787 m; 25 October 2017; D. A. Hennen, J. C. Means leg.; VTEC, MPE03377, MPE03384, MPE03385, MPE03386, MPE03388, MPE03389, MPE03383, MPE03387 • 1 ♀; Union County, Blairsville: Vogel State Park, Trahlyta Falls Trail; 34.7701°N, -83.9163°W, ± 12m; elev. 697 m; 14 October 2018; D. A. Hennen leg.; VTEC, MPE04608; **North Carolina** • 3 ♂♂ and 3 ♀♀; Macon County, trail from deep gap to top Standing Ind. Mtn.; 35.0355°N, -83.538°W; 16 August 1977; A. L. Braswell, M. Baranksi leg.; NCSM, NAN0524 • 2 ♂♂; Macon County, Coweeta Hydrologic Station; 35.0598°N, -83.4302°W; 16 June 1978; Lee Reynolds leg.; NCSM, NAN0555 • 7 ♂♂ and 3 ♀♀; same collection data as for preceding; 29 August 1977; NCSM, NAN0557 • 9 ♂♂ and 2 ♀♀; same collection data as for preceding; 20 October 1977; NCSM, NAN0559, NAN0569, NAN0577, NAN0594 • 5 ♂♂ and 7 ♀♀; same collection data as for preceding; 14 September 1977; NCSM, NAN0561, NAN0562 • 2 ♂♂ and 1 ♀; same collection data as for preceding; 7 April 1977; NCSM, NAN0563 • 8 ♂♂ and 7 ♀♀; same collection data as for preceding; 29 September 1977; NCSM, NAN0565, NAN0574, NAN0589 • 6 ♂♂ and 8 ♀♀; same collection data as for preceding; 27 October 1977; NCSM, NAN0566, NAN0580, NAN0593, NAN0634 • 10 ♂♂ and 9 ♀♀; same collection data as for preceding; 22 September 1977; NCSM, NAN0568, NAN0570, NAN0571 • 10 ♂♂ and 7 ♀♀; same collection data as for preceding; 13 October 1977; NCSM, NAN0573, NAN0588, NAN0596, NAN0598 • 2 ♂♂ and 1 ♀; same collection data as for preceding; 2 June 1978; NCSM, NAN0575, NAN0638 • 6 ♂♂ and 3 ♀♀; same collection data as for preceding; 21 April 1978; NCSM, NAN0576, NAN0625 • 5 ♂♂; same collection data as for preceding; 7 April 1978; NCSM, NAN0578, NAN0613 • 70 ♂♂ and 76 ♀♀; same collection data as for preceding; 3 November 1977; NCSM, NAN0581, NAN0582, NAN0584, NAN0600, NAN0628, NAN0629 • 88 ♂♂ and 81 ♀♀; same collection data as for preceding; 10 November 1977; NCSM, NAN0585, NAN0590, NAN0626, NAN0627, NAN0640 • 6 ♂♂ and 3 ♀♀; same collection data as for preceding; 17 November 1977; NCSM, NAN0592, NAN0642 • 4 ♂♂; same collection data as for preceding; 6 October 1977; NCSM, NAN0597, NAN0606 • 9 ♂♂ and 3 ♀♀; same collection data as for preceding; 26 May 1978; NCSM, NAN0599, NAN0607, NAN0614 • 12 ♂♂ and 5 ♀♀; same collection data as for preceding; 28 April 1978; NCSM, NAN0608, NAN0615, NAN0620, NAN0639 • 2 ♂♂ and 1 ♀; same collection data as for preceding; 14 April 1977; NCSM, NAN0609 • 14 ♂♂ and 5 ♀♀; same collection data as for preceding; 31 March 1978; NCSM, NAN0610, NAN0624, NAN0630, NAN0632, NAN0635, NAN0637 • 4 ♂♂ and 3 ♀♀; same collection data as for preceding; 9 June 1978; NCSM, NAN0611 • 20 ♂♂ and 6 ♀♀; same collection data as for preceding; 5 May 1978; NCSM, NAN0612, NAN0616, NAN0617, NAN0619, NAN0636 • 4 ♂♂ and 5 ♀♀; same collection data as for preceding; 6 September 1978; NCSM, NAN0618 • 6 ♂♂ and 3 ♀♀; same collection data as for preceding; 14 April 1978; NCSM, NAN0622, NAN0631 • 1 ♂; Macon County, Coweeta Hydro. Lab. Coldspring Gap; 35.0214°N, -83.4482°W, ± 1000m; elev. 1189 m; 17 April to 28 May 1994; J. Laerm, et al. leg.; VMNH, NAN0312 • 7 ♂♂ and 3 ♀♀; Macon County, Nr. Wine Spring Cr., burn; 35.1921°N, -83.6394°W, ± 5000m; 13 August 1996; J. Laerm, et al. leg.; VMNH, NAN0313 • 19 specimens; Macon County, Nr. Wine Spring Cr., control-C3; 35.1921°N, -83.6394°W, ± 5000m; 16 June 1995; J. Laerm, et al. leg.; VMNH, NAN0315 • 13 specimens; Macon County, Coweeta Hydrologic Laboratory, nr Norton; 35.0598°N, -83.4302°W, ± 2000m; elev. 991 m; 17 September 1964; M. R. Steeves leg.; VMNH, NAN0362 • 1 ♂; Macon County, Nantahala Mtns, Coweeta Hydrologic Lab, Watershed 14; 35.0546°N, -83.432°W; elev. 690 m; 25 September 2004; ECU TABC leg.; VTEC, SPC000367. Complete material examined information listed in Suppl. material 1.

##### Diagnosis.

Adults of *Nannariaantarctica* sp. nov. can be separated from the geographically close and morphologically similar species *N.austricola* and *N.rhododendra* sp. nov. by the following characters. Acropodite with a strong twist at anterior bend, rather than a slightly-twisted helix as in *N.rhododendra* sp. nov. Acropodite bifurcate, rather than entire as in *N.austricola*.

##### Description.

Suppl. material 2. Based on holotype (♂) MPE03311 and paratype (♀) MPE03378.

**Measurements**: Taken from holotype (♂) MPE03311: BL = 23.50, CW = 3.20, IW = 2.48, ISW = 0.78, B10W = 3.92, B10H = 2.32. **Color.** Tergites with two paranotal orange spots, collum outlined in orange, and tergites with background chestnut brown (Fig. 11). **Gonopods.** Male gonopod acropodite arc straight, with abrupt bend distally (Fig. 12A–C). Acropodite with acute twist at anterior bend, appearing somewhat scooped and folded (Fig. 12C). Acropodite medial flange tooth-like, ending in a sharp point (Fig. 12C). Acropodite tip medial flange laminate, long and curving, terminating in a distal sharp point (Fig. 12A, B). Acropodite tip lateral flange lobed, and with a short tooth-like projection dorsally (Fig. 12A). Tip of acropodite bifurcate, each branch hooked at tip and directed medially (Fig. 12B). Prefemoral process sinuously tapered and quite thick at base (Fig. 12B), curving laterally (Fig. 12A). Prefemoral process crossing acropodite ventrolaterally (Fig. 12A), and with tip directed medially. **Cyphopods.** Female cyphopod receptacle in the shape of a finger-like projection, curving over cyphopod valves and tapering distally.

##### Variation.

No noticeable variation observed.

##### Distribution.

*Nannariaantarctica* sp. nov. is known from Dawson and Towns counties, Georgia, and Macon County, North Carolina (Fig. 52).

##### Ecological notes.

This species has been collected in typical *wilsoni* species group habitat: mesic deciduous forest (oak, maple, tuliptree, birch, cherry, buckeye) and rhododendron and hemlock coves. It ranges in elevation from 690 m to 1189 m.

##### Etymology.

This species is named for the extreme cold temperatures experienced by DAH and JCM while collecting this species. The specific name is a feminine adjective in the nominative case, derived from the Greek *antarktikos*, meaning southern, and refers to the currently frozen continent at Earth’s South Pole.

#### ﻿ Nannaria austricola

Taxon classificationAnimaliaPolydesmidaXystodesmidae

Hoffman, 1950

256D0399-91CC-54DD-A84C-A1D4F0FDD5EE

**Figure 13.**
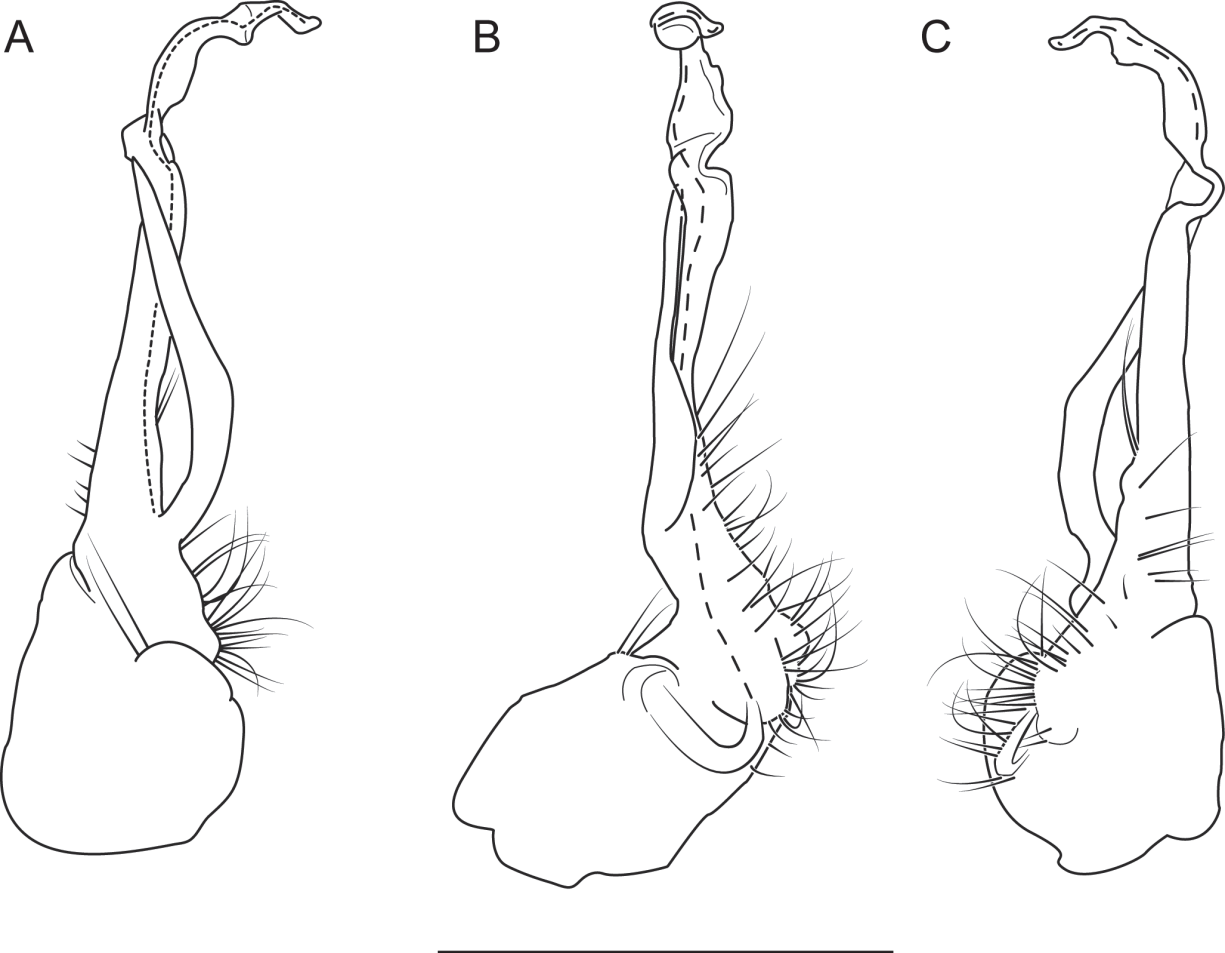
Left gonopod of *Nannariaaustricola* (NAN0072, Macon County, North Carolina) **A** anterior view **B** medial view **C** posterior view. Scale bar: 1 mm.

Vernacular name: The Highlands Twisted-Claw Millipede Fig. 13

Nannaria austricola Hoffman, 1950: 26, pl. 8, figs 26, 27. Chamberlin and Hoffman 1958: 40. Wray 1967: 152. Hoffman 1999: 365 (350 in pdf version). Shelley 2000: 196. Marek and Bond 2006: 721. Means et al. 2021a: S65. Means et al 2021b: 9, fig. 4D.

##### Type material.

***Holotype***: United States – **North Carolina** • ♀; Macon County, Highlands, Satulah Mountain; 35.036°N, -83.191°W, ±2000m; 26 July 1949; R. L. Hoffman leg.; USNM Entomology, no. 1879; (non vidi). Habitat at the type locality was a “dense, wet rhododendron thicket edging a small swift stream” (Hoffman 1950).

##### Material examined.

**Non type material**: United States – **Georgia** • 1 ♀; Rabun County, Glade Mtn.; 34.9966°N, -83.1341°W, ± 5000m; 27 July 1949; R. L. Hoffman leg.; VMNH, NAN0075; **North Carolina** • 3 ♂♂ and 1 ♀; Macon County, 5 miles NW of Highlands; 35.1038°N, -83.2594°W, ±5000m; 9 July 1958; R. L. Hoffman leg.; VMNH, NAN0072 • 1 ♂; Macon County, 2.25 mi NW Highlands, along US 64 at Bridal Veil Falls; 35.072°N, -83.2293°W; 4 April 1980; A. L. Braswell leg.; NCSM, NAN0474 • 2 ♀♀; Macon County, Highlands; 35.0525°N, -83.1969°W, ± 4322m; 1 to 2 June 1954; VMNH, NAN0071 • 2 specimens; Macon County, Highlands, Bowery Road; 35.0571°N, -83.1705°W, ± 3000m; 16 July 1962; A. Van Pelt leg.; VMNH, NAN0076 • 2 ♂♂; Macon County, Coweeta Hydrologic Station; 35.0597°N, -83.4305°W; 7 October 1977; Lee Reynolds leg.; NCSM, NAN0503, NAN0587 • 4 ♂♂; Macon County, Coweeta Hydrologic Station; 35.0598°N, -83.4302°W; 13 October 1978; Lee Reynolds leg.; NCSM, NAN0572 • 2 ♂♂; same collection data as for preceding; 20 October 1977; NCSM, NAN0583 • 1 ♂; same collection data as for preceding; 6 October 1977; NCSM, NAN0586 • 2 ♂♂; same collection data as for preceding; 3 November 1977; NCSM, NAN0595 • 4 ♂♂; same collection data as for preceding; 13 October 1977; NCSM, NAN0602 • 1 ♂ and 1 ♀; same collection data as for preceding; 26 May 1978; NCSM, NAN0623 • 1 ♂; same collection data as for preceding; 28 April 1978; NCSM, NAN0633 • 5 ♂♂ and 7 ♀♀; Macon County, Coweeta Hydro. Lab. Ball Creek Cove; 35.0595°N, -83.4288°W, ± 2000m; elev. 1311 m; 17 April to 28 May 1994; J. Laerm, et al. leg.; VMNH, NAN0291 • 1 ♂; Macon County, Coweeta Hydro. Lab. Drymans Fork Cove; 35.0433°N, -83.4275°W, ± 3000m; elev. 1311 m; 17 April to 23 July 1994; J. Laerm, et al. leg.; VMNH, NAN0311 • 4 ♂♂; Macon County, Coweeta Hydro. Lab. Coldspring Gap; 35.0214°N, -83.4482°W, ± 1000m; elev. 1189 m; 17 April to 28 May 1994; J. Laerm, et al. leg.; VMNH, NAN0312 • 1 ♂; Macon County, Nr. Wine Spring Cr., control-C3; 5.1921°N, -83.6394°W, ± 5000m; 16 June 1995; J. Laerm, et al. leg.; VMNH, NAN0314 • 1 ♂ and 1 ♀; Macon County, Nantahala Mtns, Coweeta Hydrologic Lab, Watershed 2; 35.0633°N, -83.4368°W; elev. 690 m; 24 September 2004; ECU TABC leg.; VTEC, SPC000352, SPC000353. Complete material examined information listed in Suppl. material 1.

##### Diagnosis.

Adults of *Nannariaaustricola* can be separated from the geographically close and morphologically similar species *N.antarctica* sp. nov., *N.nessa* sp. nov., and *N.scutellaria* by the following characters. Acropodite entire, not bifurcate as in *N.antarctica* sp. nov. Acropodite medial flange lobed, not acuminate and tapering as in *N.scutellaria*. Acropodite tip medial flange lobed, rather than absent as in *N.nessa* sp. nov. Prefemoral process without a notch on medial side, which is present in *N.nessa* sp. nov.

##### Description.

Suppl. material 2. Based on (♂) and (♀) specimens from lot NAN0072.

**Measurements**: Taken from (♂) specimen NAN0072: BL = 22.70, CW = 3.10, IW = 2.48, ISW = 0.75, B10W = 4.10, B10H = 2.40. **Color.** Tergites with two paranotal pink spots, collum outlined in pink, and tergites with background olive-brown. **Gonopods.** Male gonopod acropodite arc straight, with an abrupt bend distally (Fig. 13A–C). Acropodite with acute twist at anterior bend, appearing crimped and folded (Fig. 13C). Acropodite medial flange lobed (Fig. 13C), acropodite tip lateral flange absent. Acropodite tip entire, directed medially (Fig. 13B). Prefemoral process sinuous, thicker at base and tapering distally, curving laterally and crossing acropodite dorsolaterally (Fig. 13A). Prefemoral process tip directed cephalically (Fig. 13A). **Cyphopods.** Female cyphopod receptacle triangular.

##### Variation.

Acropodite medial flange size varies from narrow to wide.

##### Distribution.

*Nannariaaustricola* is only known from Macon County, North Carolina and adjacent Rabun County, Georgia (Fig. 52).

##### Ecological notes.

The type specimen was collected in a rhododendron thicket near a stream (Hoffman 1950) on Satulah Mountain in the town of Highlands, North Carolina. Elevation notes on the labels of other specimens range from 690 meters to 1311 meters, and may indicate that *N.austricola* is restricted to cool, high elevation habitats.

##### Etymology.

The specific epithet derives from the Latin word *australis*, meaning “of the south,” and was named for being the most southern species of *Nannaria* known at the time (Hoffman 1950).

#### ﻿ Nannaria cymontana sp. nov.

Taxon classificationAnimaliaPolydesmidaXystodesmidae

DFABA897-957B-5CCD-BD22-962B82F6A7AE

http://zoobank.org/46B272E0-9D49-48D6-B172-33415A9C7640

**Figure 14.**
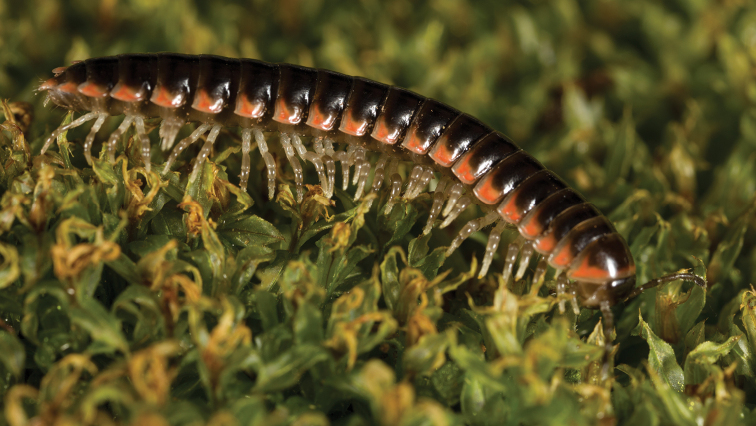
*Nannariacymontana* sp. nov., in situ, male holotype (MPE00460) from Floyd County, Virginia.

**Figure 15.**
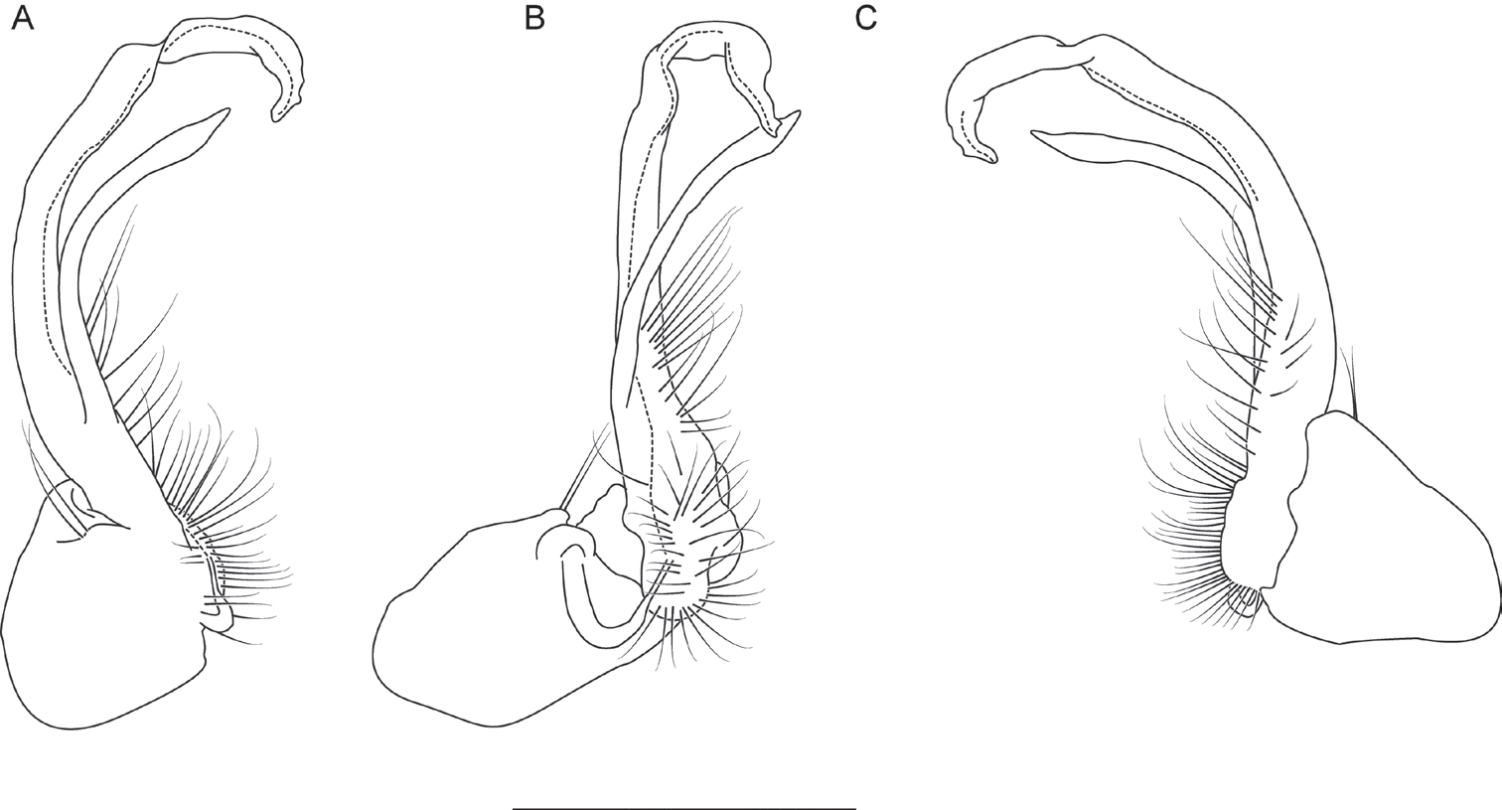
Left gonopod of *Nannariacymontana* sp. nov., male holotype (MPE00460, Floyd County, Virginia) **A** anterior view **B** medial view **C** posterior view. Scale bar: 1 mm.

Vernacular name: The Blue Ridge Twisted-Claw Millipede Figs 14 , 15

##### Material examined.

**Type material: *Holotype***: United States – **Virginia** • ♂; Floyd County, bottomland in Rocky Knob Recreation Area near Rock Castle Creek; 36.7856°N, -80.3726°W, ±2m; elev. 830 m; 27 May 2015; J. C. Means, A. Chazal leg.; ex forest with ferns, yam, witch-hazel, oak, magnolia, maple; VTEC, MPE00460. ***Paratypes***: United States – **Virginia** • 1 ♂; Floyd County, Rocky Knob Picnic Area, on hill edge by side of road; 36.8132°N, -80.3495°W, ±3m; elev. 970 m; 18 September 2015; J. C. Means, P. E. Marek, K. Lawler, P. Shorter, V. Wong leg.; much undergrowth in chestnut oak woods; VTEC, MPE00822. • 4 ♂♂; Floyd County, Rocky Knob Park; 36.8195°N, -80.3422°W, ±3000m; 3 July 1947; R. L. Hoffman leg.; VMNH, NAN0279. **Non type material**: United States – **Virginia** • 5 ♂♂ and 2 ♀♀; Carroll County, Blacksnake Meadery, bottomland near creek at entrance; 36.776°N, -80.5446°W; 14 September 2014; J. C. Means leg.; VTEC, MPE00200, MPE00205, MPE00209, MPE00218, MPE00220, MPE00206, MPE00207 • 1 specimen; Floyd County, Buffalo Mtn.; 36.7869°N, -80.4509°W, ± 3000m; elev. 1067 m; 5 October 1997; VMNH survey leg.; VMNH, NAN0086 • 1 ♂; Floyd County, Buffalo Mountain, pitfall site on north slope; 36.7958°N, -80.4772°W, ± 3000m; elev. 1067 m; 19 August to 1 October 1992; VMNH survey leg.; VMNH, NAN0281 • 1 ♂; Floyd County, Buffalo Mtn.; 36.7958°N, -80.4772°W, ± 3000m; elev. 1067 m; 24 October 1958; R. L. Hoffman leg.; VMNH, NAN0282 • 1 ♂; Floyd County, Willis Ridge, w of Floyd, Rt. 892; 36.9328°N, -80.4046°W, ± 3000m; 17 June 1990; J. M. Anderson, R. L. Hoffman leg.; VMNH, NAN0283 • 1 ♂; Floyd County, Willis Ridge, ca. 4 mi. west of Floyd CH; 36.9328°N, -80.4046°W, ± 3000m; elev. 914 m; 5 September 1982; R. L. Hoffman leg.; VMNH, NAN0284 • 1 ♂; Floyd County, Felker’s property Rt 726; 36.8008°N, -80.3988°W, ± 10000m; 3 June 1995; J. M. Anderson leg.; VMNH, NAN0285 • 1 ♂; Floyd County, Mile 176, Blue Ridge Parkway; 36.7501°N, -80.4047°W, ± 3000m; 6 June 1976; R. L. Hoffman leg.; VMNH, NAN0287 • 3 specimens; Floyd County, 2 mi. SW of Copper Valley; 36.9703°N, -80.5426°W, ± 5000m; 15 October 1974; R. L. Hoffman leg.; VMNH, NAN0288 • 2 specimens; Floyd County, Chestnut Creek Natural Area Preserve, off Rt. 771, 4 km SE of Willis; 36.8373°N, -80.4421°W, ± 4000m; 15 October 2007; S. M. Roble, R. L. Hoffman leg.; VMNH, NAN0290 • 1 ♂ and 2 ♀♀; Floyd County, Blue Ridge Parkway mile post 174.3, private gravel drive crossing Laurel Creek; 36.7716°N, -80.4047°W; elev. 916 m; 24 June 2014; P. E. Marek, E. Francis, J. C. Means leg.; VTEC, MPE00071, MPE00057, MPE00060 • 1 ♂; Floyd County, hardwood opening in rhododendron forest off Blue Ridge Pkwy south of Mabry Mill; 36.7448°N, -80.3994°W, ± 2m; elev. 923 m; 26 May 2015; J. C. Means, P. E. Marek, A. Chazal, P. L. Shorter leg.; VTEC, MPE00450 • 1 ♀; Floyd County, off Buffalo Mtn. Road intersection with Blue Ridge Pkwy; 36.7724°N, -80.4054°W, ± 3m; elev. 912 m; 26 May 2015; J. C. Means, P. E. Marek, A. Chazal, P. L. Shorter leg.; VTEC, MPE00452 • 1 ♂; Floyd County, 2 km north of Mabry Mill on Blue Ridge Parkway; 36.7678°N, -80.4049°W, ± 2m; elev. 913 m; 27 May 2015; J. C. Means, A. Chazal leg.; VTEC, MPE00476 • 1 ♂; Floyd County, 0.8 km SW Mabry Mill; 36.7437°N, -80.4097°W, ± 4m; elev. 961 m; 27 May 2015; J. C. Means leg.; VTEC, MPE00488 • 1 ♂; Floyd County, right side of gravel road off Blue Ridge Pkwy. in a marshy area between two streams; 36.7796°N, -80.3984°W, ± 3m; elev. 937 m; 30 June 2015; J. C. Means, P. L. Shorter leg.; VTEC, MPE00720 • 2 ♂♂; Floyd County, Rocky Knob Recreation Area; 36.7848°N, -80.3733°W; 19 September 2015; J. C. Means, P. E. Marek leg.; VTEC, MPE00826, MPE03707 • 3 ♂♂ and 1 ♀; Floyd County, 3.5 km NNE Mabry Mill, gully behind farm off Blue Ridge Parkway, on small island in creek; 36.7825°N, -80.3997°W; elev. 945 m; 26 April 2017; J. C. Means, P. L. Shorter leg.; VTEC, MPE02513, MPE02514, MPE03670, MPE02504 • 1 ♂; Floyd County, 3.7 km NNE Mabry Mill; 36.7836°N, -80.4°W; elev. 975 m; 26 April 2017; J. C. Means, P. L. Shorter leg.; VTEC, MPE02524 • 1 ♂; Floyd County, Rhododendron/oak forest behind Mabry Mill off Blue Ridge Parkway; 36.7505°N, -80.405°W; elev. 869 m; 26 April 2017; J. C. Means, P. L. Shorter leg.; VTEC, MPE02542 • 1 ♂; Floyd County, 3.5 km NNE Mabry Mill, slight clearing by rhododendron cove on Laurel Creek by Blue Ridge Parkway; 36.7805°N, -80.395°W; elev. 918 m; 4 May 2017; J. C. Means, P. L. Shorter leg.; VTEC, MPE02628 • 1 ♂; Montgomery County, Trail to Bottom Creek at end of VA. Rt 637; 37.1161°N, -80.2051°W, ± 1000m; 6 May 1990; R. L. Hoffman leg.; VMNH, NAN0304 • 3 ♂♂; Montgomery County, Riner: in wetland below lake; 36.9662°N, -80.4179°W; elev. 773 m; 17 October 2014; J. C. Means leg.; VTEC, MPE00234, MPE00243, MPE00245 • 5 specimens; Patrick County, Pinnacles of Dan; 36.6738°N, -80.4388°W, ± 1807m; 15 October 1950; L. Hubricht leg.; VMNH, NAN0277 • 4 specimens; Patrick County, along Laurel Creek, MP 174.5, Blue Ridge Parkway; 36.7676°N, -80.4062°W, ± 3000m; 20 May 1983; R. L. Hoffman leg.; VMNH, NAN0278 • 2 specimens; Patrick County, Below Townes Dam, Pinnacles of Dan; 36.6859°N, -80.4308°W, ± 3000m; 22 April 1972; R. L. Hoffman leg.; VMNH, NAN0280 • 1 specimen; Patrick County, 1.5 miles NW of Patrick Springs; 36.6571°N, -80.2142°W, ± 3000m; 16 November 1952; L. Hubricht leg.; VMNH, NAN0286 • 1 specimen; Patrick County, Pinnacles of Dan, 4 miles SW of Vesta; 36.6756°N, -80.4092°W, ± 5000m; 19 April 1957; R. L. Hoffman leg.; VMNH, NAN0289 • 3 ♂♂; Patrick County, Rock Castle Creek, Rock Castle Gorge Trail, Rocky Knob Recreation Center; 36.7858°N, -80.3717°W, ± 7m; elev. 839 m; 21 September 2015; J. C. Means, P. E. Marek leg.; VTEC, MPE00828, MPE00829, MPE00831 • 1 ♂; Patrick County, Pinnacles of Dan area, top of gated road leading to Townes Reservoir, Lower Dam Rd., SW of Meadows of Dan; 36.6866°N, -80.4415°W, ± 5m; elev. 842 m; 5 June 2016; D. A. Hennen leg.; VTEC, MPE02059 • 3 ♂♂; Roanoke County, Bradshaw Creek, ca. 5 miles N of LaFayette; 37.2991°N, -80.2238°W, ± 5000m; 1 March 1957; R. L. Hoffman leg.; VMNH, NAN0303 • 3 ♂♂ and 1 ♀; Roanoke County, Poor Mtn.; 37.1975°N, -80.1525°W, ± 2000m; 15 October 1956; R. L. Hoffman leg.; VMNH, NAN0305 • 1 ♂ and 2 ♀♀; Roanoke County, Side of road & gully off Blue Ridge Parkway, Poor Mountain view pull off; 37.1369°N, -80.1108°W; elev. 899 m; 26 April 2017; J. C. Means, P. L. Shorter leg.; VTEC, MPE02495. Complete material examined information listed in Suppl. material 1.

##### Diagnosis.

Adults of *Nannariacymontana* sp. nov. can be separated from the geographically close and morphologically similar species *N.ericacea*, *N.lutra* sp. nov., and *N.wilsoni* by the following characters. Acropodite arc gradually curving, not straight with an abrupt bend at tip as in *N.ericacea*. Acropodite medial flange lacking a dorsal median projection, not with a rounded projection as in *N.lutra* sp. nov. Acropodite medial flange small rather than large and laminate as in *N.wilsoni*, acropodite tip lateral flange sharp instead of rounded as in *N.wilsoni*, and prefemoral process tip spear-shaped, not gradually tapered as in *N.wilsoni*.

##### Description.

Suppl. material 2. Based on holotype (♂) MPE00460 and paratype (♀) MPE00452.

**Measurements**: Taken from holotype (♂) MPE00460: BL = 27.60, CW = 3.72, IW = 2.70, ISW = 0.99, B10W = 4.60, B10H = 2.85. **Color.** Tergites with two paranotal red spots, collum outlined in red, and tergites with background black (Fig. 14). **Gonopods.** Male gonopod acropodite arc gradually curving (Fig. 15A–C). Acropodite gently twisted at anterior bend (Fig. 15A). Acropodite medial flange slightly lobed (Fig. 15C). Acropodite tip medial flange absent, acropodite tip lateral flange slightly bilobed, both projections rounded and small (Fig. 15A). Acropodite tip blunt, entire, directed ventrally (Fig. 15B). Prefemoral process sinuous and slightly spear-shaped distally, with tip directed medially (Fig. 15A). **Cyphopods.** Female cyphopod receptacle triangular.

##### Variation.

The size of the acropodite tip lateral flanges differs slightly among specimens, ranging from small to medium-sized.

##### Distribution.

Known from the following Virginia counties: Carroll, Floyd, Montgomery, Patrick, and Roanoke (Fig. 51).

##### Ecological notes.

This species has been collected most often from rhododendron cove habitats, but is also sometimes found in mesic mixed forests.

##### Etymology.

This species is named for its type locality, the Blue Ridge Parkway, an invaluable stretch of protected land which provides recreation for millions of people a year and conserves over 370 km^2^ of natural habitat. The word *cymontana* is a feminine adjective, a combination of the Latin *cymatilis* meaning blue and *montanus* meaning “of mountains.”

#### ﻿ Nannaria ericacea

Taxon classificationAnimaliaPolydesmidaXystodesmidae

Hoffman, 1949

6F786C37-9394-5550-8EC3-162480AF1397

**Figure 16.**
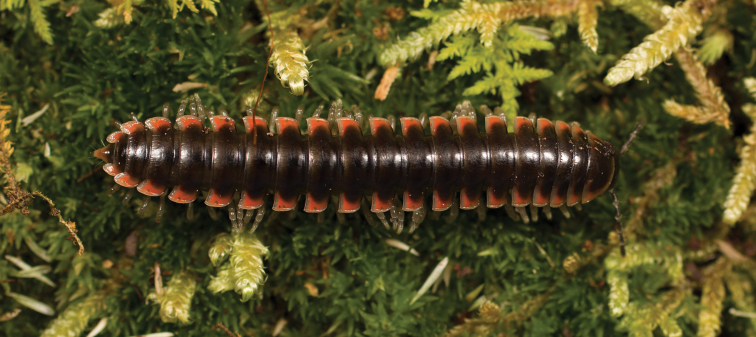
*Nannariaericacea*, in situ, male (MPE02263) from Roanoke County, Virginia.

**Figure 17.**
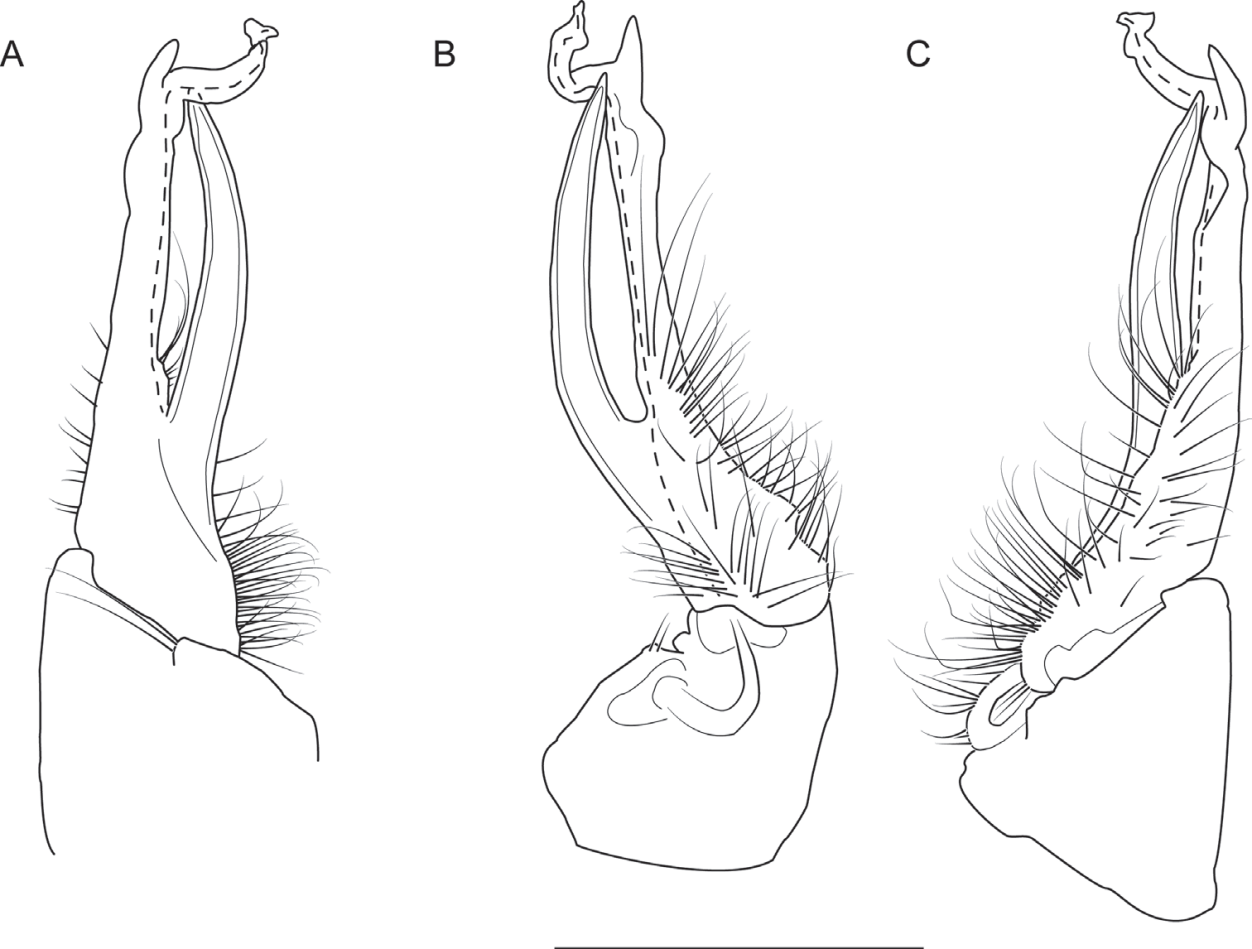
Left gonopod of *Nannariaericacea* (MPE02263, Roanoke County, Virginia) **A** anterior view **B** medial view **C** posterior view. Scale bar: 1 mm.

Vernacular name: The Heathland Twisted-Claw Millipede Figs 16 , 17

Nannaria ericacea Hoffman, 1949b: 381, figs 9, –10. Chamberlin and Hoffman 1958: 40. Hoffman 1999: 366 (351 in pdf version). Means et al. 2021a: 4, 5, S67, fig. 3. Means et al 2021b: 6, 9, figs 2, 4F.

##### Type material.

***Holotype***: United States – **Virginia** • ♂; Alleghany County, McGraw Gap, 3 miles [4.8 km] northwest of Clifton Forge; 37.858°N, - 79.864°W, ±1000m; 13 April 1947; R. L. Hoffman leg.; USNM Entomology, no. 1784; (non vidi). ***Paratypes***: United States – **Virginia** • 1 ♂; same collection data as for holotype; 1 June 1948; VMNH, NAN0366 (vidi) • 1 ♂ and 1 ♀; same collection data as for holotype; 27 April 1947; VMNH, NAN0367 (vidi). Hoffman (1949b) indicated that he collected “several topoparatypes” for his personal collection from the type locality and listed three specimens with his personal collection codes “RLH Nos. 4-2747-1, 5-1847-1c, and 6-147-1a.” The VMNH specimens included here, NAN0366 and NAN0367, represent these specimens. The jar that contains these specimens is labeled “PARATYPE” and the vials each have labels reading “TOPOPARATYPES.”

##### Material examined.

**Non type material**: United States – **Virginia** • 1 ♂; Alleghany County, Pott’s Mtn. Pond; 37.602°N, -80.1385°W, ± 5000m; elev. 1097 m; 2 January 1954; R. L. Hoffman leg.; VMNH, NAN0441 • 2 specimens; Alleghany County, Top of Little Mtn, east of Natural Well on FS 342; 37.9223°N, -79.9134°W, ± 3000m; 30 January 2002; M. W. Donahue leg.; VMNH, NAN0446 • 50 specimens; Bath County, Douthat State Park; 37.8967°N, -79.8022°W; 13 September 1988; R. M. Shelley leg.; NCSM, NAN0465 • 2 ♂♂; Bath County, 5.4 km NW Warm Springs, Meadow Lane Farm; 38.0792°N, -79.8358°W; elev. 577 m; 8 April 2017; J. C. Means leg.; VTEC, MPE02434, MPE03672 • 1 specimen; Botetourt County, Roaring Run DF site, ca. 7 mi. NW Eagle Rock; 37.7079°N, -79.8933°W, ± 2000m; 22 August to 21 September 1996; M. W. Donahue, R. S. Hocan leg.; VMNH, NAN0436 • 1 specimen; same collection data as for preceding; 22 September to 26 October 1996; VMNH, NAN0438 • 1 ♂; Botetourt County, Jefferson National Forest, along forest road just north of Blackhorse Gap; 37.4256°N, -79.7578°W; elev. 727 m; 11 June 2017; C. W. Harden leg.; VTEC, MPE02861 • 1 specimen; Craig County, West side Johns Creek Mountain near Maggie; 37.3982°N, -80.3865°W, ± 5000m; 20 April 1976; R. L. Hoffman, et fils leg.; VMNH, NAN0443 • 2 specimens; Craig County, Barbours Creek, NW of Newcastle; 37.5536°N, -80.0576°W, ± 5000m; June 1947; R. L. Hoffman leg.; VMNH, NAN0447 • 2 ♂♂; Craig County, Appalachian Trail parking lot near Caldwell Fields campground. Side of stream, off 621; 37.3795°N, -80.2503°W; elev. 490 m; 13 June 2018; J. C. Means leg.; VTEC, MPE04304, MPE04309 • 1 ♂ and 1 ♀; Giles County, 4 miles N Pembroke, Cascades Recreation Area, Jefferson National Forest; 37.3535°N, -80.5994°W; 3 August 1981; R. M. Shelley leg.; NCSM, NAN0460 • 2 specimens; Montgomery County, Brush Mtn. nr. Blacksburg; 37.2822°N, -80.465°W, ± 5000m; 28 November 1960; R. L. Hoffman leg.; VMNH, NAN0431 • 2 specimens; Montgomery County, RAAP - Radford, 30 m E of RDAISA bldg.; 37.1859°N, -80.5366°W, ± 3000m; 8 May 1998; S. Garriock leg.; VMNH, NAN0432 • 2 specimens; Montgomery County, Blacksburg; 37.221°N, -80.4162°W, ± 500m; 26 September 1957; R. L. Hoffman leg.; VMNH, NAN0434 • 1 specimen; same collection data as for preceding; 28 October 1956; VMNH, NAN0435 • 2 specimens; same collection data as for preceding; 26 October 1957; VMNH, NAN0442 • 4 specimens; same collection data as for preceding; 13 April 1958; R. L. Hoffman, R. Crabill leg.; VMNH, NAN0448 • 1 ♀; Montgomery County, Dry Run, 5 mi. NE of Blacksburg; 37.2792°N, -80.3143°W, ± 2000m; 20 April 1957; R. L. Hoffman leg.; VMNH, NAN0440 • 1 specimen; Montgomery County, 1 mi. NE of Vicker Station; 37.1738°N, -80.4751°W, ± 4000m; 11 April 1964; R. L. Hoffman leg.; VMNH, NAN0444 • 1 specimen; Montgomery County, Trillium Vale, Blacksburg; 37.2286°N, -80.3902°W, ± 1000m; 10 October 1950; R. L. Hoffman leg.; VMNH, NAN0445 • 2 ♂♂ and 1 ♀; Montgomery County, Gateway Trail, Jefferson National Forest on Brush Mtn.; 37.2488°N, -80.4604°W; 18 May 2014; P. E. Marek leg.; VTEC, MPE00032, MPE00038, MPE00039 • 3 ♂♂ and 1 ♀; Montgomery County, Stadium Woods, Virginia Tech Campus; 37.22°N, -80.4156°W; elev. 640 m; 15 June 2014; P. E. Marek leg.; VTEC, MPE00040, MPE00042, MPE00043, MPE00041 • 2 ♂♂; Montgomery County, Gateway Trail, 1/2 mile in from main trail by stream; 37.2506°N, -80.461°W, ± 3m; elev. 642 m; 26 September 2014; J. C. Means, P. E. Marek, E. Francis, K. Lawler, N. Zegler leg.; VTEC, MPE00226, MPE00229 • 1 ♂; Montgomery County, Golden Hills Disc Golf course, in gully; 37.1729°N, -80.4078°W, ± 3m; elev. 628 m; 8 November 2014; J. C. Means, D. A. Hennen, P. E. Marek leg.; VTEC, MPE00276 • 1 ♂; Montgomery County, On Price Mtn., north side, in gully off old road; 37.1916°N, -80.4577°W, ± 3m; elev. 674 m; 28 July 2015; P. E. Marek, P. L. Shorter, J. C. Means, V. Wong leg.; VTEC, MPE00765 • 2 ♂♂; Montgomery County, Pandapas Pond, in bottomland below horse trail parking lot; 37.2824°N, -80.4485°W; 22 September 2015; J. C. Means leg.; VTEC, MPE00835, MPE00837 • 1 ♂; Montgomery County, Blacksburg: Along the edge of the Duck Pond, Virginia Tech Campus; 37.2251°N, -80.4272°W; 15 October 2015; K. Lawler leg.; VTEC, MPE00896 • 1 ♀; Montgomery County, Blacksburg: Virginia Tech campus, Stadium Woods; 37.2208°N, -80.4164°W; 12 October 2015; K. Lawler leg.; VTEC, MPE00897 • 2 ♂♂ and 4 ♀♀; Montgomery County, Jefferson Nat’l Forest, off Craig Creek Rd. (621); 37.3477°N, -80.3251°W, ± 2m; elev. 599 m; 6 October 2016; P. L. Shorter, P. E. Marek, J. C. Means, V. Wong leg.; VTEC, MPE02145, MPE02155, MPE02153, MPE02154, MPE02156, MPE02157 • 5 ♂♂; Montgomery County, Blacksburg: Stadium Woods; 37.2208°N, -80.4167°W; elev. 635 m; 9 November 2017; J. C. Means, D. A. Hennen, G. G. Schiermeyer leg.; VTEC, MPE03459, MPE03460, MPE03461, MPE03462, MPE03463 • 1 ♀; Montgomery County, Stadium Woods; 37.2208°N, -80.4167°W; 27 October 2017; G. G. Schiermeyer leg.; VTEC, MPE03676 • 1 ♂; Montgomery County, Gateway Trail, near entrance; 37.2488°N, -80.4604°W; elev. 603 m; 25 September 2013; P. E. Marek leg.; VTEC, MPE03716 • 2 ♂♂; Montgomery County, Blacksburg, end of Valley View Drive near quarry; 37.2218°N, -80.3894°W, ± 3m; elev. 680 m; 13 September 2019; F. Vasquez, J. C. Means, P. E. Marek, D. A. Hennen, I. Huerta leg.; VTEC, MPE04999, MPE05000 • 1 specimen; Roanoke County; 37.28°N, -80.05°W; 7 April 1965; VMNH, NAN0437 • 1 specimen; Roanoke County, Along Rt. 624, 4.5 miles SW of Catawba; 37.3512°N, -80.1737°W, ± 1000m; 9 April 1964; R. L. Hoffman leg.; VMNH, NAN0439 • 3 ♂♂ and 1 ♀; Roanoke County, Salem, Carvin’s Cove, down a huge gully off the Trough Trail, bank of stream; 37.3599°N, -79.9939°W; elev. 451 m; 20 November 2016; J. C. Means leg.; VTEC, MPE02263, MPE02264, MPE03711, MPE02265. Complete material examined information listed in Suppl. material 1.

##### Diagnosis.

Adults *of Nannariaericacea* can be separated from the geographically close and morphologically similar species *N.liriodendra* sp. nov. and *N.paraptoma* sp. nov. by the following characters. Acropodite arc straight, with abrupt bend at tip, rather than gradually curving as in *N.liriodendra* sp. nov. and *N.paraptoma* sp. nov. Acropodite tip medial flange triangular, not as long as the dorsal triangular projection of *N.paraptoma* sp. nov.

##### Description.

Suppl. material 2. Based on (♂) MPE02263 and (♀) MPE02265.

**Measurements**: Taken from (♂) specimen MPE02263: BL = 38.48, CW = 4.35, IW = 3.12, ISW = 0.85, B10W = 5.63, B10H = 3.44. **Color.** Tergites with two paranotal orange-red spots, collum outlined in orange-red, and tergites with background black to brown (Fig. 16). **Gonopods.** Male gonopod acropodite arc straight, with abrupt bend distally (Fig. 17A–C). Acropodite anterior bend with gentle twist (Fig. 17C), acropodite medial flange absent. Acropodite tip medial flange a large triangular process, slightly curved (Fig. 17C). Acropodite tip lateral flange triangular (Fig. 17A). Acropodite tip entire, slightly expanded with small lateral and medial lobes (Fig. 17A), acropodite tip directed ventrally. Prefemoral process simple, curving (Fig. 17A), with tip directed cephalically (Fig. 17B). **Cyphopods.** Female cyphopod receptacle triangular.

##### Variation.

Acropodite tip sometimes lacking small distal lobes, size of acropodite medial flange typically large, but sometimes smaller.

##### Distribution.

Restricted to Virginia, *Nannariaericacea* has been recorded from the following counties: Alleghany, Bath, Botetourt, Craig, Giles, Montgomery, and Roanoke (Fig. 51).

##### Ecological notes.

*Nannariaericacea* has been collected in a variety of habitats, ranging from pine-oak forests to ericaceous forests of rhododendron and hemlock. Elevation records range from 451 meters to 1097 meters. Hoffman (1949b) provided a thorough description of the habitat at the type locality, describing it as “a deep watergap in sandstone ridges, with the forest composed chiefly of *Tsugacanadensis*, *Liriodendrontulipifera*, *Quercusalba*, *Q.prinus* [now *Quercusmontana* Willd.], *Acerrubrum*, *A.pennsylvanicum*, *Rhododendronmaximum*, and *Kalmialatifolia*. Herbaceous plants at the type locality include *Urticadioica*, *Mitelladiphylla*, *Mitchellarepens*, and the ferns *Polypodiumvirginianum* and *Polystichumacrostichoides*.”

##### Etymology.

The specific epithet *ericacea* derives from the Greek *ereike*, heather, referring to the flowering plant family Ericaceae, known as the heath or heather family. The name was given based on the abundance of this species in ericaceous habitats (Hoffman 1949b).

#### ﻿ Nannaria filicata sp. nov.

Taxon classificationAnimaliaPolydesmidaXystodesmidae

90D76E0D-1057-5DD1-B6D6-AB7E9B373AA6

http://zoobank.org/0C195C72-A567-472B-99A5-3952628273AC

**Figure 18.**
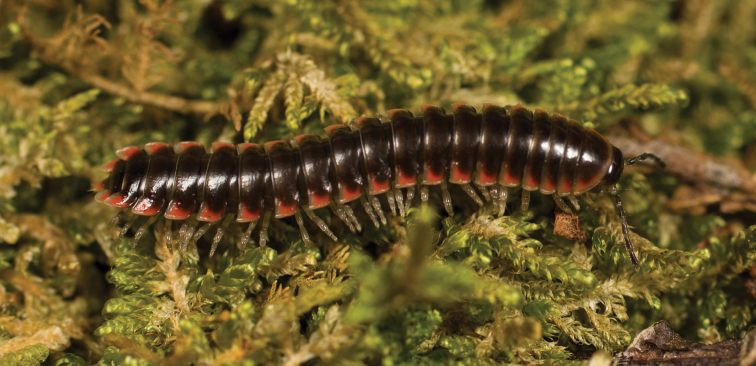
*Nannariafilicata* sp. nov., in situ, male (MPE02451) from Allegheny County, Virginia.

**Figure 19.**
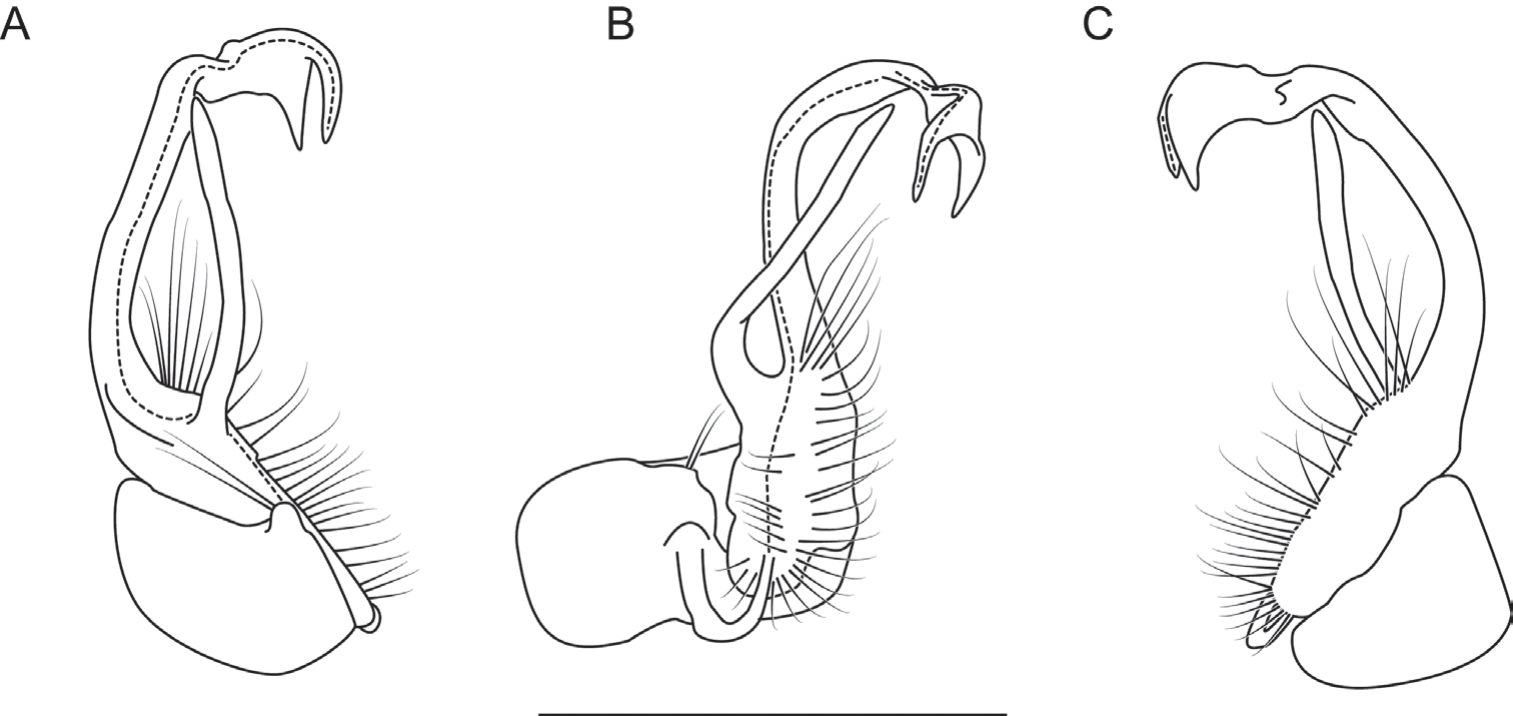
Left gonopod of *Nannariafilicata* sp. nov., male holotype (MPE02110, Allegheny County, Virginia) **A** anterior view **B** medial view **C** posterior view. Scale bar: 1 mm.

Vernacular name: The Fern Twisted-Claw Millipede Figs 18 , 19

##### Material examined.

**Type material: *Holotype***: United States – **Virginia** • ♂; Alleghany County, McGraw Gap, about 4 km NW of Clifton Forge on RT 606 at fishing area in George Washington National Forest along Smith Creek; 37.8526°N, -79.8512°W, ±9 m; elev. 428 m; 21 June 2016; D. A. Hennen, J. C. Means leg.; dry deciduous litter near stream in forest of oak, rhododendron, maple, mountain laurel, and witch hazel, soil a bit sandy; VTEC, MPE02110. ***Paratypes***: United States – **Virginia** • 1 ♂ and 1 ♀; same collection data as for holotype; VTEC, MPE01824, MPE01830 • 1 ♂ and 1 ♀; Alleghany County, McGraw Gap, ca 3 mi. [4 km] NW Clifton Forge; 37.8580°N, -79.8661°W, ±4000m; 27 April 1947; R. L. Hoffman leg.; VMNH, NAN0217. **Non type material**: United States – **Virginia** • 2 specimens; Alleghany County, Big Knob, Warm Springs Mtn, ca. 4.5 mi. NE of Covington; 37.8486°N, -79.9272°W, ± 4000m; elev. 1241 m; 15 May 1949; R. L. Hoffman leg.; VMNH, NAN0214 • 4 ♂♂ and 3 ♀♀; Alleghany County, McGraw Gap, about 4 km NW of Clifton Forge on Rt 606 at fishing area in George Washington National Forest along Smith Creek; 37.8526°N, -79.8512°W, ± 9m; elev. 428 m; 21 June 2016; D. A. Hennen, J. C. Means leg.; VTEC, MPE02231, MPE02451, MPE02457, MPE02458, MPE01831, MPE01832, MPE01833. Complete material examined information listed in Suppl. material 1.

##### Diagnosis.

Adults of *Nannariafilicata* sp. nov. can be separated from the geographically close and morphologically similar species *N.ericacea*, *N.orycta* sp. nov., and *N.vellicata* sp. nov. by the following characters. Acropodite arc gradually curving, not straight with abrupt bend at tip as in *N.ericacea* and *N.vellicata* sp. nov. Acropodite anterior bend only slightly twisted, not acutely bent as in *N.orycta* sp. nov. Acropodite tip medial flange directed caudally, rather than medially as in *N.vellicata* sp. nov.

##### Description.

Suppl. material 2. Based on holotype (♂) MPE02110 and paratype (♀) MPE01830.

**Measurements**: Taken from holotype (♂) MPE02110: BL = 18.90, CW = 3.25, IW = 2.25, ISW = 0.78, B10W = 3.56, B10H = 2.19. **Color.** Tergites with two paranotal red-orange spots, collum outlined in red-orange, and tergites with background chestnut brown (Fig. 18). **Gonopods.** Male gonopod acropodite arc a gradual curve (Fig. 19A–C). Acropodite with a smooth helical twist at apical bend (Fig. 19C). Acropodite medial flange slightly lobed (Fig. 19C). Acropodite tip medial flange large, laminate and produced into a caudally-directed pointed tip larger than acropodite tip (Fig. 19C). Acropodite tip lateral flange absent. Acropodite tip directed caudally (Fig. 19B). Prefemoral process simple, gradually curving, and crossing acropodite ventrolaterally (Fig. 19B). Prefemoral process tip directed laterally (Fig. 19A). **Cyphopods.** Female cyphopod receptacle enlarged into a triangular hood, covering cyphopod valves.

##### Variation.

No noticeable variation observed.

##### Distribution.

Only known from a small area in Allegheny County, Virginia (Fig. 51).

##### Ecological notes.

Label data indicates this species is found in mesic deciduous forests and rhododendron coves from 428 m to 1241 m in elevation.

##### Etymology.

The specific name is a feminine adjective derived from the Latin *filicatus*, meaning “adorned with ferns.” This is in reference to the many ferns observed at its collection localities, with *Polystichumacrostichoides* (Michx.) Schott typically being the dominant fern species.

#### ﻿ Nannaria liriodendra sp. nov.

Taxon classificationAnimaliaPolydesmidaXystodesmidae

CECD4430-6019-50CC-87AB-42C85C6839D3

http://zoobank.org/EE80123A-E236-407F-B4C2-177B4C2AF706

**Figure 20.**
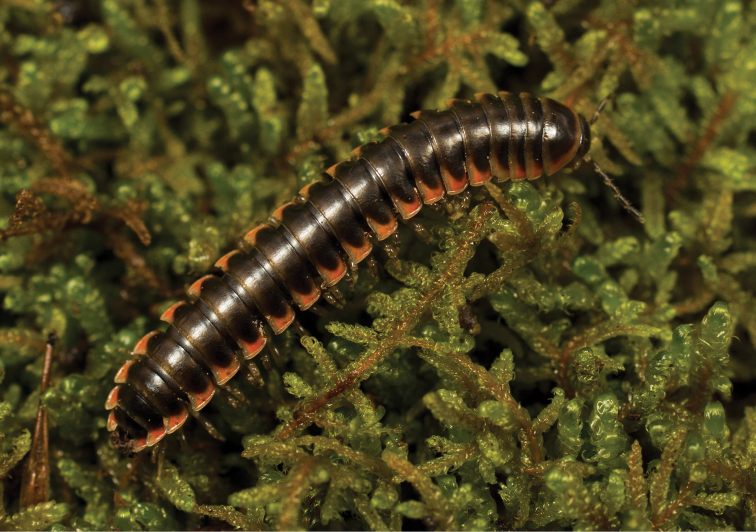
*Nannarialiriodendra* sp. nov., in situ, male holotype (MPE04200) from Craig County, Virginia.

**Figure 21.**
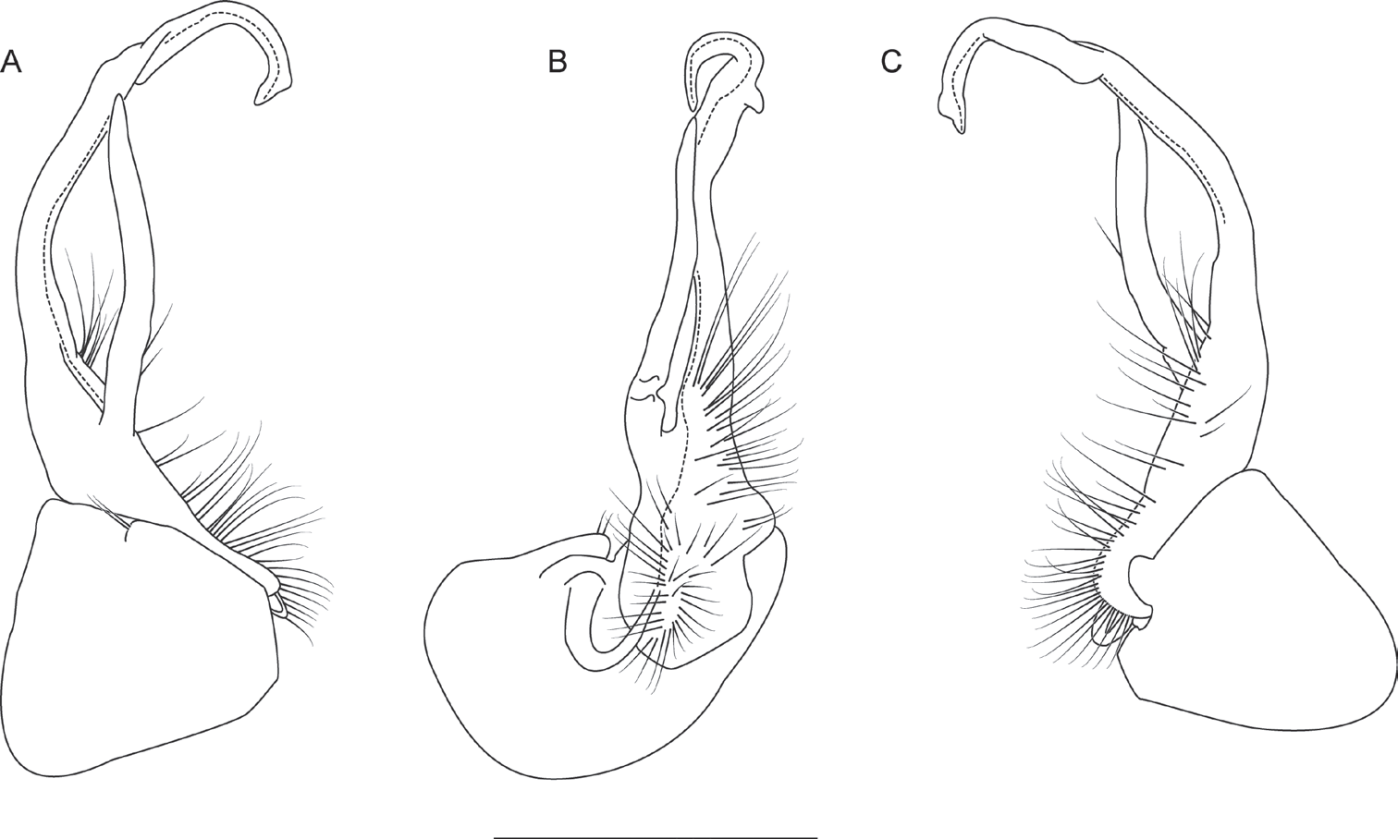
Left gonopod of *Nannarialiriodendra* sp. nov., male holotype (MPE04200, Craig County, Virginia) **A** anterior view **B** medial view **C** posterior view. Scale bar: 1 mm. (Note: the tip of the gonopod acropodite is broken in the holotype specimen, so the tip of a male paratype (NAN0057) was used to illustrate its shape).

Vernacular name: The Tuliptree Twisted-Claw Millipede Figs 20 , 21

##### Material examined.

**Type material: *Holotype***: United States – **Virginia** • ♂; Craig County, Potts Cove, along Cove Branch, next to jeep trail; 37.5833°N, -80.1604°W, ±6m; elev. 772 m; 25 June 2018; D. A. Hennen, J. C. Means, K. Williamson, P. E. Marek leg.; forest of tulip tree, oak, persimmon; VTEC, MPE04200. ***Paratypes***: United States – **Virginia** • 1 ♂ and 1 ♀; same collection data as for holotype; VTEC, MPE04316, MPE04201 • 3 ♂♂ and 1 ♀; Craig County, top of Potts Mtn., E of Paint Bank; 37.6012°N, -80.1533°W, ±5000m; 13 April 1962; R. L. Hoffman leg.; VMNH, NAN0057. **Non type material**: United States – **Virginia** • 14 ♂♂ and 10 ♀♀; Craig County, Potts Cove, Cove Branch, next to jeep trail; 37.5833°N, -80.1604°W, ± 6m; elev. 772 m; 25 June 2018; K. Williamson, D. A. Hennen, J. C. Means, P. E. Marek leg.; VTEC, MPE04310, MPE04317, MPE04318, MPE04319, MPE04320, MPE04321, MPE04322, MPE04323, MPE04324, MPE04325, MPE04326, MPE04327, MPE04328, MPE04329, MPE04202, MPE04203, MPE04204, MPE04205, MPE04330, MPE04331, MPE04332, MPE04333, MPE04334, MPE04335. Complete material examined information listed in Suppl. material 1.

##### Diagnosis.

Adults of *Nannarialiriodendra* sp. nov. can be separated from the geographically close and morphologically similar species *N.acroteria* sp. nov. and *N.ericacea* by the following characters. Acropodite arc gradually curving, not abruptly bent distally as in *N.ericacea*. Acropodite medial flange lacking a dorsal projection, rather than having a triangular projection as in *N.acroteria* sp. nov. Prefemoral process crimped and with an indentation near base.

##### Description.

Suppl. material 2. Based on holotype (♂) MPE04200 and paratype (♀) MPE4201.

**Measurements**: Taken from holotype (♂) MPE04200: BL = 35.70, CW = 4.70, IW = 3.32, ISW = 1.12, B10W = 5.75, B10H = 3.13. **Color.** Tergites with two paranotal red spots, collum outlined in red, and tergites with background black (Fig. 20). **Gonopods.** Male gonopod acropodite arc gradually curving (Fig. 21A–C). Acropodite anterior bend with gentle twist (Fig. 21A). Acropodite medial flange lobed (Fig. 21C), somewhat triangular in medial view (Fig. 21B). Acropodite tip medial flange absent, acropodite tip lateral flange lobed, rounded (Fig. 21A). Acropodite tip directed caudally (Fig. 21B). Prefemoral process slightly sinuous (Fig. 21A) and crossing acropodite dorsolaterally (Fig. 21A). Prefemoral process with slight indentation near base, appearing crimped and bent (Fig. 21B). Prefemoral process tip directed cephalically (Fig. 21A). **Cyphopods.** Female cyphopod receptacle an enlarged triangular hood, covering cyphopod valves.

##### Variation.

No noticeable variation observed.

##### Distribution.

Only known from the vicinity of Potts Mountain, in Craig County, Virginia (Fig. 51).

##### Ecological notes.

The forest at the type locality is a deciduous oak-tuliptree forest surrounding a stream.

##### Etymology.

The specific name is a feminine adjective derived from the Greek words *leirion* for lily and *dendron* for tree. It is a reference to the tuliptree, *Liriodendrontulipifera* L., known for its flowers that resemble tulips. This beautiful tree is commonly encountered during millipede collecting trips in the eastern U.S.

#### ﻿ Nannaria lithographa sp. nov.

Taxon classificationAnimaliaPolydesmidaXystodesmidae

9D2B40B3-4DDB-5016-A147-07262BD26948

**Figure 22.**
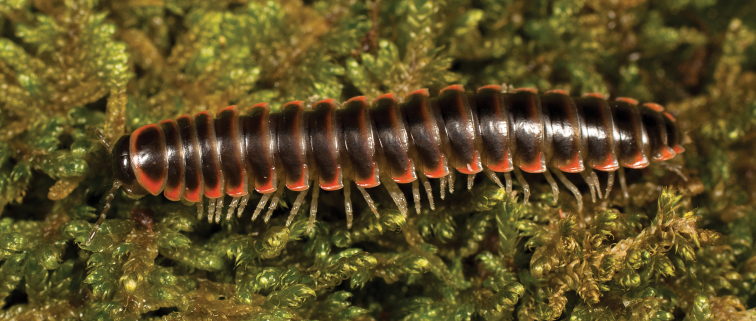
*Nannarialithographa* sp. nov., in situ, female paratype (MPE03296) from Rutherford County, North Carolina.

**Figure 23.**
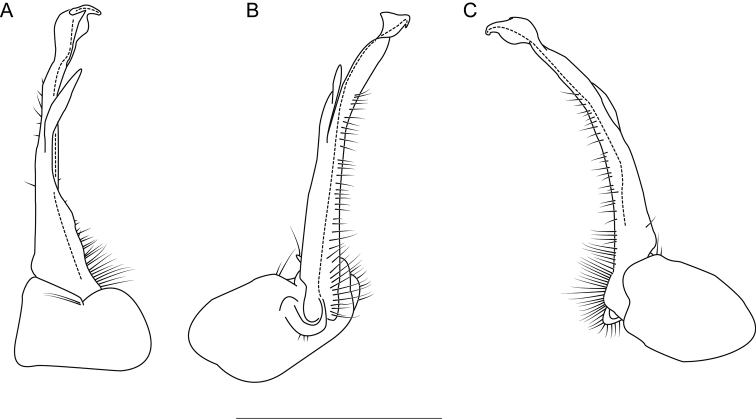
Left gonopod of *Nannarialithographa* sp. nov., male holotype (MPE03430, Rutherford County, North Carolina) **A** anterior view **B** medial view **C** posterior view Scale bar: 1 mm.

Vernacular name: The Stonewriter Twisted-Claw Millipede Figs 22 , 23

##### Material examined.

**Type material: *Holotype***: United States – **North Carolina** • ♂; Rutherford County, Chimney Rock State Park, gully on Four Seasons Trail; 35.4315°N, -82.2435°W, ±8m; elev. 457 m; 21 Oct. 2017; D. A. Hennen, J. C. Means leg.; VTEC, MPE03430. ***Paratypes***: United States – **North Carolina** • 1 ♀; same collection data as for holotype; VTEC, MPE03296 • 1 ♂; Buncombe County, 3 miles [SE] Fairview on US 74, 0.1 mile W Henderson County line; 35.4892°N, -82.3605°W, ±2000m; 28 May 1983; R. M. Shelley, Staton leg.; NCSM, NAN0495. **Non type material**: United States – **North Carolina** • 1 ♂; Rutherford County, along 1306, just E jct. 1314, 3.9 N jct. 64–74, 4 mi. E Chimney Rock; 35.4599°N, -82.187°W; 6 June 1978; R. M. Shelley, W. B. J. leg.; NCSM, NAN0521 • 4 ♀♀; Rutherford County, Chimney Rock State Park, gully on Four Seasons Trail; 35.4315°N, -82.2435°W, ± 8m; elev. 457 m; 21 October 2017; D. A. Hennen, J. C. Means leg.; VTEC, MPE03320, MPE03321, MPE03763, MPE03764; **South Carolina** • 2 ♂♂; Greenville County, Walnut Mountain, 1 mi. N of Chestnut Springs; 35.1897°N, -82.4052°W, ± 2000m; L. Hubricht leg.; VMNH, NAN0317. Complete material examined information listed in Suppl. material 1.

##### Diagnosis.

Adults of *Nannarialithographa* sp. nov. can be separated from the geographically close and morphologically similar species *N.austricola* and *N.scutellaria* by the following characters. Acropodite anterior bend slightly twisted rather than acutely twisted or crimped as in *N.austricola* and *N.scutellaria*. Acropodite tip with medial flange laminate and lateral flange produced and hooked, together forming a tapering hood-like structure instead of the parallel-sided acropodite tips of *N.austricola* and *N.scutellaria*. Prefemoral process spear-shaped and attached about halfway up the lateral side of the acropodite, rather than sinuously tapered and attached near base of acropodite as in *N.austricola* and *N.scutellaria*.

##### Description.

Suppl. material 2. Based on holotype (♂) MPE03430 and paratype (♀) MPE03296.

**Measurements**: Taken from holotype (♂) MPE03430: BL = 26.20, CW = 3.68, IW = 2.76, ISW = 0.90, B10W = 4.88, B10H = 2.81. **Color.** Tergites with two paranotal red spots, collum outlined in red, and tergites with background black (Fig. 22). **Gonopods.** Male gonopod acropodite arc straight, with bend at midpoint (Fig. 23A–C). Acropodite anterior bend with gently undulating twist (Fig. 23C). Acropodite medial flange absent. Acropodite tip medial flange laminate, smooth (Fig. 23C). Acropodite tip lateral flange hooked and produced into a sharp point and curving dorsally (Fig. 23A, B). Acropodite tip with a hood-like appearance, distally tapering and blunt (Fig. 23A). Prefemoral process spear-shaped, arising from halfway up the lateral side of the acropodite, laminate (Fig. 23A, B). Prefemoral process straight, with slight medial curve distally (Fig. 23B). Prefemoral process tip directed medially (Fig. 23B). Setae present from base of acropodite to three-quarters distally up the full acropodite length (Fig. 23B). **Cyphopods.** Female cyphopod receptacle capsule-shaped; blunt, rounded distally.

##### Variation.

The length of the acropodite tip lateral flange and acropodite tip both vary, and the curve of the acropodite tip varies from medial to ventral.

##### Distribution.

*Nannarialithographa* sp. nov. is known from Buncombe and Rutherford counties in southwest North Carolina, and adjacent Greenville County, South Carolina (Fig. 52). It is bounded at the eastern edge of its distribution by the transition to the Piedmont physiographic region of the Carolinas. On the western edge of its distribution, it may be sympatric with *N.scutellaria*.

##### Ecological notes.

This species has been collected from rhododendron coves in mesic deciduous and ericaceous forests.

##### Etymology.

The specific name is a noun in apposition derived from the Greek *lithos*, meaning stone, and *grapho*, meaning write. The species is named in honor of Chimney Rock State Park, the type locality, where this species was collected at the last moment just as field notes were being recorded before leaving the park.

#### ﻿ Nannaria lutra sp. nov.

Taxon classificationAnimaliaPolydesmidaXystodesmidae

1B3699BC-E59E-57F1-93EF-5A4C64A0FAAE

http://zoobank.org/E7E48D0E-44E4-46ED-B7AA-ED4D5C5C9186

**Figure 24.**
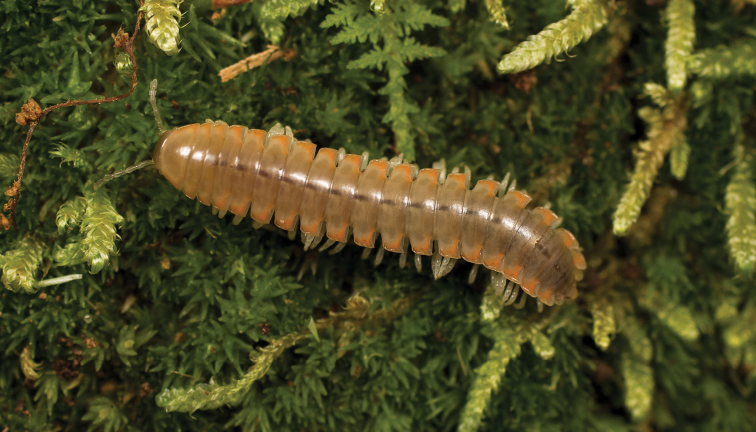
*Nannarialutra* sp. nov., in situ, male holotype (MPE02147) from Rockbridge County, Virginia.

**Figure 25.**
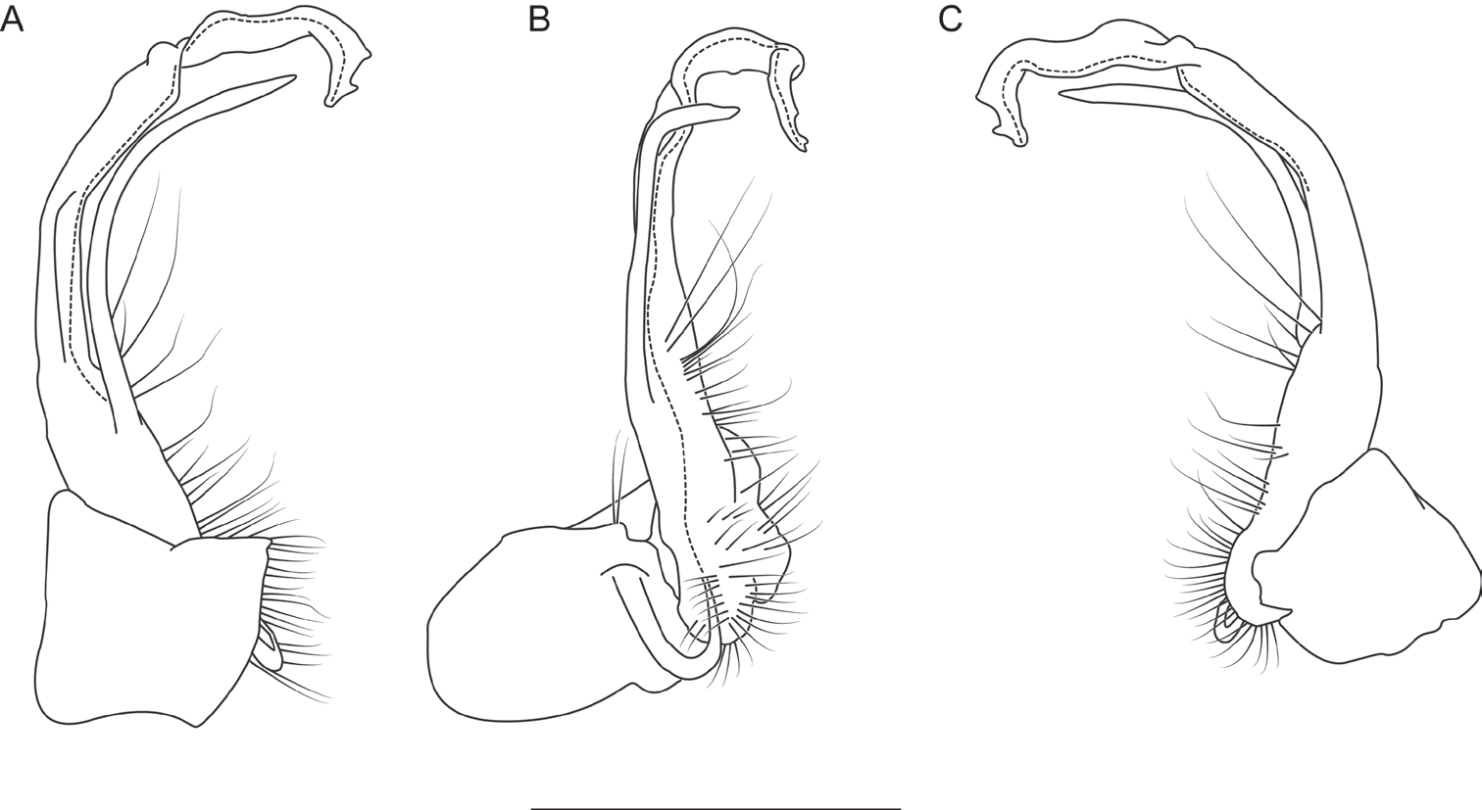
Left gonopod of *Nannarialutra* sp. nov., male holotype (MPE02147, Rockbridge County, Virginia) **A** anterior view **B** medial view **C** posterior view. Scale bar: 1 mm.

Vernacular name: The Otter Twisted-Claw Millipede Figs 24 , 25

##### Material examined.

**Type material: *Holotype***: United States – **Virginia** • ♂; Rockbridge County, Glasgow: Jefferson National Forest, James River Face Wilderness, Balcony Falls Trailhead, at end of SR 782 off SR 759; 37.6185°N, -79.4709°W, ±9m; elev. 250 m; 18 August 2016; D. A. Hennen, J. C. Means, V. Wong leg.; riparian area near trail in sandy soil, moist oak, maple litter, forest with pawpaw, sourwood; VTEC, MPE02147. ***Paratypes***: United States – **Virginia** • 1 ♂ and 1 ♀; same collection data as for holotype; VTEC, MPE02232, MPE02233 • 1 ♂; Bedford County, Flat Top Mountain, Peaks of Otter, near mile marker 82.8, Blue Ridge Parkway; 37.4690°N, -79.5801°W, ±500m; 27 October 1986; J. C. Mitchell leg.; VMNH, NAN0388. **Non type material**: United States – **Virginia** • 2 specimens; Bedford County, Blue Ridge Parkway, Floyd Field; 37.4937°N, -79.5493°W, ± 1000m; 13 May 1990; J. C. Mitchell leg.; VMNH, NAN0377 • 2 specimens; Bedford County, Peaks of Otter, Blue Ridge Parkway maintenance area; 37.4512°N, -79.5991°W, ± 3000m; 21 June 1990; J. C. Mitchell leg.; VMNH, NAN0381 • 8 specimens; Bedford County, Peaks of Otter, at base of Flat Top; 37.4545°N, -79.5995°W, ± 2000m; 19 May 1967; R. L. Hoffman, et al. leg.; VMNH, NAN0383 • 1 specimen; Bedford County, Peaks of Otter; 37.4512°N, -79.5991°W, ± 3000m; elev. 852 m; 14 to 15 October 1962; R. L. Hoffman leg.; VMNH, NAN0385 • 4 specimens; Bedford County, Peaks of Otter; 37.4512°N, -79.5991°W, ± 3000m; 15 October 1955; R. B., R. L. Hoffman leg.; VMNH, NAN0386 • 2 specimens; Bedford County, Headforemost Mtn Overlook, B. R. P.; 37.4815°N, -79.5633°W, ± 500m; 18 September 1989; C. A. Pague, K. A. Buhlmann leg.; VMNH, NAN0387 • 7 specimens; Bedford County, MP 81, Blue Ridge Pkw.; 37.4929°N, -79.5522°W, ± 500m; elev. 975 m; 20 October 1956; R. L. Hoffman, Highton leg.; VMNH, NAN0391 • 1 specimen; Bedford County, Road to radar station, Apple Orchard Mountain; 37.5176°N, -79.509°W, ± 500m; elev. 1219 m; 15 June 1997; R. L. Hoffman leg.; VMNH, NAN0393 • 6 ♂♂; Bedford County, Blue Ridge Parkway, MM 88, Side of road in gully; 37.4581°N, -79.6295°W, ± 6m; elev. 741 m; J. C. Means leg.; VTEC, MPE00156, MPE00158, MPE00159, MPE00160, MPE00161, MPE00162 • 1 ♂; Bedford County, at base of Sharp Top Mtn. trail behind Nature Center; 37.4407°N, -79.6033°W; 18 June 2016; P. L. Shorter, N. Rodgers leg.; VTEC, MPE01933 • 5 specimens; Botetourt County, “Sugarland” on SW side of Apple Orchard Mtn.; 37.5127°N, -79.5294°W, ± 1000m; 27 May 1962; R. L. Hoffman leg.; VMNH, NAN0363 • 2 specimens; Botetourt County, MP 92, Blue Ridge Parkway; 37.4768°N, -79.6895°W, ± 1000m; 20 October 1956; M. Highton leg.; VMNH, NAN0374 • 5 specimens; Botetourt County, Jefferson National Forest, Wildcat Mountain; 37.5495°N, -79.5367°W, ± 2000m; 29 October 1989; J. C. Mitchell leg.; VMNH, NAN0375 • 1 specimen; Botetourt County, “Sugarland” on W side of Apple Orchard Mtn.; 37.5127°N, -79.5294°W, ± 1000m; 1 June 1962; R. L. Hoffman leg.; VMNH, NAN0376 • 1 specimen; Botetourt County, west side Apple Orchard Mtn.; 37.5127°N, -79.5294°W, ± 1000m; elev. 1036 m; 11 April 1976; R. L. Hoffman leg.; VMNH, NAN0378 • 2 specimens; Botetourt County, N side of Bearwallow Gap, 3 mi S of Buchanan on Rt. 43; 37.4841°N, -79.6684°W, ± 1000m; 27 May 1962; R. L. Hoffman leg.; VMNH, NAN0379 • 2 specimens; Botetourt County, west side Apple Orchard Mtn near crest, along AT, berleseate; 37.5179°N, -79.5143°W, ± 1000m; 16 May 1998; R. L. Hoffman leg.; VMNH, NAN0380 • 2 specimens; Botetourt County, west slope Apple Orchard Mountain, off USFS 812; 37.5171°N, -79.527°W, ± 1000m; 13 May 1990; J. C. Mitchell leg.; VMNH, NAN0382 • 1 specimen; Botetourt County, North Creek, ca. 1–3 mi. E of Arcadia; 37.5417°N, -79.5847°W, ± 1000m; 13 October 1973; R. L. Hoffman leg.; VMNH, NAN0384 • 7 specimens; Botetourt County, Apple Orchard Mtn.; 37.5324°N, -79.512°W, ± 2000m; elev. 975 m; 14 October 1962; Bio. Club leg.; VMNH, NAN0389 • 1 ♀; Botetourt County, west side of Apple Orchard Mtn., east of Arcadia; 37.5171°N, -79.527°W, ± 2000m; 22 July 1990; J. C. Mitchell leg.; VMNH, NAN0390 • 1 ♀; Botetourt County, Jefferson National Forest, nr Parkers Gap, off FS 812, Apple Orchard Mtn; 37.5351°N, -79.5208°W, ± 1000m; 8 November 1990; J. C. Mitchell leg.; VMNH, NAN0392 • 13 specimens; Botetourt County, Apple Orchard Mtn., ca 6 mi. E of Buchanan; 37.5171°N, -79.527°W, ± 2000m; elev. 914 m; 3 September 1973; R. L. Hoffman leg.; VMNH, NAN0394 • 1 ♂; Rockbridge County, Glasglow: Jefferson National Forest, James River Face Wilderness, Balcony Falls Trailhead, at end of CR 782 off 759; 37.6185°N, -79.4709°W, ± 9m; elev. 250 m; 18 August 2016; J. C. Means, D. A. Hennen, V. Wong leg.; VTEC, MPE03667. Complete material examined information listed in Suppl. material 1.

##### Diagnosis.

Adults of *Nannarialutra* sp. nov. can be separated from the geographically close and morphologically similar species *N.cymontana* sp. nov. and *N.ericacea* by the following characters. Acropodite arc gently curving, rather than straight with an abrupt bend at tip and triangular projection as in *N.ericacea*. Acropodite with a median dome-like projection near medial flange, which is absent in *N.cymontana* sp. nov.

##### Description.

Suppl. material 2. Based on holotype (♂) MPE02147 and paratype (♀) MPE02233.

**Measurements**: Taken from holotype (♂) MPE02147: BL = 25.60, CW = 3.80, IW = 2.76, ISW = 0.87, B10W = 4.75, B10H = 2.69. **Color.** Tergites with two paranotal orange spots, collum outlined in orange, and tergites with background chestnut brown (Fig. 24). **Gonopods.** Male gonopod acropodite arc gradually curving (Fig. 25A–C). Acropodite undivided, and with an undulating twist at anterior bend (Fig. 25A). Acropodite medial flange slightly lobed, with a median dome-like projection dorsally (Fig. 25C). Acropodite tip medial flange absent. Acropodite tip lateral flange bilobed, the more distal lobe slightly smaller (Fig. 25A). Acropodite tip directed caudally (Fig. 25B). Prefemoral process sinuous; tip directed medially (Fig. 25B). **Cyphopods.** Female cyphopod receptacle triangular.

##### Variation.

Size and shape of the median dome-like projection varies and is sometimes more pointed than rounded. Acropodite tip lateral flange sometimes with lobes smaller and rounder, rather than pointed.

##### Distribution.

*Nannarialutra* sp. nov. is known from the following Virginia counties: Bedford, Botetourt, and Rockbridge (Fig. 51).

##### Ecological notes.

This species is known from mixed hardwood forests at elevations ranging from 250 m to 1219 m.

##### Etymology.

The specific name is a noun in apposition derived from the Latin word *lutra*, meaning otter. The name is an honorific for the Peaks of Otter, a trio of mountain peaks along the Blue Ridge Parkway in Bedford County and Botetourt County, Virginia.

#### ﻿ Nannaria marianae sp. nov.

Taxon classificationAnimaliaPolydesmidaXystodesmidae

309CEDA4-4A60-5E88-A586-E47E35B39E0E

http://zoobank.org/A929D071-FB10-4D61-A63F-802EE42AC414

**Figure 26.**
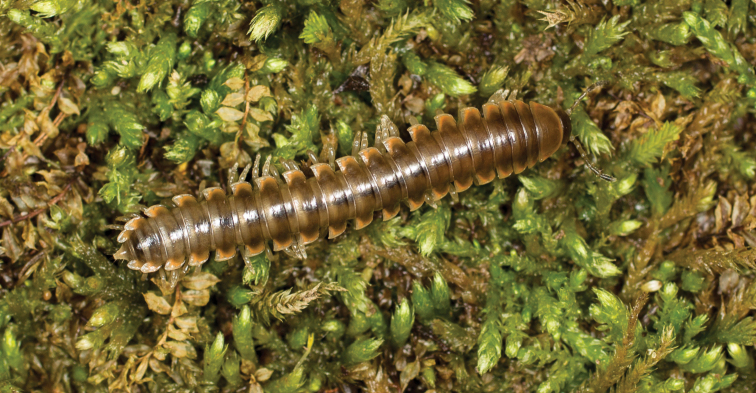
*Nannariamarianae* sp. nov., in situ, male holotype (MPE05006) from Augusta County, Virginia.

**Figure 27.**
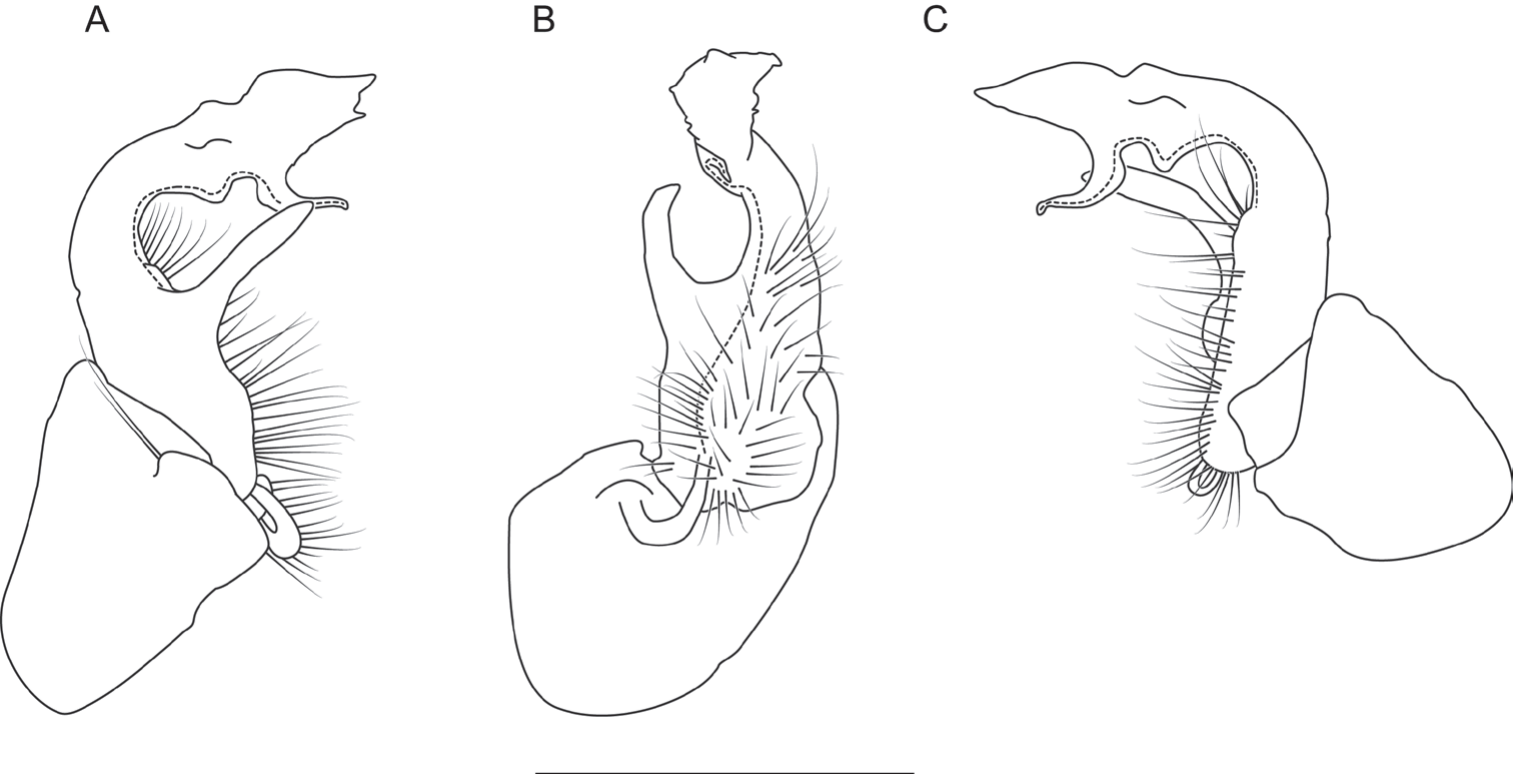
Left gonopod of *Nannariamarianae* sp. nov., male holotype (MPE05006, Augusta County, Virginia) **A** anterior view **B** medial view **C** posterior view. Scale bar: 1 mm.

Vernacular name: The Maple Flats Twisted-Claw Millipede Figs 26 , 27

##### Material examined.

**Type material: *Holotype***: United States – **Virginia** • ♂; Augusta County, Sherando: George Washington National Forest, Maple Flat Ponds area; 37.9776°N, -78.9938°W, ±7 m; elev. 480 m; 12 June 2019; D. A. Hennen, J. C. Means, M. Hellier, P. E. Marek; white oak, hickory, tuliptree forest around sinkhole ponds; VTEC, MPE05006. ***Paratypes***: United States – **Virginia** • 2 ♂♂ and 1 ♀; same collection data as for holotype; VTEC, MPE05005, VMNH, MPE04982, VTEC, MPE05010. **Non type material**: United States – **Virginia** • 3 ♂♂; Augusta County; VMNH, NAN0158 • 1 ♀; Augusta County, St. Mary’s Wilderness Cellar Mountain Trail, near trailhead; 37.9429°N, -79.1382°W, ± 2m; elev. 624 m; 24 June 2017; P. E. Marek, C. Hall leg.; VTEC, MPE02900 • 3 ♀♀; Augusta County, George Washington National Forest, St. Mary’s Wilderness, St. Mary’s River Trail; 37.9256°N, -79.1309°W, ± 6m; elev. 542 m; 24 May 2018; D. A. Hennen, J. C. Means leg.; VTEC, MPE04013, MPE04014, MPE04017 • 5 ♂♂ and 3 ♀♀; Augusta County, Sherando: George Washington National Forest, Maple Flat Ponds area; 37.9776°N, -78.9938°W, ± 7m; elev. 480 m; 12 June 2019; D. A. Hennen, J. C. Means, P. E. Marek, M. Hellier leg.; VTEC, MPE04983, MPE04986, MPE05007, MPE05008, MPE05009, MPE04984, MPE04985, MPE05010. Complete material examined information listed in Suppl. material 1.

##### Diagnosis.

Adults of *Nannariamarianae* sp. nov. can be separated from the geographically close and morphologically similar species *N.morrisoni* and *N.orycta* sp. nov. by the following characters. Immediately recognizable by its stout prefemoral process, which projects at a 45° angle from the telopodite, rather than the acicular or slightly curving prefemoral process of *N.morrisoni* and *N.orycta* sp. nov. Additionally, the acropodite tip lateral flange is laminate and very large, with jagged teeth along its edges, unlike any other *Nannaria* species.

##### Description.

Suppl. material 2. Based on holotype (♂) MPE05006 and paratype (♀) MPE05010.

**Measurements**: Taken from holotype (♂) MPE05006: BL = 29.60, CW = 3.60, IW = 2.57, ISW = 0.82, B10W = 4.35, B10H = 2.67. **Color.** Tergites with two paranotal orange spots, collum outlined in orange, and tergites with background chestnut brown (Fig. 26). **Gonopods.** Male gonopod acropodite arc straight, with acute bend at midpoint (Fig. 27A–C). Acropodite anterior bend twist lacking or almost imperceptible (Fig. 27C). Acropodite medial flange lobed (Fig. 27C). Acropodite tip medial flange absent. Acropodite tip lateral flange laminate and greatly enlarged, with various small teeth along its edges and culminating in a broad, triangular tip (Fig. 27A), extremely divergent to any species of *Nannaria*. Acropodite tip sinuous, bifurcate, directed medially (Fig. 27B). Prefemoral process stout, thick; projecting straight at a 45° angle from the acropodite (Fig. 27A), dissimilar to any other *wilsoni* species group prefemoral process. **Cyphopods.** Female cyphopod receptacle enlarged into a triangular hood, covering cyphopod valves.

##### Variation.

The number and shape of the teeth on the laminate acropodite tip lateral flange varies, but are generally quite jagged.

##### Distribution.

*Nannariamarianae* sp. nov. is known from a small area at the base of the Blue Ridge Mountains in southern Augusta County, Virginia (Fig. 51). Males have only been collected at the Maple Flats Sinkhole Ponds complex near Sherando, but genetic analysis places female *Nannaria* specimens collected in the nearby St. Mary’s Wilderness area with these males.

##### Ecological notes.

This species has been collected in a mesic white oak, hickory, and tuliptree forest and a mesic oak, hemlock, and mountain laurel forest along a stream. The Maple Flat Ponds area is known to harbor disjunct plant species (Buhlmann et al. 1999). Elevation for these sites ranges from 480 m to 624 m.

##### Etymology.

The specific name is a noun in the genitive case derived as a matronym, named in honor of Marian Winsor Hennen (née Wood), the wife of DAH, in recognition of her patience, love, and support during DAH’s doctoral studies. She grew up in Augusta County, Virginia, where all collections of this species have occurred, and has participated in her fair share of millipede collecting trips. The vernacular name for this species references the type locality, where this species was finally found after five years of searching for it at locations throughout Augusta County. Before its discovery at Maple Flats, this species was known only from VMNH specimens that stated the locality simply as “Augusta County.”

#### ﻿ Nannaria morrisoni

Taxon classificationAnimaliaPolydesmidaXystodesmidae

Hoffman, 1948

63269600-1F2D-5055-8C0A-08DD2B11CB0E

**Figure 28.**
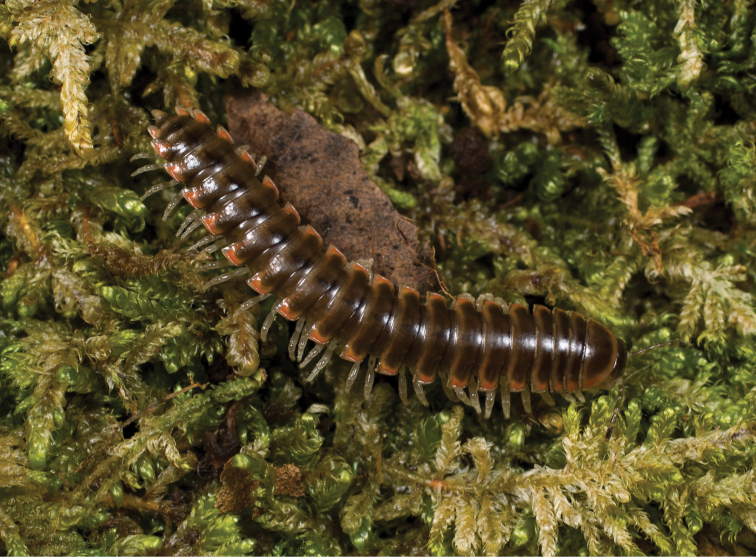
*Nannariamorrisoni*, in situ, male (MPE02595) from Greene County, Virginia.

**Figure 29.**
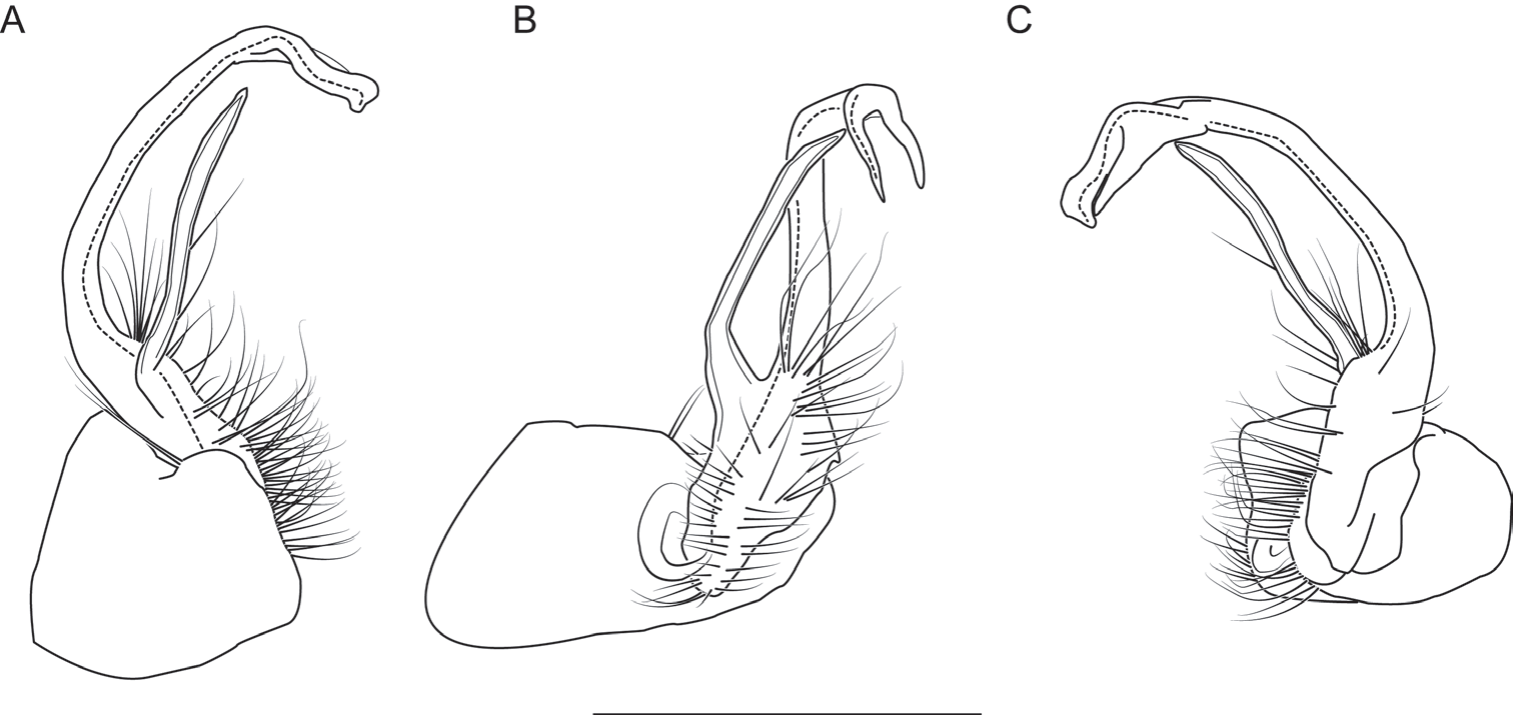
Left gonopod of *Nannariamorrisoni* (MPE02595, Greene County, Virginia) **A** anterior view **B** medial view **C** posterior view. Scale bar: 1 mm.

Vernacular name: Morrison’s Twisted-Claw Millipede Figs 28 , 29

Nannaria morrisoni Hoffman, 1948: 348, figs 3–4. Chamberlin and Hoffman 1958: 41. Hoffman 1999: 367 (351 in pdf version). Means et al. 2021a: S68, fig. 3.

##### Type material.

***Holotype***: United States – **Virginia** • ♂; Albemarle County, Saddle Hollow, about 3 miles [4.8 km] west of Crozet; 38.082°N, -78.731°W, ±1000m; elev. ca. 610 m; 28 March 1948; R. L. Hoffman leg.; “Dominant vegetation *Liriodendrontulipifera*, *Quercus* spp., and *Cerciscanadensis*” (Hoffman 1948); USNM Entomology, no. 1834; (non vidi). ***Paratypes***: United States – **Virginia** • 2 ♂♂; Page County, along Skyline Drive, 4 miles [6.4 km] north of Thornton Gap; 38.7067°N, -78.3189°W, ±1000m; April 1936; I. Fox, J. P. E. Morrison leg.; USNM Entomology, no. 1836 (non vidi).

##### Material examined.

**Non type material**: United States – **Virginia** • 2 specimens; Albemarle County, Sugar Hollow; 38.129°N, -78.7234°W, ± 2000m; 21 March 1949; R. L. Hoffman leg.; VMNH, NAN0400 • 1 ♀; Albemarle County, Carters Bridge: Peter Rausse’s land, near tasting room before construction, by stream; 37.9456°N, -78.4842°W; 19 July 2014; J. C. Means leg.; VTEC, MPE00114 • 2 ♀♀; Albemarle County, Sugar Hollow, entrance by parking lot; 38.1256°N, -78.7139°W; 28 February 2016; J. C. Means leg.; VTEC, MPE01007, MPE03715 • 1 ♂ and 4 ♀♀; Albemarle County, Sugar Hollow, North of Charlottesville Reservoir; 38.1474°N, -78.7443°W, ± 9m; elev. 487 m; 22 June 2016; J. C. Means, D. A. Hennen leg.; VTEC, MPE02107, MPE01885, MPE02015, MPE02016, MPE02149 • 2 ♂♂ and 3 ♀♀; Albemarle County, Shenandoah NP, nr JCT CR-611 & Appalachian Tr., Jarman Gap, near spring; 38.0966°N, -78.7779°W; elev. 709 m; 6 June 2005; P. E. Marek, C. Lin leg.; VTEC, SPC000495, SPC000497, SPC000496, SPC000498, SPC000499 • 13 specimens; Amherst County, South side Tar Jacket Ridge, FS 1167; 37.761°N, -79.1844°W, ± 2000m; 5 June 1998; VMNH survey leg.; VMNH, NAN0395 • 4 specimens; same collection data as for preceding; 15 August to 13 November 1999; VMNH, NAN0397 • 8 specimens; same collection data as for preceding; 6 May 1998; VMNH, NAN0398 • 23 specimens; same collection data as for preceding; 4 August 1998; VMNH, NAN0410 • 8 specimens; Amherst County, DF site on Tar Jacket Ridge, head of Piney River; 37.7707°N, -79.1749°W, ± 2000m; 9 July 1998; Jon Schilling leg.; VMNH, NAN0401 • 1 specimen; Amherst County, Pitfall on Tar Jacket Ridge, off FS 1167; 37.761°N, -79.1844°W, ± 2000m; 7 November to 18 December 1997; VMNH survey leg.; VMNH, NAN0404 • 21 specimens; Amherst County, East slope of Tar Jacket Ridge, ca. 5 mi NE of Oronoco; 37.7712°N, -79.184°W, ± 2000m; 14 May to 23 June 1999; VMNH survey leg.; VMNH, NAN0405 • 1 specimen; Amherst County, DF site on Tar Jacket Ridge, off FS 1167; 37.761°N, -79.1844°W, ± 2000m; 21 October 1997; VMNH survey leg.; VMNH, NAN0406 • 2 ♂♂ and 1 ♀; Amherst County, gully along Mt. Pleasant hiking trail; 37.7547°N, -79.185°W; elev. 1020 m; 31 December 2016; J. C. Means leg.; VTEC, MPE02491, MPE02830, MPE02829 • 1 specimen; Greene County, Hightop Mtn, 2 mi S Swift Run Gap; 38.3376°N, -78.5523°W, ± 2000m; 17 October 1990; C. A. Pague leg.; VMNH, NAN0399 • 1 ♂; Greene County, Shenandoah National Park: Appalachian Trail off South River Falls Trail; 38.385°N, -78.5147°W; elev. 896 m; 29 April 2017; J. C. Means leg.; VTEC, MPE02591 • 3 ♂♂ and 2 ♀♀; Greene County, Shenandoah National Park: tributary above South River Falls; 38.3802°N, -78.5036°W; elev. 679 m; 29 April 2017; J. C. Means leg.; VTEC, MPE02595, MPE02607, MPE02608, MPE02596, MPE02606 • 1 ♂; Greene County, Shenandoah National Park: base of South River Falls; 38.3791°N, -78.4997°W; elev. 607 m; 29 April 2017; J. C. Means leg.; VTEC, MPE02605 • 1 ♀; Greene County, Shenandoah National Park: Fire road intersecting South River Falls Trail; 38.382°N, -78.5097°W; elev. 811 m; 29 April 2017; J. C. Means leg.; VTEC, MPE03669 • 2 ♂♂ and 1♀; Madison County, Luray; 38.6649°N, -78.4593°W; July 1966; C. Ewing leg.; NCSM, NAN0489 • 1 specimen; Madison County, Shenandoah National Park, Little Stony Man Trail; 38.6118°N, -78.3616°W, ± 2000m; 27 May 1990; C. A. Pague leg.; VMNH, NAN0396 • 2 ♀♀; Nelson County, Humpback Mountain; 37.9487°N, -78.8986°W, ± 2000m; 14 October 1948; R. L. Hoffman leg.; VMNH, NAN0409 • 2 ♂♂; Nelson County, Appalachian Trail crossing of 56, up hill over Tye River; 37.8388°N, -79.0213°W; elev. 339 m; 17 September 2016; J. C. Means leg.; VTEC, MPE02115, MPE02116 • 1 ♀; Nelson County, VA-826, 0.8 rd km S of US-56; 37.8425°N, -79.1165°W, ± 2m; elev. 878 m; 24 June 2017; P. E. Marek, C. Hall leg.; VTEC, MPE02872 • 3 specimens; Page County, Panorama, Skyline Drive; 38.6592°N, -78.3213°W, ± 2000m; 21 July 1938; E. M. Loomis, H. F. Loomis leg.; VMNH, NAN0403 • 1 specimen; Rockbridge County, W side of Rocky Mtn. on BRP; 37.7963°N, -79.1988°W, ± 2000m; 24 August 1949; R. L. Hoffman leg.; VMNH, NAN0407. Complete material examined information listed in Suppl. material 1.

##### Diagnosis.

Adults of *Nannariamorrisoni* can be separated from the geographically close and morphologically similar species *N.shenandoa* and *N.orycta* sp. nov. by the following characters. Acropodite tip medial flange triangular, directed medially rather than laminate with an acuminate tip and directed caudally, as in *N.orycta* sp. nov. Prefemoral process acicular and needle-like, not curving laterally as in *N.shenandoa*.

##### Description.

Suppl. material 2. Based on (♂) (MPE02595) and ♀ (MPE02596).

**Measurements**: Taken from (♂) specimen MPE02595: BL = 28.88, CW = 3.54, IW = 2.54, ISW = 0.75, B10W = 4.30, B10H = 2.80. **Color.** Tergites with two paranotal pink to orange spots, collum outlined in pink to orange, and tergites with background chestnut brown to black (Fig. 28). **Gonopods.** Male gonopod acropodite arc a gradual curve (Fig. 29A–C). Acropodite medial flange absent, acropodite tip medial flange extended into a large, lobed, triangular projecting process, giving the tip of the acropodite a bifurcate appearance (Fig. 29B). Acropodite tip lateral flange absent. Tips of acropodite directed medially, with a slightly ventral angle (Fig. 29A). Mesal tip of acropodite carrying the seminal groove with slight dorsal expansion. Tip of medial flange ending in a sharp point (Fig. 29C). Prefemoral process acicular, needle-like, with tip directed cephalically (Fig. 29A). **Cyphopods.** Female cyphopod receptacle much enlarged, laminate.

##### Variation.

No appreciable variation was noticed between specimens.

##### Distribution.

Only known from Virginia, *Nannariamorrisoni* occurs in the following counties: Albemarle, Amherst, Bland, Greene, Madison, Nelson, Page, and Rockbridge (Fig. 51). Its distribution in these counties is almost exclusively restricted to the spine of the Blue Ridge Mountains, northeast of the James River.

##### Ecological notes.

Specimen label data indicates *Nannariamorrisoni* is collected in deciduous forests of oak, tuliptree, beech, and maple, rather than in areas with more rhododendron and other ericaceous plants. Many of the sites with habitat data list riparian areas as the habitat for this species. Specimens been collected from elevations as low as 339 m to as high as 1067 m.

##### Etymology.

*Nannariamorrisoni* is named after Dr. Joseph P. E. Morrison, a mollusk curator at the Smithsonian Museum of Natural History in Washington, D.C., who collected millipedes for Richard Hoffman during his own collecting trips for land snails (Hoffman 1948).

#### ﻿ Nannaria nessa sp. nov.

Taxon classificationAnimaliaPolydesmidaXystodesmidae

5927E059-A038-5E90-8285-510A7F4EC9CD

http://zoobank.org/111DCE2E-629C-418E-B114-E76348830B7B

**Figure 30.**
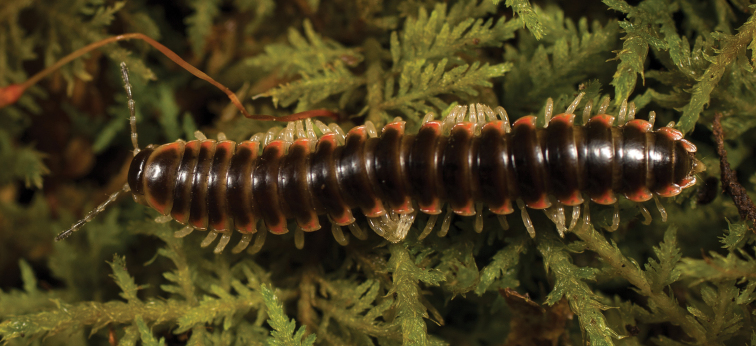
*Nannarianessa* sp. nov., in situ, male holotype (MPE01465) from Macon County, North Carolina.

**Figure 31.**
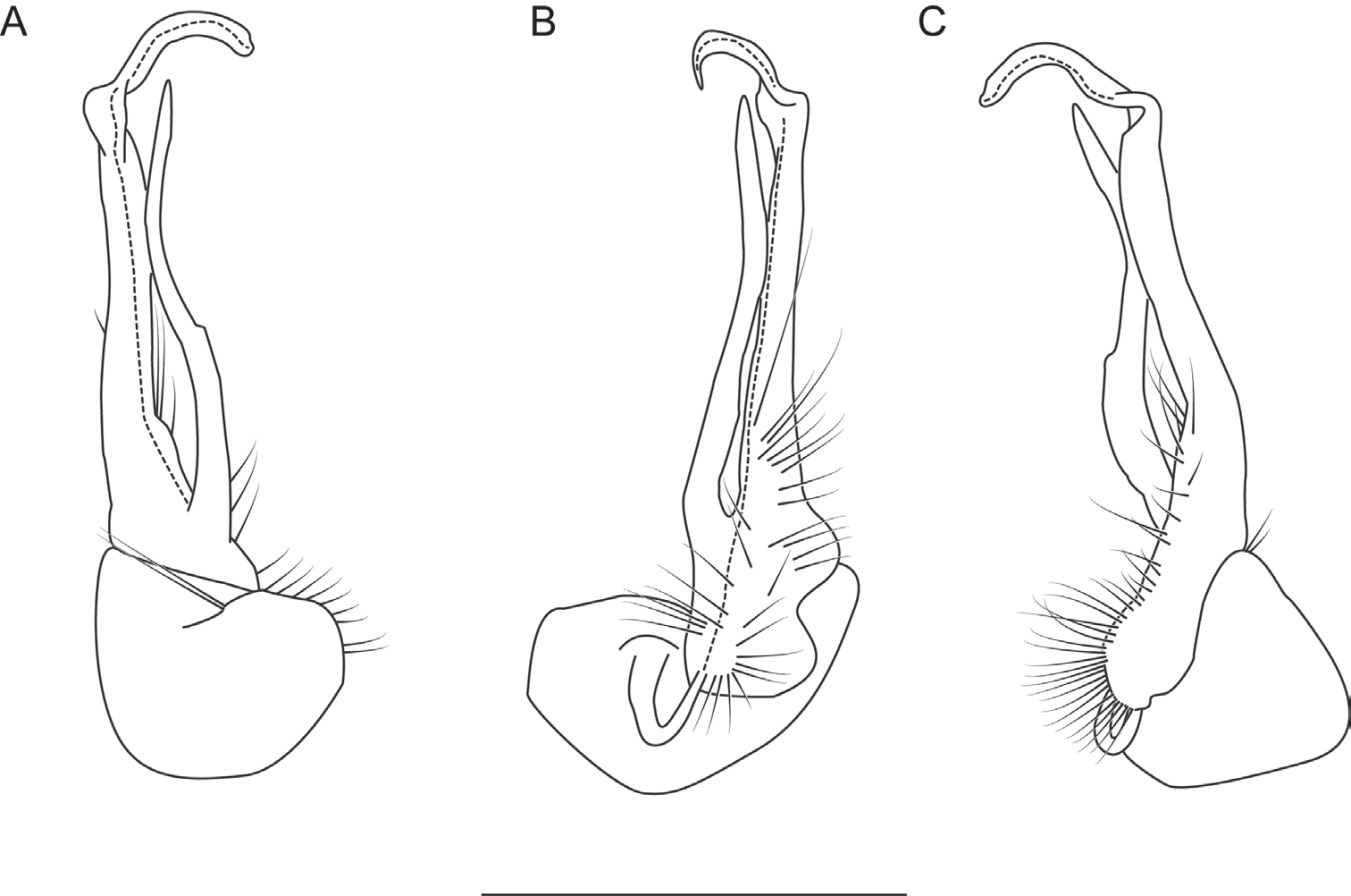
Left gonopod of *Nannarianessa* sp. nov., male holotype (MPE01465, Macon County, North Carolina) **A** anterior view **B** medial view **C** posterior view. Scale bar: 1 mm.

Vernacular name: The Duck Twisted-Claw Millipede Figs 30 , 31

Nannaria “Puc Puggy”: Means et al. 2021a: fig. 3.

##### Material examined.

**Type material: *Holotype***: United States – **North Carolina** • ♂; Macon County, 8.2 km WSW of Highlands on NC-106, Nantahala National Forest, near start of Puc Puggy Trail; 35.0288°N, -83.2823°W, ±7m; elev. 1124 m; 7 June 2016; D. A. Hennen, J. C. Means leg.; on hillside along path, moist oak, tuliptree, rhododendron forest, litter of medium depth; VTEC, MPE01465. ***Paratype***: United States – **North Carolina** • 1 ♀; same collection data as for holotype; VTEC, MPE01500. **Non type material**: United States – **Georgia** • 3 ♂♂; Rabun County, Rabun Bald, about 1 mile south trailhead at Beegum Gap; 34.9694°N, -83.3°W, ± 1500m; 26 October 2019; C. W. Harden leg.; VTEC, MPE05015, MPE05016, MPE05017; **North Carolina** • 1 ♀; Macon County, 8.2 km WSW of Highlands on NC-106, Nantahala National Forest, near start of Puc Puggy Trail; 35.0288°N, -83.2823°W, ± 7m; elev. 1124 m; 7 June 2016; D. A. Hennen, J. C. Means leg.; VTEC, MPE01501. Complete material examined information listed in Suppl. material 1.

##### Diagnosis.

Adults of *Nannarianessa* sp. nov. can be separated from the geographically close and morphologically similar species *N.austricola* and *N.spalax* sp. nov. by the following characters. Acropodite medial flange lobed, rather than laminate and with a short, triangular tip medial flange as in *N.spalax* sp. nov. Acropodite tip medial flange absent, not lobed as in *N.austricola*. Prefemoral process only slightly curving and with a notch on its medial side near midpoint, not curving laterally as in *N.austricola* or thick and with a jagged medial margin as in *N.spalax* sp. nov.

##### Description.

Suppl. material 2. Based on holotype (♂) MPE01465 and paratype (♀) MPE01500.

**Measurements**: Taken from holotype (♂) MPE01465: BL = 22.10, CW = 3.28, IW = 2.73, ISW = 0.99, B10W = 3.95, B10H = 2.75. **Color.** Tergites with two paranotal red spots, collum outlined in red, and tergites with background black (Fig. 30). **Gonopods.** Male gonopod acropodite arc straight, with abrupt bend at tip (Fig. 31A–C). Acropodite with acute twist at anterior bend, giving the acropodite a pinched appearance (Fig. 31C). Acropodite medial flange lobed (Fig. 31C). Acropodite tip medial flange and lateral flange both absent. Acropodite tip blunt, directed caudally (Fig. 31B). Prefemoral process sinuously tapered, with basal jutting shelf medially (Fig. 31A, C). Prefemoral process tip directed cephalically (Fig. 31A). **Cyphopods.** Female cyphopod receptacle triangular.

##### Variation.

No noticeable variation observed.

##### Distribution.

*Nannarianessa* sp. nov. is only known from Macon County, North Carolina and adjacent Rabun County, Georgia (Fig. 52).

##### Ecological notes.

This species is found in deciduous and rhododendron cove habitats, within leaf litter and under rocks.

##### Etymology.

The specific name is a noun in apposition derived from the Greek *nessa*, meaning duck. It is named after Duck Mountain, a mountain near the type locality.

#### ﻿ Nannaria orycta sp. nov.

Taxon classificationAnimaliaPolydesmidaXystodesmidae

B7FB8D7C-EC20-580C-B599-37D25299627A

http://zoobank.org/37BBA209-55EE-4453-B995-2CABA512AC75

**Figure 32.**
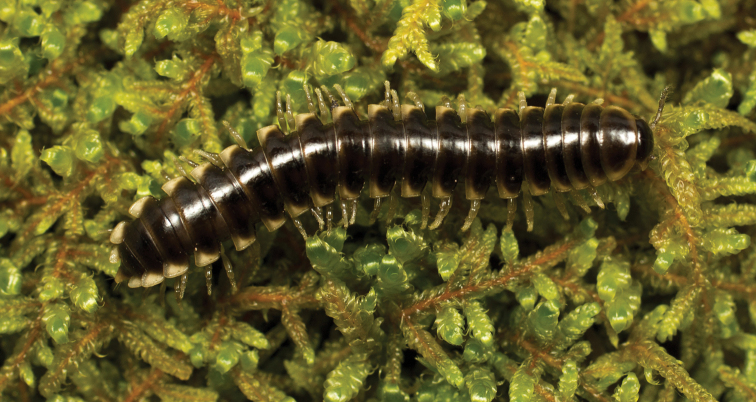
*Nannariaorycta* sp. nov., in situ, male holotype (MPE03810) from Rockbridge County, Virginia.

**Figure 33.**
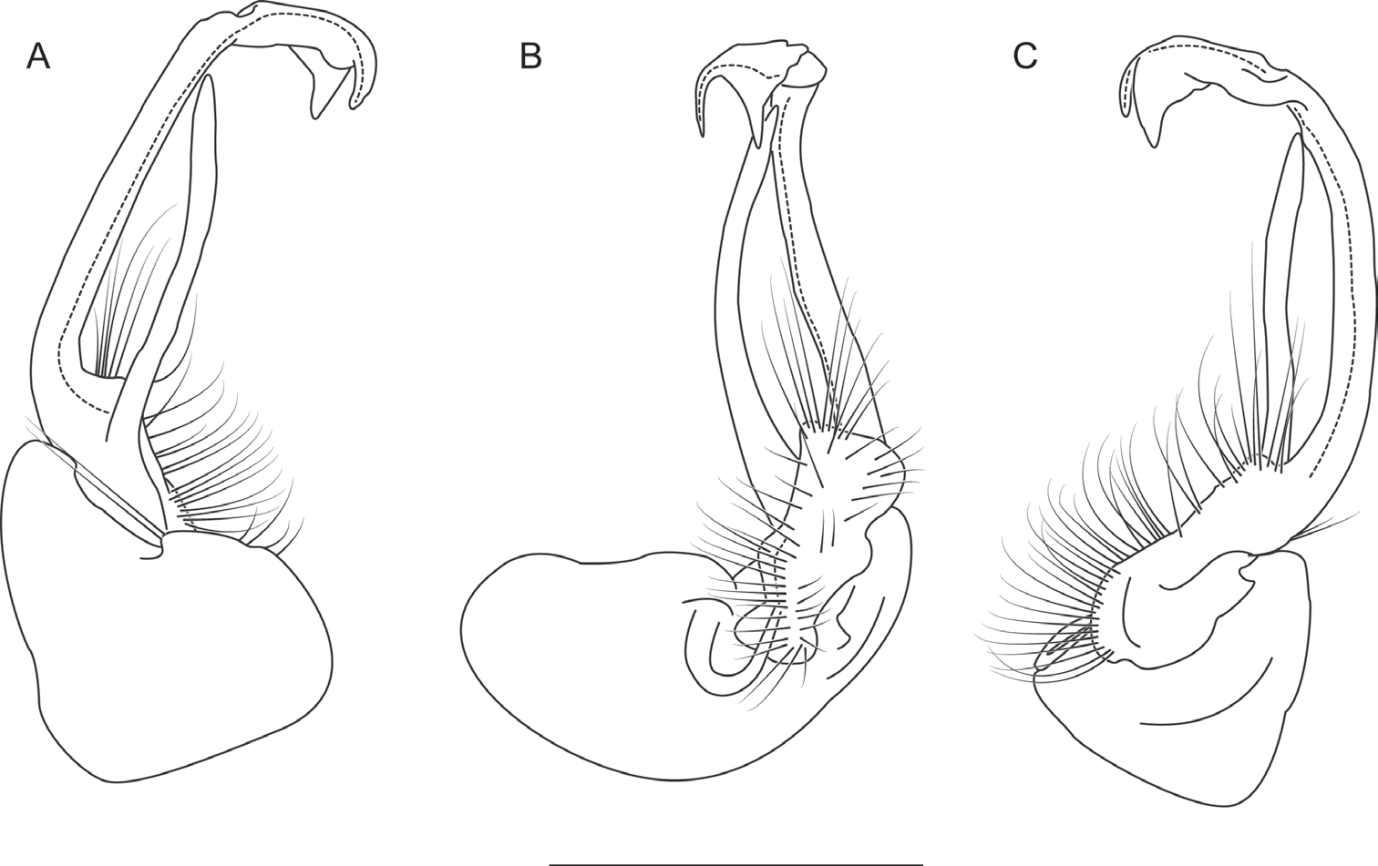
Left gonopod of *Nannariaorycta* sp. nov., male holotype (MPE03810, Rockbridge County, Virginia) **A** anterior view **B** medial view **C** posterior view. Scale bar: 1 mm.

Vernacular name: The Digging Twisted-Claw Millipede Figs 32 , 33

##### Material examined.

**Type material: *Holotype***: United States – **Virginia** • ♂; Rockbridge County, 3.2 air km NW of Collierstown: Lake Robertson Recreation Area, Mountain Trail, hillside near Hawks Creek stream crossing; 37.8065°N, -79.6152°W, ±10m; elev. 457 m; 20 Feb 2018; D. A. Hennen, J. C. Means leg.; tuliptree, maple, sycamore woods, moist, dark soil; VTEC, MPE03810. ***Paratypes***: United States – **Virginia** • 1 ♂ and 1 ♀; same collection data as for holotype; VTEC, MPE03819, MPE03823. **Non type material**: United States – **Virginia** • 1 specimen; Amherst County, Staton Creek Gorge, George Washington Natl. For. NE of Buena Vista; 37.7717°N, -79.2437°W, ± 2000m; 1 August 1974; H. Levi, L. Levi, F. Levi leg.; VMNH, NAN0063 • 1 specimen; Augusta County, E of Steele’s Tavern, W slope of Blue Ridge 1 mile below summit; 37.9261°N, -79.1151°W, ± 5000m; 24 September 1949; J. P. E. Morrison leg.; VMNH, NAN0061 • 2 specimens; Nelson County, along Appalachian Trail to pitfall site on The Priest, ca. 4 mi SE of Montebello; 37.8254°N, -79.0788°W, ± 3000m; elev. 914 m; 20 September 1992; R. L. Hoffman leg.; VMNH, NAN0058 • 2 specimens; Nelson County, Wintergreen Mountain Resort; 37.9349°N, -78.935°W, ± 2000m; 22 September 1996; R. L. Hoffman leg.; VMNH, NAN0059 • 4 specimens; Nelson County, The Priest, 4 mi. SE Montebello DF site; 37.8202°N, -79.065°W, ± 3000m; elev. 1189 m; 20 September to 18 October 1992; VMNH survey leg.; VMNH, NAN0060 • 2 specimens; same collection data as for preceding; 28 August to 21 September 1991; VMNH, NAN0062 • 3 ♂♂; Rockbridge County, 3.2 air km NW of Collierstown: Lake Robertson Recreation Area, Mountain Trail, hillside near Hawks Creek stream crossing; 37.8065°N, -79.6152°W, ± 10m; elev. 457 m; 20 February 2018; D. A. Hennen, J. C. Means leg.; VTEC, MPE03820, MPE03821, MPE03822 • 11 ♂♂ and 9 ♀♀; Rockingham County, Lexington: Washington and Lee University campus, hillside beside community gardens; 37.7956°N, -79.4427°W, ± 6m; elev. 330 m; 14 November 2017; J. C. Means, D. A. Hennen leg.; VTEC, MPE03487, MPE03488, MPE03489, MPE03502, MPE03505, MPE03507, MPE03508, MPE03510, MPE03512, MPE03514, MPE03519, MPE03496, MPE03497, MPE03501, MPE03504, MPE03506, MPE03509, MPE03511, MPE03513, MPE03515. Complete material examined information listed in Suppl. material 1.

##### Diagnosis.

Adults of *Nannariaorycta* sp. nov. can be separated from the geographically close and morphologically similar species *N.filicata* sp. nov., *N.marianae* sp. nov., and *N.morrisoni* by the following characters. Acropodite anterior bend acutely twisted and folded, not absent as in *N.marianae* sp. nov. and *N.morrisoni* or only slightly twisted as in *N.filicata* sp. nov. Acropodite tip directed caudally, not medially as in *N.marianae* sp. nov. and *N.morrisoni*. Acropodite with basomedial process, rather than absent as in *N.filicata* sp. nov.

##### Description.

Suppl. material 2. Based on holotype (♂) MPE03810 and paratype (♀) MPE03823.

**Measurements**: Taken from holotype (♂) MPE03810: BL = 28.30, CW = 4.00, IW = 2.88, ISW = 0.90, B10W = 4.90, B10H = 3.00. **Color.** Tergites with two paranotal white spots, collum outlined in white, and tergites with background black (Fig. 32). **Gonopods.** Male gonopod acropodite arc straight, with abrupt bend distally (Fig. 33A–C). Acropodite anterior bend with acute twist, appearing folded over (Fig. 33C). Acropodite medial flange lobed and concave dorsally (Fig. 33C). Acropodite tip medial flange greatly enlarged and laminate, directed caudally with a pointed tip (Fig. 33C). Acropodite tip lateral flange absent. Acropodite tip directed caudally (Fig. 33B). Acropodite with quadrate basomedial process. Prefemoral process gradually curving, with tip directed laterally (Fig. 33A). **Cyphopods.** Female cyphopod receptacle triangular.

##### Variation.

The color of the paranotal spots in *Nannariaorycta* sp. nov. may be either white or red. The male gonopod twist at the anterior bend ranges from acutely twisted to strongly crimped, with the distal portion of the acropodite almost recurved 270°.

##### Distribution.

This species is known from the following Virginia counties, where it mainly inhabits the Blue Ridge Mountains: Amherst, Augusta, Nelson, Rockbridge, and Rockingham (Fig. 51).

##### Ecological notes.

Label data indicates this species inhabits mesic mixed deciduous woods, at elevations ranging from 330 m to 1189 m.

##### Etymology.

The specific name is a feminine adjective derived from the Greek word *oryktes*, meaning “one who digs,” and alludes to the fossorial habits of *Nannaria*.

#### ﻿ Nannaria paraptoma sp. nov.

Taxon classificationAnimaliaPolydesmidaXystodesmidae

27C0A594-CF6D-51CE-8411-160087C35BD0

http://zoobank.org/D3166398-0636-4513-B5AA-2144169EED8E

**Figure 34.**
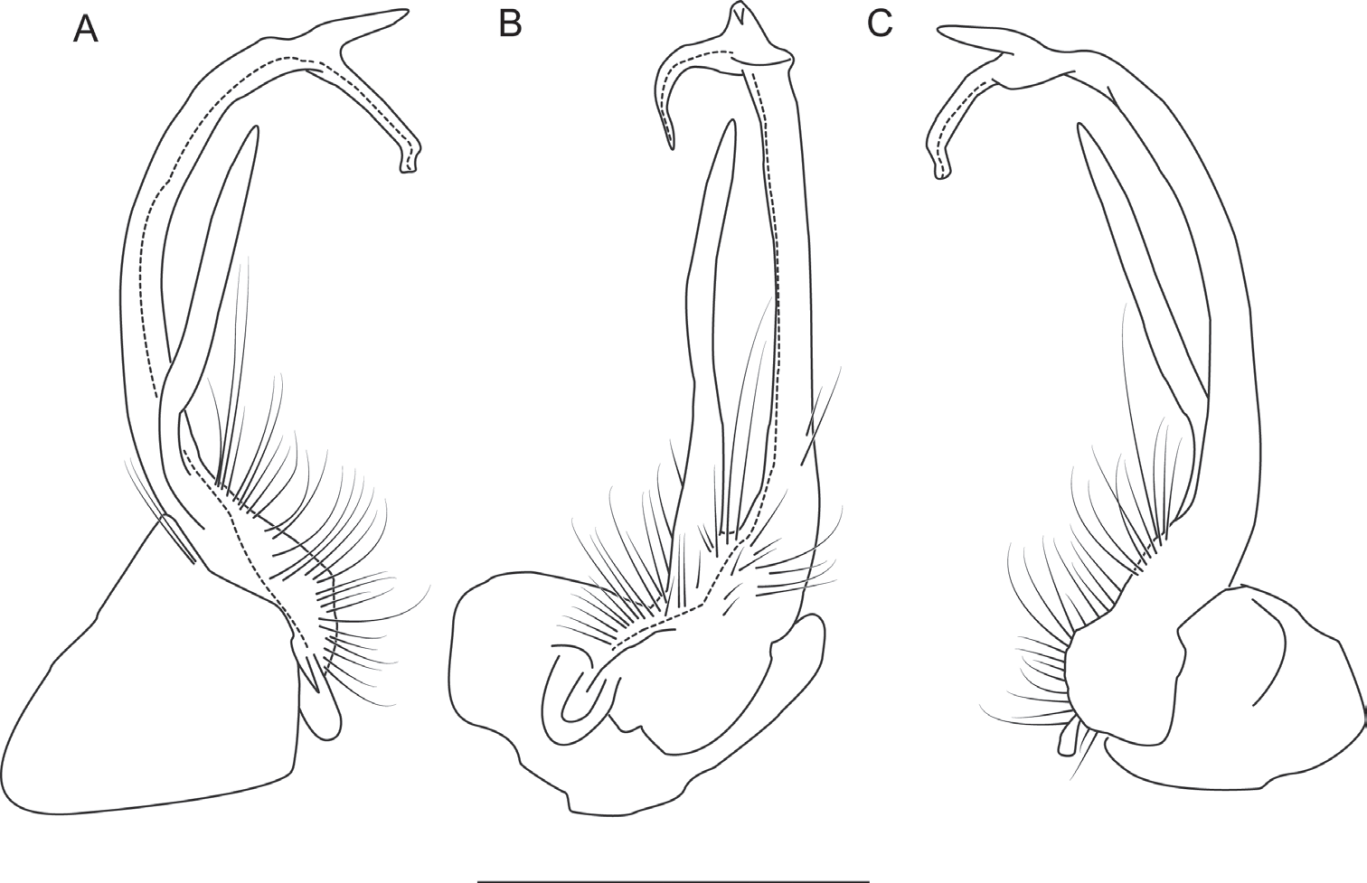
Left gonopod of *Nannariaparaptoma* sp. nov., male holotype (NAN0650, Bath County, Virginia) **A** anterior view **B** medial view **C** posterior view. Scale bar: 1 mm.

Vernacular name: The Tumbling Twisted-Claw Millipede Fig. 34

##### Material examined.

***Holotype***: United States – **Virginia** • ♂; Bath County, Sunrise, George Washington National Forest, Long Spring Run, above Little Back Creek; 38.2213°N, -79.8387°W, ± 2000m; pitfall trap; 24 July 1992; J. Pagels, D. Kobuszewski leg.; VMNH, NAN0650. ***Paratype***: United States – **Virginia** • ♂; same collection data as holotype; 11 September 1992; D. Kobuszewski leg.; VMNH, NAN0655.

**Non type material**: United States – **West Virginia** • 2 ♂♂; Pocahontas County, Watoga State Park, 3 mi. SE of Seebert; 38.1129°N, -80.1217°W, ± 5000m; 17 October 1971; R. L. Hoffman, L. S. Knight leg.; VMNH, NAN0292. Complete material examined information listed in Suppl. material 1.

##### Diagnosis.

Adults of *Nannariaparaptoma* sp. nov. can be separated from the geographically close and morphologically similar species *N.ericacea*, *N.shenandoa*, and *N.spiralis* sp. nov. by the following characters. Acropodite arc gradually curving, rather than straight with an abrupt bend distally as in *N.ericacea*. Acropodite medial flange with a large dorsal triangular projection, rather than having a small triangular tip medial flange as in *N.ericacea*. Acropodite medial flange lobed, rather than laminate and shield-shaped as in *N.spiralis*. Acropodite undivided distally, not bifurcate as in *N.shenandoa*.

##### Description.

Suppl. material 2. Based on holotype (♂) NAN0650. No females have been collected, thus the female morphology is unknown.

**Measurements**: Taken from holotype (♂) NAN0650: BL = 27.80, CW = 4.31, IW = 2.95, ISW = 1.02, B10W = 5.06, B10H = 2.69. **Color.** Color in life unknown, but specimen with typical bimaculate pattern, faded in alcohol. **Gonopods.** Male gonopod acropodite arc gradually curving (Fig. 34A–C). Acropodite with gently undulating twist at apical bend (Fig. 34C). Acropodite medial flange lobed, small, with a large, dorsal triangular projection (Fig. 34C). Acropodite tip medial and lateral flanges absent. Acropodite tip entire, with a small preapical bump, and directed caudally (Fig. 34A). Prefemoral process acicular, mostly straight but with a slight curve basally (Fig. 34A). Prefemoral process tip directed cephalically (Fig. 34A).

##### Variation.

No noticeable variation observed.

##### Distribution.

*Nannariaparaptoma* sp. nov. is only known from a small area of Pocahontas County, West Virginia and Bath County, Virginia (Fig. 51).

##### Ecological notes.

The label data from the collected specimens do not provide any notes on the habitat, but on a collecting trip to the type locality to search for live individuals by DAH and PEM, the habitat was found to be a mesic oak, hickory, tuliptree, and hemlock forest dissected by streams down in a holler, with an elevation of 840 m.

##### Etymology.

The specific name is a noun in apposition and derives from the Greek word *paraptoma*, meaning false step or slip. This is in reference to the holotype having been collected from a pitfall trap.

#### ﻿ Nannaria rhododendra sp. nov.

Taxon classificationAnimaliaPolydesmidaXystodesmidae

AEA682E9-62E4-5629-90D4-C5BC6D498A11

http://zoobank.org/2415F648-DC81-4793-AECD-384BBD17A3D5

**Figure 35.**
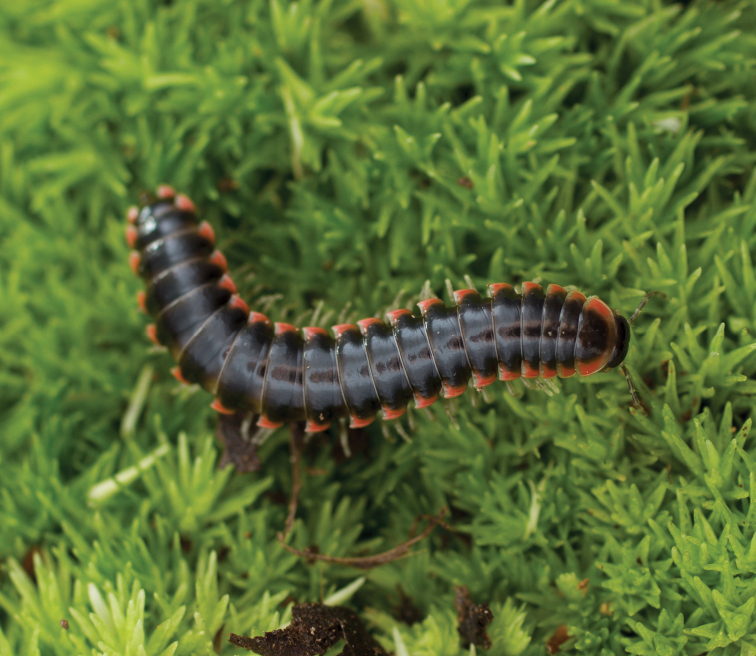
*Nannariarhododendra* sp. nov., in situ, female paratype (MPE04399) from Towns County, Georgia.

**Figure 36.**
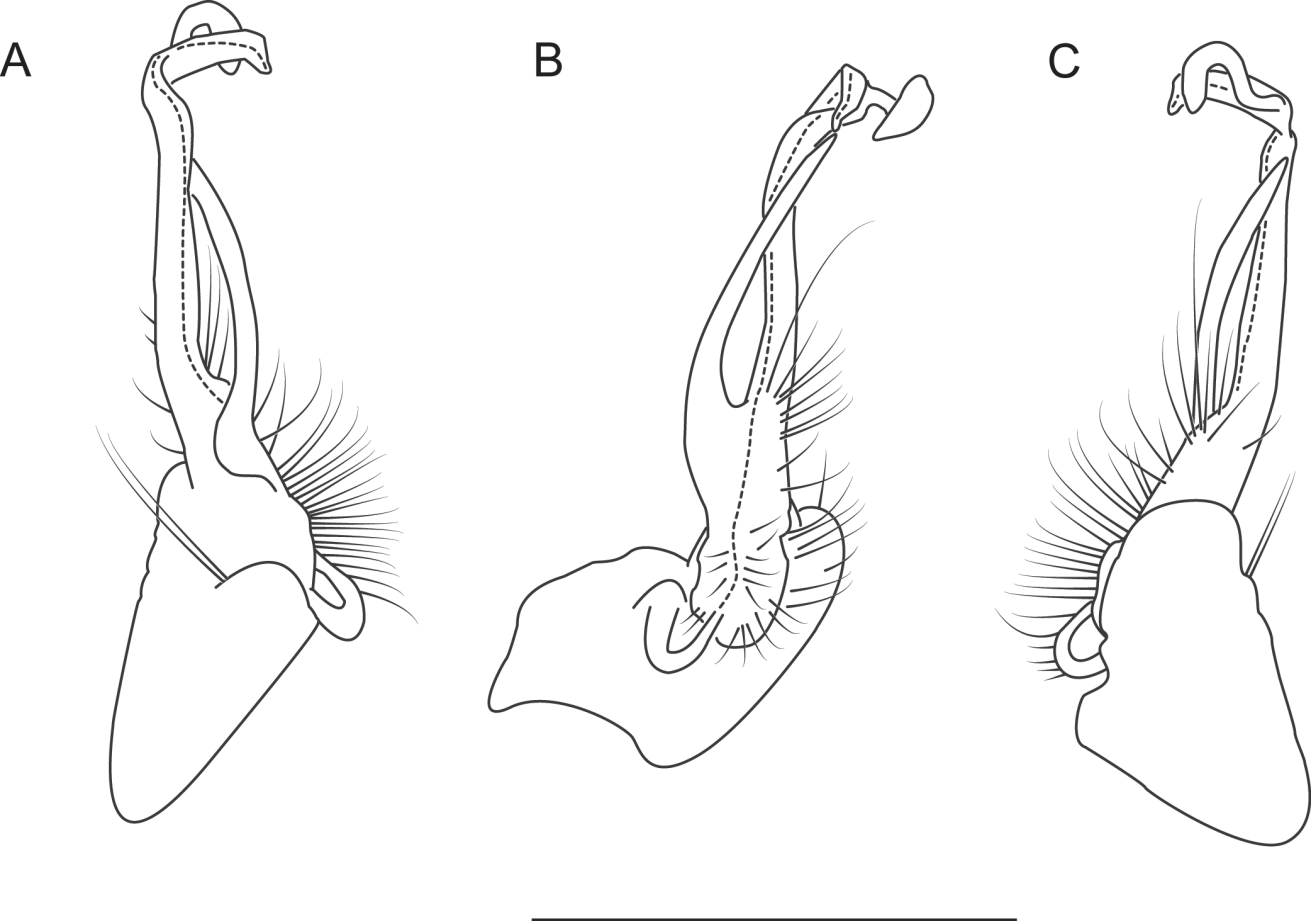
Left gonopod of *Nannariarhododendra* sp. nov., male holotype (MPE04401, Towns County, Georgia) **A** anterior view **B** medial view **C** posterior view. Scale bar: 1 mm.

Vernacular name: The Rhododendron Twisted-Claw Millipede Figs 35 , 36

##### Material examined.

**Type material: *Holotype***: United States – **Georgia** • ♂; Towns County, Hiawassee: Long Ridge Campground, Harris Cove, off Burch Branch Road; 34.9256°N, -83.7776°W, ±6m; elev. 647 m; 15 October 2018; D. A. Hennen leg.; moist litter and soil in rhododendron, mountain laurel, tuliptree, beech forest near stream; VTEC, MPE04401. ***Paratypes***: United States – **Georgia** • 1 ♂ and 1 ♀; same collection data as for holotype; VTEC, MPE04796, MPE04399 • 1 ♂; Towns County, Harris Cove, 3 miles SW of Hiawassee; 34.9218°N, -83.7770°W, ±2000m; 1 November 1981; R. L. Hoffman leg.; VMNH, NAN0210. **Non type material**: United States – **Georgia** • 3 ♂♂; Lumpkin County, Chattahoochee NF, Woody Gap Rec Area; 34.675°N, -84.0015°W, ± 9m; elev. 977 m; 14 October 2018; D. A. Hennen leg.; VTEC, MPE04342, MPE04343, MPE04344 • 2 ♂♂; Lumpkin County, Chattahoochee NF, DeSoto Falls Rec Area, Lower Falls Trail; 34.7078°N, -83.915°W, ± 8m; elev. 633 m; 14 October 2018; D. A. Hennen leg.; VTEC, MPE04363, MPE04364 • 2 ♂♂; Towns County, Harris Cove, 2 mi. SW of Hiawassee; 34.9218°N, -83.777°W, ± 2000m; 14 October 1978; R. L. Hoffman leg.; VMNH, NAN0211 • 2 ♀♀; Towns County, Hiawassee: Long Ridge Campground, Harris Cove, off Burch Branch Road; 34.9256°N, -83.7776°W, ± 6m; elev. 647 m; 15 October 2018; D. A. Hennen leg.; VTEC, MPE04400, MPE04799 • 1 ♂ and 1 ♀; Towns County, Hiawassee: Towns County Park; 34.9688°N, -83.7733°W, ± 9m; elev. 610 m; 15 October 2018; D. A. Hennen leg.; VTEC, MPE04336, MPE04337 • 1 ♂ and 5 ♀♀; Towns County, Young Harris: Brasstown Valley Resort, Lodge Connector Trail to Miller Trek; 34.9538°N, -83.8359°W, ± 7m; elev. 670 m; 15 October 2018; D. A. Hennen leg.; VTEC, MPE04348, MPE04349, MPE04350, MPE04351, MPE04352, MPE04353. Complete material examined information listed in Suppl. material 1.

##### Diagnosis.

Adults of *Nannariarhododendra* sp. nov. can be separated from the geographically close and morphologically similar species *N.amicalola* sp. nov. and *N.antarctica* sp. nov. by the following characters. Acropodite tip medial flange produced into a recurved lobe, not laminate and giving the acropodite the appearance of being bifurcate, as in *N.antarctica* sp. nov. Prefemoral process sinuously tapered and curving, rather than erect and laminate as in *N.amicalola* sp. nov.

##### Description.

Suppl. material 2. Based on holotype (♂) MPE04401 and paratype (♀) MPE04399.

**Measurements**: Taken from holotype (♂) MPE04401: BL = 20.20, CW = 3.10, IW = 2.38, ISW = 0.78, B10W = 3.80, B10H = 2.63. **Color.** Tergites with two paranotal red spots, collum outlined in red, and tergites with background black (Fig. 35). **Gonopods.** Male gonopod acropodite arc straight, with abrupt bend distally (Fig. 36A–C). Acropodite with a smoothly undulating twist at acropodite bend (Fig. 36A). Acropodite medial flange slightly lobed (Fig. 36B, C). Acropodite tip medial flange produced into a recurved lobe with a rounded tip and shaped like a rabbit ear (Fig. 36C). Acropodite tip lateral flange slightly lobed before acropodite (Fig. 36A). Acropodite tip blunt, tapering; directed caudally (Fig. 36B). Prefemoral process sinuously tapered, thicker at base (Fig. 36A). Prefemoral process curving laterally (Fig. 36A). Prefemoral process crossing acropodite ventrolaterally; tip directed laterally (Fig. 36B). **Cyphopods.** Female cyphopod receptacle in the shape of a finger-like projection, curving over cyphopod valves and tapering distally.

##### Variation.

No noticeable variation observed.

##### Distribution.

*Nannariarhododendra* sp. nov. is known from two counties in northern Georgia: Towns and Lumpkin (Fig. 52).

##### Ecological notes.

This species has been collected both in dry and moist situations, ranging from upland oak-hickory forest to riparian cove forests of rhododendron and hemlock. Elevations at collecting localities are between 610 and 977 m.

##### Etymology.

The specific name is a feminine adjective derived from the Greek words *rhodon*, meaning rose, and *dendron*, meaning tree. The species is named for the ericaceous plant genus *Rhododendron* L., which commonly grows in areas where *Nannaria* is found and was abundant at the type locality of *N.rhododendra*.

#### ﻿ Nannaria scutellaria

Taxon classificationAnimaliaPolydesmidaXystodesmidae

Causey, 1942

37063BA9-A7DE-5C3F-9715-975E51686847

**Figure 37.**
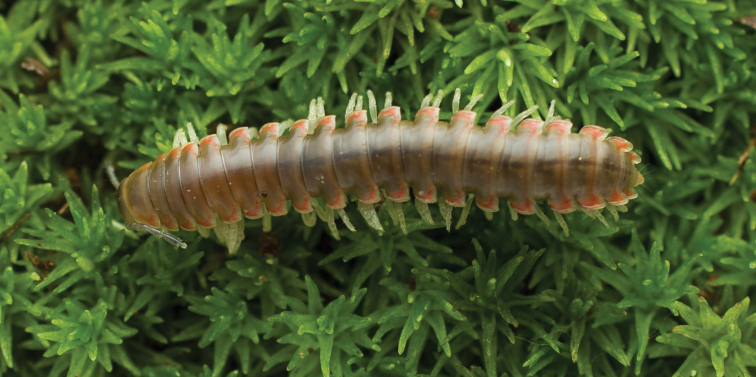
*Nannariascutellaria*, in situ, male (MPE04346) from Transylvania County, North Carolina.

**Figure 38.**
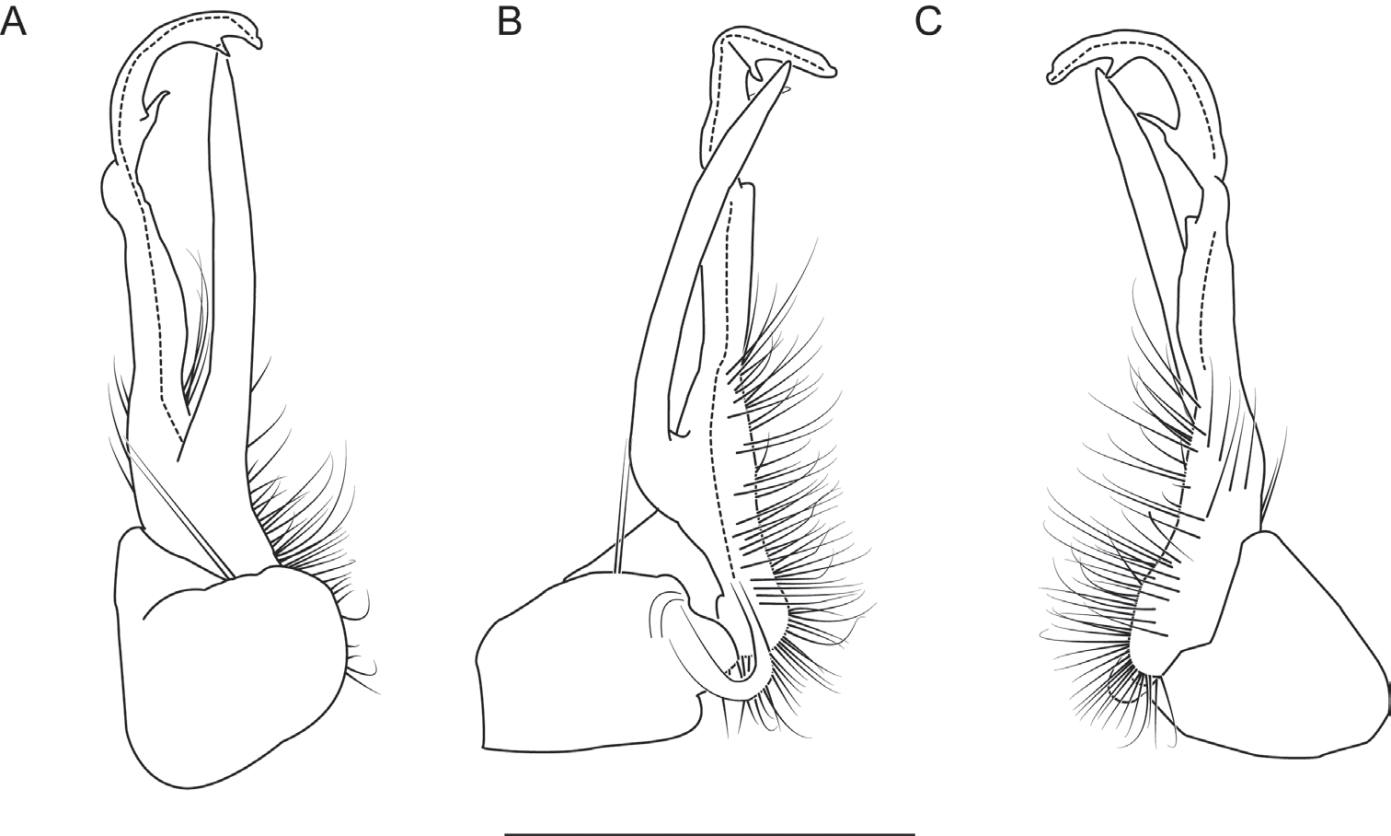
Left gonopod of *Nannariascutellaria* (MPE01474, Sevier County, Tennessee) **A** anterior view **B** medial view **C** posterior view. Scale bar: 1 mm.

Vernacular name: The Smoky Mountains Twisted-Claw Millipede Figs 37 , 38

Nannaria scutellaria Causey, 1942: 168, figs 6, 7. Causey 1950: 198, figs 1–6. Chamberlin and Hoffman 1958: 41. Wray 1967: 152. Hoffman 1999: 367 (352 in pdf version). Shelley 2000: 197. Means et al. 2021a: S69, fig. 3.

##### Type material.

***Holotype***: United States – **Tennessee** • ♂; Sevier County, Great Smoky Mountains National Park, near Chimneys; 35.6369°N, -83.4883°W, ±1000 m; 21 June 1940; N. B. Causey leg.; ANSP; (non vidi). ***Paratype***: United States – **Tennessee** • 1 ♀; same collection data as for holotype; FSCA; (non vidi). The paratype was retained in Causey’s personal collection (Causey 1942), now at the FSCA, where Causey’s collection was transferred after her death.

##### Material examined.

**Non type material**: United States – **North Carolina** • 1 ♀; Clay County, Fires Creek Campground, ca 1.1 mi N Mission Dam Rd on Fires Creek Rd (Fires Creek Wildlife Area), trail due north of parking area; 35.0882°N, -83.866°W; elev. 550 m; 19 April 2003; B. E. Hendrixson leg.; VTEC, MPE03745 • 10 specimens; Haywood County, Great Smoky Mountains National Park: ATBI Plot, Cataloochee; 35.5844°N, -83.0815°W, ± 300m; 15 November 2000; I. C. Stocks leg.; GRSM • 1 specimen; same collection data as for preceding; 30 November 2000; GRSM • 2 specimens; same collection data as for preceding; 10 October 2001; GRSM • 13 specimens; same collection data as for preceding; 26 October 2001; GRSM • 13 specimens;

Transylvania County, Pisgah NF, Daniel Ridge Falls aka Jackson Falls Trail; 35.2851°N, -82.8286°W, ± 6m; elev. 818 m; 12 October 2018; D. A. Hennen leg.; VTEC, MPE04346; **Tennessee** • 2 specimens; Cocke County, Great Smoky Mountains National Park: ATBI Plot, Albright Grove; 35.7314°N, -83.2806°W, ± 300m; 14 November 2000; I. C. Stocks leg.; GRSM • 1 specimen; same collection data as for preceding; 9 May 2001; M. McCord leg.; GRSM • 1 specimen; same collection data as for preceding; 6 July 2001; GRSM • 1 specimen; same collection data as for preceding; 22 May 2001; I. C. Stocks, M. Williams leg.; GRSM • 1 ♂;

Cocke County, Cosby Picnic Area and Trail, GSMNP; 35.757°N, -83.2087°W; 18 May 1978; R. M. Shelley, W. B. J. leg.; NCSM, NAN0483 • 1 ♂; Cocke County, Brushy Mtn ATBI plot, pitfall 46; 35.8512°N, -82.9468°W, ± 5000m; 15 to 30 November 2000; Parker, et al. leg.; VMNH, NAN0371 • 1 specimen; Sevier County, Great Smoky Mtns. Natl. Park, Cades Cove; 35.58°N, -83.84°W, ± 3000m; 13 September 1953; D. H. Kistner leg.; FMNH, FMNHINS 0000 002 102 • 2 specimens; Sevier County, Great Smoky Mtns. Natl. Park, Greenbriar Cove; 35.7°N, -83.38°W, ± 2000m; elev. 610 m; 17 September 1946; H. S. Dybas leg.; FMNH, FMNHINS 0000 002 106 • 2 specimens; Sevier County, Great Smoky Mountains National Park: ATBI Plot, Goshen Prong; 35.6088°N, -83.5427°W, ± 300m; 10 November 2000; I. C. Stocks leg.; GRSM • 1 specimen; same collection data as for preceding; 28 November 2000; GRSM • 1 specimen; same collection data as for preceding; 26 February 2001; GRSM • 1 specimen; same collection data as for preceding; 2 July 2001; GRSM • 1 specimen; same collection data as for preceding; 17 September 2001; GRSM • 4 specimens; same collection data as for preceding; 22 October 2001; GRSM • 3 specimens; same collection data as for preceding; 5 November 2001; GRSM • 8 specimens; same collection data as for preceding; 5 December 2001; GRSM • 4 specimens; same collection data as for preceding; 15 October 2002; GRSM • 5 specimens; same collection data as for preceding; 21 November 2002; GRSM • 1 specimen; same collection data as for preceding; 27 April 2001; I. C. Stocks, J. Breeden leg.; GRSM • 1 specimen; same collection data as for preceding; 7 June 2001; I. C. Stocks, M. Butler leg.; GRSM • 2 specimens; same collection data as for preceding; 18 December 2001; C. R. Parker leg.; GRSM • 1 specimen; same collection data as for preceding; 10 February 2002; D. Stair leg.; GRSM • 3 specimens; same collection data as for preceding; 1 April 2002; I. C. Stocks, R. Saczawa, D. Fowler leg.; GRSM • 1 specimen; same collection data as for preceding; 11 April 2002; J. Saczawa, R. Saczawa leg.; GRSM • 1 specimen; same collection data as for preceding; 25 April 2002; GRSM • 1 specimen; same collection data as for preceding; 23 May 2002; GRSM • 1 specimen; same collection data as for preceding; 19 August 2002; GRSM • 1 specimen; same collection data as for preceding; 20 December 2002; I. C. Stocks, G. Graves leg.; GRSM • 1 specimen; Sevier County, Great Smoky Mountains National Park: ATBI Plot, Twin Creeks; 35.6841°N, -83.4994°W, ± 300m; 15 October 2001; I. C. Stocks leg.; GRSM • 2 specimens; same collection data as for preceding; 5 November 2001; GRSM • 1 specimen; same collection data as for preceding; 5 December 2001; GRSM • 1 specimen; same collection data as for preceding; 21 June 2002; B. Merritt leg.; GRSM • 1 specimen; same collection data as for preceding; 16 July 2002; GRSM • 8 specimens; Sevier County, Camping area on Greenbrier Rd.; 35.7119°N, -83.3839°W, ± 5000m; 30 August 1961; VMNH, NAN0195 • 2 ♂♂ and 1 ♀; Sevier County, Chimneys; 35.637°N, -83.4882°W, ± 500m; 28 July 1949; R. L. Hoffman leg.; VMNH, NAN0372 • 1 ♂; Sevier County, Great Smoky Mountains National Park, Chimneys Picnic Area, Cove Hardwood Trail; 35.6360°N, -83.4937°W, ±7m; elev. 877 m; 8 June 2016; D. A. Hennen, J. C. Means leg.; along trail, moist hemlock, oak, maple, tuliptree litter; VTEC, MPE01474. Complete material examined information listed in Suppl. material 1.

##### Diagnosis.

Adults of *Nannariascutellaria* can be separated from the geographically close and morphologically similar species *N.antarctica* sp. nov. and *N.austricola* by the following characters. Acropodite with a thin, triangular medial flange, rather than lobed and slightly expanded as in *N.austricola*. Acropodite tip undivided, not bifurcate as in *N.antarctica* sp. nov.

##### Description.

Suppl. material 2. Based on (♂) MPE01474 and (♀) MPE01480.

**Measurements**: Taken from (♂) specimen MPE01474: BL = 29.70, CW = 3.08, IW = 2.54, ISW = 0.81, B10W = 3.92, B10H = 2.64. **Color.** Tergites with two paranotal pink to red spots, collum outlined in pink, and tergites with background chestnut brown to black (Fig. 37). **Gonopods.** Male gonopod acropodite arc straight, with abrupt bend distally (Fig. 38A–C). Acropodite anterior bend with acute twist, a scooped-out area visible in posterior view (Fig. 38C). Acropodite medial flange a thin, tooth-like, triangular projection (Fig. 38C). Acropodite tip medial flange a medium-sized triangular process (Fig. 38C). Acropodite tip lateral flange absent. Acropodite tip undivided, curving, and directed ventrally. Prefemoral process with a sinuous taper, thicker at base and curving slightly laterally, crossing acropodite ventrolaterally (Fig. 38A). **Cyphopods.** Female cyphopod receptacle in the shape of an enlarged and elongated club.

##### Variation.

*Nannariascutellaria* has a large amount of gonopodal variation, as documented by Causey (1950b). Her study included males from localities within the Great Smoky Mountains National Park and showed variation in the shape of the acropodite medial flange and number of subterminal teeth (acropodite tip medial flange) near the tip of the acropodite, as well as the shape of the acropodite tip. Additionally, males examined for this study from across the distribution of *N.scutellaria* exhibited a large amount of variation in the processes on the distal portion of the acropodite, and this variation may indicate that *N.scutellaria* is a cryptic species complex. The observed variation included specimens that have a very long acropodite medial flange and lack an acropodite tip medial flange entirely (NAN0077, Transylvania County, North Carolina), specimens with a typical morphology as illustrated in Fig. 38, with an acropodite medial flange and acropodite tip medial flange (NAN0368, Jackson County, North Carolina), and in one case, a specimen with a laminate acropodite tip medial flange, giving the acropodite tip a leaf-like appearance (NAN0369, Swain County, North Carolina). However, without more specimens available for genetic analysis, splitting *N.scutellaria* would be ill-advised, and all of these variable forms are included within this species.

##### Distribution.

The distribution of *Nannariascutellaria* straddles the border between Tennessee and North Carolina in the southern Appalachian Mountains, and is known from the following counties: (Tennessee) Blount, Cocke, and Sevier; (North Carolina) Jackson, Haywood, Swain, and Transylvania (Fig. 52).

##### Ecological notes.

*Nannariascutellaria* has been collected mostly from moist mixed oak, maple, tuliptree, hemlock, and rhododendron forests. It has been found at elevations between 610 m and 1918 m.

##### Etymology.

Causey (1942) did not specify the precise meaning of the specific epithet, but it derives from the Latin word *scutella*, meaning a small dish or platter.

#### ﻿ Nannaria shenandoa

Taxon classificationAnimaliaPolydesmidaXystodesmidae

Hoffman, 1949

3503A0DB-4C14-520A-BA20-3A6510F73E8B

**Figure 39.**
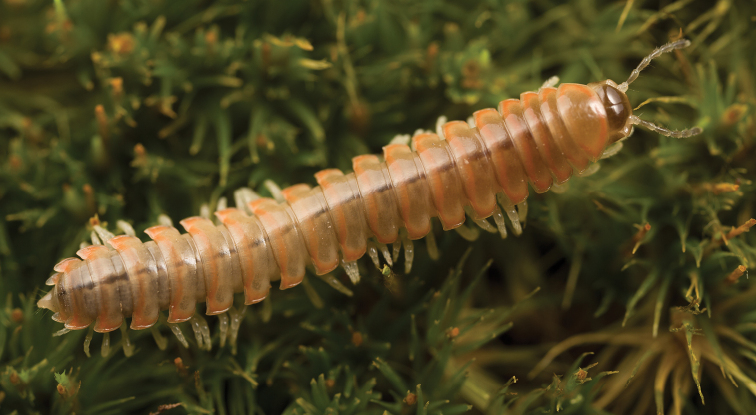
*Nannariashenandoa*, in situ, male (MPE00231) from Marion County, West Virginia.

**Figure 40.**
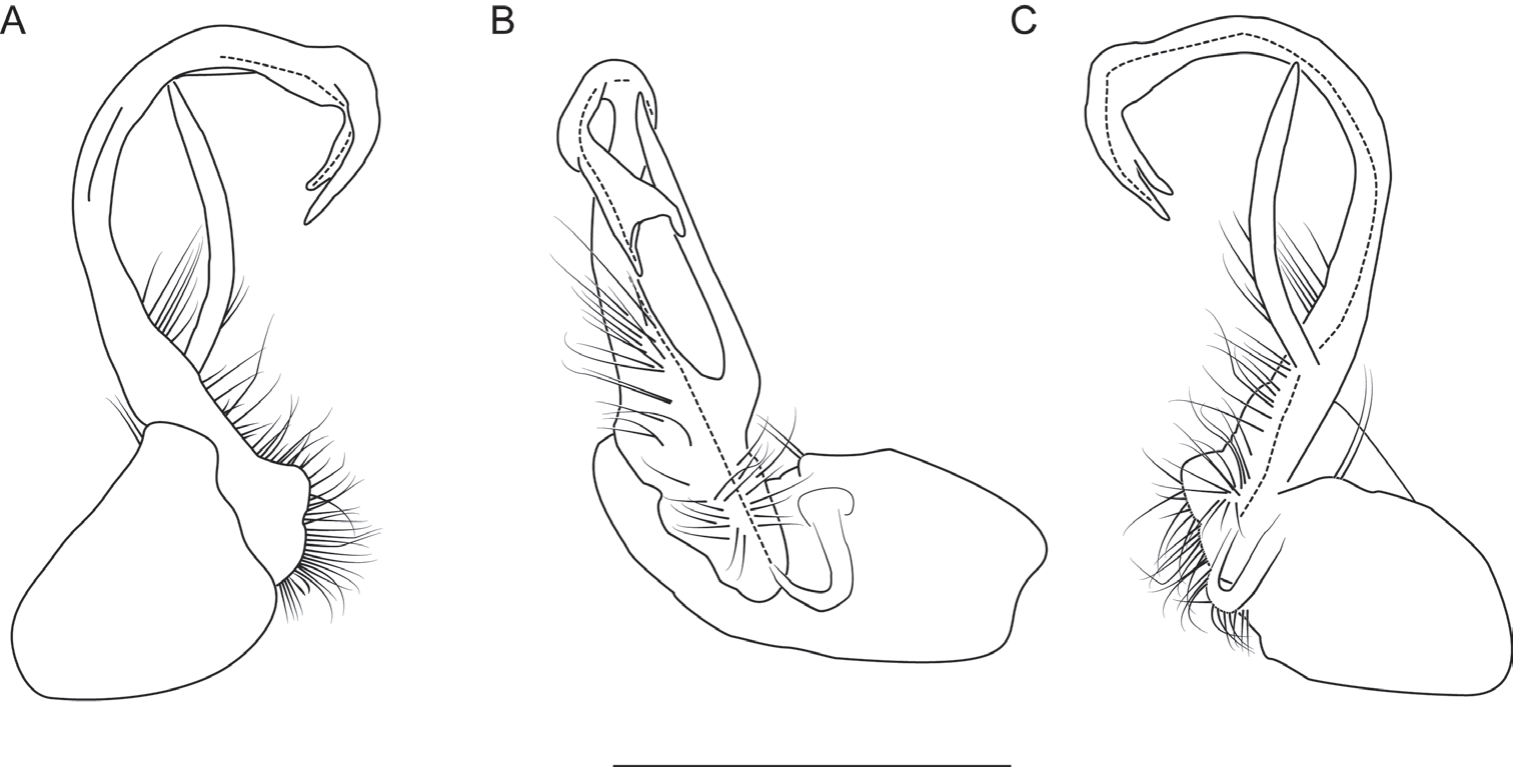
Left gonopod of *Nannariashenandoa* (MPE03994, Shenandoah County, Virginia) **A** anterior view **B** medial view **C** posterior view. Scale bar: 1 mm.

Vernacular name: The Shenandoah Twisted-Claw Millipede Figs 39 , 40

Nannaria shenandoa Hoffman, 1949a: 82, figs 1–4. Chamberlin and Hoffman 1958: 41. Hoffman 1999: 368 (352 in pdf version). Means et al. 2021a: S69. Means et al. 2021b: 9, fig. 4E.

##### Type material.

***Holotype***: United States – **Virginia** • ♂; Rockingham County, Shenandoah Mountain, about 15 miles [24.1 km] west of Harrisonburg; 38.57°N, -79.16°W, ± 2000m; elev. ca. 1067 m; 3 July 1948; R. L. Hoffman leg.; Habitat at the type locality was a “rather dry stand of *Quercus* (*Q.alba* and related species) with undergrowth mainly scrub oak and laurel (*Kalmialatifolia*)” on loose sandstone (Hoffman 1949a); USNM Entomology, no. 1848; (non vidi). ***Paratypes***: United States – **Virginia** • 1 ♂ and 1 ♀; same collection data as for holotype; USNM Entomology, no. 1848 (non vidi). • 2 ♂♂ and 2 ♀♀; same collection data as for holotype; 4 July 1948; VMNH, NAN0067 (vidi). Four paratypes from the same locality were retained in Hoffman’s personal collection (Hoffman 1949a), with catalog number RLH no. 160. The VMNH specimen lot NAN0067 is labeled “PARATYPE” and matches this description.

##### Material examined.

**Non type material**: United States – **Kentucky** • 1 ♂; Powell County, Slade: Natural Bridge State Park, Original Trail; 37.7748°N, -83.6825°W, ± 7m; elev. 325 m; 25 September 2017; D. A. Hennen, J. C. Means leg.; VTEC, MPE03104; **Virginia** • 2 specimens; Augusta County, DF site off FS 85, ca 3 mi. NE of jct. with FS 95, Shenandoah Mt.; 38.4306°N, -79.2743°W, ± 3000m; 17 June 1988; K. A. Buhlmann leg.; VMNH, NAN0064 • 1 ♂; Rockingham County, Shenandoah Mt.; 38.57°N, -79.16°W, ± 5000m; 4 April 1948; MCZ, 87616 • 1 ♂; Rockingham County, Tomahawk Mtn., ca 7 mi NNW Rawley Springs, DF site off FS 72; 38.599°N, -79.1062°W, ± 5335m; elev. 1006 m; 19 November 1988; K. A. Buhlmann leg.; VMNH, NAN0065 • 2 ♂♂; same collection data as for preceding; 17 June 1988; VMNH, NAN0066 • 6 specimens; same collection data as for preceding; 28 May 1988; VMNH, NAN0068 • 2 specimens; Rockingham County, Grottoes; 38.2576°N, -78.7664°W, ± 5000m; 20 April 1950; R. L. Hoffman leg.; VMNH, NAN0408 • 4 ♂♂; Rockingham County, Shenandoah Mountain, DF site off Va. 924, ca. 0.5 mi. E of WVA state line, jct with FS 85; 38.7879°N, -79.0418°W, ± 2000m; 17 June 1988; K. A. Buhlmann leg.; VMNH, NAN0670 • 1 specimen; Shenandoah County, George Washington National Forest near New Market off Rt. 211 on Rd 274 (Massanutten Visitors Center); 38.6531°N, -78.6033°W, ± 2000m; 26 April 1975; T. Eisner leg.; VMNH, NAN0402 • 1 ♂; Shenandoah County, Passage Creek Gorge ~ 2 mi S jct of Rte 678 and VA-55; 38.9407°N, -78.3058°W; 4 May 2018; C. W. Harden leg.; VTEC, MPE03994 **West Virginia** • 8 specimens; Greenbrier County, Droop Mountain Battlefield State Park; 38.1119°N, -80.2716°W, ± 2289m; 23 June 1968; R. L. Hoffman leg.; VMNH, NAN0216 • 1 ♂; Marion County, Palatine: Bunner’s Ridge; 39.4369°N, -79.9852°W; 10 October 2014; M. Kasson leg.; VTEC, MPE00231 • 77 specimens; Pocahontas County, Droop Mtn State Park; 38.1137°N, -80.2653°W, ± 3000m; elev. 933 m; 30 April 1972; W. A. Shear leg.; VMNH, NAN0198, NAN0199 • 10 specimens; Pocahontas County, Droop Mountain Battlefield State Park; 38.1119°N, -80.2716°W, ± 2289m; 17 October 1971; R. L. Hoffman, L. S. Knight leg.; VMNH, NAN0215 • 2 specimens; Preston County, Terra Alta; 39.4455°N, -79.5466°W, ± 1876m; 26 June 1971; W. A. Shear leg.; VMNH, NAN0069. Complete material examined information listed in Suppl. material 1.

##### Diagnosis.

Adults of *Nannariashenandoa* can be separated from the geographically close and morphologically similar species *N.morrisoni* and *N.spiralis* sp. nov. by the following characters. Acropodite bifurcate, with acropodite tip medial flange lobed and expanded into another branch, rather than acropodite entire as in *N.spiralis* sp. nov. Tips of acropodite directed caudally, rather than medially as in *N.morrisoni*. Prefemoral process curving laterally, rather than acicular as in *N.morrisoni* or sinuously tapering as in *N.spiralis* sp. nov.

##### Description.

Suppl. material 2. Based on (♂) MPE03994 and (♀) NAN0067.

**Measurements**: Taken from (♂) specimen MPE03994: BL = 27.95, CW = 3.46, IW = 2.51, ISW = 0.85, B10W = 4.25, B10H = 2.70. **Color.** Tergites with two paranotal peach-pink spots, collum outlined in peach, and tergites with background olive-brown (Fig. 39). **Gonopods.** Male gonopod acropodite arc gradually curving (Fig. 40A–C). Acropodite medial flange absent. Acropodite tip medial flange lobed, giving acropodite tip a bifurcate appearance (Fig. 40C). Acropodite tip lateral flange absent. Acropodite lacking an obvious twist at anterior bend. Prefemoral process simple, curving laterally (Fig. 40A). Prefemoral process tip crossing acropodite ventrolaterally; tip directed dorsally. **Cyphopods.** Female cyphopod receptacle enlarged into a triangular hood, covering cyphopod valves.

##### Variation.

No notable variation was observed between specimens.

##### Distribution.

The distribution of *Nannariashenandoa* extends across eastern West Virginia into northwestern Virginia and includes the following counties: (West Virginia) Marion, Preston, Pocahontas, Greenbrier; (Virginia) Augusta, Rockingham, Shenandoah (Fig. 51). Additionally, one male specimen was collected from Natural Bridge State Park in Powell County, Kentucky, which appears morphologically identical to the specimens from West Virginia and Virginia; genetically, it is located in the same group as the other *N.shenandoa* specimens, and so is included here. Further study may reveal either a larger distribution than is currently known, connecting the Kentucky population with the West Virginia population, or a cryptic species complex within *N.shenandoa*.

##### Ecological notes.

Label information for *Nannariashenandoa* notes the species has been collected in mixed deciduous forests of oak, maple, and tuliptree, along with hemlock and rhododendron forests. It ranges in elevation from 325 m to 1006 m.

##### Etymology.

This species is named after its type locality, Shenandoah Mountain in Rockingham County, Virginia.

#### ﻿ Nannaria spalax sp. nov.

Taxon classificationAnimaliaPolydesmidaXystodesmidae

83FCE413-12BD-534A-9B27-DEFF949BBD48

http://zoobank.org/12995EEE-4A07-499C-9D79-1A3BE3302848

**Figure 41.**
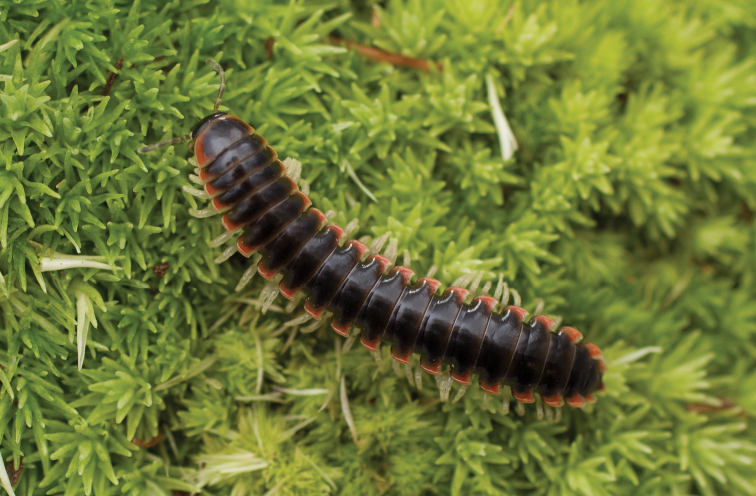
*Nannariaspalax* sp. nov., in situ, male holotype (MPE04377) from Rabun County, Georgia.

**Figure 42.**
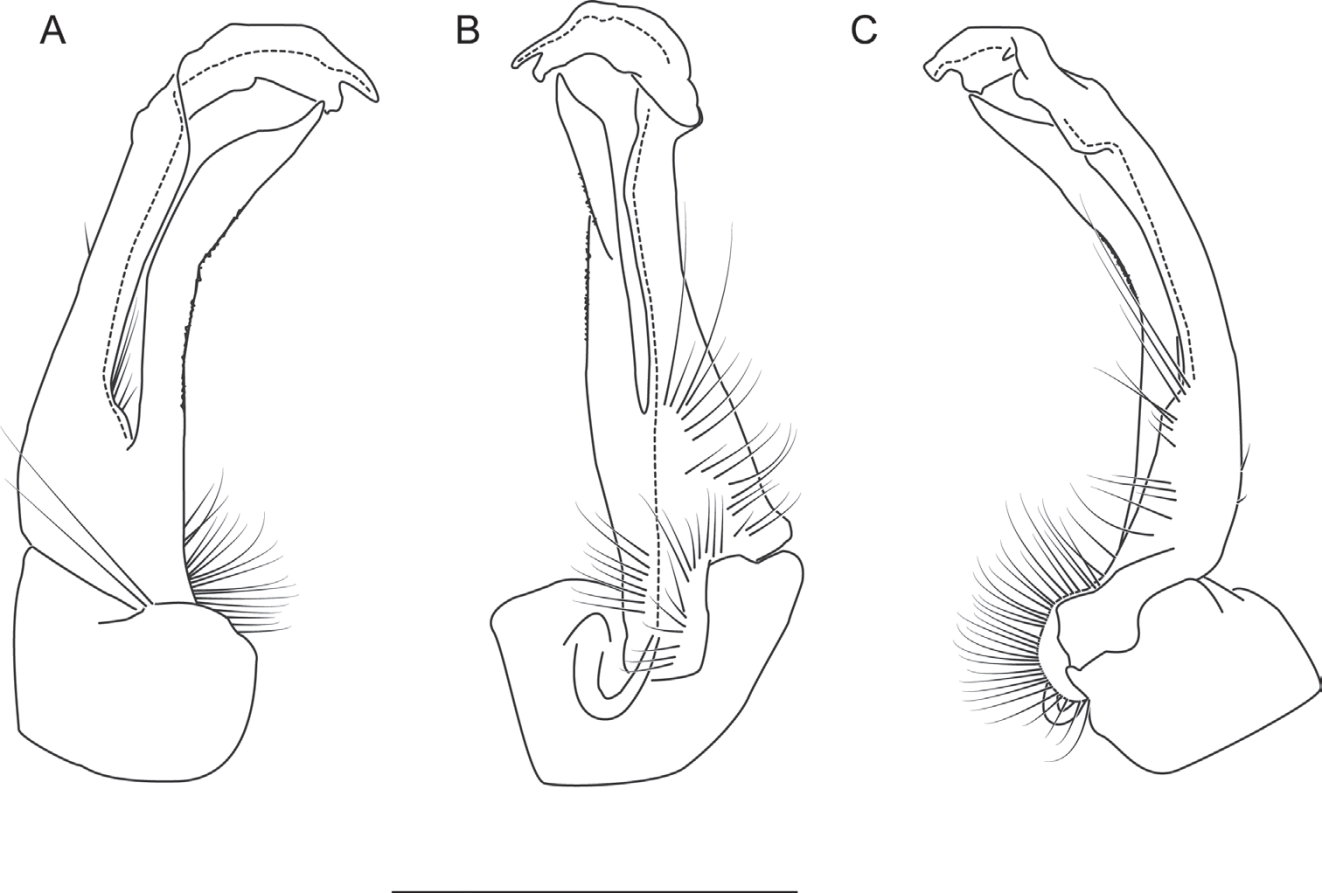
Left gonopod of *Nannariaspalax* sp. nov., male holotype (MPE04377, Rabun County, Georgia) **A** anterior view **B** medial view **C** posterior view. Scale bar: 1 mm.

Vernacular name: The Mole Twisted-Claw Millipede Figs 41 , 42

##### Material examined.

**Type material: *Holotype***: United States – **Georgia** • ♂; Rabun County, Clayton: Chattahoochee National Forest, Warwoman Dell Recreation Area, nature trail near picnic area; 34.8813°N, -83.3539°W, ±8m; elev. 644 m; 13 October 2018; D. A. Hennen leg.; buried in dark sandy soil in patch of beech, tuliptree, rhododendron woods; VTEC, MPE04377. ***Paratypes***: United States – **Georgia** • 2 ♂♂; same collection data as for holotype; VTEC, MPE04378, MPE04388). Complete material examined information listed in Suppl. material 1.

##### Diagnosis.

Adults of *Nannariaspalax* sp. nov. can be separated from the geographically close and morphologically similar species *N.antarctica* sp. nov. and *N.nessa* sp. nov. by the following characters. Acropodite medial flange laminate, rather than lobed as in *N.nessa* sp. nov. Acropodite tip lateral flange a projecting lobe, making the acropodite appear distally bilobed, but not deeply bifurcate as in *N.antarctica* sp. nov. Prefemoral process laminate and curving medially, with medial margin jagged, rather than notched at midpoint as in *N.nessa* sp. nov. or curving laterally as in *N.antarctica* sp. nov.

##### Description.

Suppl. material 2. Based on holotype (♂) MPE04377. Female morphology unknown.

**Measurements**: Taken from holotype (♂) MPE04377: BL = 26.30, CW = 3.70, IW = 2.88, ISW = 0.92, B10W = 4.50, B10H = 2.40. **Color.** Tergites with two paranotal red spots, collum outlined in red, and tergites with background black (Fig. 41). **Gonopods.** Male gonopod acropodite arc straight, with abrupt bend distally (Fig. 42A–C). Acropodite with wide, acute twist at anterior bend, giving the acropodite a folded appearance (Fig. 42A). Acropodite medial flange laminate and slightly undulating (Fig. 42C). Acropodite tip medial flange a short, triangular projection (Fig. 42C). Acropodite tip lateral flange a projecting lobe (Fig. 42A). Acropodite tip directed medially and bilobed distally, with a dorsal sharp, tapering, horn-like process and a shorter ventral rounded process (Fig. 42A, B). Prefemoral process laminate, curving medially (Fig. 42A). Prefemoral process with a slight taper distally, tip directed cephalically. Medial margin of prefemoral process somewhat jagged (Fig. 42A).

##### Variation.

Acropodite medial flange size varies from small to large. No other noticeable variation observed.

##### Distribution.

*Nannariaspalax* sp. nov. is only known from the type locality in Rabun County, Georgia (Fig. 52).

##### Ecological notes.

Specimens were found buried 3–5 cm in sandy soil near a stream in a cove forest of beech, tuliptree, and rhododendron.

##### Etymology.

The specific name is a noun in apposition derived from the Greek word *spalax*, meaning mole. The name references the mole-like digging behavior of this species, as only three specimens were collected at the type locality, and all were well-buried in the soil.

#### ﻿ Nannaria spiralis sp. nov.

Taxon classificationAnimaliaPolydesmidaXystodesmidae

A6BA5A79-2763-56B2-A1C3-FD2648A8249D

http://zoobank.org/119628C6-23BC-4A6A-BD47-89DE9F3A156F

**Figure 43.**
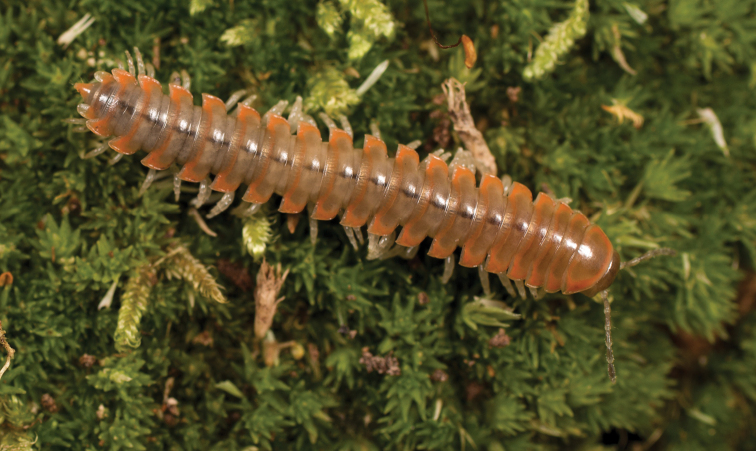
*Nannariaspiralis* sp. nov., in situ, male holotype (MPE02109) from Pendleton County, West Virginia.

**Figure 44.**
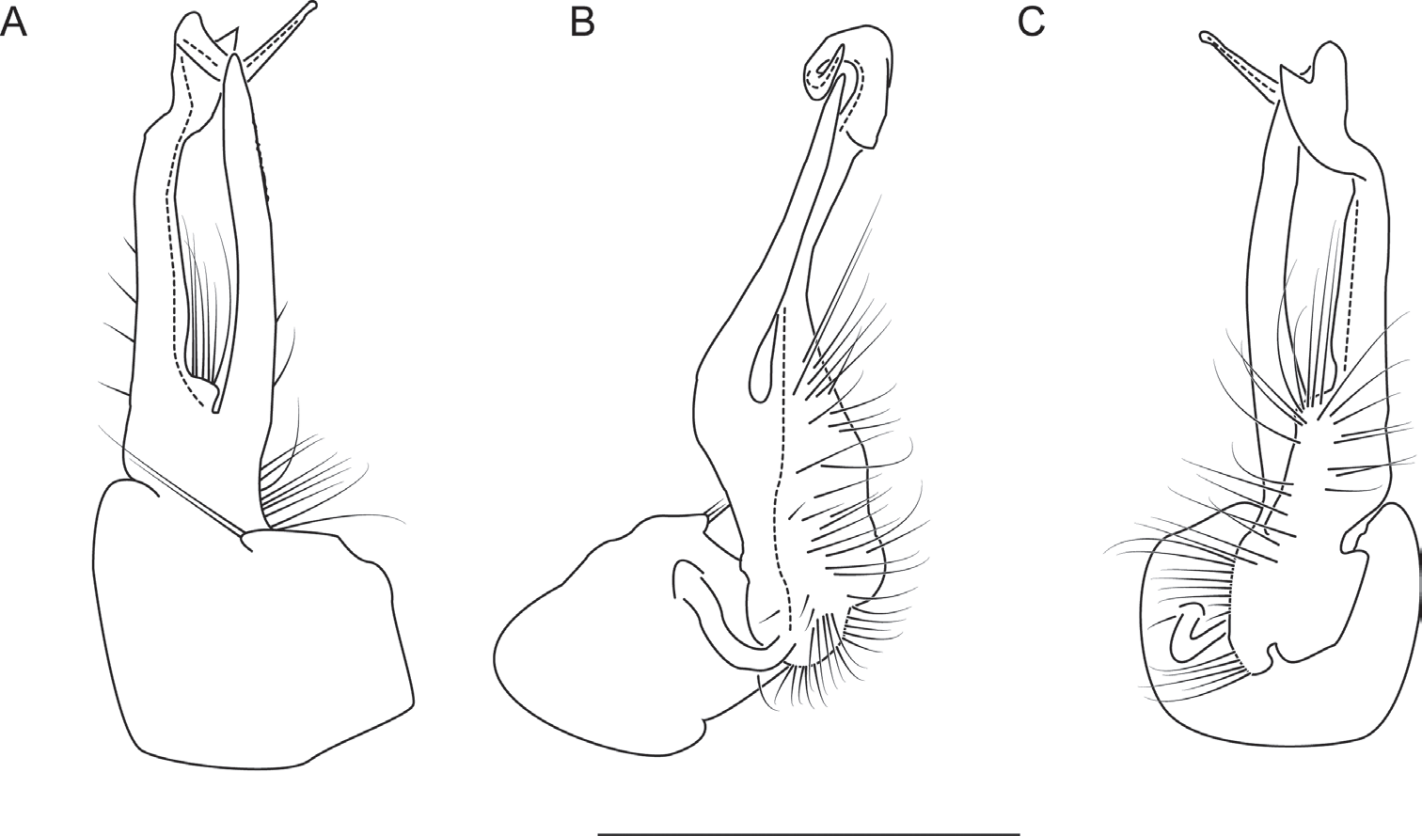
Left gonopod of *Nannariaspiralis* sp. nov. (MPE02111, Pendleton County, West Virginia) **A** anterior view **B** medial view **C** posterior view. Scale bar: 1 mm.

Vernacular name: The Spiral Twisted-Claw Millipede Figs 43 , 44

##### Material examined.

**Type material: *Holotype***: United States – **West Virginia** • ♂; Pendleton County, Brandywine Recreation Area, behind campsite 20, George Washington National Forest; 38.5948°N, -79.1982°W, ±5m; elev. 619 m; 21 June 2016; D. A. Hennen, J. C. Means leg.; moist deciduous litter (oak, maple, hickory) near stream; VTEC, MPE02109. ***Paratypes***: United States – **West Virginia** • 1 ♂ and 1♀; same collection data as for holotype; VTEC, MPE02111, MPE02112; **Virginia** • 1 ♂; Rockingham County, Tomahawk Mountain, ca. 7 mi NNW Rawley Springs, DF site off FS 72; 38.6234°N, -79.0726°W, ±5000 m; elev. 1006 m; 28 May 1988; K. A. Buhlmann leg.; VMNH, NAN0107. **Non type material**: United States – **Virginia** • 1 ♂; Augusta County, 5 mi W Stokesville, comp. 452-8A, trap 2; 38.3524°N, -79.2416°W, ± 2000m; 23 April 1989; B. Flamm leg.; VMNH, NAN0099 • 5 specimens; Augusta County, GWNF, 5 miles west of Stokesville Comp.: 452-8A Trap 3; 38.3524°N, -79.2416°W, ± 2000m; 12 November 1988; B. Flamm leg.; VMNH, NAN0100 • 7 specimens; Augusta County, 5 mi W Stokesville, comp. 452-6 trap 3; 38.3524°N, -79.2416°W, ± 2000m; 12 November 1988; B. Flamm leg.; VMNH, NAN0101 • 17 specimens; same collection data as for preceding; 24 April 1989; VMNH, NAN0102 • 2 specimens; Augusta County, 5 miles W of Stokesville, GWNF comp. 453-1B trap 3; 38.3524°N, -79.2416°W, ± 2000m; 12 November 1988; B. Flamm leg.; VMNH, NAN0103 • 3 specimens; Augusta County, GWNF, ca. 5 miles west of Stokesville Comp. 453-1A, Trap 2; 38.3524°N, -79.2416°W, ± 2000m; 12 November 1988; B. Flamm leg.; VMNH, NAN0104 • 2 specimens; Augusta County, 5 miles W of Stokesville: GWNF comp. 452-6, trap 3; 38.3524°N, -79.2416°W, ± 2000m; 2 September 1989; B. Flamm leg.; VMNH, NAN0105 • 4 specimens; Augusta County, DF site off FS 85, 3 km NE jct. FS 95, Shenandoah Mtn.; 38.4306°N, -79.2743°W, ± 3000m; 28 May 1988; K. A. Buhlmann leg.; VMNH, NAN0106 • 1 specimen; Augusta County, 5 mi W Stokesville, comp. 460-12 trap 3; 38.3524°N, -79.2416°W, ± 2000m; 16 June 1989; B. Flamm leg.; VMNH, NAN0108 • 1 specimen; Augusta County, Shenandoah Mountain, Civil War Hist. site, US 250; 38.3113°N, -79.3842°W, ± 300m; 2 May 1991; C. A. Pague leg.; VMNH, NAN0109 • 1 specimen; Augusta County, 5 mi W Stokesville, comp. 460-5, trap 3; 38.3524°N, -79.2416°W, ± 2000m; 7 August 1989; B. Flamm leg.; VMNH, NAN0110 • 1 specimen; Augusta County, 5 mi W Stokesville, comp. 452-16, trap 2; 38.3524°N, -79.2416°W, ± 2000m; 15 October 1988; B. Flamm leg.; VMNH, NAN0111 • 1 specimen; Augusta County, 5 mi W Stokesville, comp. 452-6, trap 1; 38.3524°N, -79.2416°W, ± 2000m; 24 April 1989; B. Flamm leg.; VMNH, NAN0112 • 2 specimens; Augusta County, GWNF, 5 mi W of Stokesville, comp. 460-5, trap 1; 38.3524°N, -79.2416°W, ± 2000m; 8 July 1989; B. Flamm leg.; VMNH, NAN0113 • 1 specimen; Augusta County, 5 miles W of Stokesville: GWNF comp. 453-18, trap 3; 38.3524°N, -79.2416°W, ± 2000m; 15 October 1988; B. Flamm leg.; VMNH, NAN0114 • 3 specimens; Augusta County, GWNF, ca. 5 mi W of Stokesville, comp. 460-5, trap 1; 38.3524°N, -79.2416°W, ± 2000m; 18 May 1989; B. Flamm leg.; VMNH, NAN0116 • 1 specimen; Augusta County, Ramsey’s Draft, 18 mi N of Staunton; 38.3121°N, -79.356°W, ± 10000m; 19 June 1969; W. A. Shear leg.; VMNH, NAN0118 • 6 specimens; Augusta County, GWNF, 5 mi W of Stokesville Comp. 452-6, Trap 3; 38.3524°N, -79.2416°W, ± 2000m; 17 September 1988; B. Flamm leg.; VMNH, NAN0119 • 1 specimen; Augusta County, GWNF, 5 mi W of Stokesville, comp. 460-12, trap 2; 38.3524°N, -79.2416°W, ± 2000m; 11 November 1988; B. Flamm leg.; VMNH, NAN0530 • 1 specimen; Augusta County, GWNF 460 - 12 T2; 38.3636°N, -79.2602°W, ± 2000m; 14 October 1988; VMNH, NAN0648 • 3 specimens; same collection data as for preceding; 22 December 1988; VMNH, NAN0660 • 1 specimen; Augusta County, GWNF 460 - 3, T1; 38.3658°N, -79.2513°W, ± 2000m; 11 November 1988; VMNH, NAN0649 • 1 specimen; Augusta County, GWNF 453 - 11, T2; 38.3633°N, -79.2382°W, ± 2000m; 12 December 1988; VMNH, NAN0661 • 2 specimens; Augusta County, GWNF 460 - 12 T2; 38.3636°N, -79.2602°W, ± 2000m; 23 April 1989; VMNH, NAN0662 • 5 specimens; Augusta County, GWNF 460 - 12, T3; 38.3636°N, -79.2602°W, ± 2000m; 23 April 1989; VMNH, NAN0652 • 6 specimens; same collection data as for preceding; 11 November 1988; VMNH, NAN0666 • 1 specimen; Highland County, Along US 220, ca. 2 mi. S of Monterey; 38.3882°N, -79.6011°W, ± 3000m; 31 May 1991; K. A. Buhlmann leg.; VMNH, NAN0115 • 1 ♂; Rockingham County, 2.3 km NW of Briery Branch, off Hone Quarry Rd, George Washington National Forest, Hidden Cliff Rocks Trail; 38.452°N, -79.1144°W, ± 5m; elev. 536 m; 24 May 2018; D. A. Hennen, J. C. Means leg.; VTEC, MPE04010; **West Virginia** • 2 ♂♂ and 5 ♀♀; Pendleton County, Brandywine Recreation Area, George Washington National Forest; 38.5948°N, -79.1982°W, ± 5m; elev. 619 m; 21 June 2016; J. C. Means, D. A. Hennen leg.; VTEC, MPE03666, MPE03704, MPE01840, MPE01861, MPE02113, MPE02114, MPE02146. Complete material examined information listed in Suppl. material 1.

##### Diagnosis.

Adults of *Nannariaspiralis* sp. nov. can be separated from the geographically close and morphologically similar species *N.shenandoa* and *N.vellicata* sp. nov. by the following characters. Acropodite undivided, rather than bifurcate as in *N.shenandoa*. Acropodite tip medial flange broad, shield-shaped, rather than triangular with a laminate base as in *N.vellicata* sp. nov. Quadrate process present basomedially on acropodite.

##### Description.

Suppl. material 2. Based on holotype (♂) MPE02109 and paratype (♀) MPE02112.

**Measurements**: Taken from holotype (♂) MPE02109: BL = 27.30, CW = 4.10, IW = 2.85, ISW = 0.96, B10W = 4.85, B10H = 2.70. **Color.** Tergites with two paranotal orange spots, collum outlined in orange, and tergites with background chestnut brown (Fig. 43). **Gonopods.** Male gonopod acropodite arc straight, with abrupt bend distally (Fig. 44A–C). Acropodite with gently undulating twist at anterior bend (Fig. 44C). Acropodite medial flange absent. Acropodite tip medial flange broad and laminate, produced into a shield-shaped process with a sharp point (Fig. 44C). Acropodite tip lateral flange absent. Acropodite tip thin and pointed, acutely recurved (Fig. 44A); tip directed antero-medially. Acropodite with basomedial quadrate process (Fig. 44A). Prefemoral process sinuously tapered (Fig. 44B). Prefemoral process crossing acropodite dorsolaterally; tip directed cephalically (Fig. 44A). **Cyphopods.** Female cyphopod receptacle quadrate, recurved distally.

##### Variation.

No noticeable variation was observed.

##### Distribution.

*Nannariaspiralis* sp. nov. is known from Pendleton County, West Virginia, and the following adjacent Virginia counties: Augusta, Highland, and Rockingham (Fig. 51).

##### Ecological notes.

This species has been found in mesic forests of oak, hickory, and maple, as well as in a rhododendron grove in a rhododendron, hemlock, and oak forest near a stream.

##### Etymology.

This species is named for the spiraling morphology of the acropodite tip, best seen in medial view. The species name is a feminine adjective in the nominative singular, derived from the Latin word *spiralis*, meaning a coil or twist.

#### ﻿ Nannaria swiftae sp. nov.

Taxon classificationAnimaliaPolydesmidaXystodesmidae

63FEF611-15AA-511E-97A9-E88A1AC525F3

http://zoobank.org/BBF344F8-7FC3-49B2-AAE9-6E5CC30EEAF7

**Figure 45.**
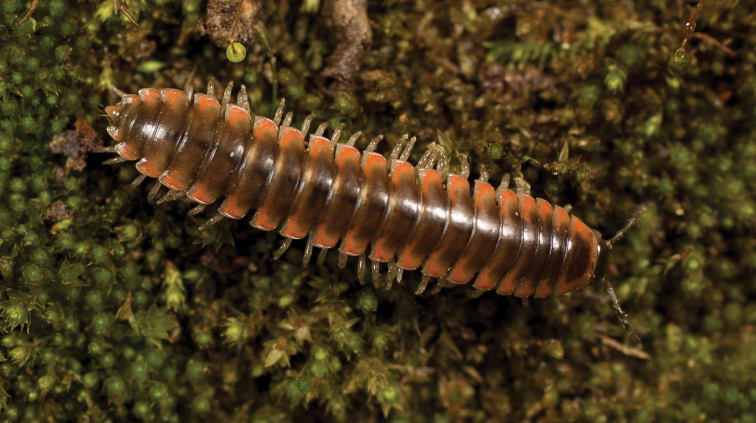
*Nannariaswiftae* sp. nov., in situ, male holotype (MPE01222) from Van Buren County, Tennessee.

**Figure 46.**
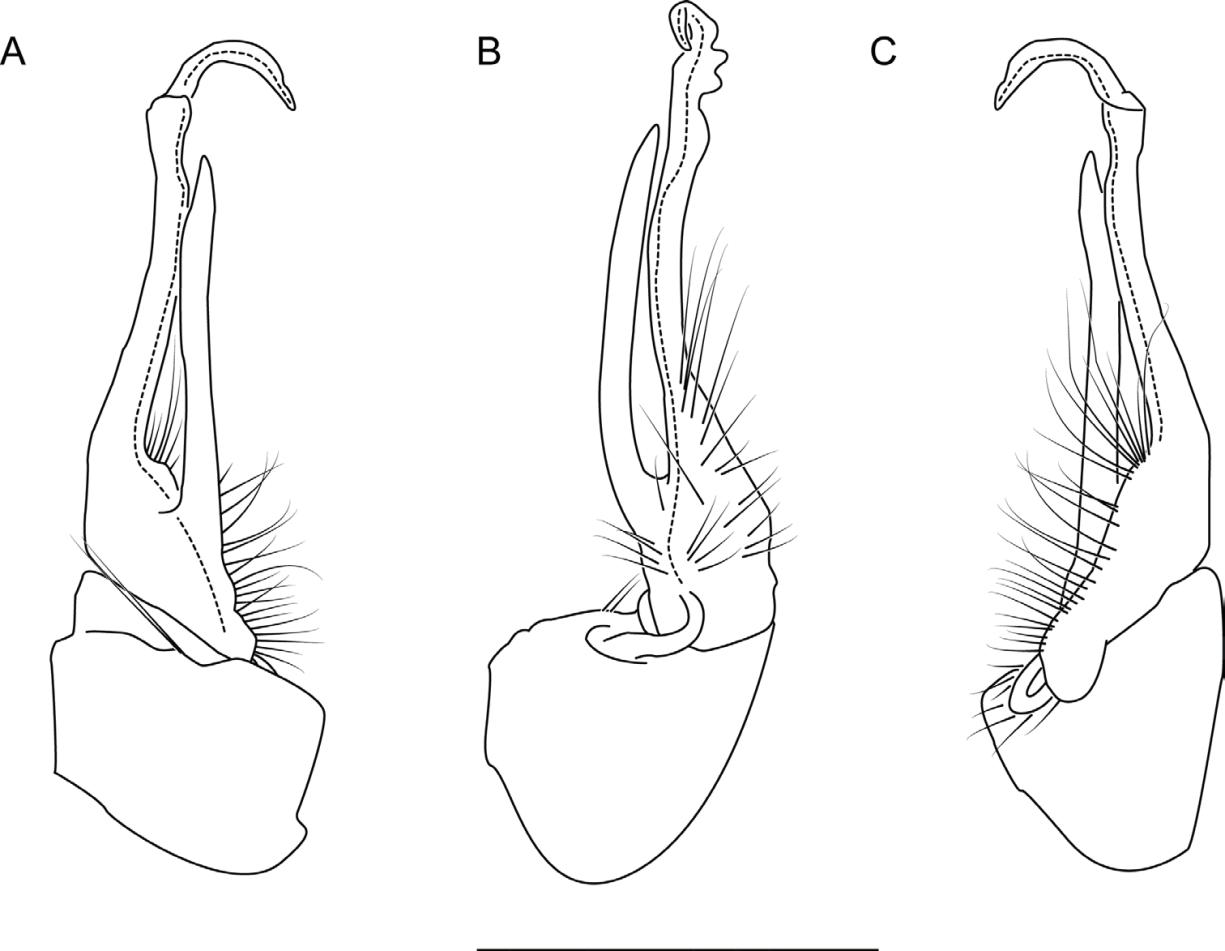
Left gonopod of *Nannariaswiftae* sp. nov., male holotype (MPE01222, Van Buren County, Tennessee) **A** anterior view **B** medial view **C** posterior view. Scale bar: 1 mm.

Vernacular name: The Swift Twisted-Claw Millipede Figs 45 , 46

Nannaria sp. nov. ‘Cratagae’: Means et al. 2021b: 111.

##### Material examined.

**Type material: *Holotype***: United States – **Tennessee** • ♂; Van Buren County, Fall Creek Falls State Park, Crane Creek Falls overlook; 35.6628°N, -85.3498°W, ±5m; elev. 483 m; 22 May 2016; J. C. Means, D. A. Hennen leg.; at base of witch hazel tree, in deciduous forest; VTEC, MPE01222. ***Paratypes***: United States – **Tennessee** • 1 ♀; Van Buren County, Fall Creek Falls State Park, George’s Hole area, hillside beside parking lot; 35.6612°N, -85.3464°W, ±7m; elev. 481 m; 22 May 2016; D. A. Hennen, J. C. Means leg.; moist hemlock, maple, tuliptree, oak, and pine litter; VTEC, MPE01226 • 3 ♂♂; Van Buren County, Fall Creek Falls State Park; 35.6561°N, -85.3478°W ±3000m; 13 May 1979; R. M. Shelley leg.; NCSM, NAN0190. **Non type material**: United States – **Tennessee** • 1 ♂ and 3 ♀♀; Cumberland County, Hwy 68, 9 air mi. SE Crossville; 35.8521°N, -84.9182°W; 8 May 1979; R. K. Tardell leg.; NCSM, NAN0457 • 1 ♂; Monroe County, Little Haw Knob; 35.315°N, -84.0361°W, ± 3000m; elev. 1539 m; 27 May 1958; L. Hubricht leg.; VMNH, NAN0189 • 2 ♀♀; Van Buren County, Fall Creek Falls State Park, George’s hole area, hillside beside parking lot on both sides of road; 35.6612°N, -85.3464°W, ± 7m; elev. 481 m; 22 May 2016; J. C. Means, D. A. Hennen leg.; VTEC, MPE01268, MPE03713. Complete material examined information listed in Suppl. material 1.

##### Diagnosis.

Adults of *Nannariaswiftae* sp. nov. can be separated from the geographically close and morphologically similar species *N.austricola* and *N.scutellaria* by the following characters. Acropodite medial flange lobed and with two small bumps, not simply lobed as in *N.austricola* or with a thin, acuminate triangular process as in *N.scutellaria*. Acropodite tip medial flange absent, rather than lobed as in *N.austricola* or triangular as in *N.scutellaria*.

##### Description.

Suppl. material 2. Based on holotype (♂) MPE01222 and paratype (♀) MPE01226.

**Measurements**: Taken from holotype (♂) MPE01222: BL = 22.40, CW = 3.50, IW = 2.50, ISW = 0.88, B10W = 4.35, B10H = 2.55. **Color.** Tergites with two paranotal orange spots, collum outlined in orange, and tergites with background chestnut brown (Fig. 45). **Gonopods.** Male gonopod acropodite arc straight, with abrupt bend distally (Fig. 46A–C). Acropodite with acute twist at anterior bend, folding distal zone of acropodite (Fig. 46C). Acropodite medial flange lobed, with two small, rounded bumps visible in medial view (Fig. 46B). Acropodite tip medial and lateral flanges absent. Acropodite tip slightly tapering distally and directed medially (Fig. 46A). Acropodite with small basomedial quadrate process. Prefemoral process acicular (Fig. 46A). Prefemoral process tip directed cephalically. **Cyphopods.** Female cyphopod receptacle sinuous, blunt, and rounded distally.

##### Variation.

No noticeable variation observed.

##### Distribution.

*Nannariaswiftae* sp. nov. is only known from Tennessee and has been collected in the following counties: Cumberland, Monroe, and Van Buren (Fig. 52).

##### Ecological notes.

This species has been collected in mesic forests with hemlock, maple, oak, tuliptree, witch hazel, and pine, at elevations ranging from 481 m to 1539 m.

##### Etymology.

The specific name is a noun in the genitive case derived as a matronym, and is named in honor of the artist Taylor Swift, in recognition of her talent as a songwriter and performer and in appreciation of the enjoyment her music has brought DAH.

#### ﻿ Nannaria vellicata sp. nov.

Taxon classificationAnimaliaPolydesmidaXystodesmidae

076796ED-18B1-5A6C-BB63-5C8F02C52DB6

http://zoobank.org/F764E7D5-A1C2-4DBD-AC12-5818269A75AE

**Figure 47.**
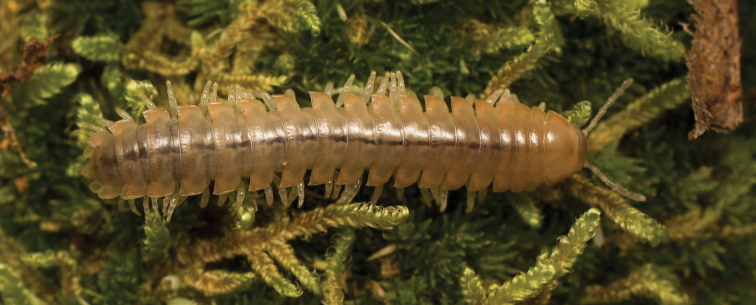
*Nannariavellicata* sp. nov., in situ, male holotype (MPE02060) from Augusta County, Virginia.

**Figure 48.**
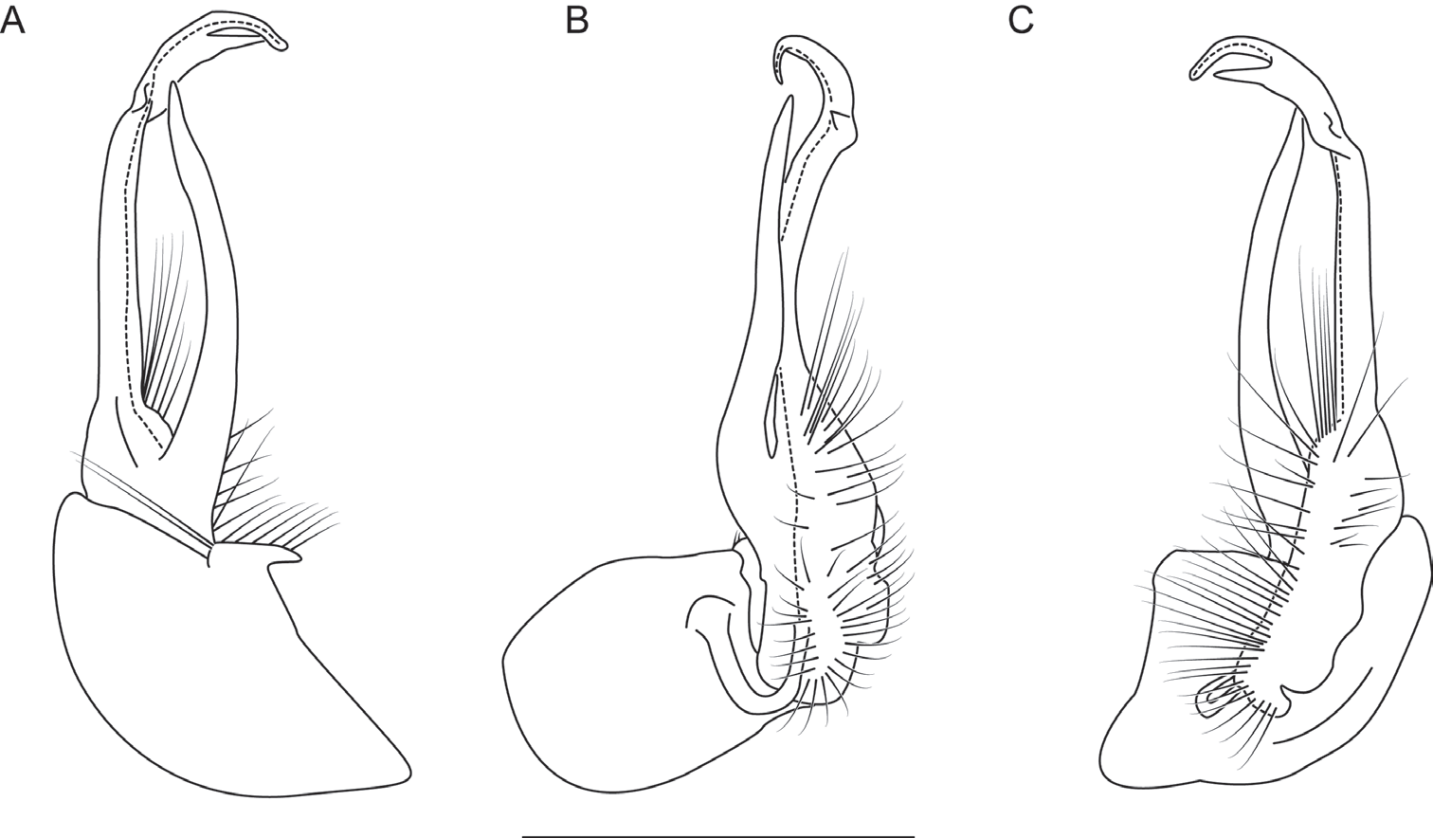
Left gonopod of *Nannariavellicata* sp. nov., male holotype (MPE02060, Augusta County, Virginia) **A** anterior view **B** medial view **C** posterior view. Scale bar: 1 mm.

Vernacular name: The Misty Twisted-Claw Millipede Figs 47 , 48

##### Material examined.

**Type material: *Holotype***: United States – **Virginia** • ♂; Augusta County, 4.9 km WNW Stokesville, 0.8 km SE Todd Lake Recreation Area, George Washington National Forest, Trimble Mountain Trail off Forest Road 95; 38.3605°N, -79.2048°W, ±9m; elev. 602 m; 24 June 2016; D. A. Hennen, J. C. Means leg.; moist oak, maple forest with scattered pines, on hillside by path; VTEC, MPE02060. ***Paratypes***: United States – **Virginia** • 1 ♂; Alleghany County, Griffith; 37.8658°N, -79.7269°W, ±3000m; 8 October 1949; R. L. Hoffman leg.; VMNH, NAN0054 • 1 ♀; Rockbridge County, 12.4 km SW Goshen along VA-780, George Washington National Forest beside Brattons Run; 37.9021°N, -79.5882°W, ±7m; elev. 571 m; 14 November 2017; D. A. Hennen, J. C. Means leg.; base of hillside beside stream in rhododendron and hemlock forest with moist sandy soil, in litter of tuliptree, rhododendron, hemlock, red oak; VTEC, MPE03629. **Non type material**: United States – **Virginia** • 2 ♂♂; Alleghany County, Griffith; 37.8658°N, -79.7269°W, ± 3000m; 5 April 1950; R. L. Hoffman leg.; VMNH, NAN0053 • 3 ♂♂ and 3♀♀; Rockbridge County, 12.4 km SW Goshen along VA-780, George Washington National Forest beside Brattons Run; 37.9021°N, -79.5882°W, ± 7m; elev. 571 m; 14 November 2017; J. C. Means, D. A. Hennen leg.; VTEC, MPE03492, MPE03628, MPE03631, MPE03630, MPE03632, MPE03633. Complete material examined information listed in Suppl. material 1.

##### Diagnosis.

Adults of *Nannariavellicata* can be separated from the geographically close and morphologically similar species *N.orycta* sp. nov. and *N.spiralis* sp. nov. by the following characters. Acropodite anterior bend with a gentle twist, rather than acutely twisted as in *N.orycta* sp. nov. Acropodite tip medial flange triangular with a laminate base, rather than broad and shield-shaped as in *N.spiralis* sp. nov. Acropodite undivided distally, rather than bifurcate as in *N.orycta* sp. nov.

##### Description.

Suppl. material 2. Based on holotype (♂) MPE02060 and paratype (♀) MPE03629.

**Measurements**: Taken from holotype (♂) MPE02060: BL = 23.50, CW = 4.20, IW = 2.82, ISW = 1.02, B10W = 5.00, B10H = 2.75. **Color.** Tergites with two paranotal orange spots, collum outlined in orange, and tergites with background chestnut brown (Fig. 47). **Gonopods.** Male gonopod acropodite arc straight, with abrupt bend distally (Fig. 48A–C). Acropodite anterior bend with gentle twist (Fig. 48A). Acropodite medial flange lobed (Fig. 48C). Acropodite tip medial flange triangular, extended and with a laminate base (Fig. 48C). Acropodite tip entire, directed medially and slightly curved ventrally (Fig. 48A, B). Acropodite tip lateral flange absent. Prefemoral process simple, curving laterally (Fig. 48A). Prefemoral process tip directed laterally. **Cyphopods.** Female cyphopod receptacle triangular.

##### Variation.

No noticeable variation observed.

##### Distribution.

*Nannariavellicata* sp. nov. is known from three counties in Virginia: Alleghany, Augusta, and Rockbridge (Fig. 51).

##### Ecological notes.

This species has been collected from both rhododendron-hemlock cove habitats and general mesic deciduous forests, at elevations between 571 m and 602 m.

##### Etymology.

This species is named for the shape of the acropodite tip and medial flange, which together resemble a thumb and index finger in a pinching motion. The specific epithet is a feminine adjective derived from the Latin word *vellico*, meaning pinch.

#### ﻿ Nannaria wilsoni

Taxon classificationAnimaliaPolydesmidaXystodesmidae

Hoffman, 1949

CD098CA7-FC4C-5B97-8380-0405AE0C351B

**Figure 49.**
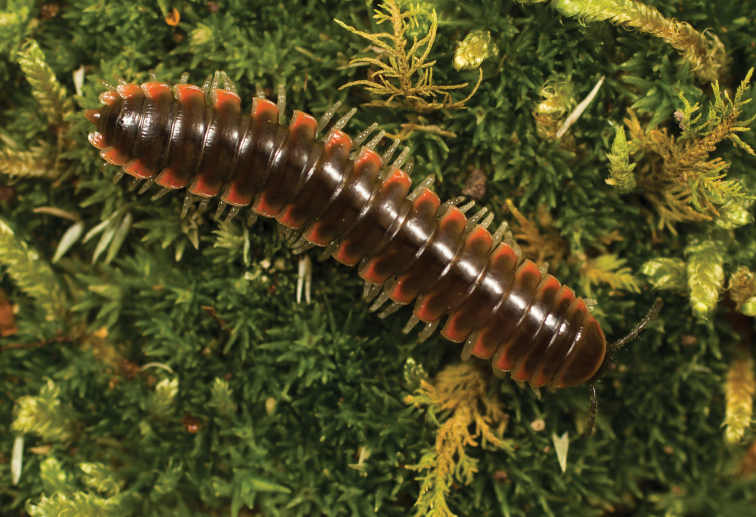
*Nannariawilsoni*, in situ, male (MPE02123) from Giles County, Virginia.

**Figure 50.**
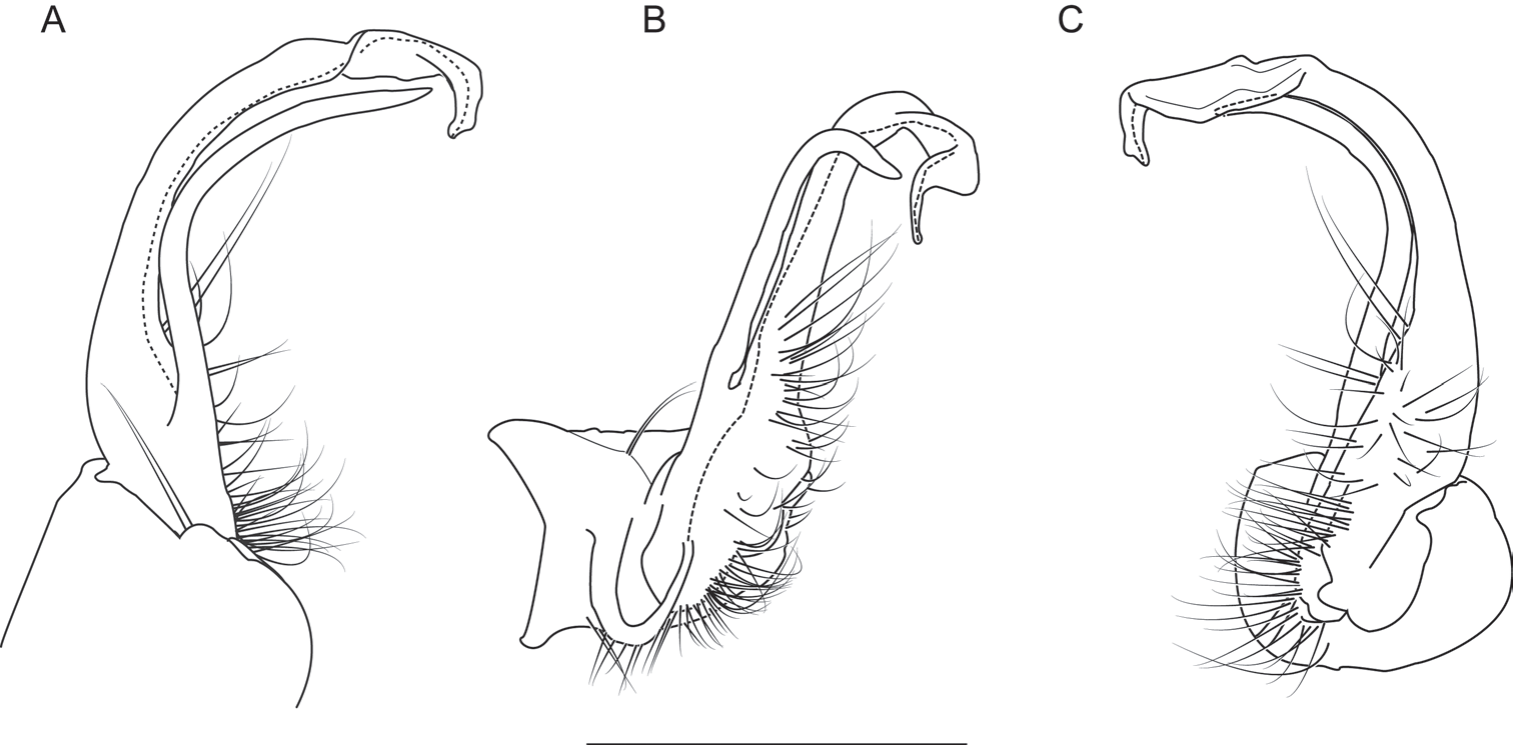
Left gonopod of *Nannariawilsoni* (MPE02123, Giles County, Virginia) **A** anterior view **B** medial view **C** posterior view. Scale bar: 1 mm.

Vernacular name: The Wilson Twisted-Claw Millipede Figs 49 , 50

Nannaria wilsoni Hoffman, 1949b: 386, figs 15, 16. Chamberlin and Hoffman 1958: 42. Hoffman 1999: 368 (352 in pdf version). Means et al. 2021a: S70, fig. 3.

##### Type material.

***Holotype***: United States – **Virginia** • ♂; Giles County, Mountain Lake Biological Station; 37.3769°N, -80.5227°W, ±1000 m; June and July 1947; Hobbs, Walton, Wilson leg.; USNM Entomology, no. 1808; (non vidi). ***Paratypes***: United States – **Virginia** • 1 ♂; same collection data as for holotype; USNM Entomology, no. 1808; (non vidi) • 6 ♂♂; Giles County, Mountain Lake Biological Station; 37.3769°N, -80.5227°W, ±1000 m; Wilson, R. L. Hoffman leg.; 28 August 1947; VMNH, NAN0300 (vidi). Habitat at the type locality was a deciduous forest of oak, maple, and yellow poplar (tuliptree), with an undergrowth composed of ericaceous shrubs (Hoffman 1949b). In the original description of this species, Hoffman noted that he retained six “topoparatypes” in his personal collection (Hoffman 1949b). These specimens are VMNH lot NAN0300, a vial that contains six males in a jar labeled “PARATYPE.”

##### Material examined.

**Non type material**: United States – **Virginia** • 1 specimen; Giles County, Mt. Lake; 37.3769°N, -80.5227°W, ± 1000m; 27 August 1947; C. M. Wilson leg.; MCZ, 87614 • 1 specimen; Giles County, Mountain Lake Biological Station; 37.3743°N, -80.524°W, ± 2000m; W. A. Shear leg.; VMNH, NAN0035 • 1 specimen; same collection data as for preceding; 1 July 1936; P. R. Burch leg.; VMNH, NAN0299 • 4 specimens; Giles County, Cascades of Little Stony Creek; 37.3617°N, -80.5866°W, ± 5000m; 17 May 1961; R. L. Hoffman leg.; VMNH, NAN0301 • 1 ♀; Giles County, near Mountain Lake; 37.3743°N, -80.524°W, ± 5000m; 4 August 1963; Kosztarab leg.; VMNH, NAN0302 • 2 ♀♀; Giles County, gully off path in Mountain Lake Biological Station Wilderness, off road to Salt Pond Mountain; 37.3891°N, -80.5052°W; 1 May 2016; J. C. Means leg.; VTEC, MPE01149, MPE01150 • 3 ♂♂ and 3 ♀♀; Giles County, Mountain Lake Wilderness, near Mountain Lake Biological Station, on trail to War Spur Overlook, off Rt. 613; 37.3892°N, -80.5057°W; elev. 1125 m; 3 October 2016; J. C. Means, D. A. Hennen, V. Wong leg.; under moss at base of white oak tree, in deciduous woods of witch hazel, chestnut oak, white oak; VTEC, MPE02123, MPE02132, MPE02133, MPE02131, MPE02134, MPE02135 • 1 ♂; Giles County, Mountain Lake Biological Station Wilderness; 37.3751°N, -80.5218°W, ± 5000m; 2017; J. D. Montemayor leg.; VTEC, MPE02480 • 2 specimens; Montgomery County, Radford; 37.136°N, -80.5665°W, ± 5000m; 1 October 1937; P. R. Burch leg.; VMNH, NAN0297. Complete material examined information listed in Suppl. material 1.

##### Diagnosis.

Adults of *Nannariawilsoni* can be separated from the geographically close and morphologically similar species *N.acroteria* sp. nov. and *N.cymontana* sp. nov. by the following characters. Acropodite medial flange laminate and wide, rather than small and lobed as in *N.cymontana* sp. nov. Dorsal projection near medial flange of acropodite absent, rather than present as in *N.acroteria* sp. nov. Prefemoral process gradually tapering, rather than spear-shaped distally, as in *N.cymontana* sp. nov. Acropodite lacking the small triangular projection near base of prefemoral process present in *N.acroteria* sp. nov.

##### Description.

Suppl. material 2. Based on ♂ (MPE02123) and ♀ (MPE02131).

**Measurements**: Taken from ♂ specimen MPE02123: BL = 27.30, CW = 3.88, IW = 2.73, ISW = 0.93, B10W = 5.06, B10H = 3.00. **Color.** Tergites with two paranotal peach spots, collum outlined in peach, and tergites with background chestnut brown (Fig. 49). **Gonopods.** Male gonopod acropodite arc gradually curving (Fig. 50A–-C). Acropodite with a gently undulating twist at anterior bend (Fig. 50A). Acropodite medial flange laminate (Fig. 50C), acropodite tip medial flange absent. Acropodite tip lateral flange lobed, with a small bump just before the tip (Fig. 50A). Acropodite tip blunt, directed ventrally (Fig. 50A, B). Prefemoral process sinuous and curving medially (Fig. 50A). Prefemoral process tip directed medially. **Cyphopods.** Female cyphopod receptacle an enlarged triangular hood.

##### Variation.

No notable variation observed.

##### Distribution.

**Figure 51.**
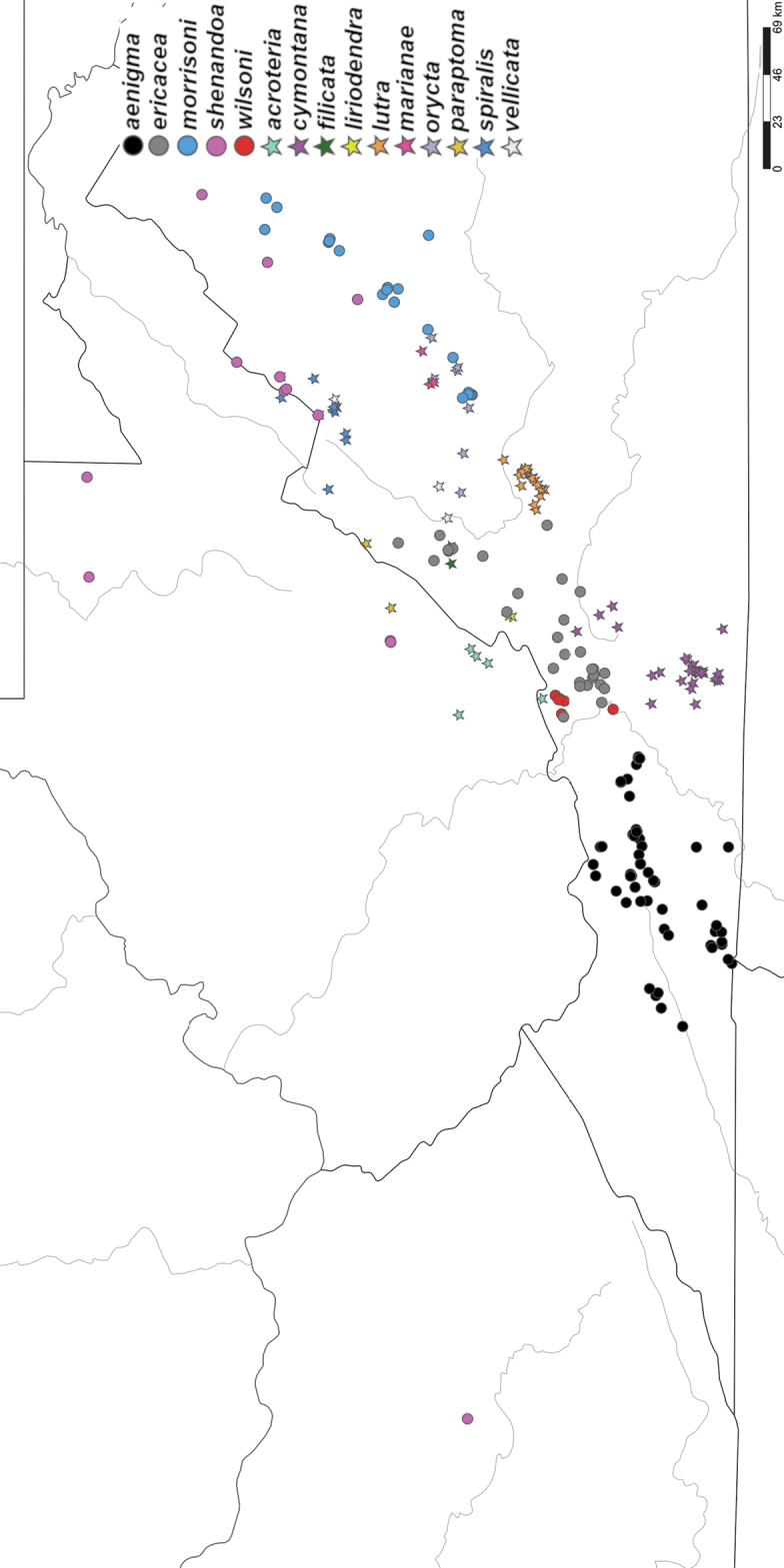
Distribution of the Central Appalachian species of the *Nannariawilsoni* species group. Dots = described species, stars = new species.

*Nannariawilsoni* is limited almost entirely to Giles County, with one dubious record from Montgomery County in southwest Virginia (Fig. 51).

##### Ecological notes.

Habitat information from museum specimens is limited, but *Nannariawilsoni* has been collected from oak, maple, and rhododendron forests.

##### Etymology.

This species was named in honor of Charles M. Wilson, who collected the species at Mountain Lake Biological Station in Giles County (Hoffman 1949b).

### ﻿Key to the *wilsoni* species group of *Nannaria* Chamberlin, 1918

**Table:** 

1	Gonopod acropodite anterior bend twist absent (Fig. 29)	**2**
–	Gonopod acropodite anterior bend twist present (Figs 13, 50)	**4**
2	Prefemoral process stout, projecting at a 45° angle from telopodite (Fig. 27A); acropodite tip with laminate, jagged lateral flange, much larger than other acropodite branch. Augusta Co., Virginia	***N.marianae* sp. nov. (Figs 26, 27)**
–	Prefemoral process thin, acicular or curving, not projecting at a 45° angle from telopodite (Fig. 29A); acropodite tip lacking lateral flange; bifurcations of acropodite of similar size and shape	**3**
3	Prefemoral process acicular; acropodite tip directed medially (Fig. 29A). Blue Ridge Mountains from Page Co. south to Amherst Co., Virginia	***N.morrisoni* (Figs 28, 29)**
–	Prefemoral process curving; acropodite tip directed caudally (Fig. 40A). Augusta, Rockingham, and Shenandoah cos., Virginia; west to Greenbrier, Marion, Pocahontas, Preston cos., West Virginia; and Powell Co., Kentucky	***N.shenandoa* (Figs 39, 40)**
4	Acropodite anterior bend slightly twisted, in the shape of a smoothly-undulating helix (Fig. 50)	**5**
–	Acropodite anterior bend acutely bent, appearing crimped or pinched (Fig. 13)	**17**
5	Prefemoral process arising from midway up acropodite (Fig. 23); acropodite arc straight, with bend at midpoint (Fig. 23); acropodite setae present from base of acropodite to three-quarters up the full acropodite length. Buncombe and Rutherford cos., North Carolina and Greenville Co., South Carolina	***N.lithographa* sp. nov. (Figs 22, 23)**
–	Prefemoral process arising from base of acropodite (Fig. 50); acropodite arc either gradually curving (Fig. 34), or straight with abrupt bend at tip (Fig. 17); acropodite setae present from base of acropodite to halfway up the full acropodite length	**6**
6	Acropodite arc straight, with abrupt bend at tip (Fig. 17)	**7**
–	Acropodite arc gradually curving (Fig. 34)	**10**
7	Acropodite medial flange absent (Fig. 17)	**8**
–	Acropodite medial flange present (Fig. 48)	**9**
8	Acropodite tip medial flange triangular (Fig. 17); acropodite tip lateral flange triangular, fin-shaped; acropodite weakly curving, not tapering towards tip. Ridge and Valley region, from Bath Co. south to Giles and Montgomery cos., Virginia	***N.ericacea* (Figs 16, 17)**
–	Acropodite tip medial flange laminate, shield-shaped (Fig. 44); acropodite tip lateral flange absent; acropodite spiraling and tapering towards tip. Augusta, Highland, Rockingham cos., Virginia and Pendleton Co., West Virginia	***N.spiralis* sp. nov. (Figs 43, 44)**
9	Acropodite tip medial flange lobed and recurved (Fig. 36); acropodite tip lateral flange small, lobed; acropodite not tapering distally. Lumpkin and Towns cos., Georgia	***N.rhododendra* sp. nov. (Figs 35, 36)**
–	Acropodite tip medial flange triangular (Fig. 48); acropodite tip lateral flange absent; acropodite tapering distally. Alleghany, Augusta, and Rockbridge cos., Virginia	***N.vellicata* sp. nov. (Figs 47, 48)**
10	Acropodite medial flange lacking a dorsal median projection (Fig. 50)	**11**
–	Acropodite medial flange with a dorsal median projection (Fig. 8)	**14**
11	Acropodite tip bifurcate (Fig. 19); acropodite tip medial flange laminate, sharp, caudally-directed. Alleghany Co., Virginia	***N.filicata* sp. nov. (Figs 18, 19)**
–	Acropodite tip entire, undivided (Fig. 21); acropodite tip medial flange absent	**12**
12	Base of prefemoral process crimped and bent on medial side (Fig. 21B); prefemoral process straight, not curving in anterior view (Fig. 21A); prefemoral process tip directed cephalically. Craig Co., Virginia	***N.liriodendra* sp. nov. (Fig. 20, 21)**
–	Base of prefemoral process not crimped, either unmodified (Fig. 15B) or with at most a basal swelling (Fig. 50B); prefemoral process curving medially in anterior view (Fig. 15A); prefemoral process tip directed medially	**13**
13	Acropodite medial flange large, laminate (Fig. 50C); acropodite tip lateral flange rounded (Fig. 50C); base of prefemoral process with a slight swelling on medial side; prefemoral process tip without preapical lateral expansion. Giles and Montgomery cos., Virginia	***N.wilsoni* (Figs 49, 50)**
–	Acropodite medial flange small, lobed (Fig. 15C); acropodite tip lateral flange sharp (Fig. 15A); base of prefemoral process unmodified; prefemoral process tip spear-shaped, with preapical lateral expansion. Carroll, Floyd, Montgomery, Patrick, and Roanoke cos., Virginia	***N.cymontana* sp. nov. (Figs 14, 15)**
14	Prefemoral process acicular to slightly curving (Fig. 8); prefemoral process 3/4 the length of the acropodite or less (Fig. 34)	**15**
–	Prefemoral process sinuous, strongly curving (Fig. 6); prefemoral process greater than 3/4 the length of the acropodite (Fig. 25)	**16**
15	Acropodite dorsal triangular projection small to medium-sized (Fig. 8A); acropodite tip smoothly curving (Fig. 8A). Southwest Virginia west of the New River	***N.aenigma* (Figs 7, 8)**
–	Acropodite dorsal triangular projection large (Fig. 34A); acropodite tip straight, with distal kink (Fig. 34A). Pocahontas Co., West Virginia and Bath Co., Virginia	***N.paraptoma* sp. nov. (Fig. 34)**
16	Acropodite dorsal projection triangular (Fig. 6A); acropodite tip lateral flange absent (Fig. 6C); base of prefemoral process with a short, thin triangular process (Fig. 6B). Greenbrier and Monroe cos., West Virginia and Giles Co., Virginia	***N.acroteria* sp. nov. (Figs 5, 6)**
–	Acropodite dorsal projection rounded, dome-shaped (Fig. 25A); acropodite tip lateral flange bilobed (Fig. 25C); base of prefemoral process lacking a triangular process (Fig. 25B). Bedford, Botetourt, Rockbridge cos., Virginia	***N.lutra* sp. nov. (Figs 24, 25)**
17	Acropodite tip bifurcate (Figs 12, 33); acropodite tip medial flange large, laminate, expanded into a separate acropodite branch (Figs 12, 33)	**18**
–	Acropodite tip entire, not split into two branches of similar size (Fig. 10); acropodite tip medial flange absent (Fig. 31), triangular Fig. 38), or lobed (Fig. 13)	**19**
18	Acropodite medial flange pointed, tooth-like (Fig. 12C); acropodite lateral flange lobed (Fig. 12A); acropodite tips directed medially (Fig. 12C); prefemoral process sinuously tapering, with a broad base (Fig. 12A). Macon Co., North Carolina south through Dawson, Towns, and Union cos., Georgia	***N.antarctica* sp. nov. (Figs 11, 12)**
–	Acropodite medial flange laminate Fig. 33C); acropodite lateral flange absent; acropodite tips directed caudally (Fig. 33A); prefemoral process curving slightly, slightly tapering distally (Fig. 33A). Central Virginia, north of the James River from Rockbridge Co. east through Nelson Co., Virginia	***N.orycta* sp. nov. (Figs 32, 33)**
19	Acropodite medial flange lobed (Fig. 13)	**20**
–	Acropodite medial flange laminate (Fig. 42C) or tooth-like (Fig. 10C)	**22**
20	Acropodite tip medial flange present, lobed (Fig. 13A); prefemoral process curving laterally (Fig. 13A). Macon Co., North Carolina and Rabun Co., Georgia	***N.austricola* (Fig. 13)**
–	Acropodite tip medial flange absent (Fig. 31A); prefemoral process erect (Fig. 46A) or with at most only a slight curve (Fig. 31A)	**21**
21	Acropodite medial flange singly lobed (Fig. 31C); acropodite tip even-sided (Fig. 31A); prefemoral process notched on medial side at midpoint (Fig. 31A). Macon Co., North Carolina and Rabun Co., Georgia	***N.nessa* sp. nov. (Figs 30, 31)**
–	Acropodite medial flange composed of two rounded bumps (Fig. 46B); acropodite tip tapered distally (Fig. 46A); prefemoral process even-sided, lacking a notch at midpoint (Fig. 46A). Cumberland, Monroe, and Van Buren cos., Tennessee	***N.swiftae* sp. nov. (Figs 45, 46)**
22	Acropodite medial flange laminate (Fig. 42C); acropodite tip lateral flange lobed, projecting (Fig. 42A); prefemoral process medial margin jagged (Fig. 42A). Rabun Co., Georgia	***N.spalax* sp. nov. (Figs 41, 42)**
–	Acropodite medial flange tooth-like (Figs 10C, 38C); acropodite tip lateral flange absent (Fig. 10A); prefemoral process medial margin smooth (Fig. 10A)	**23**
23	Acropodite medial flange hooked, even-sided (Fig. 10C); acropodite tip medial flange always absent (Fig. 10); prefemoral process laminate (Fig. 10). Dawson and Lumpkin cos., Georgia	***N.amicalola* sp. nov. (Figs 9, 10)**
–	Acropodite medial flange straight, tapered (Fig. 38C); acropodite tip medial flange triangular (Fig. 38), small to large or rarely laminate, sometimes absent; prefemoral process sinuously tapered (Fig. 38). Sevier and Cocke cos., Tennessee and Clay, Haywood, Jackson, Swain, and Transylvania cos., North Carolina	***N.scutellaria* (Figs 37, 38)**

### ﻿Distributional notes

The distribution of the Central Appalachian species of the *wilsoni* species group extends from southwest Virginia and adjacent Kentucky north to West Virginia and the Maryland border (Fig. 51). Species distributions in this geographic cluster are often limited by rivers and mountain ranges. This is shown in the distribution of *N.aenigma*, for example, which only occurs west of the New River. The courses of the James and Roanoke Rivers also delimit portions of the distribution of multiple species, as do the Blue Ridge Mountains, and to a lesser extent, the mountains in the Ridge and Valley Province of Virginia. One species in this cluster that does not have its distribution greatly affected by physical geography is *N.shenandoa*. This species has the largest distribution of any species in the cluster (and within the *wilsoni* species group), extending from eastern Kentucky to northern Virginia across multiple mountain ranges and rivers. Specimens of *N.shenandoa* from across its range, however, have no discernable differences in gonopod morphology, and genetically appear to form a single species. Concerted collecting in the gap in its distribution in West Virginia and Kentucky may reveal more localities for this species.

**Figure 52.**
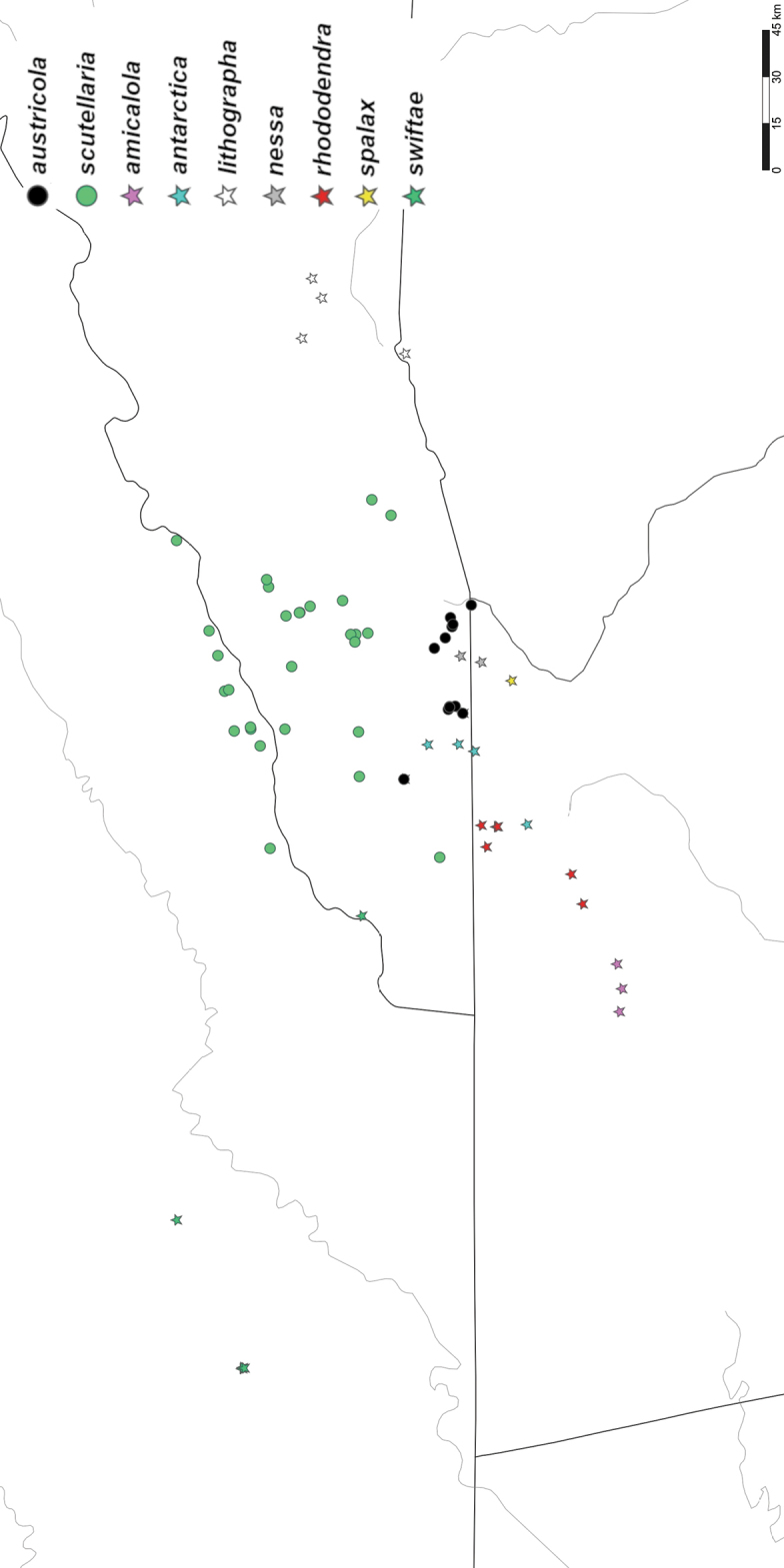
Distribution of the Southern Appalachian species of the *Nannariawilsoni* species group. Dots = described species, stars = new species.

The distribution of the Southern Appalachian species cluster extends from western North Carolina and adjacent Tennessee south through northern Georgia and South Carolina (Fig. 52). The distributions of species in this cluster tend to be small, with many species only known from a few collection localities. The only major known river barrier for this cluster is the French Broad River, which separates the distributions of *N.lithographa* sp. nov. and *N.scutellaria*. The French Broad River is also known to act as a biogeographical barrier causing vicariance for other dispersal-limited arthropod taxa, including flightless beetles (Kane et al. 1990; Caterino and Langton-Myers 2019), harvestmen (Thomas and Hedin 2008; Hedin and McCormack 2017), and *Nesticus* Thorell, 1869 spiders (Hedin 1997). The influence of other regional rivers, such as the Little Tennessee and Hiwassee Rivers, as barriers to species dispersal in this group remains to be investigated. *Nannariascutellaria*, for example, has high morphological variation, and its distribution crosses both the Little Tennessee and Tuckasegee Rivers. Future investigations into the phylogeographical concordance of its distribution and morphological variation may help to resolve questions of cryptic species within *N.scutellaria*. The southern extent of this species cluster is limited by the transition zone from the Blue Ridge physiographic province to the Piedmont in northern Georgia.

## ﻿Discussion

With the description of 17 new species, the species diversity of the *Nannariawilsoni* species group has more than tripled to 24 described species and the total richness in the Xystodesmidae has increased to 539 species. The Southern Appalachian Mountains held a plethora of new species: only two of the species in that region had been previously described. It is almost certain that more *Nannaria* species will be found in the region, particularly in the mesic cove habitats of northern Georgia and adjacent South Carolina. Both the Central Appalachian and Southern Appalachian Regions likely harbor new species. In Central Appalachia, large portions of West Virginia have not been sampled for *Nannaria* and other millipedes. Recent revisionary work on the genus *Brachoria* Chamberlin, 1939 revealed ten new species in the Appalachian Mountains, eight of which were described from extreme southwestern Virginia, eastern Kentucky, and southern West Virginia (Marek 2010). Millipedes in the genus *Brachoria* are large-bodied (40–60 mm in length), brightly colored, and comparatively easier to collect in the field than *Nannaria*, so it is likely new *Nannaria* species may have been missed during previous collecting in this area, and that species diversity of the region is significantly underestimated.

The number of described species in the genus *Nannaria* now stands at 78 (Table 1), with its center of species diversity in the Appalachian Mountains of eastern North America. This makes *Nannaria* the largest genus in the Xystodesmidae, a record previously held by the genus *Rhysodesmus* Cook, 1895, which contains 72 species (Means et al. 2021a). Among all North American diplopods, its species richness is only second to the chordeumatidan genus *Cleidogona* Cook, 1895 with 84 species. The great diversity of *Nannaria* in the Appalachian Mountains mirrors that of the genus *Sigmoria*, which exhibits a nonadaptive radiation of 67 mostly parapatric species (Means et al. 2021a). *Nannaria* species have cryptic color patterns, and none of the species can be considered aposematic like apheloriine species, but the genus has still speciated tremendously in the Appalachians, likely due in part to the physiographic history of the region. The Appalachian Mountains are an old mountain range, estimated to have formed around 480 mya during the Ordovician Period, and the region has a markedly rugged terrain. An important factor influencing speciation in *Nannaria* could be vicariance as a result of allopatric fragmentation of populations, as has been suggested for *Brachoria* and other Xystodesmidae (Marek 2010) in the Appalachian Mountains.

The well-delineated clades of the *Nannariaminor* and *Nannariawilsoni* species groups suggest that the distinction between the two may warrant division at a higher taxonomic level, rather than as species groups, and further study is underway to determine if these groups merit division. The differences between them are supported by genetic, morphological, and biogeographical data. The splits between the *minor* and *wilsoni* species groups, as well as amongst the genus *Nannaria* and *O.pulchella* are all well supported, with bootstrap values greater than 70.

The *minor* and *wilsoni* species groups are also separable morphologically based on the structure of the gonopods (see Genus diagnosis above). The attachment of the prefemoral process at the base of the acropodite in the *wilsoni* species group; lack of an elongation between coxa and telopodite (the basal zone), presence of a twist at the anterior bend, and lack of setae past more than three-quarters the length of the acropodite separate it from the *minor* species group. Species in the *minor* species group have a prefemoral process attached to an acropodite shelf, an elongation between the coxa and telopodite, lack a twist at the anterior bend, and often have setae present at the anterior bend of the acropodite. The valves of the cyphopods appear to vary intraspecifically, but the epigyne, the anterior ventral margin of the third body ring in females (Blower 1985; Zahnle et al. 2020), appears to have utility for female species identification and is perhaps intraspecifically static. Additionally, the receptacle of the female cyphopods differs greatly in size and shape, and may allow identification of females to species in the *wilsoni* group. The *minor* species group is not known to possess such modifications to its cyphopod receptacle, though in *O.pulchella*, the cyphopod receptacle is a large, erect, laminate structure that entirely covers the cyphopod valves. The morphology of the cyphopod receptacles is noted in the morphological matrix and species accounts for each species for which female specimens were available. This will permit future comparison of female specimens and other investigations into the utility of the female genitalia for species identification in the Nannariini. The most common shape of the cyphopod receptacle in the *wilsoni* species group is small and triangular, but many modifications to this configuration are found, particularly in the Southern Appalachian Mountains species cluster.

The utility of female genitalic characters for the identification of genera or species in Polydesmida is not as well-investigated as is the characters of the male genitalia. However, female genitalic characters have been shown to be useful for species identification in some cases, such as with the genera *Euryurus* and *Eurymerodesmus* (Hoffman 1978a; Shelley 1990), and were used to form the defining characters of the subfamily Parafontariinae Hoffman, 1978 (Hoffman 1978b), now a tribe, Parafontariini (Means et al. 2021a). Other variations within the Xystodesmidae are known, such as in the genus *Cherokia* Chamberlin, 1949, which lacks a receptacle entirely (Hoffman 1960) and the genus *Pachydesmus* Cook, 1895, in which females have modified cyphopodal apertures and receptacles (Hoffman 1958). Other families of Polydesmida are also known to have modified female genitalia, such as the Chelodesmidae, with the species *Plectrogonodesmusgounellei* (Brölemann, 1902) having an enlarged epigyne and recurved receptacles with tubular projections (Brölemann 1902; Bouzan et al. 2019). In the Polydesmidae, *Pseudopolydesmus* Attems, 1898 has recently-identified cyphopod structures that may be useful for species identification (Zahnle et al. 2020).

### ﻿Biogeographical clusters

Two regional distributions were observed within the *wilsoni* species group: a cluster in the Central Appalachian Mountains, with species known from Kentucky, West Virginia, and Virginia; and another cluster in the Southern Appalachian Mountains, with species known from Georgia, North Carolina, South Carolina, and Tennessee (Fig. 2). These clusters are separated by a gap of approximately 140 km, in which no specimens of the *wilsoni* species group have been collected, despite repeated collecting efforts that revealed multiple species of the *minor* species group occurring in this area. Due to the presence of other *Nannaria* in this area and no lack of collecting, this gap was determined to be a reflection of the biological reality of the *wilsoni* species group, rather than an artifact of a lack of collecting effort. The gap extends from near Abingdon, Virginia south to the vicinity of Asheville, North Carolina, and no obvious biogeographical barriers exist to limit the distribution of each cluster, as evidenced by the presence of species from the *minor* species group. This distributional gap is not directly reflected in the phylogeny (i.e., there are not two distinct clades separating the biogeographical clusters), though generally species are more closely related to other species from their biogeographical cluster. Future collecting in the intervening area may clarify the exact boundaries of this gap. Despite the lack of obvious biogeographical barriers in this gap, there may be unknown abiotic factors that favor the presence of only the *minor* species group, which may be more adaptable to a wider variety of habitats, as exhibited by their presence west of the Appalachian Mountains and in the drier Ozark Mountains and intervening lowlands. Alternatively, although not mutually exclusive of adaptability to a wider variety of habitats, the *minor* species group may be older and therefore have had a longer period to disperse widely. There is also a possibility that species of the *wilsoni* group are rarer in this region and may eventually be found at lower population levels in unsampled habitats in the gap.

*Central Appalachian Mountains Biogeographical Cluster*. In the Central Appalachian Mountains Biogeographical Cluster, 15 species of the *wilsoni* group are known, and 10 of these species are newly described. In this cluster, species typically have a gradually curving acropodite or a straight acropodite with an abrupt bend distally. In the case of *N.marianae* sp. nov., the acropodite is bent at the midpoint. The acropodite anterior bend twist is generally absent or in the shape of a smoothly-undulating helix, except for *N.orycta* sp. nov., which is acutely twisted at the anterior bend of the acropodite. Interestingly, the boundaries of the distributions of four species in this cluster (*N.aenigma*, *N.cymontana* sp. nov., *N.ericacea*, and *N.wilsoni*) provide evidence that the New River acts as a geographical barrier for those taxa and has resulted in the vicariance of these species. Only one species, *N.aenigma*, occurs west of the New River, while the other three species all occur east of the river, and none of these species occur on both sides of the New River (Fig. 51). Similarly, *N.cymontana* sp. nov. appears to have parts of its northern distribution limited by the Roanoke River, and *N.ericacea*, *N.filicata* sp. nov., *N.lutra* sp. nov., *N.morrisoni*, *N.orycta* sp. nov., and *N.vellicata* sp. nov. are limited in portions of their distributions by the James River (Fig. 51).

*Southern Appalachian Mountains Biogeographical Cluster*. In the Southern Appalachian Mountains Biogeographical Cluster, nine species of the *wilsoni* species group are known, and seven of these species are newly described. In this cluster, all but one species have a straight acropodite with an abrupt bend distally, and only *N.lithographa* sp. nov. differs by having an acropodite bent at its midpoint. The acropodite anterior bend twist is typically acutely contorted and appears almost crimped or pinched, except in *N.rhododendra* sp. nov. and *N.lithographa* sp. nov., in which the twist is in the shape of a smoothly-undulating helix. Acropodite anterior bends with acute, crimped twists are most common in species of the Southern Appalachian Mountains Biogeographical Cluster. Only one species from the Central Appalachian Mountains Biogeographical Cluster, *N.orycta* sp. nov., has an acropodite anterior bend with this condition. In the Southern Appalachian Mountains Biogeographical Cluster, the acropodite tip is typically smooth and entire, although in a few species it is bifurcate. The cyphopod receptacle is typically modified in this cluster, rather than being small and triangular. The most unusual modification of the receptacle is observed in *N.antarctica* sp. nov. and *N.rhododendra* sp. nov., in which the receptacle is modified into a long, finger-like structure that projects outside of the cyphopod aperture and can be observed without dissecting the millipede.

### ﻿Molecular phylogenetics

The maximum likelihood phylogeny inferred with our analysis (Fig. 3) generally agrees with the morphology-based species concepts, with some exceptions. *Nannarialithographa* sp. nov. is sister to all other species in the *wilsoni* species group, an unsurprising result considering its divergent gonopod anatomy, with the presence of acropodite setae on up to three-quarters the length of the acropodite, and its spear-shaped prefemoral process attached distally on the acropodite, two morphological characters not present in any other species in the group. The low bootstrap support (average 45) for higher level relationships within the *wilsoni* species group phylogeny precludes discussion about which clades are most closely related, but in general the species in each biogeographical cluster are more closely related to each other than to species from the other geographic cluster. Most species clades from the Central Appalachian Mountains Biogeographical Cluster were highly supported by bootstrap values greater than 70, while few of the species clades from the Southern Appalachian Mountains Biogeographical Cluster had similar high support. Higher-level relationships within the *wilsoni* species group were not well supported, and some species, such as *N.rhododendra* sp. nov., *N.scutellaria*, and *N.vellicata* sp. nov., were recovered in multiple positions in the phylogeny. This may be due to lower quality genetic data, as indicated by longer branches in the phylogeny (e.g., *N.rhododendra* sp. nov. and *N.vellicata* sp. nov.), or possibly cryptic speciation (e.g., *N.scutellaria*).

The two widely separated groups of *N.scutellaria* recovered by the phylogeny make a strong case for cryptic speciation. The three specimens of *N.scutellaria* recovered as monophyletic (spare MPE03745 and MPE04346) were collected from the northern portion of its distribution, and includes a specimen (VTEC, MPE01474) collected at its type locality, Chimneys Picnic Area in Great Smoky Mountains National Park that matches closely with the gonopod morphology of the holotype (Causey 1942). The other specimens of *N.scutellaria* in this type-group vary in the size and position of the acropodite medial flange tooth and in the curve of the acropodite tip. The separate group of *N.scutellaria* does not include the type, and the species warrants further genetic analyses from a wider array of specimens to still determine whether *N.scutellaria* is composed of several species. Nonetheless, due to overall morphological similarity of these specimens they are treated as a single species. Within the *wilsoni* species group, this species is by far the most highly variable in its gonopod morphology. We included many specimens with variable gonopod morphology in *N.scutellaria*. Specimens ascribed to this species typically have a tooth-like acropodite medial flange, a triangular acropodite tip medial flange, and a prefemoral process that is quite wide basally. The gonopod characters may vary in the size of the medial flange, the distance between the tip medial flange and acropodite tip, the size and position of the tip medial flange, the direction the acropodite tip is pointed, and the distal curve of the acropodite. In some specimens, the acropodite tip medial flange is absent. A specimen collected near Nantahala, North Carolina (VMNH, NAN0369) even has the acropodite tip medial flange expanded and laminate, forming a concave leaf- or cup-like shape. The specimens recovered in the non-type *N.scutellaria* group were collected in Transylvania and Clay counties, North Carolina, at the southern edge of the species distribution in the Blue Ridge Mountains, and the collection of fresh material from the southern edge of this distribution could further elucidate the status of this species.

Inclusion of additional specimens of species from the Southern Appalachian Mountains Biogeographical Cluster could resolve some of the low support values recovered for those taxa, which generally had fewer specimens included in the analysis than species from the Central Appalachian Mountains Biogeographical Cluster. Despite being recovered at three separate positions in the phylogeny, no significant gonopod variation was observed in all examined specimens of *N.rhododendra* sp. nov. This situation is likely due to lesser quality genetic data for this species, rather than possible cryptic speciation, as may be the case for *N.scutellaria*.

While the evolutionary relationships between species in the *wilsoni* group remain unclear, this may be rectified in the future with analysis of additional genetic data. Morphology-based species delimitation based on gonopod shape in *Nannaria* has been proven useful in previous work on the genus (Means et al. 2021a, b), and we are confident the species delimited here will be supported by future genetic analysis that solves the previously-identified inconsistencies.

### ﻿Conservation

Much remains to be learned about the ecology of the *Nannariawilsoni* species group. Its species are typically limited to mesic deciduous forests or rhododendron coves in the Appalachian Mountains, with few exceptions. The Blue Ridge Mountains are home to many of these species, as are the Alleghany Mountains in the more northern parts of their distribution. More undescribed species in the genus surely await description, particularly in little-collected areas of northern Georgia, eastern Tennessee and Kentucky, and eastern West Virginia. The introduction of the forest pest *Adelgestsugae* Annand, 1924, the hemlock wooly adelgid (Hemiptera: Adelgidae), into the United States from Japan poses a threat to hemlock habitats commonly favored by *Nannaria* species. In some areas, this pest can kill up to 92% of hemlock trees (Rohr et al. 2009), which could greatly impact these millipedes if forests change from the formerly shady, cool hemlock forest microhabitats *Nannaria* prefers to drier habitats less amenable to their preferences. In addition to hemlock habitat loss, rhododendron dieback threatens to affect *Nannaria* populations in the Appalachian Mountains. Like hemlock, the presence of rhododendron provides the cool, shady microhabitats that *Nannaria* prefers, and its importance will increase with the loss of hemlock (Baird et al. 2014). Rhododendron dieback has been reported to affect *Rhododendronmaximum* L., which is widespread in the Appalachians, and appears to be caused by environmental stress that weakens the plants and allows opportunistic pests and pathogens to attack them (Baird et al. 2014; Brooks et al. 2019). Additionally, a fungal pathogen specializing on millipedes was recently found to infect the genus (Hodge et al. 2017), but little is known about its effects at a population level.

The small, highly endemic distributions of *Nannaria* are susceptible to habitat loss and fragmentation, a cause for concern as the hemlock wooly adelgid spreads and invades new areas of forest. Habitat loss from human development and invasive species’ effects on forests are the primary conservation threats for the *wilsoni* group, particularly due to the small distributions of many of its species. The distributions of some species are limited to small areas in only a few counties, or as in the case of *N.spalax* sp. nov., only one known locality. Due to the secretive habits of *Nannaria*, there is also a risk that microendemic undescribed species exist and may be driven to extinction before they are formally described.

## ﻿Conclusions

In this study, we accomplished three main objectives, we: (1) confirmed the monophyly of the *wilsoni* species group using molecular phylogenetics, (2) described new species of this species group, and (3) revised the taxonomy of the genus with an investigation of the natural history and ecology of the genus. The genus *Nannaria* has two large clades with species that are morphologically, genetically, and biogeographically separable from each other. We described 17 new species in the *wilsoni* species group, more than tripling the number of known species, from 7 to 24, and increased total species in the family Xystodesmidae to 539. Based on numerous collecting events, natural history data, and data from museum collections, we discovered that *Nannaria* prefers riparian habitats in rhododendron-hemlock cove forests and mesic deciduous forests in North America. Species in the *wilsoni* species group prefer mesic forests of the Appalachian Mountains, and are fossorial in their habits, being found buried in the soil or at the soil-leaf litter interface. The discovery of additional species diversity in the *wilsoni* species group and in the *minor* species group reveals the tribe Nannariini to be one of the most species-rich lineages within the Xystodesmidae, rivaling the species diversity of the Apheloriini. This may be due in part to continuous nonadaptive radiation of *Nannaria* and repeated vicariance of their distributions by the physiographic history of the Appalachian Mountains in eastern North America. Nonadaptive radiation has been previously suggested to occur in the family Xystodesmidae (Marek and Moore 2015, Means et al. 2021a). Additional undescribed species of Nannariini undoubtedly exist in eastern North America, and will be revealed by further fieldwork in undersampled regions of the Central and Southern Appalachian Mountains. Before this study, the true species diversity of the *wilsoni* species group was unknown, particularly in the Southern Appalachian Mountains. The results presented in this study, along with those of Means et al. (2021a, b) have vastly expanded our knowledge of the tribe, and provide a solid taxonomic foundation for further study of this group. The genus *Nannaria* now contains 78 species (Table 1), making it the largest genus in the Xystodesmidae.

## Supplementary Material

XML Treatment for Nannariini

XML Treatment for Nannaria

XML Treatment for Nannaria acroteria

XML Treatment for Nannaria aenigma

XML Treatment for Nannaria amicalola

XML Treatment for Nannaria antarctica

XML Treatment for Nannaria austricola

XML Treatment for Nannaria cymontana

XML Treatment for Nannaria ericacea

XML Treatment for Nannaria filicata

XML Treatment for Nannaria liriodendra

XML Treatment for Nannaria lithographa

XML Treatment for Nannaria lutra

XML Treatment for Nannaria marianae

XML Treatment for Nannaria morrisoni

XML Treatment for Nannaria nessa

XML Treatment for Nannaria orycta

XML Treatment for Nannaria paraptoma

XML Treatment for Nannaria rhododendra

XML Treatment for Nannaria scutellaria

XML Treatment for Nannaria shenandoa

XML Treatment for Nannaria spalax

XML Treatment for Nannaria spiralis

XML Treatment for Nannaria swiftae

XML Treatment for Nannaria vellicata

XML Treatment for Nannaria wilsoni

## Supplementary materials

Supplementary material 1 *Nannariawilsoni* species group specimens examined Data type Occurrences. Explanation note A Darwin Core Standard spreadsheet of the *Nannariawilsoni* species group specimens examined. https://binary.pensoft.net/file/673005This dataset is made available under the Open Database License (http://opendatacommons.org/licenses/odbl/1.0/). The Open Database License (ODbL) is a license agreement intended to allow users to freely share, modify, and use this Dataset while maintaining this same freedom for others, provided that the original source and author(s) are credited.Derek A. Hennen, Jackson C. Means, Paul E. Marek

Supplementary material 2 *Nannariawilsoni* species group morphology scoring matrix Data type Morphological scoring matrix. Explanation note A spreadsheet with the morphological characters scored for each species in the *Nannariawilsoni* group. https://binary.pensoft.net/file/673006This dataset is made available under the Open Database License (http://opendatacommons.org/licenses/odbl/1.0/). The Open Database License (ODbL) is a license agreement intended to allow users to freely share, modify, and use this Dataset while maintaining this same freedom for others, provided that the original source and author(s) are credited.Derek A. Hennen, Jackson C. Means, Paul E. Marek

Supplementary material 3 Key to *Nannariawilsoni* species group morphological characters Data type Key to morphological matrix. Explanation note Key listing the qualitative morphological characters used for descriptions and diagnoses. https://binary.pensoft.net/file/673007This dataset is made available under the Open Database License (http://opendatacommons.org/licenses/odbl/1.0/). The Open Database License (ODbL) is a license agreement intended to allow users to freely share, modify, and use this Dataset while maintaining this same freedom for others, provided that the original source and author(s) are credited.Derek A. Hennen, Jackson C. Means, Paul E. Marek

Supplementary material 4 Amplification procedures for genes Data type Methodology. Explanation note Summary of amplification procedures for the newly developed genes used in this project. https://binary.pensoft.net/file/673008This dataset is made available under the Open Database License (http://opendatacommons.org/licenses/odbl/1.0/). The Open Database License (ODbL) is a license agreement intended to allow users to freely share, modify, and use this Dataset while maintaining this same freedom for others, provided that the original source and author(s) are credited.Derek A. Hennen, Jackson C. Means, Paul E. Marek

Supplementary material 5 List of taxa and Genbank numbers Data type Genetic. List of taxa used in molecular analyses, organized alphabetically by genus and species. Acc# refer to the NCBI Genbank database. All specimens available from the corresponding author by request and stored in the Virginia Tech Insect Collection, Blacksburg, Virginia, USA. https://binary.pensoft.net/file/673009This dataset is made available under the Open Database License (http://opendatacommons.org/licenses/odbl/1.0/). The Open Database License (ODbL) is a license agreement intended to allow users to freely share, modify, and use this Dataset while maintaining this same freedom for others, provided that the original source and author(s) are credited.Derek A. Hennen, Jackson C. Means, Paul E. Marek

Supplementary material 6 Molecular phylogeny of *Nannaria* and outgroups Data type Phylogenetic. Explanation note Full maximum likelihood phylogeny of *Nannaria* and outgroups used in the analysis. Phylogeny estimated from the gene regions 16S, COI, EF1a, 28S, RPB1, and fbox. Branches colored according to clades: *Oenomaeapulchella* (green), Eurymerodesmini (magenta), Euryurini (red), *Nannariaminor* group (brown), and *Nannariawilsoni* group (blue). Bootstrap support values > 70 indicated by asterisks (*). https://binary.pensoft.net/file/673010This dataset is made available under the Open Database License (http://opendatacommons.org/licenses/odbl/1.0/). The Open Database License (ODbL) is a license agreement intended to allow users to freely share, modify, and use this Dataset while maintaining this same freedom for others, provided that the original source and author(s) are credited.Derek A. Hennen, Jackson C. Means, Paul E. Marek

Supplementary material 7 Individual gene trees Data type Phylogenetic. Explanation note Maximum likelihood phylogenies of *Nannaria* and outgroups used in the analysis. Phylogenies estimated from the gene regions 16S(A), 28s(B), COI(C), EF1a(D), fbox(E), and RPB1(F). Branches colored according to clades: *Oenomaeapulchella* (green), Eurymerodesmini (magenta), Euryurini (red), *Nannariaminor* group (brown), and *Nannariawilsoni* group (blue). Bootstrap support values > 70 indicated by asterisks (*). https://binary.pensoft.net/file/673011This dataset is made available under the Open Database License (http://opendatacommons.org/licenses/odbl/1.0/). The Open Database License (ODbL) is a license agreement intended to allow users to freely share, modify, and use this Dataset while maintaining this same freedom for others, provided that the original source and author(s) are credited.Derek A. Hennen, Jackson C. Means, Paul E. Marek
