# Supplementary material for: ﻿A revision of the wilsoni species group in the millipede genus Nannaria Chamberlin, 1918 (Diplopoda, Polydesmida, Xystodesmidae)
Source: Zookeys. 2022 Apr 15;1096:17–118. doi: 10.3897/zookeys.1096.73485 (PMC9033750; doi:10.3897/zookeys.1096.73485)
Supplement: Supplementary material 3 — Key to Nannariawilsoni species group morphological characters [file zookeys-1096-017-s003.docx]

**Supplementary Material 3.** Qualitative morphological characters used for descriptions and diagnoses, both binary and multistate. 1–22, 51-53, male gonopodal characters; 23-27, 48-50, female cyphopodal characters; 28–47, 54, male somatic characters.

#1. Gonopods

1. do not cross in situ

2. cross in situ

#2. Prefemoral basal spine, presence

1. absent

2. present

#3. Prefemoral basal spine

1. absent

2. separate from prefemoral process for some part of length

3. entire length fused with prefemoral process

#4. Prefemoral basal spine, size

1. absent

2. large, pronounced

3. small, reduced

#5. Prefemoral basal spine, shape

1. absent

2. pointed, sharp

3. pointed, blunt

4. rectangular, shelf-like

5. reduced to rounded bulge

#6. Prefemoral process arising from

1. base of acropodite

2. top of prefemoral spine

3. prefemoral spine, dorsomedially

4. prefemoral spine, ventromedially

5. absent

6. midway up the acropodite

#7.Prefemoral process shape

1. acicular

2. laminate

3. stout

4. sinuous

5. simple

6. absent

7. sinuous taper

#8. Prefemoral process curve, when viewed anteriorly

1. straight

2. medially

3. laterally

4. absent

5. ventrally

#9. Prefemoral process crosses acropodite, when viewed anteriorly

1. does not cross

2. crosses dorsolaterally

3. crosses ventrolaterally

#10. Prefemoral process tip directed

1. ventrally

2. cephalically

3. medially

4. dorsally

5. absent

6. laterally

#11. Gonopodal basal zone lateral bulge, presence

1. absent

2. present

#12. Basal zone width, when viewed anteriorly

1. wider than space between acropodite and prefemoral process at greatest divide

2. subequal to space between acropodite and prefemoral process at greatest divide

3. thinner than space between acropodite and prefemoral process at greatest divide

#13. Basal zone height, when viewed anteriorly

1. < half length of acropodite

2. subequal to half length of acropodite

3. > half length of acropodite

#14. Acropodite arc

1. straight

2. straight, with abrupt bend at tip

3. gradual curve

4. sinuous

5. straight, with bend at midpoint

#15. Acropodite cingulum, presence

1. absent

2. present

#16. Acropodite, swelling

1. swollen before apex

2. not swollen before apex

#17. Acropodite medial flange, shape

1. absent

2. lobed

3. laminate

4. tooth-like

#18. Acropodite tip medial flange, shape

1. absent

2. triangular

3. lobed

4. laminate

#19. Acropodite tip lateral flange, shape

1. absent

2. triangular

3. lobed

4. hooked

5. laminate

#20. Acropodite tip directed, when viewed anteriorly

1. medially

2. caudally

3. laterally

4. cephalically

5. dorsally

6. ventrally

#21. Gonopodal distal zone, length

1. absent

2. short, less than 0.4x length of acropodite

3. long, greater than or equal to 0.5x length of acropodite

#22. Gonopodal tip, shape

1. sharp

2. blunt

#23. Cyphopodal receptacle, presence

1. absent

2. present

#24. Cyphopodal receptacle, size at widest part

1. absent

2. shorter than prefemur length

3. subequal to prefemur length

4. wider than prefemur length

#25. Cyphopodal valves, symmetry

1. symmetric

2. asymmetric

#26. Cyphopodal valves, orientation

1. ventrally

2. anteroventrally

3. posteroventrally

4. twisted posterior

5. laterally

#27. Cyphopodal receptacle cuticle, surface

1. absent

2. smooth

3. sculptured

#28. Gnathochilarium lateral emargination, presence

1. absent

2. present

#29. Antennomere one distal cuticle, conformation

1. cylindrical, not wrapped around cones

2. wrapped around cones

#30. Collum ridges, presence

1. absent

2. present

#31. Caudolateral corners, paranota I-X, shape

1. acute, projecting caudally

2. rounded

#32. Caudolateral corners, paranota I-XIX, shape

1. acute, projecting caudally on all segments

2. rounded on segments I-X only

3. rounded on segments I-XIX

#33. Metatergal pores, metatergites IX+X, presence

1. absent

2. present

#34. Metatergal linear bump-pores, presence

1. absent

2. present

#35. Lateral wrinkles, tergites IX+X

1. tightly wrinkled

2. loosely wrinkled

#36. Repugnatorial pores, paranota IX+X, orientation

1. laterally

2. dorsally

#37. Metatergal dorsal slits, presence

1. absent

2. present

#38. Metatergal dorsal microsculpture, mesh shape

1. isodiametric

2. anisodiametric

#39. Paranota-dorsum, segments IX+X, angle

1. 130 degrees

2. 180 degrees

#40. Paranotal segments IX+X, width

1. thin

2. thick

#41. Gonapophyses, shape

1. cylinder-shaped

2. goblet-shaped

#42. Sternal knobs, 4th leg pair, presence

1. absent

2. present

#43. Pleural process, segments IX+X, presence

1. absent

2. present

#44. Sternal triangular spines, segments IX+X, presence

1. absent

2. present

#45. Sternal median bulge, segments IX+X, presence

1. absent

2. present

#46. Setae, sterna IX+X, presence

1. absent

2. present

#47. Ventral excavation, sterna IX-X, presence

1. absent

2. present

#48. Female leg 2 coxa, shape

1. normal, not expanded

2. laterally expanded, covering cyphopod bursa

#49. Cyphopodal receptacle length, size compared to valves

1. absent

2. < 12 as large as the valves

3. 12 as large as the valves

4. 34 as large as the valves

5. subequal in size to the valves

6. larger than valves

#50. Cyphopodal receptacle, shape

1. absent

2. triangular

3. capsule-shaped, with rounded tip

4. finger-like projection

5. laminate

6. elongated club

7. enlarged triangular hood, covering valves

8. quadrate, recurved distally

9. sinuous S-shaped, with blunted end

10. dome-shaped, slightly tapered distally

#51. Gonopod acropodite basomedial process, presence

1. absent

2. present

#52. Gonopod acropodite anterior bend twist, presence

1. absent

2. shape of a smoothly-undulating helix

3. acutely bent, appearing crimped

#53. Gonopod acropodite tip, shape

1. single, smooth, entire branch

2. bifurcate

#54. Leg 10 prefemur spine, length

1. < than half as long as femur

2. half as long as femur

3. > half as long as femur

4. subequal in size to femur
