# Supplementary material for: ﻿A revision of the wilsoni species group in the millipede genus Nannaria Chamberlin, 1918 (Diplopoda, Polydesmida, Xystodesmidae)
Source: Zookeys. 2022 Apr 15;1096:17–118. doi: 10.3897/zookeys.1096.73485 (PMC9033750; doi:10.3897/zookeys.1096.73485)
Supplement: Supplementary material 4 — Amplification procedures for genes [file zookeys-1096-017-s004.docx]

**Supplementary Material 4**

**Amplification Procedures**

DNA amplification procedures were as follows. Only the procedures for the newly-designed primers are given. For COI, EF1-a, and 28S procedures, see Means & Marek (2017). The following master mixes [with volumes calculated for 6 samples plus a negative and a positive control (49 µL)] and thermocycler protocols were used.

*Nannaria 16S (5’ end)*: master mix – 290.25 µL water, 45 µL dNTP’s, 45 µL 10X buffer, 22.5 µL LR-J-Nan16S (forward primer, CGCTGTTATCCCTACAGTAA), 22.5 µL SR-N-Nan16S (reverse primer, CGATGAATAACGAGCTATAG), 13.5 µL DMSO and 2.25 µL Taq; thermocycler protocol - Start at 95 °C for 120 seconds, then 29 cycles of denaturing (94 °C for 30 seconds), annealing (52 °C for 30 seconds) and extension (72 °C for 60 seconds) with a 2 minute final extension at 72 °C (Marek and Bond 2006; 2007; Marek and Moore 2015).

*Nannaria fbox (5’ end)*: master mix – 290.25 µL water, 45 µL dNTP’s, 45 µL 10X buffer, 22.5 µL J-Nanfbox (forward primer, GGACGAGCTGCTCAGGTGTG), 22.5 µL N-Nanfbox (reverse primer, CCTGCAATATGAGACGCTCC), 13.5 µL DMSO and 2.25 µL Taq; thermocycler protocol - Start at 95 °C for 120 seconds, then 29 cycles of denaturing (94 °C for 30 seconds), annealing (52 °C for 30 seconds) and extension (72 °C for 60 seconds) with a 2 minute final extension at 72 °C (Marek and Bond 2006; 2007; Marek and Moore 2015).

*Nannaria RNApol2 (5’ end)*: master mix – 290.25 µL water, 45 µL dNTP’s, 45 µL 10X buffer, 22.5 µL J-NanRNAPol2 (forward primer, CGTTGGGCAATTAATCCAGC), 22.5 µL N-NanRNAPol2 (reverse primer, CGAACTCCTGATCTTCTGC), 13.5 µL DMSO and 2.25 µL Taq; thermocycler protocol - Start at 95 °C for 120 seconds, then 29 cycles of denaturing (94 °C for 30 seconds), annealing (52 °C for 30 seconds) and extension (72 °C for 60 seconds) with a 2 minute final extension at 72 °C (Marek and Bond 2006; 2007; Marek and Moore 2015).

**References**

Marek PE, Bond JE (2006) Phylogenetic systematics of the colorful, cyanide-producing millipedes of Appalachia (Polydesmida, Xystodesmidae, Apheloriini) using a total evidence Bayesian approach. Molecular Phylogenetics and Evolution 41: 704–729. <https://doi.org/10.1016/j.ympev.2006.05.043>

Marek PE, Bond JE (2007) A reassessment of apheloriine millipede phylogeny: additional taxa, Bayesian inference, and direct optimization (Polydesmida: Xystodesmidae). Zootaxa 1610: 27–39. <https://doi.org/10.11646/zootaxa.1610.1.2>

Marek PE, Moore W (2015) Discovery of a glowing millipede in California and the gradual evolution of bioluminescence in Diplopoda. Proceedings of the National Academy of Sciences. <https://doi.org/10.1073/pnas.1500014112>

Means JC, Marek PE (2017) Is geography an accurate predictor of evolutionary history in the millipede family Xystodesmidae? PeerJ 5. <https://doi.org/10.7717/peerj.3854>
