# Supplementary material for: ﻿A revision of the wilsoni species group in the millipede genus Nannaria Chamberlin, 1918 (Diplopoda, Polydesmida, Xystodesmidae)
Source: Zookeys. 2022 Apr 15;1096:17–118. doi: 10.3897/zookeys.1096.73485 (PMC9033750; doi:10.3897/zookeys.1096.73485)
Supplement: Supplementary material 5 — List of taxa and Genbank numbers [file zookeys-1096-017-s005.docx]

**Supplementary Material 5.** List of taxa used in molecular analyses, organized alphabetically by genus and species. Acc# refer to the NCBI Genbank database. All specimens available from the corresponding author by request and stored in the Virginia Tech Insect Collection, Blacksburg, Virginia, USA.

| **Taxon** | **Spc#** | **Latitude** | **Longitude** | **State, County** | **Acc# 16S** | **Acc# EF1a** | **Acc# 28S** | **Acc# COI** | **Acc# RPB1** | **ACC# fbox** |
| --- | --- | --- | --- | --- | --- | --- | --- | --- | --- | --- |
| **Xystodesmidae** |  |  |  |  |  |  |  |  |  |  |
| **Eurymerodesmini** |  |  |  |  |  |  |  |  |  |  |
| *Eurymerodesmus oliphantus* | MPE02825 | 37.4964 | -89.3572 | Illinois, Union | **MN658236** | **MN719679** | **-** | **MN699746** | **-** | **-** |
| *Eurymerodesmus varius* | MPE03297 | 31.4351 | -86.0541 | Alabama, Coffee | **MN658237** | **MN719683** | **-** | **MN699747** | **-** | **-** |
|  |  |  |  |  |  |  |  |  |  |  |
| **Euryurini** |  |  |  |  |  |  |  |  |  |  |
| *Euryurus cingulatus* | MPE01341 | 34.1017 | -87.3203 | Alabama, Winston | **MT497579** | **MN719658** | **-** | **MN699748** | **-** | **-** |
| *Euryurus erythropygos* | MPE04786 | 34.139 | -82.3145 | South Carolina, Abbeville | **MN658215** | **MN719707** | **-** | **MN699722** | **-** | **-** |
| *Euryurus leachii* | MPE04740 | 39.542 | -82.5755 | Ohio, Hocking | **MN817795** | **MN719703** | **MT504410** | **MN699749** | **-** | **-** |
| *Euryurus orestes* | MPE01309 | 34.5593 | -84.2496 | Georgia, Dawson | **MN658238** | **MN719655** | **-** | **MN699750** | **-** | **-** |
|  |  |  |  |  |  |  |  |  |  |  |
| **Nannariini** |  |  |  |  |  |  |  |  |  |  |
| *Nannaria* | MPE00585 | 38.655 | -80.0713 | Virginia, Randolph | **MT302702** | **MT319927** | **-** | **MT311383** | **MT503360** | **MT349984** |
| *Nannaria* | MPE00792 | 37.4035 | -80.3414 | Virginia, Craig | **MT302710** | **MT319928** | **MT319836** | **MT311384** | **-** | **MT349985** |
| *Nannaria* | MPE01690 | 39.4913 | -82.5797 | Ohio, Hocking | **MT302670** | **MT319936** | **-** | **-** | **MT503382** | **MT350009** |
| *Nannaria* | MPE02117 | 35.8366 | -78.7623 | North Carolina, Wake | **-** | **MT319948** | **MT319854** | **MT311407** | **-** | **-** |
| *Nannaria* | MPE02242 | 38.1802 | -81.8384 | West Virginia, Boone | **MT302675** | **MT319949** | **MT319858** | **MT311412** | **MT503401** | **MT350029** |
| *Nannaria* | MPE02801 | 37.4667 | -83.9164 | Kentucky, Jackson | **MT302661** | **MT319959** | **MT319869** | **MT311424** | **MT503419** | **MT350047** |
| *Nannaria* | MPE02831 | 37.2321 | -80.0867 | Virginia, Roanoke | **MT302648** | **-** | **-** | **MT311425** | **MT503420** | **MT350048** |
| *Nannaria* | MPE02832 | 38.239 | -81.5736 | West Virginia, Kanawha | - | **MT515415** | - | **MT491276** | - | - |
| *Nannaria* | MPE02879 | 36.3973 | -81.0517 | North Carolina, Wilkes | **MT302715** | **-** | **MT319871** | **MT311427** | **MT503422** | **MT350050** |
| *Nannaria* | MPE02891 | 36.5605 | -80.7469 | North Carolina, Surry | **MT302722** | **-** | **MT319872** | **MT311428** | **MT503423** | **MT350051** |
| *Nannaria* | MPE02937 | 36.5022 | -82.4825 | Tennessee, Sullivan | **MT302692** | **-** | **-** | **MT311429** | **MT503425** | **MT350053** |
| *Nannaria* | MPE02983 | 36.7941 | -82.0904 | Virginia, Washington | **-** | **-** | **MT319874** | **MT311430** | **MT503426** | **MT350054** |
| *Nannaria* | MPE03145 | 37.8915 | -84.3972 | Kentucky, Fayette | **MT302671** | **MT319968** | **MT319881** | **-** | **MT503432** | **MT350061** |
| *Nannaria* | MPE03469 | 37.3452 | -80.9105 | West Virginia, Mercer | **MT302689** | **-** | **MT319888** | **MT311437** | **MT503439** | **MT350068** |
| *Nannaria* | MPE03639 | 38.0658 | -77.3803 | Virginia, Caroline | **MT302711** | **MT319977** | **MT319893** | **MT311441** | **MT503443** | **MT350074** |
| *Nannaria* | MPE03655 | 36.1913 | -81.6023 | North Carolina, Watauga | **MT302657** | **MT319979** | **-** | **MT311443** | **MT503445** | **-** |
| *Nannaria* | MPE03674 | 38.3071 | -79.7699 | Virginia, Highland | **MT302718** | **-** | **MT319895** | **MT311444** | **MT503446** | **MT350076** |
| *Nannaria* | MPE04027 | 36.7521 | -80.6079 | Virginia, Carroll | **MT302704** | **-** | **-** | **MT311447** | **-** | **MT350079** |
| *Nannaria* | MPE04051 | 36.3897 | -82.3677 | Tennessee, Washington | **MT302682** | **-** | **MT319898** | **MT311449** | **MT503450** | **MT350081** |
| *Nannaria* | MPE04087 | 37.3795 | -80.2503 | Virginia, Craig | **MT302709** | **-** | **-** | **MT311452** | **MT503452** | **MT350084** |
| *Nannaria* | MPE04175 | 36.4329 | -82.0724 | Tennessee, Carter | **MT302713** | **-** | **MT319907** | **MT311457** | **MT503458** | **MT350091** |
| *Nannaria* | SPC000689 | 38.119 | -80.154 | West Virginia, Pocahontas | **MT302697** | **-** | **-** | **MT311459** | **MT503462** | **MT350096** |
| *Nannaria acroteria* sp. nov. | MPE02500 | 37.4493 | -80.5201 | Virginia, Giles | **MT302729** | **-** | **MT319865** | **MT311419** | **MT503410** | **MT350038** |
| *Nannaria aenigma* | MPE00002 | 36.9859 | -81.4076 | Virginia, Bland | **MT497556** | **MT515381** | **MT504364** | **-** | **MT665900** | **MT503639** |
| *Nannaria aenigma* | MPE00006 | 37.0593 | -81.2906 | Virginia, Bland | **MT497557** | **MT515382** | **-** | **MT491244** | **MT665901** | **MT503640** |
| *Nannaria aenigma* | MPE00072 | 36.7067 | -81.6028 | Virginia, Smyth | **MT497560** | **-** | **MT504366** | **MT491247** | **MT665903** | **MT503643** |
| *Nannaria aenigma* | MPE00172 | 36.9112 | -81.5317 | Virginia, Smyth | **MT497562** | **-** | **-** | **MT491249** | **MT665905** | **MT503645** |
| *Nannaria aenigma* | MPE00268 | 37.0148 | -81.4101 | Virginia, Tazewell | **MT302733** | **MT319923** | **MT319832** | **-** | **MT503355** | **MT349977** |
| *Nannaria aenigma* | MPE00350 | 37.1018 | -80.8867 | Virginia, Pulaski | **MT497566** | **MT515388** | **MT504370** | **-** | **-** | **MT503648** |
| *Nannaria aenigma* | MPE00409 | 37.073 | -80.873 | Virginia, Pulaski | **MT497567** | **-** | **-** | **MT491252** | **MT665907** | **MT503649** |
| *Nannaria aenigma* | MPE00551 | 36.9199 | -81.4448 | Virginia, Bland | **MT497570** | **MT515391** | - | - | **MT665910** | - |
| *Nannaria aenigma* | MPE00576 | 37.0195 | -81.1355 | Virginia, Wythe | **MT497571** | **MT515392** | - | **MT491255** | **MT665911** | **MT503652** |
| *Nannaria aenigma* | MPE00658 | 37.0223 | -81.2048 | Virginia, Wythe | **MT497572** | **-** | **MT504373** | **MT491256** | **MT665912** | **-** |
| *Nannaria aenigma* | MPE00857 | 37.0248 | -80.7842 | Virginia, Pulaski | **MT497577** | **MT515397** | **MT504378** | **MT491260** | **MT665917** | **MT503657** |
| *Nannaria aenigma* | MPE01008 | 37.1924 | -81.1703 | Virginia, Bland | **MN817805** | **MN719649** | **MN817838** | **MN699769** | **MT503367** | **MT349992** |
| *Nannaria aenigma* | MPE01055 | 37.0352 | -81.1055 | Virginia, Bland | **-** | **MT515398** | **-** | **MT491261** | **MT665918** | **MT503658** |
| *Nannaria aenigma* | MPE01060 | 37.0493 | -81.1161 | Virginia, Bland | **MT302737** | **-** | **MT319841** | **MT311389** | **MT503369** | **MT349994** |
| *Nannaria aenigma* | MPE01648 | 36.9477 | -81.8242 | Virginia, Washington | **MT302734** | **MT319934** | **MT504382** | **MT311396** | **MT503381** | **MT350007** |
| *Nannaria aenigma* | MPE02106 | 36.8304 | -81.9593 | Virginia, Washington | **MT302735** | **MT319942** | **MT319849** | **MT311403** | **MT503390** | **MT350018** |
| *Nannaria aenigma* | MPE02415 | 36.9526 | -81.3247 | Virginia, Wythe | **MT497585** | **MT515406** | - | **MT491269** | **MT665926** | **MT503665** |
| *Nannaria aenigma* | MPE02632 | 36.7454 | -81.4259 | Virginia, Smyth | **MT497594** | **-** | **MT504396** | **MT491275** | **MT665935** | **MT503674** |
| *Nannaria aenigma* | MPE03701 | 37.0329 | -81.1043 | Virginia, Bland | **MT497599** | **MT515418** | **MT504397** | **MT491281** | **MT665939** | **MT503677** |
| *Nannaria aenigma* | MPE03709 | 37.0157 | -81.246 | Virginia, Wythe | **MT497600** | **MT515419** | **MT504398** | **MT491282** | **MT665940** | **MT503678** |
| *Nannaria alpina* | MPE03150 | 36.9156 | -84.5182 | Kentucky, Pulaski | **MT302658** | **-** | **MT319882** | **MT311436** | **MT503433** | **MT350062** |
| *Nannaria ambulatrix* | MPE00178 | 36.9112 | -81.5317 | Virginia, Smyth | **-** | **-** | **MT319826** | **MT311375** | **MT503349** | **MT349971** |
| *Nannaria amicalola* sp. nov. | MPE01230 | 34.5753 | -84.1757 | Georgia, Lumpkin | **MT302751** | **-** | **-** | **MT311393** | **MT503374** | **MT350000** |
| *Nannaria antarctica* sp. nov. | MPE03311 | 35.1241 | -83.5392 | North Carolina, Macon | **MT497597** | **MT319971** | **-** | **MT319885** | **MT503436** | **MT350065** |
| *Nannaria antarctica* sp. nov. | MPE03317 | 34.8364 | -83.771 | Georgia, Towns | **MT302752** | **MT319972** | **-** | **MT319886** | **MT503437** | **MT350066** |
| *Nannaria antarctica* sp. nov. | MPE04608 | 34.7701 | -83.9163 | Georgia, Union | **MT497613** | **MT515432** | **-** | **MT491295** | **MT665952** | **MT503692** |
| *Nannaria antarctica* sp. nov. | SPC000367 | 35.0546 | -83.432 | North Carolina, Macon | **MT497615** | **MT515434** | **MT504411** | **MT491297** | **MT665954** | **MT503694** |
| *Nannaria asta* | MPE02419 | 36.9586 | -81.3189 | Virginia, Wythe | **-** | **MT319952** | **MT319862** | **MT311416** | **MT503407** | **MT350035** |
| *Nannaria austricola* | SPC000352 | 35.0633 | -83.4368 | North Carolina, Macon | **MT302749** | **MT319985** | **MT319909** | **-** | **MT503460** | **MT350093** |
| *Nannaria blackmountainensis* | SPC000652 | 36.7328 | -83.2216 | Kentucky, Harlan | **MT302660** | **-** | **MT319910** | **MT311458** | **MT503461** | **MT350094** |
| *Nannaria blackmountainensis* | SPC001090 | 36.9156 | -82.903 | Kentucky, Harlan | **MT302659** | **-** | **MT319912** | **MT311462** | **MT503465** | **MT350098** |
| *Nannaria bobmareki* | MMC0138 | 36.872 | -82.4847 | Virginia, Scott | **MT302685** | **-** | **-** | **MT311368** | **MT503343** | **MT349964** |
| *Nannaria bobmareki* | SPC001019 | 36.9364 | -83.3729 | Kentucky, Leslie | **MT302668** | **MT319988** | **MT319911** | **MT311461** | **MT503464** | **MT350097** |
| *Nannaria botrydium* | MPE01009 | 37.193 | -81.1827 | Virginia, Bland | **MT302687** | **MT319929** | **-** | **MT311388** | **MT503368** | **MT349993** |
| *Nannaria breweri* | MPE02191 | 36.2167 | -83.4055 | Tennessee, Hamblen | **MT302683** | **-** | **MT319856** | **MT311410** | **MT503399** | **MT350027** |
| *Nannaria breweri* | SPC001167 | 36.216 | -83.4057 | Tennessee, Hamblen | **MT302684** | **MT319989** | **-** | **MT311463** | **MT503466** | **MT350099** |
| *Nannaria castanea* | MPE02789 | 34.8051 | -88.3063 | Mississippi, Tishomingo | **MN817796** | **MN719677** | **MN817829** | **MN699760** | **MT503416** | **MT350044** |
| *Nannaria castanea* | MPE02797 | 36.4307 | -93.7576 | Arkansas, Carroll | **MN817797** | **MN719678** | **MN817831** | **MN699761** | **MT503417** | **MT350045** |
| *Nannaria castra* | MPE03470 | 37.5147 | -81.1297 | West Virginia, Mercer | **MT302705** | **-** | **MT319889** | **-** | **-** | **MT350069** |
| *Nannaria caverna* | MPE03139 | 38.3738 | -83.1142 | Kentucky, Carter | **MT302653** | **MT319967** | **MT319880** | **MT311435** | **-** | **MT350060** |
| *Nannaria cingulata* | MPE01881 | 39.0717 | -77.912 | Virginia, Clarke | **MT302698** | **MT319939** | **-** | **MT311399** | **MT503385** | **MT350013** |
| *Nannaria cingulata* | MPE02324 | 38.9332 | -78.3207 | Virginia, Shenandoah | **MT302699** | **MT319950** | **MT319860** | **MT311414** | **MT503403** | **MT350031** |
| *Nannaria cryomaia* | MPE02642 | 36.132 | -84.4978 | Tennessee, Morgan | **-** | **MT319957** | **-** | **-** | **MT503414** | **MT350042** |
| *Nannaria cymontana* sp. nov. | MPE00071 | 36.7716 | -80.4047 | Virginia, Patrick | **MT497559** | **MT515384** | **-** | **MT491246** | **MT665902** | **MT503642** |
| *Nannaria cymontana* sp. nov. | MPE00200 | 36.776 | -80.5446 | Virginia, Carroll | **MT497563** | - | - | - | - | - |
| *Nannaria cymontana* sp. nov. | MPE00205 | 36.776 | -80.5446 | Virginia, Carroll | **MT497564** | **MT515386** | **MT504367** | **MT491250** | **MT665906** | **MT503646** |
| *Nannaria cymontana* sp. nov. | MPE00234 | 36.9662 | -80.4179 | Virginia, Montgomery | **MT302731** | **MT319921** | **MT319829** | **MT311378** | **MT503352** | **MT349974** |
| *Nannaria cymontana* sp. nov. | MPE00460 | 36.7856 | -80.3726 | Virginia, Floyd | **MT497568** | **MT515389** | **MT504371** | **MT491253** | **MT665908** | **MT503650** |
| *Nannaria cymontana* sp. nov. | MPE00488 | 36.7437 | -80.4097 | Virginia, Floyd | **MT497569** | **MT515390** | **MT504372** | **MT491254** | **MT665909** | **MT503651** |
| *Nannaria cymontana* sp. nov. | MPE00720 | 36.7796 | -80.3984 | Virginia, Floyd | **MT497573** | **MT515393** | **MT504374** | **MT491257** | **MT665913** | **MT503653** |
| *Nannaria cymontana* sp. nov. | MPE00822 | 36.8132 | -80.3495 | Virginia, Floyd | **MT497575** | **MT515395** | **MT504376** | **-** | **MT665915** | **MT503655** |
| *Nannaria cymontana* sp. nov. | MPE02059 | 36.6866 | -80.4415 | Virginia, Patrick | **MT302732** | **MT319940** | **MT319847** | **MT311400** | **MT503387** | **MT350015** |
| *Nannaria cymontana* sp. nov. | MPE02495 | 37.1369 | -80.1108 | Virginia, Roanoke | **MT497589** | **MT515410** | - | - | **MT665930** | **MT503669** |
| *Nannaria cymontana* sp. nov. | MPE02513 | 36.7825 | -80.3997 | Virginia, Floyd | **MT497590** | **MT515411** | **MT504393** | **MT491272** | **MT665931** | **MT503670** |
| *Nannaria cymontana* sp. nov. | MPE02542 | 36.7505 | -80.405 | Virginia, Floyd | **MT497591** | **MT515412** | - | - | **MT665932** | **MT503671** |
| *Nannaria daptria* | MPE04156 | 35.9777 | -82.8478 | Tennessee, Greene | **MT302681** | **-** | **MT319906** | **-** | **MT503457** | **MT350090** |
| *Nannaria dilatata* | MPE02788 | 35.5914 | -86.7029 | Tennessee, Marshall | **-** | **MN719676** | **MN817830** | **MN699762** | **MT503415** | **MT350043** |
| *Nannaria domestica* | MPE00305 | 36.1386 | -81.6694 | North Carolina, Watauga | **MN817799** | **MN719647** | **MN817832** | **MN699763** | **MT503357** | **MT349980** |
| *Nannaria ericacea* | MPE00040 | 37.22 | -80.4156 | Virginia, Montgomery | **MT497558** | **MT515383** | **MT504365** | **MT491245** | **-** | **MT503641** |
| *Nannaria ericacea* | MPE00226 | 37.2506 | -80.461 | Virginia, Montgomery | **MT497565** | **MT515387** | **MT504368** | **MT491251** | **-** | **MT503647** |
| *Nannaria ericacea* | MPE00276 | 37.1729 | -80.4078 | Virginia, Montgomery | **MN817800** | **MN719646** | **MN817833** | **-** | **MT503356** | **MT349979** |
| *Nannaria ericacea* | MPE00765 | 37.1916 | -80.4577 | Virginia, Montgomery | **MT497574** | **MT515394** | **MT504375** | **MT491258** | **MT665914** | **MT503654** |
| *Nannaria ericacea* | MPE00835 | 37.2824 | -80.4485 | Virginia, Montgomery | **MT497576** | **MT515396** | **MT504377** | **MT491259** | **MT665916** | **MT503656** |
| *Nannaria ericacea* | MPE01850 | 37.8685 | -79.8713 | Virginia, Alleghany | **MT302725** | **MT319938** | **MT504383** | **MN699764** | **MT503384** | **MT350012** |
| *Nannaria ericacea* | MPE02145 | 37.3477 | -80.3251 | Virginia, Montgomery | **MT497582** | **MT515402** | **MT504388** | **MT491266** | **MT665923** | **MT503662** |
| *Nannaria ericacea* | MPE02263 | 37.3599 | -79.9939 | Virginia, Roanoke | **MT497584** | **MT515404** | **MT504390** | **MT491268** | **MT665925** | **MT503664** |
| *Nannaria ericacea* | MPE02434 | 38.0792 | -79.8358 | Virginia, Bath | **MT497586** | **MT515407** | **-** | **-** | **MT665927** | **MT503666** |
| *Nannaria ericacea* | MPE02861 | 37.4256 | -79.7578 | Virginia, Botetourt | **MT497595** | **MT515416** | **-** | **MT491277** | **MT665936** | **MT503675** |
| *Nannaria filicata* sp. nov. | MPE02110 | 37.8526 | -79.8512 | Virginia, Alleghany | **MT302739** | **MT319946** | **MT319853** | **MT491264** | **MT503394** | **MT350022** |
| *Nannaria filicata* sp. nov. | MPE02451 | 37.8579 | -79.8666 | Virginia, Alleghany | **MT497587** | **MT515408** | **-** | **MT491270** | **MT665928** | **MT503667** |
| *Nannaria fowleri* | MPE00433 | 38.6947 | -79.5146 | West Virginia, Pendleton | **MN817801** | **MN719648** | **MN817834** | **MN699765** | **MT503358** | **MT349981** |
| *Nannaria fowleri* | MPE03017 | 39.9079 | -77.4777 | Pennsylvania, Franklin | **MT302716** | **MT319962** | **MT319875** | **-** | **MT503427** | **MT350055** |
| *Nannaria fowleri* | MPE03071 | 41.1034 | -77.2435 | Pennsylvania, Clinton | **MT302717** | **MT319963** | **MT319876** | **MT311431** | **MT503428** | **MT350056** |
| *Nannaria fracta* | MPE03178 | 37.2897 | -82.2999 | Virginia, Dickenson | **MT302667** | **MT319969** | **MT319883** | **-** | **MT503434** | **MT350063** |
| *Nannaria fracta* | MPE03184 | 37.469 | -82.5462 | Kentucky, Pike | **MT302677** | **MT319970** | **MT319884** | **-** | **MT503435** | **MT350064** |
| *Nannaria fritzae* | MPE02359 | 34.5628 | -85.0681 | Georgia, Floyd | **MT302690** | **MT319951** | **MT319861** | **MT311415** | **MT503405** | **MT350033** |
| *Nannaria hardeni* | MPE02278 | 36.5577 | -79.3515 | Virginia, Danville City | **MT302652** | **-** | **MT319859** | **MT311413** | **MT503402** | **MT350030** |
| *Nannaria hippopotamus* | MPE04150 | 36.1059 | -82.6545 | Tennessee, Greene | **MT302680** | **-** | **MT319905** | **-** | **MT503456** | **MT350089** |
| *Nannaria hokie* | MPE00253 | 37.1775 | -80.6456 | Virginia, Pulaski | **-** | **-** | **MT504369** | **-** | **-** | **-** |
| *Nannaria hokie* | MPE00275 | 37.1729 | -80.4078 | Virginia, Montgomery | **MT302645** | **MT319924** | **MT319833** | **MT311381** | **-** | **MT349978** |
| *Nannaria hokie* | MPE00880 | 37.225 | -80.4276 | Virginia, Montgomery | **MN817806** | **-** | **MN817840** | **MN699771** | **MT503362** | **MT349987** |
| *Nannaria honeytreetrailensis* | MMC0334 | 36.6078 | -83.6322 | Virginia, Lee | **MT302693** | **MT319915** | **MT319821** | **MT311370** | **MT503345** | **MT349966** |
| *Nannaria ignis* | MPE00912 | 37.0257 | -81.0901 | Virginia, Wythe | **MT302707** | **-** | **MT319839** | **MT311386** | **MT503365** | **MT349990** |
| *Nannaria ignis* | MPE01063 | 37.0383 | -81.109 | Virginia, Bland | **MT302706** | **-** | **MT319842** | **MT311390** | **MT503370** | **MT349995** |
| *Nannaria ignis* | MPE01198 | 37.0478 | -81.1155 | Virginia, Bland | **MT302688** | **-** | **-** | **MT311392** | **MT503372** | **MT349998** |
| *Nannaria kassoni* | MPE00544 | 36.3072 | -84.226 | Tennessee, Campbell | **MT302703** | **MT319925** | **MT319834** | **MT311382** | **MT503359** | **MT349982** |
| *Nannaria laminata* | MPE02124 | 37.4209 | -80.5093 | Virginia, Giles | **MT302701** | **-** | **-** | **MT311408** | **MT503396** | **MT350024** |
| *Nannaria laminata* | MPE02392 | 37.9139 | -79.0207 | Virginia, Augusta | **MN817802** | **-** | **MN817835** | **MN699766** | **MT503406** | **MT350034** |
| *Nannaria laminata* | MPE02528 | 37.4717 | -80.562 | West Virginia, Monroe | **MT302700** | **-** | **MT319866** | **MT311420** | **MT503411** | **MT350039** |
| *Nannaria liriodendra* sp. nov. | MPE04200 | 37.5833 | -80.1604 | Virginia, Craig | **MT302726** | **MT319984** | **MT319908** | **-** | **MT503459** | **MT350092** |
| *Nannaria lithographa* sp. nov. | MPE03430 | 35.4315 | -82.2435 | North Carolina, Rutherford | **-** | **MT319973** | **MT319887** | **MT491278** | **MT503438** | **MT350067** |
| *Nannaria lutra* sp. nov. | MPE00156 | 37.4581 | -79.6295 | Virginia, Bedford | **MT302730** | **MT319918** | **MT319825** | **MT311374** | **MT503348** | **MT349970** |
| *Nannaria lutra* sp. nov. | MPE00158 | 37.4581 | -79.6295 | Virginia, Bedford | **MT497561** | **MT515385** | **-** | **MT491248** | **MT665904** | **MT503644** |
| *Nannaria lutra* sp. nov. | MPE01933 | 37.4407 | -79.6033 | Virginia, Bedford | **MT497580** | **MT515400** | **MT504384** | **MT491263** | **MT665921** | **MT503660** |
| *Nannaria lutra* sp. nov. | MPE02147 | 37.6185 | -79.4709 | Virginia, Rockbridge | **MT497583** | **MT515403** | **MT504389** | **MT491267** | **MT665924** | **MT503663** |
| *Nannaria marianae* sp. nov. | MPE02900 | 37.9429 | -79.1382 | Virginia, Augusta | **MT302743** | **MT319961** | **MT319873** | **-** | **MT503424** | **MT350052** |
| *Nannaria marianae* sp. nov. | MPE04013 | 37.9256 | -79.1309 | Virginia, Augusta | **MT497605** | **MT515424** | **MT504403** | **MT491286** | **MT665945** | **MT503683** |
| *Nannaria marianae* sp. nov. | MPE05006 | 37.9776 | -78.9938 | Virginia, Augusta | **MT497614** | **MT515433** | **-** | **MT491296** | **MT665953** | **MT503693** |
| *Nannaria mcelroyorum* | MPE02240 | 38.1802 | -81.8384 | West Virginia, Boone | **MT302656** | **-** | **MT319857** | **MT311411** | **MT503400** | **MT350028** |
| *Nannaria mcelroyorum* | MPE03113 | 38.3047 | -82.3512 | West Virginia, Wayne | **MT302654** | **MT319965** | **MT319878** | **MT311433** | **MT503430** | **MT350058** |
| *Nannaria mcelroyorum* | MPE03125 | 38.3475 | -82.6866 | Kentucky, Boyd | **MT302655** | **MT319966** | **MT319879** | **MT311434** | **MT503431** | **MT350059** |
| *Nannaria minor* | MPE01249 | 36.2651 | -82.23 | Tennessee, Carter | **MN817803** | **-** | **MN817836** | **MN699767** | **MT503377** | **MT350003** |
| *Nannaria minor* | MPE01313 | 36.2651 | -82.23 | Tennessee, Carter | **MT302663** | **MT319933** | **MT319845** | **MT311395** | **MT503378** | **MT350004** |
| *Nannaria minor* | MPE04060 | 36.3199 | -82.0834 | Tennessee, Carter | **MT302665** | **MT319982** | **MT319899** | **MT311450** | **-** | **MT350082** |
| *Nannaria minor* | MPE04080 | 36.1674 | -82.0984 | Tennessee, Carter | **MT302664** | **-** | **MT319900** | **MT311451** | **MT503451** | **MT350083** |
| *Nannaria minor* | MPE04100 | 36.277 | -82.3461 | Tennessee, Washington | **-** | **-** | **MT319901** | **MT311453** | **MT503453** | **MT350085** |
| *Nannaria minor* | MPE04103 | 36.1742 | -82.2982 | Tennessee, Unicoi | **MT302662** | **-** | **MT319902** | **MT311454** | **MT503454** | **MT350086** |
| *Nannaria minor* | MPE04107 | 36.1389 | -82.3468 | Tennessee, Unicoi | **MT302678** | **-** | **MT319903** | **MT311455** | **-** | **MT350087** |
| *Nannaria minor* | MPE04133 | 36.0482 | -82.5615 | Tennessee, Unicoi | **MT302679** | **MT319983** | **MT319904** | **MT311456** | **MT503455** | **MT350088** |
| *Nannaria missouriensis* | MPE02800 | 38.6706 | -90.7515 | Missouri, St. Charles | **MT302644** | **MT319958** | **-** | **MT311423** | **MT503418** | **MT350046** |
| *Nannaria monsdomia* | MPE02188 | 36.1032 | -83.7642 | Tennessee, Knox | **MT302691** | **-** | **MT319855** | **MT311409** | **MT503398** | **MT350026** |
| *Nannaria morrisoni* | MPE02015 | 38.1474 | -78.7443 | Virginia, Albemarle | **MN817804** | **MN719665** | **MN817837** | **MN699768** | **MT503386** | **MT350014** |
| *Nannaria morrisoni* | MPE02107 | 38.1474 | -78.7443 | Virginia, Albemarle | **MT302747** | **MT319943** | **MT319850** | **MT311404** | **MT503391** | **MT350019** |
| *Nannaria morrisoni* | MPE02115 | 37.8388 | -79.0213 | Virginia, Nelson | **MT302745** | **MT319947** | **MT504386** | **MT311406** | **MT503395** | **MT350023** |
| *Nannaria morrisoni* | MPE02491 | 37.7547 | -79.185 | Virginia, Amherst | **MT302746** | **MT319954** | **MT319864** | **MT311418** | **MT503409** | **MT350037** |
| *Nannaria morrisoni* | MPE02591 | 38.385 | -78.5147 | Virginia, Greene | **MT497592** | **MT515413** | **MT504394** | **MT491273** | **MT665933** | **MT503672** |
| *Nannaria morrisoni* | MPE02595 | 38.3802 | -78.5036 | Virginia, Greene | **MT302748** | **MT319955** | **MT319867** | **MT311421** | **MT503412** | **MT350040** |
| *Nannaria morrisoni* | MPE02605 | 38.3791 | -78.4997 | Virginia, Greene | **MT497593** | **MT515414** | **MT504395** | **MT491274** | **MT665934** | **MT503673** |
| *Nannaria morrisoni* | MPE02872 | 37.8425 | -79.1165 | Virginia, Nelson | **MT302744** | **MT319960** | **MT319870** | **MT311426** | **MT503421** | **MT350049** |
| *Nannaria morrisoni* | MPE03669 | 38.382 | -78.5097 | Virginia, Greene | **MT497598** | **MT515417** | **-** | **MT491280** | **MT665938** | **MT503676** |
| *Nannaria morrisoni* | SPC000495 | 38.0966 | -78.7779 | Virginia, Albemarle | **MT497616** | **MT515435** | **MT504412** | **MT491298** | **MT665955** | **-** |
| *Nannaria nessa* sp. nov. | MPE01465 | 35.0288 | -83.2823 | North Carolina, Macon | **MN817798** | **MN719660** | **MN817839** | **MN699770** | **MT503379** | **MT350005** |
| *Nannaria ohionis* | MPE00906 | 39.2107 | -81.8421 | Ohio, Athens | **-** | **-** | **MT319838** | **-** | **MT503363** | **MT349988** |
| *Nannaria ohionis* | MPE00907 | 39.4307 | -81.5003 | Ohio, Washington | **MN817807** | **-** | **MN817841** | **-** | **MT503364** | **MT349989** |
| *Nannaria ohionis* | MPE03643 | 39.242 | -81.2991 | West Virginia, Wood | **MT302712** | **MN719688** | **MT319894** | **MN699772** | **MT503444** | **MT350075** |
| *Nannaria orycta* sp. nov. | MPE03487 | 37.7956 | -79.4427 | Virginia, Rockingham | **-** | **MT319975** | **MT319890** | **MT311439** | **MT665937** | **MT350071** |
| *Nannaria orycta* sp. nov. | MPE03810 | 37.8065 | -79.6152 | Virginia, Rockbridge | **MT497602** | **MT515421** | **MT504400** | **MT491283** | **MT665942** | **MT503680** |
| *Nannaria paupertas* | MPE00108 | 37.2678 | -80.4852 | Virginia, Montgomery | **MT302708** | **MT319917** | **MT319823** | **MT311372** | **MT503346** | **MT349968** |
| *Nannaria piccolia* | MPE03809 | 37.8065 | -79.6152 | Virginia, Rockbridge | **MT302719** | **MT319980** | **MT319896** | **MT311445** | **MT503447** | **MT350077** |
| *Nannaria rhododendra* sp. nov. | MPE04336 | 34.9688 | -83.7733 | Georgia, Towns | **MT497606** | **MT515425** | **MT504404** | **MT491288** | **MT665947** | **MT503685** |
| *Nannaria rhododendra* sp. nov. | MPE04342 | 34.675 | -84.0015 | Georgia, Lumpkin | **MT497607** | **MT515426** | **MT504405** | **MT491289** | **MT665948** | **MT503686** |
| *Nannaria rhododendra* sp. nov. | MPE04348 | 34.9538 | -83.8359 | Georgia, Towns | **MT497609** | **MT515428** | **MT504407** | **MT491291** | **-** | **MT503688** |
| *Nannaria rhododendra* sp. nov. | MPE04363 | 34.7078 | -83.915 | Georgia, Lumpkin | **MT497610** | **MT515429** | **MT504408** | **MT491292** | **-** | **MT503689** |
| *Nannaria rhododendra* sp. nov. | MPE04401 | 34.9256 | -83.7776 | Georgia, Towns | **MT497612** | **MT515431** | **-** | **MT491294** | **MT665951** | **MT503691** |
| *Nannaria scholastica* | MPE03485 | 37.7956 | -79.4427 | Virginia, Rockingham | **MT302720** | **MT319974** | **-** | **MT311438** | **MT503440** | **MT350070** |
| *Nannaria scutellaria* | MPE01474 | 35.636 | -83.4937 | Tennessee, Sevier | **MN817808** | **-** | **MN817842** | **MN699773** | **MT503380** | **MT350006** |
| *Nannaria scutellaria* | MPE02105 | 35.4938 | -83.1557 | North Carolina, Jackson | **-** | **MT319941** | **MT319848** | **MT311402** | **MT503389** | **MT350017** |
| *Nannaria scutellaria* | MPE03745 | 35.0882 | -83.866 | North Carolina, Clay | **MT497601** | **MT515420** | **MT504399** | **-** | **MT665941** | **MT503679** |
| *Nannaria scutellaria* | MPE04305 | 35.3699 | -83.1212 | North Carolina, Jackson | - | - | - | **MT491287** | **MT665946** | **MT503684** |
| *Nannaria scutellaria* | MPE04346 | 35.2851 | -82.8286 | North Carolina, Transylvania | **MT497608** | **MT515427** | **MT504406** | **MT491290** | **MT665949** | **MT503687** |
| *Nannaria serpens* | MPE00202 | 36.7761 | -80.5446 | Virginia, Carroll | **MT302649** | **MT319919** | **MT319827** | **MT311376** | **MT503350** | **MT349972** |
| *Nannaria serpens* | MPE00817 | 36.8132 | -80.3495 | Virginia, Floyd | **MT302650** | **-** | **MT319837** | **MT311385** | **MT503361** | **MT349986** |
| *Nannaria serpens* | MPE02610 | 36.7182 | -80.3246 | Virginia, Patrick | **MT302651** | **MT319956** | **MT319868** | **MT311422** | **MT503413** | **MT350041** |
| *Nannaria sheari* | MPE01684 | 37.4647 | -81.0623 | Virginia, Mercer | **-** | **MT319935** | **-** | **MT311397** | **-** | **MT350008** |
| *Nannaria shenandoa* | MPE00231 | 39.4369 | -79.9852 | West Virginia, Marion | **MT302750** | **MT319920** | **MT319828** | **MT311377** | **MT503351** | **MT349973** |
| *Nannaria shenandoa* | MPE03104 | 37.7748 | -83.6825 | Kentucky, Powell | **MT497596** | **MT319964** | **MT319877** | **MT311432** | **MT503429** | **MT350057** |
| *Nannaria shenandoa* | MPE03994 | 38.9407 | -78.3058 | Virginia, Shenandoah | **MT497603** | **MT515422** | **MT504401** | **MT491284** | **MT665943** | **MT503681** |
| *Nannaria solenas* | MMC0201 | 36.9533 | -82.055 | Virginia, Russell | **MT302673** | **MT319914** | **MT319820** | **MT311369** | **MT503344** | **MT349965** |
| *Nannaria solenas* | MPE00128 | 37.5264 | -80.9879 | West Virginia, Mercer | **MT302674** | **-** | **MT319824** | **MT311373** | **MT503347** | **MT349969** |
| *Nannaria solenas* | MPE02428 | 36.9586 | -81.3189 | Virginia, Wythe | **MT302672** | **MT319953** | **MT319863** | **MT311417** | **MT503408** | **MT350036** |
| *Nannaria spalax* sp. nov. | MPE04377 | 34.8813 | -83.3539 | Georgia, Rabun | **MT497611** | **MT515430** | **MT504409** | **MT491293** | **MT665950** | **MT503690** |
| *Nannaria spiralis* sp. nov. | MPE02109 | 38.5948 | -79.1982 | West Virginia, Pendleton | **MT302740** | **MT319945** | **MT319852** | **MT311405** | **MT503393** | **MT350021** |
| *Nannaria spiralis* sp. nov. | MPE04010 | 38.452 | -79.1144 | Virginia, Rockingham | **MT497604** | **MT515423** | **MT504402** | **MT491285** | **MT665944** | **MT503682** |
| *Nannaria spruilli* | MMC0035 | 36.8949 | -82.5902 | Virginia, Wise | **MT302676** | **MT319913** | **MT319819** | **MT311367** | **MT503342** | **MT349963** |
| *Nannaria stellapolis* | MPE00252 | 37.2309 | -79.9502 | Virginia, Roanoke | **MT302646** | **-** | **MT319831** | **MT311380** | **MT503354** | **MT349976** |
| *Nannaria stellaradix* | MPE00239 | 36.9662 | -80.4179 | Virginia, Montgomery | **MT302647** | **MT319922** | **MT319830** | **MT311379** | **MT503353** | **MT349975** |
| *Nannaria suprema* | MPE00075 | 36.7067 | -81.6028 | Virginia, Smyth | **MT302714** | **MT319916** | **-** | **MT311371** | **-** | **MT349967** |
| *Nannaria swiftae* sp. nov. | MPE01222 | 35.6628 | -85.3498 | Tennessee, Van Buren | **MT302723** | **MT319931** | **MT504380** | **-** | **MT503373** | **MT349999** |
| *Nannaria swiftae* sp. nov. | MPE01226 | 35.6612 | -85.3464 | Tennessee, Van Buren | **MT497578** | **MT515399** | **MT504381** | **MT491262** | **MT665920** | **MT503659** |
| *Nannaria tasskelsoae* | MPE00578 | 38.1101 | -80.2733 | West Virginia, Hillsboro | **MT302694** | **MT319926** | **MT319835** | **-** | **-** | **MT349983** |
| *Nannaria tasskelsoae* | MPE04023 | 38.2498 | -80.4455 | West Virginia, Greenbrier | **MT302696** | **-** | **-** | **MT311446** | **MT503448** | **MT350078** |
| *Nannaria tasskelsoae* | SPC000710 | 38.249 | -80.4437 | West Virginia, Greenbrier | **MT302695** | **MT319987** | **-** | **MT311460** | **MT503463** | **-** |
| *Nannaria tennesseensis* | MPE01237 | 36.1515 | -83.517 | Tennessee, Jefferson | **MN817809** | **MN719652** | **MT319822** | **MN699774** | **MT503376** | **MT350002** |
| *Nannaria tenuis* | MPE00925 | 37.1407 | -81.1394 | Virginia, Bland | **-** | **-** | **MT319840** | **MT311387** | **MT503366** | **MT349991** |
| *Nannaria tenuis* | MPE01111 | 37.1234 | -81.1355 | Virginia, Bland | **MT302686** | **-** | **MT319843** | **MT311391** | **MT665919** | **MT349996** |
| *Nannaria terricola* | MPE01691 | 39.4913 | -82.5797 | Ohio, Hocking | **MT302669** | **MT319937** | **-** | **-** | **-** | **MT350010** |
| *Nannaria terricola* | MPE01738 | 38.7213 | -83.4335 | Ohio, Adams | **MN817810** | **MN719664** | **MN817843** | **MN699775** | **MT503383** | **MT350011** |
| *Nannaria tsuga* | MPE04047 | 36.5703 | -82.2356 | Tennessee, Sullivan | **MT302666** | **MT319981** | **MT319897** | **MT311448** | **MT503449** | **MT350080** |
| *Nannaria vellicata* sp. nov. | MPE02060 | 38.3605 | -79.2048 | Virginia, Augusta | **MT302741** | **-** | **MT504385** | **MT311401** | **MT503388** | **MT350016** |
| *Nannaria vellicata* sp. nov. | MPE03492 | 37.9021 | -79.5882 | Virginia, Rockbridge | **MT302738** | **MT319976** | **MT319891** | **MT491279** | **MT503441** | **MT350072** |
| *Nannaria wilsoni* | MPE01149 | 37.38910 | -80.5052 | Virginia, Giles | **MT302727** | **MT319930** | **MT504379** | **-** | **MT503371** | **MT349997** |
| *Nannaria wilsoni* | MPE02123 | 37.3892 | -80.5057 | Virginia, Giles | **MT497581** | **MT515401** | **MT504387** | **MT491265** | **MT665922** | **MT503661** |
| *Nannaria wilsoni* | MPE02132 | 37.3892 | -80.5057 | Virginia, Giles | **MN817811** | **MN719667** | **MN817844** | **MN699776** | **MT503397** | **MT350025** |
| *Nannaria wilsoni* | MPE02480 | 37.3751 | -80.5218 | Virginia, Giles | **MT497588** | **MT515409** | **MT504392** | **MT491271** | **MT665929** | **MT503668** |
| *Oenomaea pulchella* | MPE02353 | 36.2519 | -83.0847 | Tennessee, Hawkins | **-** | **MT515405** | **MT504391** | **-** | **MT503404** | **MT350032** |
