# Supplementary figures and images for: ﻿A revision of the wilsoni species group in the millipede genus Nannaria Chamberlin, 1918 (Diplopoda, Polydesmida, Xystodesmidae)
Source: Zookeys. 2022 Apr 15;1096:17–118. doi: 10.3897/zookeys.1096.73485 (PMC9033750; doi:10.3897/zookeys.1096.73485)

0.05 base substitutions  
per site

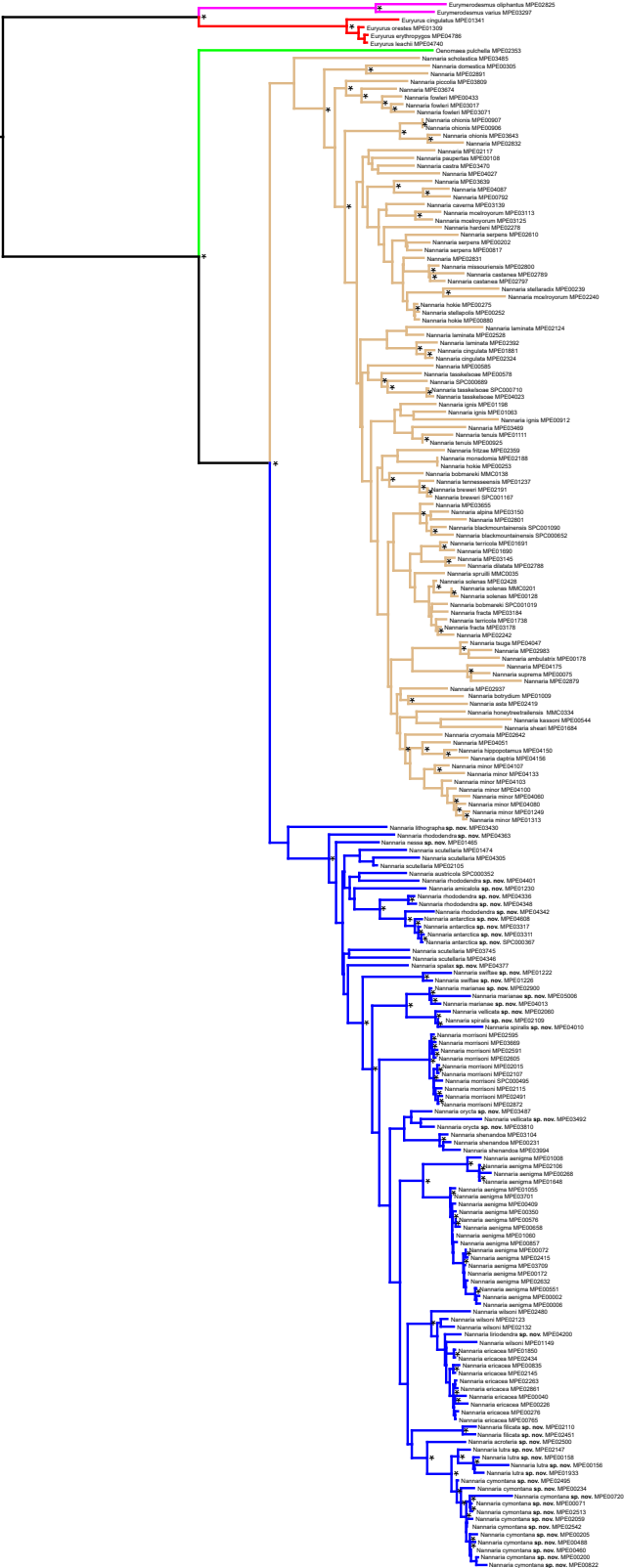

Supplement: Supplementary material 6 — Molecular phylogeny of Nannaria and outgroups [file zookeys-1096-017-s006.pdf]
