# Supplementary material for: ﻿A revision of the wilsoni species group in the millipede genus Nannaria Chamberlin, 1918 (Diplopoda, Polydesmida, Xystodesmidae)
Source: Zookeys. 2022 Apr 15;1096:17–118. doi: 10.3897/zookeys.1096.73485 (PMC9033750; doi:10.3897/zookeys.1096.73485)
Supplement: Supplementary material 7 — Individual gene trees [file zookeys-1096-017-s007.pdf]

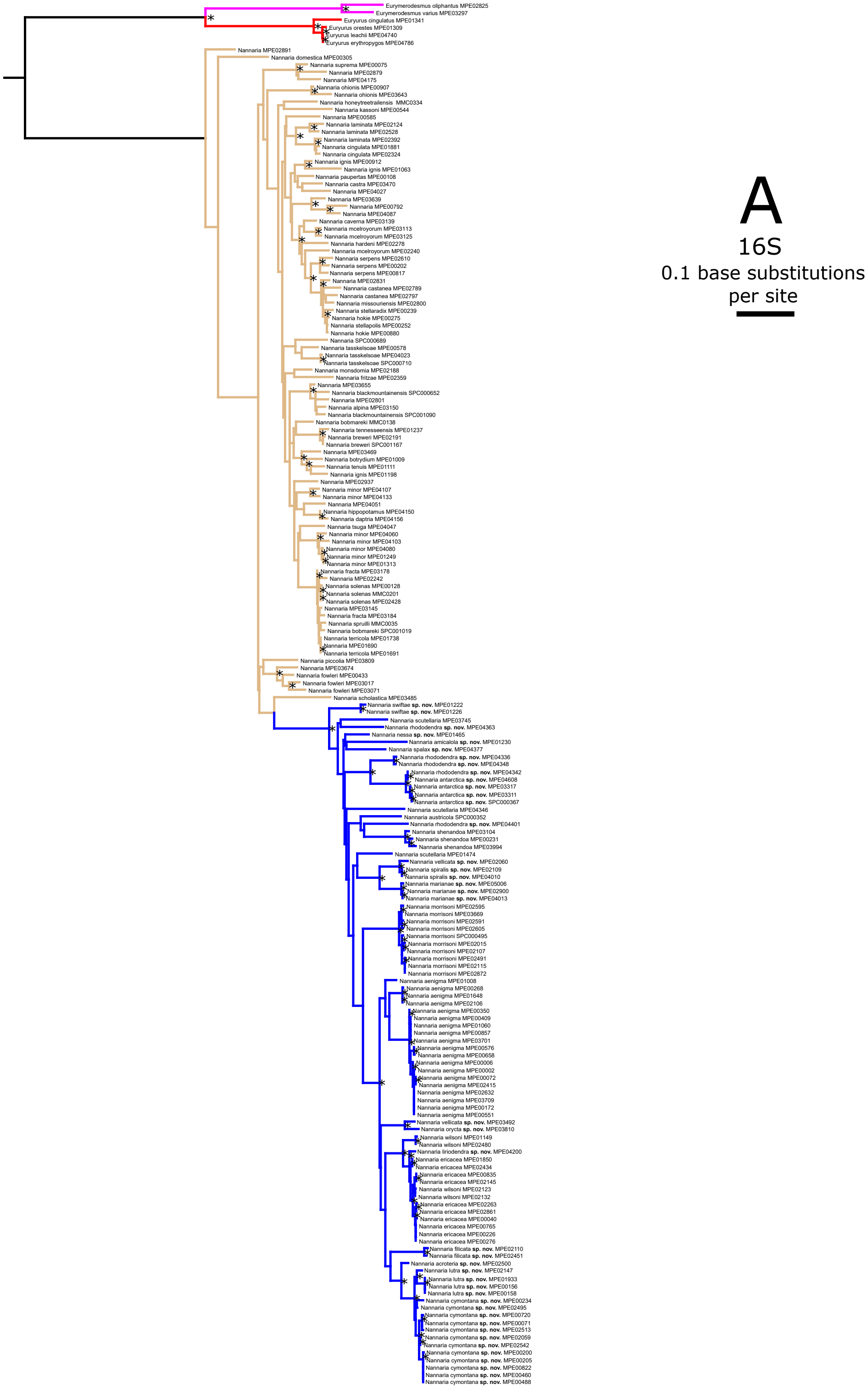
Eurymerodesmus oliphantus MPE02825
Eurymerodesmus varius MPE03297
Euryurus cingulatus MPE01341
Euryurus orestes MPE01309
Euryurus leachii MPE04740
Euryurus erythropygos MPE04786
Nannaria MPE02891
Nannaria domestica MPE00305
Nannaria suprema MPE00075
Nannaria MPE02879
Nannaria MPE04175
Nannaria ohionis MPE00907
Nannaria ohionis MPE03643
Nannaria honeytreetrailensis MMC0334
Nannaria kassoni MPE00544
Nannaria MPE00585
Nannaria laminata MPE02124
Nannaria laminata MPE02528
Nannaria laminata MPE02392
Nannaria cingulata MPE01881
Nannaria cingulata MPE02324
Nannaria ignis MPE00912
Nannaria ignis MPE01063
Nannaria paupertas MPE00108
Nannaria castra MPE03470
Nannaria MPE04027
Nannaria MPE03639
Nannaria MPE00792
Nannaria MPE04087
Nannaria caverna MPE03139
Nannaria mcelroyorum MPE03113
Nannaria mcelroyorum MPE03125
Nannaria hardeni MPE02278
Nannaria mcelroyorum MPE02240
Nannaria serpens MPE02610
Nannaria serpens MPE00202
Nannaria serpens MPE00817
Nannaria MPE02831
Nannaria castanea MPE02789
Nannaria castanea MPE02797
Nannaria missouriensis MPE02800
Nannaria stellaradix MPE00239
Nannaria hokie MPE00275
Nannaria stellapolis MPE00252
Nannaria hokie MPE00880
Nannaria SPC000689
Nannaria tasskelsoae MPE00578
Nannaria tasskelsoae MPE04023
Nannaria tasskelsoae SPC000710
Nannaria monsdomia MPE02188
Nannaria fritzae MPE02359
Nannaria MPE03655
Nannaria blackmountainensis SPC000652
Nannaria MPE02801
Nannaria alpina MPE03150
Nannaria blackmountainensis SPC001090
Nannaria bobmareki MMC0138
Nannaria tennesseensis MPE01237
Nannaria breweri MPE02191
Nannaria breweri SPC001167
Nannaria MPE03469
Nannaria botrydium MPE01009
Nannaria tenuis MPE01111
Nannaria ignis MPE01198
Nannaria MPE02937
Nannaria minor MPE04107
Nannaria minor MPE04133
Nannaria MPE04051
Nannaria hippopotamus MPE04150
Nannaria daptria MPE04156
Nannaria tsuga MPE04047
Nannaria minor MPE04060
Nannaria minor MPE04103
Nannaria minor MPE04080
Nannaria minor MPE01249
Nannaria minor MPE01313
Nannaria fracta MPE03178
Nannaria MPE02242
Nannaria solenas MPE00128
Nannaria solenas MMC0201
Nannaria solenas MPE02428
Nannaria MPE03145
Nannaria fracta MPE03184
Nannaria spruilli MMC0035
Nannaria bobmareki SPC001019
Nannaria terricola MPE01738
Nannaria MPE01690
Nannaria terricola MPE01691
Nannaria piccolia MPE03809
Nannaria MPE03674
Nannaria fowleri MPE00433
Nannaria fowleri MPE03017
Nannaria fowleri MPE03071
Nannaria scholastica MPE03485
Nannaria swiftae **sp. nov.** MPE01222
Nannaria swiftae **sp. nov.** MPE01226
Nannaria scutellaria MPE03745
Nannaria rhododendra **sp. nov.** MPE04363
Nannaria nessa **sp. nov.** MPE01465
Nannaria amicalola **sp. nov.** MPE01230
Nannaria spalax **sp. nov.** MPE04377
Nannaria rhododendra **sp. nov.** MPE04336
Nannaria rhododendra **sp. nov.** MPE04348
Nannaria rhododendra **sp. nov.** MPE04342
Nannaria antarctica **sp. nov.** MPE04608
Nannaria antarctica **sp. nov.** MPE03317
Nannaria antarctica **sp. nov.** MPE03311
Nannaria antarctica **sp. nov.** SPC000367
Nannaria scutellaria MPE04346
Nannaria austricola SPC000352
Nannaria rhododendra **sp. nov.** MPE04401
Nannaria shenandoa MPE03104
Nannaria shenandoa MPE00231
Nannaria shenandoa MPE03994
Nannaria scutellaria MPE01474
Nannaria vellicata **sp. nov.** MPE02060
Nannaria spiralis **sp. nov.** MPE02109
Nannaria spiralis **sp. nov.** MPE04010
Nannaria marianae **sp. nov.** MPE05006
Nannaria marianae **sp. nov.** MPE02900
Nannaria marianae **sp. nov.** MPE04013
Nannaria morrisoni MPE02595
Nannaria morrisoni MPE03669
Nannaria morrisoni MPE02591
Nannaria morrisoni MPE02605
Nannaria morrisoni SPC000495
Nannaria morrisoni MPE02015
Nannaria morrisoni MPE02107
Nannaria morrisoni MPE02491
Nannaria morrisoni MPE02115
Nannaria morrisoni MPE02872
Nannaria aenigma MPE01008
Nannaria aenigma MPE00268
Nannaria aenigma MPE01648
Nannaria aenigma MPE02106
Nannaria aenigma MPE00350
Nannaria aenigma MPE00409
Nannaria aenigma MPE01060
Nannaria aenigma MPE00857
Nannaria aenigma MPE03701
Nannaria aenigma MPE00576
Nannaria aenigma MPE00658
Nannaria aenigma MPE00006
Nannaria aenigma MPE00002
Nannaria aenigma MPE00072
Nannaria aenigma MPE02415
Nannaria aenigma MPE02632
Nannaria aenigma MPE03709
Nannaria aenigma MPE00172
Nannaria aenigma MPE00551
Nannaria vellicata **sp. nov.** MPE03492
Nannaria orycta **sp. nov.** MPE03810
Nannaria wilsoni MPE01149
Nannaria wilsoni MPE02480
Nannaria liriodendra **sp. nov.** MPE04200
Nannaria ericacea MPE01850
Nannaria ericacea MPE02434
Nannaria ericacea MPE00835
Nannaria ericacea MPE02145
Nannaria wilsoni MPE02123
Nannaria wilsoni MPE02132
Nannaria ericacea MPE02263
Nannaria ericacea MPE02861
Nannaria ericacea MPE00040
Nannaria ericacea MPE00765
Nannaria ericacea MPE00226
Nannaria ericacea MPE00276
Nannaria filicata **sp. nov.** MPE02110
Nannaria filicata **sp. nov.** MPE02451
Nannaria acroteria **sp. nov.** MPE02500
Nannaria lutra **sp. nov.** MPE02147
Nannaria lutra **sp. nov.** MPE01933
Nannaria lutra **sp. nov.** MPE00156
Nannaria lutra **sp. nov.** MPE00158
Nannaria cymontana **sp. nov.** MPE00234
Nannaria cymontana **sp. nov.** MPE02495
Nannaria cymontana **sp. nov.** MPE00720
Nannaria cymontana **sp. nov.** MPE00071
Nannaria cymontana **sp. nov.** MPE02513
Nannaria cymontana **sp. nov.** MPE02059
Nannaria cymontana **sp. nov.** MPE02542
Nannaria cymontana **sp. nov.** MPE00200
Nannaria cymontana **sp. nov.** MPE00205
Nannaria cymontana **sp. nov.** MPE00822
Nannaria cymontana **sp. nov.** MPE00460
Nannaria cymontana **sp. nov.** MPE00488
A
16S
0.1 base substitutions
per site

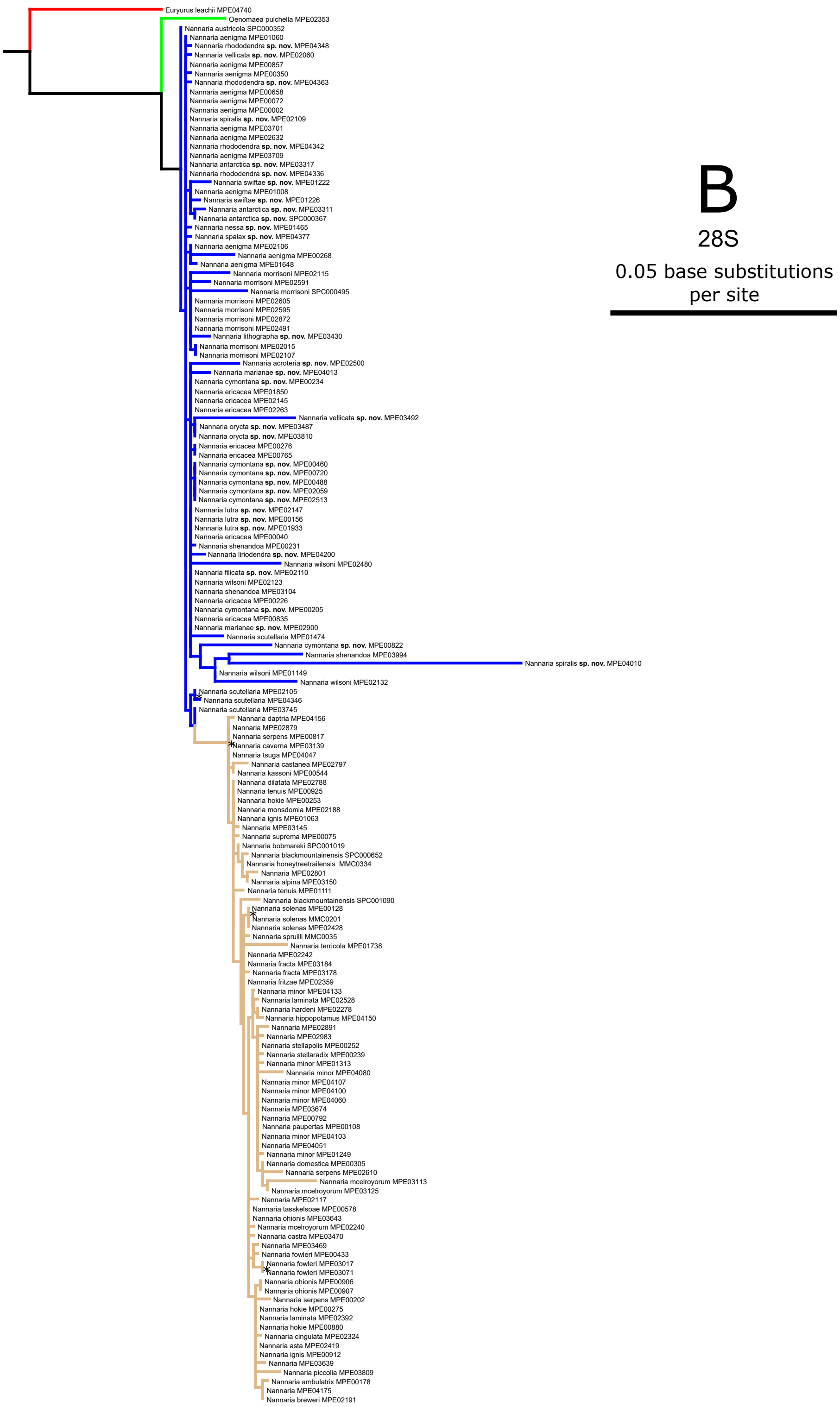
B
28S
0.05 base substitutions per site
Euryurus leachii MPE04740
Oenomaea pulchella MPE02353
Nannaria austricola SPC000352
Nannaria aenigma MPE01060
Nannaria rhododendra sp. nov. MPE04348
Nannaria vellicata sp. nov. MPE02060
Nannaria aenigma MPE00857
Nannaria aenigma MPE00350
Nannaria rhododendra sp. nov. MPE04363
Nannaria aenigma MPE00658
Nannaria aenigma MPE00072
Nannaria aenigma MPE00002
Nannaria spiralis sp. nov. MPE02109
Nannaria aenigma MPE03701
Nannaria aenigma MPE02632
Nannaria rhododendra sp. nov. MPE04342
Nannaria aenigma MPE03709
Nannaria antarctica sp. nov. MPE03317
Nannaria rhododendra sp. nov. MPE04336
Nannaria swiftae sp. nov. MPE01222
Nannaria aenigma MPE01008
Nannaria swiftae sp. nov. MPE01226
Nannaria antarctica sp. nov. MPE03311
Nannaria antarctica sp. nov. SPC000367
Nannaria nessa sp. nov. MPE01465
Nannaria spalax sp. nov. MPE04377
Nannaria aenigma MPE02106
Nannaria aenigma MPE00268
Nannaria aenigma MPE01648
Nannaria morrisoni MPE02115
Nannaria morrisoni MPE02591
Nannaria morrisoni SPC000495
Nannaria morrisoni MPE02605
Nannaria morrisoni MPE02595
Nannaria morrisoni MPE02872
Nannaria morrisoni MPE02491
Nannaria lithographa sp. nov. MPE03430
Nannaria morrisoni MPE02015
Nannaria morrisoni MPE02107
Nannaria acroteria sp. nov. MPE02500
Nannaria marianae sp. nov. MPE04013
Nannaria cymontana sp. nov. MPE00234
Nannaria ericacea MPE01850
Nannaria ericacea MPE02145
Nannaria ericacea MPE02263
Nannaria vellicata sp. nov. MPE03492
Nannaria orycta sp. nov. MPE03487
Nannaria orycta sp. nov. MPE03810
Nannaria ericacea MPE00276
Nannaria ericacea MPE00765
Nannaria cymontana sp. nov. MPE00460
Nannaria cymontana sp. nov. MPE00720
Nannaria cymontana sp. nov. MPE00488
Nannaria cymontana sp. nov. MPE02059
Nannaria cymontana sp. nov. MPE02513
Nannaria lutra sp. nov. MPE02147
Nannaria lutra sp. nov. MPE00156
Nannaria lutra sp. nov. MPE01933
Nannaria ericacea MPE00040
Nannaria shenandoa MPE00231
Nannaria liriodendra sp. nov. MPE04200
Nannaria wilsoni MPE02480
Nannaria filicata sp. nov. MPE02110
Nannaria wilsoni MPE02123
Nannaria shenandoa MPE03104
Nannaria ericacea MPE00226
Nannaria cymontana sp. nov. MPE00205
Nannaria ericacea MPE00835
Nannaria marianae sp. nov. MPE02900
Nannaria scutellaria MPE01474
Nannaria cymontana sp. nov. MPE00822
Nannaria shenandoa MPE03994
Nannaria spiralis sp. nov. MPE04010
Nannaria wilsoni MPE01149
Nannaria wilsoni MPE02132
Nannaria scutellaria MPE02105
Nannaria scutellaria MPE04346
Nannaria scutellaria MPE03745
Nannaria daptria MPE04156
Nannaria MPE02879
Nannaria serpens MPE00817
Nannaria caverna MPE03139
Nannaria tsuga MPE04047
Nannaria castanea MPE02797
Nannaria kassoni MPE00544
Nannaria dilatata MPE02788
Nannaria tenuis MPE00925
Nannaria hokie MPE00253
Nannaria monsdomia MPE02188
Nannaria ignis MPE01063
Nannaria MPE03145
Nannaria suprema MPE00075
Nannaria bobmareki SPC001019
Nannaria blackmountainensis SPC000652
Nannaria honeytreetrailensis MMC0334
Nannaria MPE02801
Nannaria alpina MPE03150
Nannaria tenuis MPE01111
Nannaria blackmountainensis SPC001090
Nannaria solenas MPE00128
Nannaria solenas MMC0201
Nannaria solenas MPE02428
Nannaria spruilli MMC0035
Nannaria terricola MPE01738
Nannaria MPE02242
Nannaria fracta MPE03184
Nannaria fracta MPE03178
Nannaria fritzae MPE02359
Nannaria minor MPE04133
Nannaria laminata MPE02528
Nannaria hardeni MPE02278
Nannaria hippopotamus MPE04150
Nannaria MPE02891
Nannaria MPE02983
Nannaria stellapolis MPE00252
Nannaria stellaradix MPE00239
Nannaria minor MPE01313
Nannaria minor MPE04080
Nannaria minor MPE04107
Nannaria minor MPE04100
Nannaria minor MPE04060
Nannaria MPE03674
Nannaria MPE00792
Nannaria paupertas MPE00108
Nannaria minor MPE04103
Nannaria MPE04051
Nannaria minor MPE01249
Nannaria domestica MPE00305
Nannaria serpens MPE02610
Nannaria mcelroyorum MPE03113
Nannaria mcelroyorum MPE03125
Nannaria MPE02117
Nannaria tasskelsoae MPE00578
Nannaria ohionis MPE03643
Nannaria mcelroyorum MPE02240
Nannaria castra MPE03470
Nannaria MPE03469
Nannaria fowleri MPE00433
Nannaria fowleri MPE03017
Nannaria fowleri MPE03071
Nannaria ohionis MPE00906
Nannaria ohionis MPE00907
Nannaria serpens MPE00202
Nannaria hokie MPE00275
Nannaria laminata MPE02392
Nannaria hokie MPE00880
Nannaria cingulata MPE02324
Nannaria asta MPE02419
Nannaria ignis MPE00912
Nannaria MPE03639
Nannaria piccolia MPE03809
Nannaria ambulatrix MPE00178
Nannaria MPE04175
Nannaria breweri MPE02191

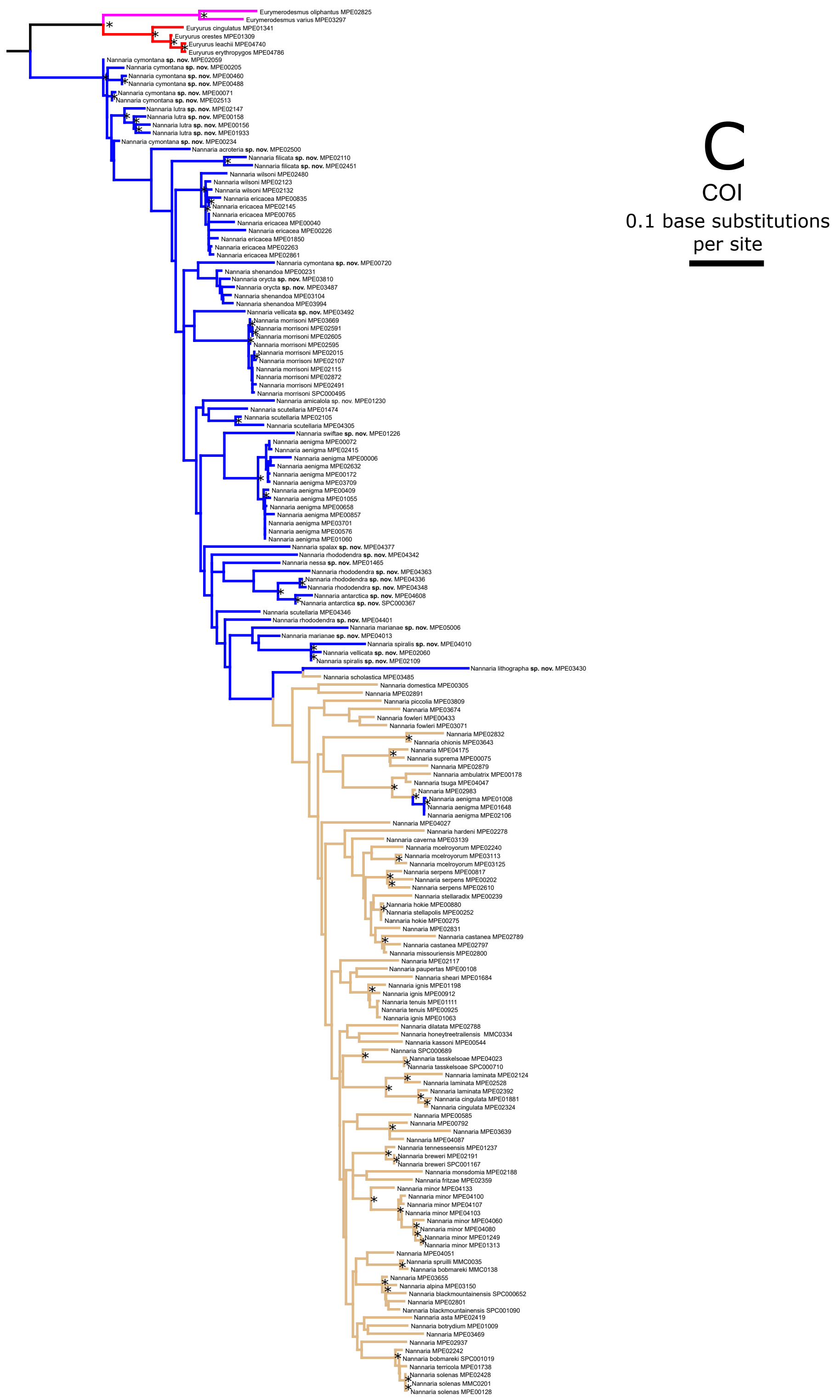
C
COI
0.1 base substitutions per site
Eurymerodesmus oliphantus MPE02825
Eurymerodesmus varius MPE03297
Euryurus cingulatus MPE01341
Euryurus orestes MPE01309
Euryurus leachii MPE04740
Euryurus erythropygos MPE04786
Nannaria cymontana sp. nov. MPE02059
Nannaria cymontana sp. nov. MPE00205
Nannaria cymontana sp. nov. MPE00460
Nannaria cymontana sp. nov. MPE00488
Nannaria cymontana sp. nov. MPE00071
Nannaria cymontana sp. nov. MPE02513
Nannaria lutra sp. nov. MPE02147
Nannaria lutra sp. nov. MPE00158
Nannaria lutra sp. nov. MPE00156
Nannaria lutra sp. nov. MPE01933
Nannaria cymontana sp. nov. MPE00234
Nannaria acroteria sp. nov. MPE02500
Nannaria filicata sp. nov. MPE02110
Nannaria filicata sp. nov. MPE02451
Nannaria wilsoni MPE02480
Nannaria wilsoni MPE02123
Nannaria wilsoni MPE02132
Nannaria ericacea MPE00835
Nannaria ericacea MPE02145
Nannaria ericacea MPE00765
Nannaria ericacea MPE00040
Nannaria ericacea MPE00226
Nannaria ericacea MPE01850
Nannaria ericacea MPE02263
Nannaria ericacea MPE02861
Nannaria cymontana sp. nov. MPE00720
Nannaria shenandoa MPE00231
Nannaria orycta sp. nov. MPE03810
Nannaria orycta sp. nov. MPE03487
Nannaria shenandoa MPE03104
Nannaria shenandoa MPE03994
Nannaria vellicata sp. nov. MPE03492
Nannaria morrisoni MPE03669
Nannaria morrisoni MPE02591
Nannaria morrisoni MPE02605
Nannaria morrisoni MPE02595
Nannaria morrisoni MPE02015
Nannaria morrisoni MPE02107
Nannaria morrisoni MPE02115
Nannaria morrisoni MPE02872
Nannaria morrisoni MPE02491
Nannaria morrisoni SPC000495
Nannaria amicalola sp. nov. MPE01230
Nannaria scutellaria MPE01474
Nannaria scutellaria MPE02105
Nannaria scutellaria MPE04305
Nannaria swiftae sp. nov. MPE01226
Nannaria aenigma MPE00072
Nannaria aenigma MPE02415
Nannaria aenigma MPE00006
Nannaria aenigma MPE02632
Nannaria aenigma MPE00172
Nannaria aenigma MPE03709
Nannaria aenigma MPE00409
Nannaria aenigma MPE01055
Nannaria aenigma MPE00658
Nannaria aenigma MPE00857
Nannaria aenigma MPE03701
Nannaria aenigma MPE00576
Nannaria aenigma MPE01060
Nannaria spalax sp. nov. MPE04377
Nannaria rhododendra sp. nov. MPE04342
Nannaria nessa sp. nov. MPE01465
Nannaria rhododendra sp. nov. MPE04363
Nannaria rhododendra sp. nov. MPE04336
Nannaria rhododendra sp. nov. MPE04348
Nannaria antarctica sp. nov. MPE04608
Nannaria antarctica sp. nov. SPC000367
Nannaria scutellaria MPE04346
Nannaria rhododendra sp. nov. MPE04401
Nannaria marianae sp. nov. MPE05006
Nannaria marianae sp. nov. MPE04013
Nannaria spiralis sp. nov. MPE04010
Nannaria vellicata sp. nov. MPE02060
Nannaria spiralis sp. nov. MPE02109
Nannaria lithographa sp. nov. MPE03430
Nannaria scholastica MPE03485
Nannaria domestica MPE00305
Nannaria MPE02891
Nannaria piccolia MPE03809
Nannaria MPE03674
Nannaria fowleri MPE00433
Nannaria fowleri MPE03071
Nannaria MPE02832
Nannaria ohionis MPE03643
Nannaria MPE04175
Nannaria suprema MPE00075
Nannaria MPE02879
Nannaria ambulatrix MPE00178
Nannaria tsuga MPE04047
Nannaria MPE02983
Nannaria aenigma MPE01008
Nannaria aenigma MPE01648
Nannaria aenigma MPE02106
Nannaria MPE04027
Nannaria hardeni MPE02278
Nannaria caverna MPE03139
Nannaria mcelroyorum MPE02240
Nannaria mcelroyorum MPE03113
Nannaria mcelroyorum MPE03125
Nannaria serpens MPE00817
Nannaria serpens MPE00202
Nannaria serpens MPE02610
Nannaria stellaradix MPE00239
Nannaria hokie MPE00880
Nannaria stellapolis MPE00252
Nannaria hokie MPE00275
Nannaria MPE02831
Nannaria castanea MPE02789
Nannaria castanea MPE02797
Nannaria missouriensis MPE02800
Nannaria MPE02117
Nannaria paupertas MPE00108
Nannaria sheari MPE01684
Nannaria ignis MPE01198
Nannaria ignis MPE00912
Nannaria tenuis MPE01111
Nannaria tenuis MPE00925
Nannaria ignis MPE01063
Nannaria dilatata MPE02788
Nannaria honeytreetrailensis MMC0334
Nannaria kassoni MPE00544
Nannaria SPC000689
Nannaria tasskelsoae MPE04023
Nannaria tasskelsoae SPC000710
Nannaria laminata MPE02124
Nannaria laminata MPE02528
Nannaria laminata MPE02392
Nannaria cingulata MPE01881
Nannaria cingulata MPE02324
Nannaria MPE00585
Nannaria MPE00792
Nannaria MPE03639
Nannaria MPE04087
Nannaria tennesseensis MPE01237
Nannaria breweri MPE02191
Nannaria breweri SPC001167
Nannaria monsdomia MPE02188
Nannaria fritzae MPE02359
Nannaria minor MPE04133
Nannaria minor MPE04100
Nannaria minor MPE04107
Nannaria minor MPE04103
Nannaria minor MPE04060
Nannaria minor MPE04080
Nannaria minor MPE01249
Nannaria minor MPE01313
Nannaria MPE04051
Nannaria spruilli MMC0035
Nannaria bobmareki MMC0138
Nannaria MPE03655
Nannaria alpina MPE03150
Nannaria blackmountainensis SPC000652
Nannaria MPE02801
Nannaria blackmountainensis SPC001090
Nannaria asta MPE02419
Nannaria botrydium MPE01009
Nannaria MPE03469
Nannaria MPE02937
Nannaria MPE02242
Nannaria bobmareki SPC001019
Nannaria terricola MPE01738
Nannaria solenas MPE02428
Nannaria solenas MMC0201
Nannaria solenas MPE00128

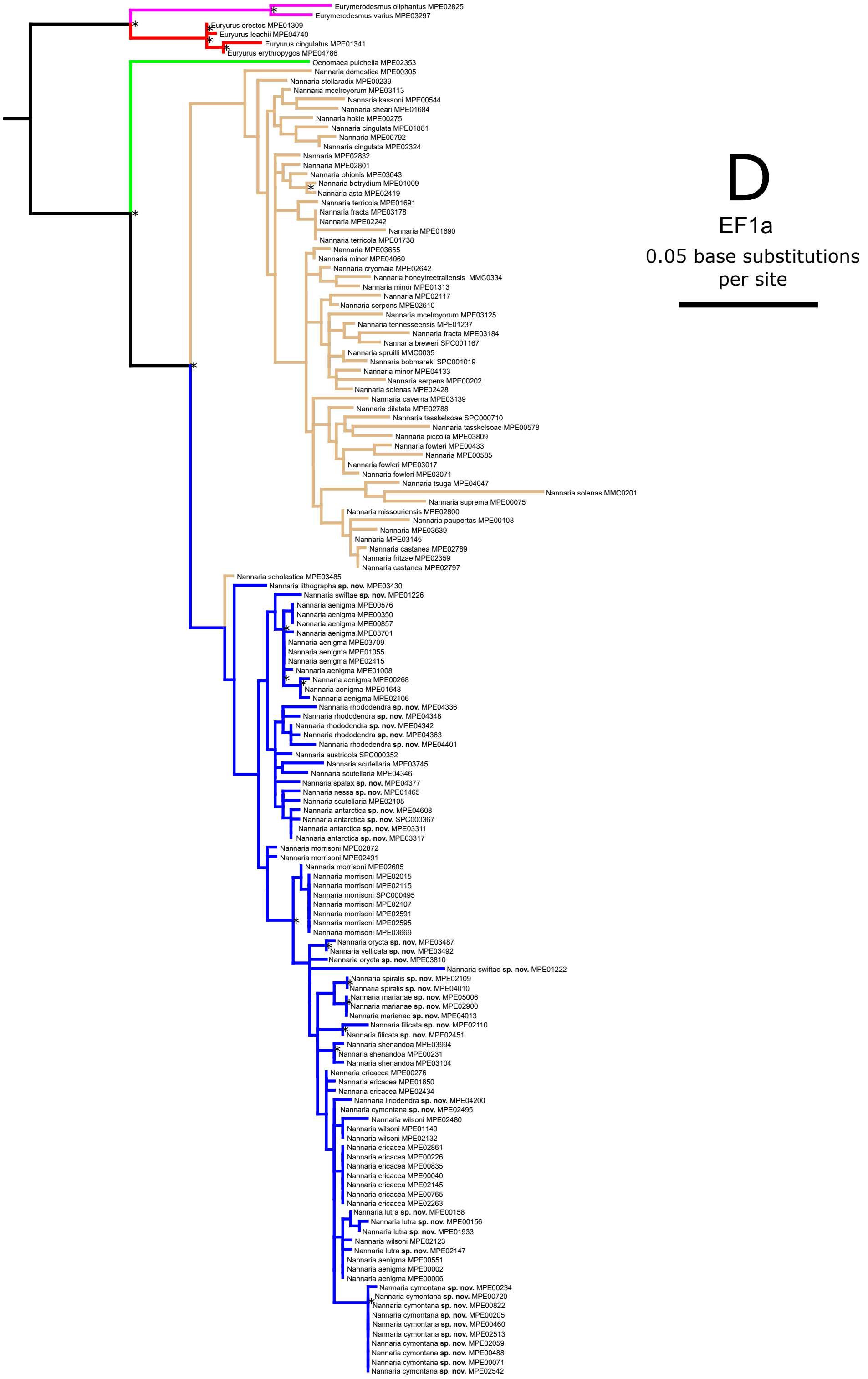

D
EF1a
0.05 base substitutions per site
Eurymerodesmus oliphantus MPE02825
Eurymerodesmus varius MPE03297
Euryurus orestes MPE01309
Euryurus leachii MPE04740
Euryurus cingulatus MPE01341
Euryurus erythropygos MPE04786
Oenomaea pulchella MPE02353
Nannaria domestica MPE00305
Nannaria stellaradix MPE00239
Nannaria mcelroyorum MPE03113
Nannaria kassoni MPE00544
Nannaria sheari MPE01684
Nannaria hokie MPE00275
Nannaria cingulata MPE01881
Nannaria MPE00792
Nannaria cingulata MPE02324
Nannaria MPE02832
Nannaria MPE02801
Nannaria ohionis MPE03643
Nannaria botrydium MPE01009
Nannaria asta MPE02419
Nannaria terricola MPE01691
Nannaria fracta MPE03178
Nannaria MPE02242
Nannaria MPE01690
Nannaria terricola MPE01738
Nannaria MPE03655
Nannaria minor MPE04060
Nannaria cryomaia MPE02642
Nannaria honeytreetrailensis MMC0334
Nannaria minor MPE01313
Nannaria MPE02117
Nannaria serpens MPE02610
Nannaria mcelroyorum MPE03125
Nannaria tennesseensis MPE01237
Nannaria fracta MPE03184
Nannaria breweri SPC001167
Nannaria spruilli MMC0035
Nannaria bobmareki SPC001019
Nannaria minor MPE04133
Nannaria serpens MPE00202
Nannaria solenas MPE02428
Nannaria caverna MPE03139
Nannaria dilatata MPE02788
Nannaria tasskelsoae SPC000710
Nannaria tasskelsoae MPE00578
Nannaria piccolia MPE03809
Nannaria fowleri MPE00433
Nannaria MPE00585
Nannaria fowleri MPE03017
Nannaria fowleri MPE03071
Nannaria tsuga MPE04047
Nannaria solenas MMC0201
Nannaria suprema MPE00075
Nannaria missouriensis MPE02800
Nannaria paupertas MPE00108
Nannaria MPE03639
Nannaria MPE03145
Nannaria castanea MPE02789
Nannaria fritzae MPE02359
Nannaria castanea MPE02797
Nannaria scholastica MPE03485
Nannaria lithographa sp. nov. MPE03430
Nannaria swiftae sp. nov. MPE01226
Nannaria aenigma MPE00576
Nannaria aenigma MPE00350
Nannaria aenigma MPE00857
Nannaria aenigma MPE03701
Nannaria aenigma MPE03709
Nannaria aenigma MPE01055
Nannaria aenigma MPE02415
Nannaria aenigma MPE01008
Nannaria aenigma MPE00268
Nannaria aenigma MPE01648
Nannaria aenigma MPE02106
Nannaria rhododendra sp. nov. MPE04336
Nannaria rhododendra sp. nov. MPE04348
Nannaria rhododendra sp. nov. MPE04342
Nannaria rhododendra sp. nov. MPE04363
Nannaria rhododendra sp. nov. MPE04401
Nannaria austricola SPC000352
Nannaria scutellaria MPE03745
Nannaria scutellaria MPE04346
Nannaria spalax sp. nov. MPE04377
Nannaria nessa sp. nov. MPE01465
Nannaria scutellaria MPE02105
Nannaria antarctica sp. nov. MPE04608
Nannaria antarctica sp. nov. SPC000367
Nannaria antarctica sp. nov. MPE03311
Nannaria antarctica sp. nov. MPE03317
Nannaria morrisoni MPE02872
Nannaria morrisoni MPE02491
Nannaria morrisoni MPE02605
Nannaria morrisoni MPE02015
Nannaria morrisoni MPE02115
Nannaria morrisoni SPC000495
Nannaria morrisoni MPE02107
Nannaria morrisoni MPE02591
Nannaria morrisoni MPE02595
Nannaria morrisoni MPE03669
Nannaria orycta sp. nov. MPE03487
Nannaria vellicata sp. nov. MPE03492
Nannaria orycta sp. nov. MPE03810
Nannaria swiftae sp. nov. MPE01222
Nannaria spiralis sp. nov. MPE02109
Nannaria spiralis sp. nov. MPE04010
Nannaria marianae sp. nov. MPE05006
Nannaria marianae sp. nov. MPE02900
Nannaria marianae sp. nov. MPE04013
Nannaria filicata sp. nov. MPE02110
Nannaria filicata sp. nov. MPE02451
Nannaria shenandoa MPE03994
Nannaria shenandoa MPE00231
Nannaria shenandoa MPE03104
Nannaria ericacea MPE00276
Nannaria ericacea MPE01850
Nannaria ericacea MPE02434
Nannaria liriodendra sp. nov. MPE04200
Nannaria cymontana sp. nov. MPE02495
Nannaria wilsoni MPE02480
Nannaria wilsoni MPE01149
Nannaria wilsoni MPE02132
Nannaria ericacea MPE02861
Nannaria ericacea MPE00226
Nannaria ericacea MPE00835
Nannaria ericacea MPE00040
Nannaria ericacea MPE02145
Nannaria ericacea MPE00765
Nannaria ericacea MPE02263
Nannaria lutra sp. nov. MPE00158
Nannaria lutra sp. nov. MPE00156
Nannaria lutra sp. nov. MPE01933
Nannaria wilsoni MPE02123
Nannaria lutra sp. nov. MPE02147
Nannaria aenigma MPE00551
Nannaria aenigma MPE00002
Nannaria aenigma MPE00006
Nannaria cymontana sp. nov. MPE00234
Nannaria cymontana sp. nov. MPE00720
Nannaria cymontana sp. nov. MPE00822
Nannaria cymontana sp. nov. MPE00205
Nannaria cymontana sp. nov. MPE00460
Nannaria cymontana sp. nov. MPE02513
Nannaria cymontana sp. nov. MPE02059
Nannaria cymontana sp. nov. MPE00488
Nannaria cymontana sp. nov. MPE00071
Nannaria cymontana sp. nov. MPE02542

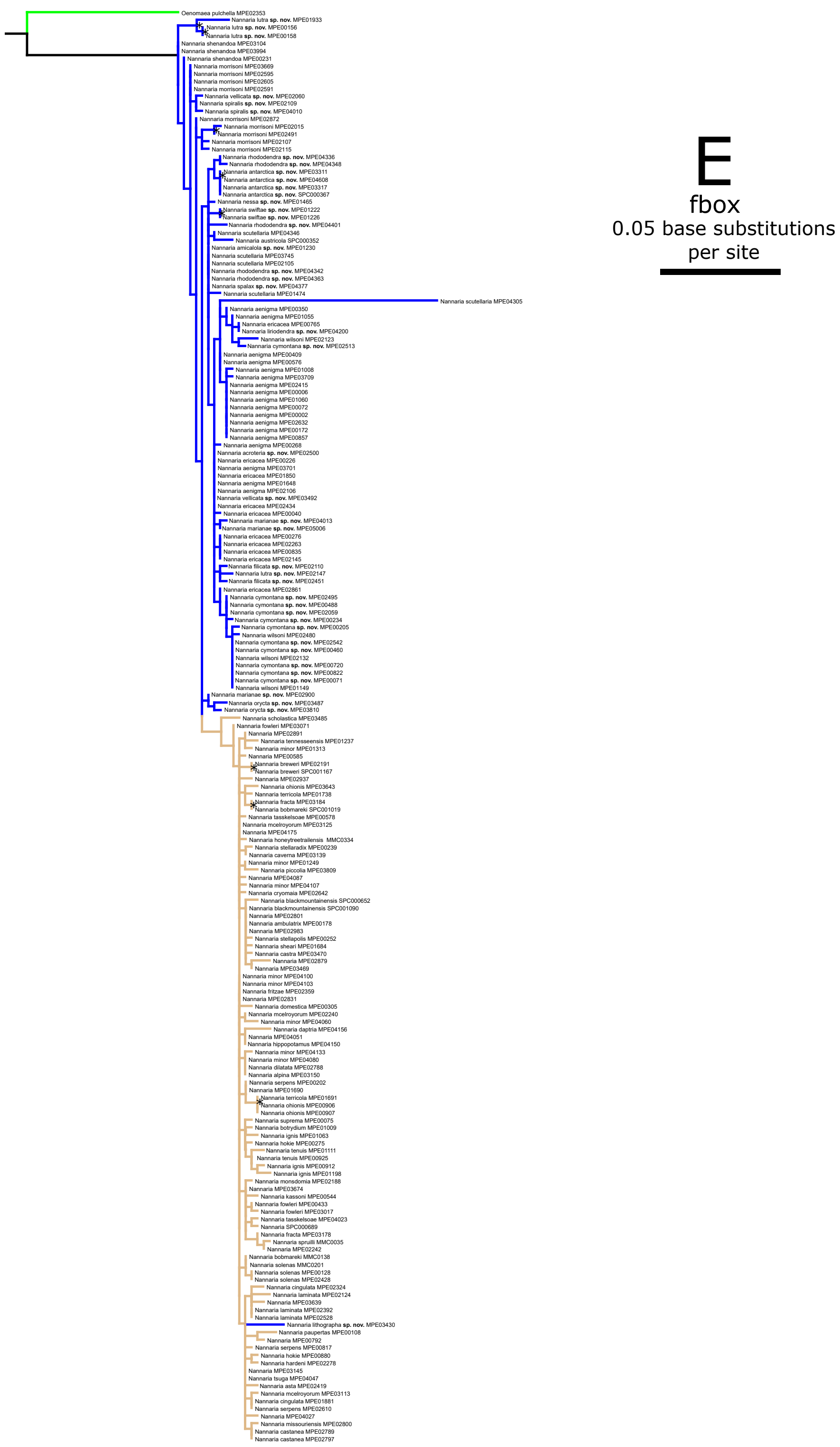
E
fbox
0.05 base substitutions per site
Oenomaea pulchella MPE02353
Nannaria lutra sp. nov. MPE01933
Nannaria lutra sp. nov. MPE00156
Nannaria lutra sp. nov. MPE00158
Nannaria shenandoa MPE03104
Nannaria shenandoa MPE03994
Nannaria shenandoa MPE00231
Nannaria morrisoni MPE03669
Nannaria morrisoni MPE02595
Nannaria morrisoni MPE02605
Nannaria morrisoni MPE02591
Nannaria vellicata sp. nov. MPE02060
Nannaria spiralis sp. nov. MPE02109
Nannaria spiralis sp. nov. MPE04010
Nannaria morrisoni MPE02872
Nannaria morrisoni MPE02015
Nannaria morrisoni MPE02491
Nannaria morrisoni MPE02107
Nannaria morrisoni MPE02115
Nannaria rhododendra sp. nov. MPE04336
Nannaria rhododendra sp. nov. MPE04348
Nannaria antarctica sp. nov. MPE03311
Nannaria antarctica sp. nov. MPE04608
Nannaria antarctica sp. nov. MPE03317
Nannaria antarctica sp. nov. SPC000367
Nannaria nessa sp. nov. MPE01465
Nannaria swiftae sp. nov. MPE01222
Nannaria swiftae sp. nov. MPE01226
Nannaria rhododendra sp. nov. MPE04401
Nannaria scutellaria MPE04346
Nannaria austricola SPC000352
Nannaria amicalola sp. nov. MPE01230
Nannaria scutellaria MPE03745
Nannaria scutellaria MPE02105
Nannaria rhododendra sp. nov. MPE04342
Nannaria rhododendra sp. nov. MPE04363
Nannaria spalax sp. nov. MPE04377
Nannaria scutellaria MPE01474
Nannaria scutellaria MPE04305
Nannaria aenigma MPE00350
Nannaria aenigma MPE01055
Nannaria ericacea MPE00765
Nannaria liriodendra sp. nov. MPE04200
Nannaria wilsoni MPE02123
Nannaria cymontana sp. nov. MPE02513
Nannaria aenigma MPE00409
Nannaria aenigma MPE00576
Nannaria aenigma MPE01008
Nannaria aenigma MPE03709
Nannaria aenigma MPE02415
Nannaria aenigma MPE00006
Nannaria aenigma MPE01060
Nannaria aenigma MPE00072
Nannaria aenigma MPE00002
Nannaria aenigma MPE02632
Nannaria aenigma MPE00172
Nannaria aenigma MPE00857
Nannaria aenigma MPE00268
Nannaria acroteria sp. nov. MPE02500
Nannaria ericacea MPE00226
Nannaria aenigma MPE03701
Nannaria ericacea MPE01850
Nannaria aenigma MPE01648
Nannaria aenigma MPE02106
Nannaria vellicata sp. nov. MPE03492
Nannaria ericacea MPE02434
Nannaria ericacea MPE00040
Nannaria marianae sp. nov. MPE04013
Nannaria marianae sp. nov. MPE05006
Nannaria ericacea MPE00276
Nannaria ericacea MPE02263
Nannaria ericacea MPE00835
Nannaria ericacea MPE02145
Nannaria filicata sp. nov. MPE02110
Nannaria lutra sp. nov. MPE02147
Nannaria filicata sp. nov. MPE02451
Nannaria ericacea MPE02861
Nannaria cymontana sp. nov. MPE02495
Nannaria cymontana sp. nov. MPE00488
Nannaria cymontana sp. nov. MPE02059
Nannaria cymontana sp. nov. MPE00234
Nannaria cymontana sp. nov. MPE00205
Nannaria wilsoni MPE02480
Nannaria cymontana sp. nov. MPE02542
Nannaria cymontana sp. nov. MPE00460
Nannaria wilsoni MPE02132
Nannaria cymontana sp. nov. MPE00720
Nannaria cymontana sp. nov. MPE00822
Nannaria cymontana sp. nov. MPE00071
Nannaria wilsoni MPE01149
Nannaria marianae sp. nov. MPE02900
Nannaria orycta sp. nov. MPE03487
Nannaria orycta sp. nov. MPE03810
Nannaria scholastica MPE03485
Nannaria fowleri MPE03071
Nannaria MPE02891
Nannaria tennesseensis MPE01237
Nannaria minor MPE01313
Nannaria MPE00585
Nannaria breweri MPE02191
Nannaria breweri SPC001167
Nannaria MPE02937
Nannaria ohionis MPE03643
Nannaria terricola MPE01738
Nannaria fracta MPE03184
Nannaria bobmareki SPC001019
Nannaria tasskelsoae MPE00578
Nannaria mcelroyorum MPE03125
Nannaria MPE04175
Nannaria honeytreetrailensis MMC0334
Nannaria stellaradix MPE00239
Nannaria caverna MPE03139
Nannaria minor MPE01249
Nannaria piccolia MPE03809
Nannaria MPE04087
Nannaria minor MPE04107
Nannaria cryomaia MPE02642
Nannaria blackmountainensis SPC000652
Nannaria blackmountainensis SPC001090
Nannaria MPE02801
Nannaria ambulatrix MPE00178
Nannaria MPE02983
Nannaria stellapolis MPE00252
Nannaria sheari MPE01684
Nannaria castra MPE03470
Nannaria MPE02879
Nannaria MPE03469
Nannaria minor MPE04100
Nannaria minor MPE04103
Nannaria fritzae MPE02359
Nannaria MPE02831
Nannaria domestica MPE00305
Nannaria mcelroyorum MPE02240
Nannaria minor MPE04060
Nannaria daptria MPE04156
Nannaria MPE04051
Nannaria hippopotamus MPE04150
Nannaria minor MPE04133
Nannaria minor MPE04080
Nannaria dilatata MPE02788
Nannaria alpina MPE03150
Nannaria serpens MPE00202
Nannaria MPE01690
Nannaria terricola MPE01691
Nannaria ohionis MPE00906
Nannaria ohionis MPE00907
Nannaria suprema MPE00075
Nannaria botrydium MPE01009
Nannaria ignis MPE01063
Nannaria hokie MPE00275
Nannaria tenuis MPE01111
Nannaria tenuis MPE00925
Nannaria ignis MPE00912
Nannaria ignis MPE01198
Nannaria monsdomia MPE02188
Nannaria MPE03674
Nannaria kassoni MPE00544
Nannaria fowleri MPE00433
Nannaria fowleri MPE03017
Nannaria tasskelsoae MPE04023
Nannaria SPC000689
Nannaria fracta MPE03178
Nannaria spruilli MMC0035
Nannaria MPE02242
Nannaria bobmareki MMC0138
Nannaria solenas MMC0201
Nannaria solenas MPE00128
Nannaria solenas MPE02428
Nannaria cingulata MPE02324
Nannaria laminata MPE02124
Nannaria MPE03639
Nannaria laminata MPE02392
Nannaria laminata MPE02528
Nannaria lithographa sp. nov. MPE03430
Nannaria paupertas MPE00108
Nannaria MPE00792
Nannaria serpens MPE00817
Nannaria hokie MPE00880
Nannaria hardeni MPE02278
Nannaria MPE03145
Nannaria tsuga MPE04047
Nannaria asta MPE02419
Nannaria mcelroyorum MPE03113
Nannaria cingulata MPE01881
Nannaria serpens MPE02610
Nannaria MPE04027
Nannaria missouriensis MPE02800
Nannaria castanea MPE02789
Nannaria castanea MPE02797

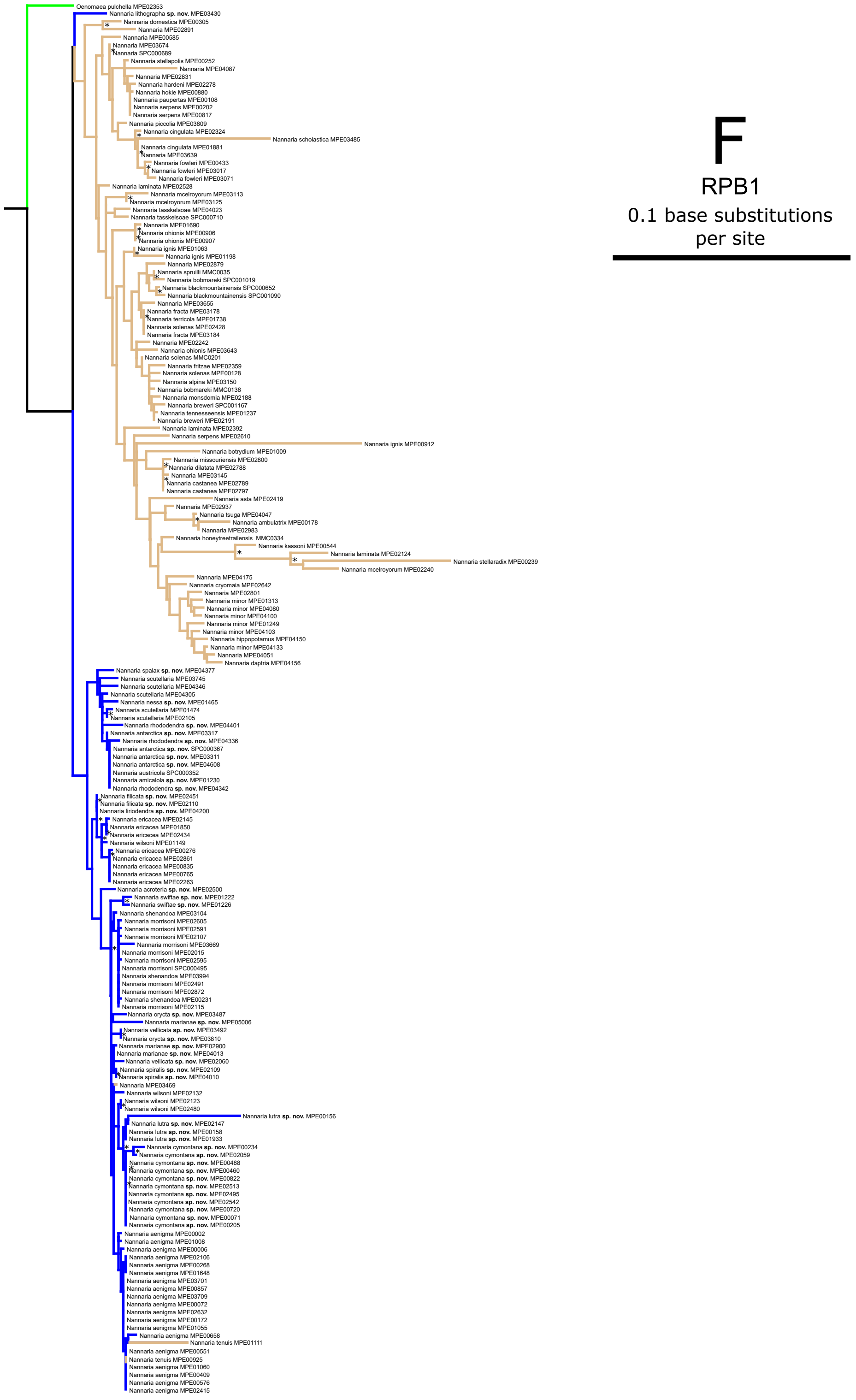
F
RPB1
0.1 base substitutions
per site
Oenomaea pulchella MPE02353
Nannaria lithographa sp. nov. MPE03430
Nannaria domestica MPE00305
Nannaria MPE02891
Nannaria MPE00585
Nannaria MPE03674
Nannaria SPC000689
Nannaria stellapolis MPE00252
Nannaria MPE04087
Nannaria MPE02831
Nannaria hardeni MPE02278
Nannaria hokie MPE00880
Nannaria paupertas MPE00108
Nannaria serpens MPE00202
Nannaria serpens MPE00817
Nannaria piccolia MPE03809
Nannaria cingulata MPE02324
Nannaria scholastica MPE03485
Nannaria cingulata MPE01881
Nannaria MPE03639
Nannaria fowleri MPE00433
Nannaria fowleri MPE03017
Nannaria fowleri MPE03071
Nannaria laminata MPE02528
Nannaria mcelroyorum MPE03113
Nannaria mcelroyorum MPE03125
Nannaria tasskelsoae MPE04023
Nannaria tasskelsoae SPC000710
Nannaria MPE01690
Nannaria ohionis MPE00906
Nannaria ohionis MPE00907
Nannaria ignis MPE01063
Nannaria ignis MPE01198
Nannaria MPE02879
Nannaria spruilli MMC0035
Nannaria bobmareki SPC001019
Nannaria blackmountainensis SPC000652
Nannaria blackmountainensis SPC001090
Nannaria MPE03655
Nannaria fracta MPE03178
Nannaria terricola MPE01738
Nannaria solenas MPE02428
Nannaria fracta MPE03184
Nannaria MPE02242
Nannaria ohionis MPE03643
Nannaria solenas MMC0201
Nannaria fritzae MPE02359
Nannaria solenas MPE00128
Nannaria alpina MPE03150
Nannaria bobmareki MMC0138
Nannaria monsdomia MPE02188
Nannaria breweri SPC001167
Nannaria tennesseensis MPE01237
Nannaria breweri MPE02191
Nannaria laminata MPE02392
Nannaria serpens MPE02610
Nannaria ignis MPE00912
Nannaria botrydium MPE01009
Nannaria missouriensis MPE02800
Nannaria dilatata MPE02788
Nannaria MPE03145
Nannaria castanea MPE02789
Nannaria castanea MPE02797
Nannaria asta MPE02419
Nannaria MPE02937
Nannaria tsuga MPE04047
Nannaria ambulatrix MPE00178
Nannaria MPE02983
Nannaria honeytreetrailensis MMC0334
Nannaria kassoni MPE00544
Nannaria laminata MPE02124
Nannaria stellaradix MPE00239
Nannaria mcelroyorum MPE02240
Nannaria MPE04175
Nannaria cryomaia MPE02642
Nannaria MPE02801
Nannaria minor MPE01313
Nannaria minor MPE04080
Nannaria minor MPE04100
Nannaria minor MPE01249
Nannaria minor MPE04103
Nannaria hippopotamus MPE04150
Nannaria minor MPE04133
Nannaria MPE04051
Nannaria daptria MPE04156
Nannaria spalax sp. nov. MPE04377
Nannaria scutellaria MPE03745
Nannaria scutellaria MPE04346
Nannaria scutellaria MPE04305
Nannaria nessa sp. nov. MPE01465
Nannaria scutellaria MPE01474
Nannaria scutellaria MPE02105
Nannaria rhododendra sp. nov. MPE04401
Nannaria antarctica sp. nov. MPE03317
Nannaria rhododendra sp. nov. MPE04336
Nannaria antarctica sp. nov. SPC000367
Nannaria antarctica sp. nov. MPE03311
Nannaria antarctica sp. nov. MPE04608
Nannaria austricola SPC000352
Nannaria amicalola sp. nov. MPE01230
Nannaria rhododendra sp. nov. MPE04342
Nannaria filicata sp. nov. MPE02451
Nannaria filicata sp. nov. MPE02110
Nannaria liriodendra sp. nov. MPE04200
Nannaria ericacea MPE02145
Nannaria ericacea MPE01850
Nannaria ericacea MPE02434
Nannaria wilsoni MPE01149
Nannaria ericacea MPE00276
Nannaria ericacea MPE02861
Nannaria ericacea MPE00835
Nannaria ericacea MPE00765
Nannaria ericacea MPE02263
Nannaria acroteria sp. nov. MPE02500
Nannaria swiftae sp. nov. MPE01222
Nannaria swiftae sp. nov. MPE01226
Nannaria shenandoa MPE03104
Nannaria morrisoni MPE02605
Nannaria morrisoni MPE02591
Nannaria morrisoni MPE02107
Nannaria morrisoni MPE03669
Nannaria morrisoni MPE02015
Nannaria morrisoni MPE02595
Nannaria morrisoni SPC000495
Nannaria shenandoa MPE03994
Nannaria morrisoni MPE02491
Nannaria morrisoni MPE02872
Nannaria shenandoa MPE00231
Nannaria morrisoni MPE02115
Nannaria orycta sp. nov. MPE03487
Nannaria marianae sp. nov. MPE05006
Nannaria vellicata sp. nov. MPE03492
Nannaria orycta sp. nov. MPE03810
Nannaria marianae sp. nov. MPE02900
Nannaria marianae sp. nov. MPE04013
Nannaria vellicata sp. nov. MPE02060
Nannaria spiralis sp. nov. MPE02109
Nannaria spiralis sp. nov. MPE04010
Nannaria MPE03469
Nannaria wilsoni MPE02132
Nannaria wilsoni MPE02123
Nannaria wilsoni MPE02480
Nannaria lutra sp. nov. MPE00156
Nannaria lutra sp. nov. MPE02147
Nannaria lutra sp. nov. MPE00158
Nannaria lutra sp. nov. MPE01933
Nannaria cymontana sp. nov. MPE00234
Nannaria cymontana sp. nov. MPE02059
Nannaria cymontana sp. nov. MPE00488
Nannaria cymontana sp. nov. MPE00460
Nannaria cymontana sp. nov. MPE00822
Nannaria cymontana sp. nov. MPE02513
Nannaria cymontana sp. nov. MPE02495
Nannaria cymontana sp. nov. MPE02542
Nannaria cymontana sp. nov. MPE00720
Nannaria cymontana sp. nov. MPE00071
Nannaria cymontana sp. nov. MPE00205
Nannaria aenigma MPE00002
Nannaria aenigma MPE01008
Nannaria aenigma MPE00006
Nannaria aenigma MPE02106
Nannaria aenigma MPE00268
Nannaria aenigma MPE01648
Nannaria aenigma MPE03701
Nannaria aenigma MPE00857
Nannaria aenigma MPE03709
Nannaria aenigma MPE00072
Nannaria aenigma MPE02632
Nannaria aenigma MPE00172
Nannaria aenigma MPE01055
Nannaria aenigma MPE00658
Nannaria tenuis MPE01111
Nannaria aenigma MPE00551
Nannaria tenuis MPE00925
Nannaria aenigma MPE01060
Nannaria aenigma MPE00409
Nannaria aenigma MPE00576
Nannaria aenigma MPE02415
